# Predictive models for health outcomes due to SARS-CoV-2, including the effect of vaccination: a systematic review

**DOI:** 10.1186/s13643-023-02411-1

**Published:** 2024-01-16

**Authors:** Oscar Espinosa, Laura Mora, Cristian Sanabria, Antonio Ramos, Duván Rincón, Valeria Bejarano, Jhonathan Rodríguez, Nicolás Barrera, Carlos Álvarez-Moreno, Jorge Cortés, Carlos Saavedra, Adriana Robayo, Oscar H. Franco

**Affiliations:** 1 Directorate of Analytical, Economic and Actuarial Studies in Health, Instituto de Evaluación Tecnológica en Salud (IETS) & Economic Models and Quantitative Methods Research Group, Centro de Investigaciones para el Desarrollo, Universidad Nacional de Colombia, Bogotá, D.C. Colombia; 2 Directorate of Analytical, Economic and Actuarial Studies in Health, Instituto de Evaluación Tecnológica en Salud (IETS), Bogotá, Colombia; 3 Faculty of Medicine, Universidad Nacional de Colombia, Bogotá, D.C. Colombia; 4 University Medical Center Utrecht, Utrecht University & Harvard T.H. Chan School of Public Health, Harvard University, Cambridge, USA

## Abstract

**Background:**

The interaction between modelers and policymakers is becoming more common due to the increase in computing speed seen in recent decades. The recent pandemic caused by the SARS-CoV-2 virus was no exception. Thus, this study aims to identify and assess epidemiological mathematical models of SARS-CoV-2 applied to real-world data, including immunization for coronavirus 2019 (COVID-19).

**Methodology:**

PubMed, JSTOR, medRxiv, LILACS, EconLit, and other databases were searched for studies employing epidemiological mathematical models of SARS-CoV-2 applied to real-world data. We summarized the information qualitatively, and each article included was assessed for bias risk using the Joanna Briggs Institute (JBI) and PROBAST checklist tool. The PROSPERO registration number is CRD42022344542.

**Findings:**

In total, 5646 articles were retrieved, of which 411 were included. Most of the information was published in 2021. The countries with the highest number of studies were the United States, Canada, China, and the United Kingdom; no studies were found in low-income countries. The SEIR model (susceptible, exposed, infectious, and recovered) was the most frequently used approach, followed by agent-based modeling. Moreover, the most commonly used software were R, Matlab, and Python, with the most recurring health outcomes being death and recovery. According to the JBI assessment, 61.4% of articles were considered to have a low risk of bias.

**Interpretation:**

The utilization of mathematical models increased following the onset of the SARS-CoV-2 pandemic. Stakeholders have begun to incorporate these analytical tools more extensively into public policy, enabling the construction of various scenarios for public health. This contribution adds value to informed decision-making. Therefore, understanding their advancements, strengths, and limitations is essential.

**Supplementary Information:**

The online version contains supplementary material available at 10.1186/s13643-023-02411-1.

## Introduction

Coronavirus 2019 (COVID-19) is a pathology caused by the SARS-CoV-2 virus. Since 2020, it has resulted in numerous cases and fatalities [1]. Its transmission primarily occurs from person to person, exhibiting high transmissibility and a variable, unpredictable course [2]. Consequently, in March 2020, the World Health Organization (WHO) classified COVID-19 as a pandemic due to its global spread.

Throughout the initial 2 years of the pandemic, cases spread swiftly across the world, albeit asynchronously, yielding heterogeneous effects among different territories. At present, there exist multiple highly effective vaccines against COVID-19, with over 12.5 billion doses administered worldwide [3]. It is important to note that the success of COVID-19 vaccination hinges on factors such as the duration of immunity conferred by vaccines, their efficacy against new SARS-CoV-2 variants, and the implementation protocols in each country [4–6].

The scientific community has been fervently engaged in describing and studying epidemiological phenomena through theoretical and methodological modeling. Hence, this review delves into diverse facets, including the types of epidemiological mathematical model employed, the simulation software used as well as sociodemographic, socioeconomic, clinical, and vaccination-related factors [7]. Epidemiological mathematical modeling furnishes vital information for informed decision-making in public policy. It plays a pivotal role in comprehending and managing infectious diseases, as its tools facilitate the simplification of complex and uncertain scenarios [8]. These quantitative models serve to build scenario planning, evaluate possible scenarios, and analyze them according to their potential risks based on different health outcomes.

Furthermore, the interaction between modelers and policymakers has become more prevalent due to the escalating computing speed in recent decades. This relationship is characterized by complex models that collect the reality of the epidemiological situation in detail and interventions to mitigate it [7]. The recent SARS-CoV-2 pandemic is closely intertwined with this interaction. As a result, the objective of this research is to compile and analyze the predictive models developed for studying diverse health outcomes stemming from COVID-19. This analysis adopts a mathematical epidemiological approach, involving the scrutiny of real-world data. Given the new generation of quantitative health analyses applied to the pandemic with real-world data, we wanted to review what kind of new approaches had been developed that could serve as a basis for future applications of predictive analytics.

## Methodology

A systematic literature search was conducted in accordance with the rapid review format guidelines established in the Cochrane international methods [9]. This approach was complemented with the literature review methodology from software engineering [10, 11]. The PROSPERO registration number for this study is CRD42022344542. A comprehensive and generic search strategy was formulated and subsequently tailored for the diverse sources of information. Language, study type, and date restrictions were not applied. The search strategy encompassed articles available up to April 1, 2022.

The study’s target population comprised individuals who had been vaccinated against COVID-19 within the context of mathematical epidemiological models. The intervention under examination was the COVID-19 vaccine, regardless of brand name. The health outcomes sought were the number of deaths, recovered, hospitalized, infected, and susceptible due to COVID-19. Inclusion criteria therefore referred to the development of mathematical epidemiological models that had used real-world data for their analysis.

The consulted databases included PubMed, JSTOR, medRxiv, LILACS, EconLit, IEEE Transactions on Software Engineering, ACM Transactions on Software Engineering Methodology (TOSEM), Empirical Software Engineering Journal, Journal of Systems and Software, and Information and Software Technology. Google Scholar was utilized for grey literature search. These databases selection was based on the quantitative approach of the literature review and the expertise of the authors.

The search strategy, outlined in Supplement N°. 1, was also complemented with manual searches. The articles included in this review provided information on mathematical epidemiological models of the SARS-CoV-2 virus, incorporating vaccination and relying on real-world data for analysis. Mendeley software facilitated reference deduplication, and Microsoft Excel® software was employed for the screening process. Two independent groups of reviewers (OE, DR, JR, and VB, AR, CS) evaluated each title and abstract in a blinded manner, with conflicts resolved by a third evaluator (OE or LM) when necessary. Full-text screening followed a similar process. Studies included in the full-text review phase were the incorporated into the data extraction phase. Excluded studies are listed in Supplement N°. 2.

Five reviewers (VB, DR, AR, CS, JR) extracted the information, which was independently verified by two reviewers (OE and LM) using a data extraction form designed in Microsoft Excel® (Supplement N°. 3A). Variable definitions are provided in Supplement N°. 3B. The extracted characteristics were summarized descriptively. Results were categorized based on the country’s income, determined by gross national income (GNI) per capita [12], into high-income countries (USD > 12695), upper-middle income (USD 4096–12695), lower-middle income (USD 1046–4095), low-income (USD < 1046), and multi-income studies encompassing countries from different income levels.

Methodological quality was assessed by independent reviewers using two tools: initially, the Joanna Briggs Institute (JBI), classic checklist based on the methodological design [13, 14], and PROBAST, a specialized tool for assessing the risk of bias and applicability assessment in predictive modeling studies in health sciences [15]. Methodological quality assessment was carried out by an expert (LM) and, when needed, agreed upon with a second methodological expert (OE). The methodological tools are shown in Supplement N°. 4.

## Results

### Literature search results

A total of 5646 references were identified from indexed databases, and an additional 197 documents were located in the grey literature. Among these, 2362 (40.4%) were excluded as duplicates, leaving 3481 (59.6%) references for title and abstract screening. From these, 398 (11.43%) references proceeded to full-text evaluation, resulting in the inclusion of 202 (5.8%) articles. Furthermore, by applying a snowball strategy using the *Connected Papers* platform (which uses advanced textual analytics techniques) and reviewing the reference lists of the included articles, an additional 209 references were incorporated, yielding a total of 411 documents included in this review. The information flowchart is depicted in the PRISMA figure (see Supplement N°. 5), and the list of included studies is presented in Supplement N°. 6.

In terms of publication distribution, 16 [16–31], 280 [32–311], and 115 [312–426] articles were published in 2020, 2021, and 2022, respectively. The majority of these articles were concentrated in high-income countries, constituting 69.3% of the publications [16, 17, 20–24, 26, 29–31, 33, 34, 37, 39–43, 46–49, 51, 52, 57–59, 61, 62, 66, 68–70, 72–77, 79, 80, 82–87, 90, 92–94, 98–102, 106–114, 116–124, 126–128, 130–137, 140, 142, 145, 147, 150, 151, 153–161, 164, 165, 167, 169–173, 175, 178–188, 191–195, 197–202, 204, 205, 207, 209, 211, 212, 217, 220, 222, 224, 227, 232–237, 239–252, 254, 255, 257, 258, 260–283, 285, 286, 288–298, 300, 302–304, 307, 310, 314–317, 323, 324, 326–328, 332, 333, 335–338, 340, 342–345, 347–349, 351–353, 355, 357–368, 372–374, 376–378, 381–385, 387–391, 393, 394, 397, 400, 401, 403–405, 407, 410, 412, 415, 416, 418, 420, 421, 423–426]. Only 8% of the articles were for low-middle income countries [36, 38, 44, 45, 54, 56, 89, 104, 105, 115, 129, 138, 143, 144, 146, 163, 206, 218, 223, 256, 287, 299, 306, 312, 318, 319, 331, 339, 369, 380, 398, 399, 414]. The United States (USA) had the most articles related to the topic, with 106 articles (25.8%) [16, 17, 21, 24, 26, 29, 31, 34, 42, 43, 47, 49, 51, 57, 62, 74, 76, 82, 85, 87, 90, 93, 94, 107, 112, 113, 118–122, 131, 133, 135, 137, 142, 147, 151, 153, 155, 159, 172, 179, 180, 182–184, 191–194, 198, 209, 217, 220, 227, 234–236, 241–245, 248, 251, 254, 255, 263, 265–267, 270, 273, 281, 290, 292, 296, 310, 328, 332, 333, 335, 337, 345, 348, 349, 351, 353, 358, 363, 366, 367, 377, 382–384, 388, 390, 391, 400, 401, 407, 410, 415, 424]. Canada followed with 22 documents (5.4%) [22, 33, 41, 66, 77, 111, 126, 145, 154, 156, 181, 197, 200, 202, 205, 240, 269, 276, 300, 317, 357, 364], and China with 20 articles (4.9%) [25, 55, 63, 81, 88, 91, 95, 97, 174, 208, 216, 221, 225, 229, 230, 320, 329, 330, 409, 411]. No information from exclusively low-income countries was found (see Table 1).

**Table 1:** General characteristics of the articles included by income level of the country

**Characteristic**		**High income**		**Upper-middle income**		**Lower-middle income**		**Multi-income**	
		***n***	**%**	***n***	**%**	***n***	**%**	***n***	**%**
**Year**	**2020**	11	2.7	3	0.7	-	-	2	0.5
	**2021**	199	48.4	36	9	23	5.6	22	5.4
	**2022**	75	18.2	16	3.9	10	2.4	14	3.4
**Country**	**USA**	106	25.8	-	-	-	-	-	-
	**Multi-country**	19	4.6	1	0.2	-	-	36	8.8
	**Canada**	22	5.4	-	-	-	-	-	-
	**China**	-	-	20	4.9	-	-	-	-
	**France**	18	4.4	-	-	-	-	-	-
	**United Kingdom**	19	4.6	-	-	-	-	-	-
	**Italy**	15	3.6	-	-	-	-	-	-
	**India**	-	-	-	-	14	3.4	-	-
	**Germany**	10	2.4	-	-	-	-	-	-
	**Others**	75	18.2	3.4	8.3	19	4.6	3	0.7

### Description of epidemiological mathematical models

The primary mathematical model employed was the SEIR (susceptible, exposed, infectious, and recovered) compartmental model, utilized in 47% [193]) of the retrieved articles [17, 19, 24–28, 31–36, 42, 45, 46, 57, 60, 65, 67, 69, 71, 73, 77, 80, 87, 89, 91, 92, 94, 96–100, 103–107, 109, 111–113, 115, 118, 122–128, 130, 131, 133, 135, 138–146, 148–150, 156, 157, 159–165, 167, 168, 174–177, 179, 180, 183, 185, 187, 188, 192, 199, 210, 212, 215, 218–220, 223–227, 233–237, 241, 243, 247, 251–255, 258, 260, 261, 264, 266, 267, 269, 271, 272, 275, 276, 279–281, 283, 284, 288, 289, 291, 294, 297–301, 304, 306, 307, 310, 312–314, 317–319, 326, 327, 331, 341, 344–347, 350, 352–355, 357, 359–361, 369, 371, 381, 382, 384, 390–392, 396, 397, 399, 401, 403, 405, 408, 412–416, 418, 420, 422, 423, 426]. The category “other models” encompasses less commonly models, such as Bayesian networks, Poisson models, and other compartmental models (see Table 2).

**Table 2:** Characterization of the types of mathematical models by income level of the country

**Model type**	**High income**		**Upper-middle income**		**Lower-middle income**		**Multi-income**		**Total**	
	***n***	**%**	***n***	**%**	***n***	**%**	***n***	**%**	***n***	**%**
**SEIR model**	128	31.1	28	6.8	22	5.4	15	3.6	193	47.0
**Agent-based model**	55	13.4	5	1.2	1	0.2	3	0.7	64	15.6
**SIR model**	37	9.0	8	1.9	3	0.7	5	1.2	53	12.9
**Compartmental model**	10	2.4	3	0.7	2	0.5	1	0.2	16	3.9
**Other models**	55	13.4	11	2.7	5	1.2	14	3.4	85	20.7
**Total**	285	69.3	55.0	13.4	33.0	8.0	38.0	9.2	411	100

Regarding the operational characteristics and accessibility to the databases and models employed in the included studies, it was noted that only 16.3% of the references did not engage in mathematical development of the proposed model [18, 20, 30, 40, 43, 52, 59, 62, 81, 83, 90, 120, 121, 131, 137, 142, 151, 154, 157, 167, 170, 172, 173, 177, 178, 188, 189, 192, 194, 202, 205–208, 211, 215, 217, 222, 242, 245, 247–250, 254, 273, 277, 285, 295, 301, 305, 324, 332, 334, 339, 340, 351, 358, 365, 372, 385, 387, 390, 393, 404, 417, 426]. The main software utilized was R, accounting for 19.7% of cases [27, 29, 32, 33, 40, 41, 53, 60, 62, 67, 76, 82, 91, 92, 94–96, 106, 109, 111, 114, 117, 132, 138, 157, 164–166, 179, 180, 211, 218, 219, 222, 238, 244–246, 248, 253, 254, 261, 264, 275, 277, 279, 283, 288, 292, 295, 296, 311, 313, 319, 323, 325, 326, 328, 330, 332, 334, 336, 346, 347, 349, 355, 361, 363, 373, 378, 380, 382–384, 388, 389, 392, 394, 397, 407, 415]. A majority of the references, 59.9%, did not present the programming code with open access. Merely 3.6% of these references developed a dashboard [60, 74, 84, 114, 180, 250, 256, 260, 280, 295, 331, 338, 378, 380, 382] (see Table 3).

**Table 3:** Operational characteristics of the mathematical models

**Characteristic**		***n***	**%**
**The model is developed**	**Yes**	344	83.7
	**No**	67	16.3
**Software**	***R***	81	19.7
	**MATLAB**	71	17.3
	**Python**	57	13.9
	**Microsoft Excel**	13	3.2
	**Julia**	11	2.7
	**C++**	11	2.7
	**Others**	9	2.2
	**No information**	158	38.4
**Available code**	**Yes**	165	40.1
	**No**	246	59.9
**Dashboard available**	**Yes**	15	3.6
	**No**	396	96.4

### Description of sociodemographic aspects

When analyzing the sociodemographic characteristics reported in the included articles, it was observed that 252 articles (61.3%) incorporated age in their models [16–18, 21, 23, 25, 26, 29, 31, 33, 34, 36, 39–42, 44, 47, 48, 50, 52–55, 58–60, 64–66, 68–70, 72, 74, 77, 79, 80, 82–88, 90, 92, 95, 96, 99, 102–106, 108, 109, 111–114, 116–126, 129, 133, 134, 137, 138, 140–142, 144, 147–151, 153–157, 160–162, 164, 165, 167–170, 174, 175, 178, 179, 184, 187, 188, 192, 196, 199, 201–206, 208, 210, 211, 213, 215–217, 219, 221, 222, 227, 229, 234–237, 241, 242, 245–249, 253–255, 258–260, 262, 263, 266, 269, 271–277, 279, 281–285, 288, 290, 291, 293–295, 297, 298, 300–304, 308–311, 313, 314, 316, 319, 320, 323–343, 345, 347–351, 354, 355, 357–359, 361, 365–367, 370, 371, 373–378, 380, 382, 386–391, 393, 394, 397, 402, 403, 405–407, 410, 416–418, 423–426]. Among these, 13 (3.2%) were documented within lower-middle-income countries [36, 44, 54, 104, 105, 129, 138, 144, 206, 319, 331, 339, 380].

Sex-related data were incorporated into mathematical modeling in 23 articles (5.6%) [59, 68, 74, 84, 95, 165, 170, 174, 203, 227, 235, 260, 263, 285, 301, 305, 334, 336, 367, 377, 388, 397, 426]. Additionally, 6 articles (1.5%) included information related to ethnicity [59, 137, 155, 235, 367, 388], and 15 articles (3.6%) included vulnerable groups [63, 64, 71, 81, 177, 291, 331, 374, 378, 380, 382, 404, 408, 409, 412] (see Table 4).

**Table 4:** Sociodemographic characteristics by income level of the country

**Sociodemographic characteristic**		**High income**		**Upper-middle income**		**Lower-middle income**		**Multi-income**		**Total**	
		***n***	**%**	***n***	**%**	***n***	**%**	***n***	**%**	***n***	**%**
**Age**	**No**	98	23.8	24	5.8	20	4.9	17	4.1	159	38.7
	**Yes**	187	45.5	31	7.5	13	3.2	21	5.1	252	61.3
**Sex**	**No**	268	65.2	50	12.2	33	8.0	37	9.0	388	94.4
	**Yes**	17	4.1	5	1.2	-	-	1	0.2	23	5.6
**Ethnicity**	**No**	279	67.9	55	13.4	33	8.0	38	9.2	405	98.5
	**Yes**	6	1.5	-	-	-	-	-	-	6	1.5
**Occupation**	**No**	246	59.9	46	11.2	31	7.5	35	8.5	358	87.1
	**Yes**	39	9.5	9	2.2	2	0.5	3	0.7	53	12.9
**Migrants**	**No**	279	67.9	49	11.9	31	7.5	37	9.0	396	96.4
	**Yes**	6	1.5	6	1.5	2	0.5	1	0.2	15	3.6

### Description of clinical and vaccine aspects

#### Comorbidities and reinfection

Among the included articles, 40 (9.7%) incorporated information related to comorbidities [42, 59, 64, 74, 77, 84, 85, 91, 99, 104, 108, 119–121, 123, 129, 137, 138, 144, 146, 155, 173, 196, 202, 203, 205, 206, 241, 253, 269, 282, 283, 300, 349, 351, 354, 366, 372, 377, 388]. Among these, 28 (6.8%) were within high-income countries. However, none of these studies provided a specific description of the identified comorbidities.

On the other hand, 117 (28.5%) references considered the reinfection variable [17, 22, 28, 32, 36–38, 44, 46, 59–61, 63, 70, 72, 77, 79, 92, 94, 103, 105, 108, 122, 125, 128, 129, 138, 140, 145, 146, 149, 153, 156, 157, 163–165, 168, 172, 175, 176, 178, 185, 190, 192, 198, 201, 217–219, 223, 226, 230, 231, 233, 241, 243, 254, 258, 264, 265, 267, 269, 276, 277, 281, 283, 285, 291, 293, 296, 299, 300, 304, 305, 308, 313–315, 318, 319, 328, 333–335, 341, 343, 344, 346, 350, 354, 358, 361, 362, 364, 373, 374, 377, 380–382, 389, 390, 396, 399, 402–404, 406, 408, 409, 411, 413, 414, 420, 421, 426]. Out of these, 90 articles (21.9%) were elaborated for high and upper-high income countries [17, 22, 28, 32, 37, 46, 59, 61, 63, 70, 72, 77, 79, 92, 94, 108, 122, 125, 128, 140, 145, 149, 153, 156, 157, 164, 165, 168, 172, 175, 176, 178, 185, 192, 198, 201, 217, 230, 233, 241, 243, 254, 258, 264, 265, 267, 269, 276, 277, 281, 283, 285, 291, 293, 296, 300, 304, 305, 308, 314, 315, 328, 333–335, 343, 344, 346, 354, 358, 361, 362, 364, 373, 374, 377, 381, 382, 389, 390, 403, 404, 406, 408, 409, 411, 413, 420, 421, 426].

#### Types of vaccines, number of vaccines, and heterologous vaccination

Although most articles indicate the use of vaccines in the modeling, many did not specify a particular vaccine. Specifically, 319 articles (77.6%) considered the use of only one type of vaccine [16–32, 34, 35, 37–41, 43–50, 53–58, 60–67, 69–71, 73–75, 79–86, 88–100, 102–105, 107, 111, 112, 115, 118, 121, 122, 124–126, 128–133, 135, 137–141, 143–151, 154, 156, 158–160, 163, 165–177, 180–195, 197–202, 206, 208, 209, 212, 213, 215–221, 223–234, 236–238, 240–247, 249–253, 255, 256, 258, 259, 261, 263–266, 269, 274, 275, 277, 278, 280–282, 284, 286–299, 301–303, 306–310, 314–317, 321–323, 327, 329–332, 334, 337–343, 345–348, 350–353, 356–358, 362, 363, 366, 368–371, 373–382, 384–402, 404–426]. Only two articles (0.5%) did not refer to this topic [106, 110].

Regarding the number of reported doses, 159 references (38.7%) included data from the first and second doses of the vaccines in the models [29, 39, 41, 43, 49, 53–55, 59, 60, 62, 63, 66, 68–70, 73, 78, 79, 82, 85, 90, 97, 100, 101, 103, 105, 109, 111, 114, 117, 121, 123–126, 128, 134, 136, 138, 140, 142, 144, 152, 153, 155, 156, 158–165, 167, 173, 178, 182, 184, 186–188, 195, 199, 202–206, 216, 217, 221, 224, 228–230, 233, 235–237, 239, 240, 242, 249, 260, 263, 265, 271, 273, 276, 278, 279, 287, 290, 292, 297, 298, 302–305, 307, 308, 310, 312, 313, 315, 319, 320, 323, 324, 329, 330, 332, 334, 335, 337, 340, 342, 348, 349, 351–353, 356, 358, 359, 361–363, 366, 367, 370, 372–374, 376–378, 381, 384, 387–389, 392–394, 397, 405, 407, 410, 415, 417, 418, 421, 423–425]. Ten (2.4%) articles reported data concerning booster doses [91, 96, 115, 122, 127, 201, 322, 333, 341, 422].

Heterogeneous vaccine was indicated for high-income countries in two (0.5%) articles [106, 382]. In 95 articles (23.1%), the difference in days between administered doses were considered; out of these, 22 articles (23.2%) considered a 21-day interval between doses [55, 60, 69, 100, 122, 133, 134, 140, 187, 216, 217, 221, 302, 303, 329, 330, 335, 342, 383, 384, 424, 425], while 22 (23.2%) considered a period of 21- to 28-day interval [42, 90, 116, 119–121, 136, 142, 151, 155, 184, 204, 205, 258, 260, 273, 324, 328, 351, 354, 356, 366]. Additionally, 11 (11.6%) articles considered a period equal to or greater than three months [203, 211, 222, 269, 300, 314, 317, 333, 334, 350, 361]. Four articles identified had dose intervals greater than 150 days, all in high-income countries [211, 222, 314, 333], with one having a 240-day interval for the booster dose [333].

Regarding trademarked vaccines, 12 articles reported a 28-day difference for the Moderna vaccine [42, 116, 119, 120, 142, 155, 184, 205, 258, 260, 351, 366]. For the Pfizer-BioNTech, 10 references indicated a 21-day difference between the first and second dose [69, 100, 133, 134, 217, 302, 303, 335, 342, 383], while one reference indicated a 28-day difference [173]. Different intervals were identified for the AstraZeneca vaccine: 70 days [139], 84 days [99], and 28, 56, 84, 112, and 140 days [350].

#### Vaccine effectiveness and difference between effectiveness

Most studies were conducted in high-income countries. Within the total references, effectiveness rates of 50%, 60%, 70%, 80%, and 100% were presented in 63, 40, 47, 52, and 33 articles, respectively. Notably, articles focused on low-middle-income countries predominantly employed an effectiveness rates of 80%. Out of the 33 articles considering an effectiveness rate of 100%, 23 (5.6%) were conducted in high-income countries.

The majority of articles did not specify the exact outcome against which the effectiveness was measured. Among those reporting a 100% effectiveness rate, two indicated effectiveness in preventing death [99, 282]. In 14 articles (3.4%), the effectiveness rates were reported to be less than 10% [23, 28, 78, 106, 112, 179, 229, 311, 319, 326, 364, 379, 391, 400]. Notably, two of these articles mentioned an effectiveness of 0%. The first dealt with the gamma variant using the CoronaVac vaccine [311], while the second addressed subsequent infection [379].

Regarding the different variants, 155 articles (37.7%) described the specific strain included in their models. Among these, the delta variant was the most frequently modeled, with a total of 41 references (10%) [43, 47, 91, 96, 105, 114, 119, 122, 124, 128, 149, 153, 218, 252, 258, 262, 278, 286, 297, 314, 318, 321, 326, 328, 329, 332, 333, 338, 340, 343, 350, 352, 357, 367, 380, 387, 401, 416, 419, 421, 425]. The alpha variant followed with 18 articles (4.4%) [60, 93, 101, 113, 156, 157, 180, 195, 199, 247–249, 296, 298, 303, 331, 393, 397] (see Table 5).

**Table 5:** Variants identified in the articles by income level of the country

**Variants**	**High income**		**Upper-middle income**		**Lower-middle income**		**Multi-income**		**Total**	
	***n***	**%**	***n***	**%**	***n***	**%**	***n***	**%**	***n***	**%**
**Combination of variants**	54	13.1	7	1.7	3	0.7	9	2.2	73	17.8
**Delta**	30	7.3	4	1.0	4	1.0	3	0.7	41	10.0
**Alpha**	16	3.9	-	-	1	0.2	1	0.2	18	4.4
**No specification**	8	1.9	3	0.7	1	0.2	1	0.2	13	3.2
**Omicron**	2	0.5	2	0.5	-	-	1	0.2	5	1.2
**Others**	4	1.0	-	-	-	-	1	0.2	5	1.2
**Gamma**	-	-	1	0.2	-	-	-	-	1	0.2
**No information**	171	41.6	38	9.2	24	5.8	22	5.4	255	62

The articles encompassed a range of evaluated outcomes, including deceased, recovered, hospitalized (e.g., in an intensive care unit), infected, and susceptible cases. Among these, the main outcome studied, death, was reported in 313 articles (76.2%) [16–22, 25, 26, 28, 29, 31–45, 47–50, 52–62, 64–66, 68–70, 72–74, 76–79, 82–91, 93, 97–100, 102–110, 112–127, 129–142, 144–148, 150, 151, 153–156, 158, 159, 163, 164, 166–170, 172, 174–176, 178–181, 183, 184, 187–189, 193–196, 198, 202–205, 207, 210–213, 215, 217–222, 224–231, 235, 237, 240–243, 245–248, 250, 252–256, 258–261, 263–267, 269–288, 290–294, 300, 301, 305, 306, 308, 310–312, 314, 315, 317–326, 328–337, 339–342, 344–346, 348–355, 358–360, 362–368, 370, 371, 374, 375, 377, 384, 385, 387–390, 392–395, 397, 398, 400, 401, 403, 404, 406, 408–421, 423, 424]. Notably, the majority of these articles, 218 (53%), were conducted in high-income countries [16, 17, 20–22, 26, 29, 31, 33, 34, 37, 39–43, 47–49, 52, 57–59, 61, 62, 66, 68–70, 72–74, 76, 77, 79, 82–87, 90, 93, 98–100, 102, 106–110, 112–114, 116–124, 126, 127, 130–137, 140, 142, 145, 147, 150, 151, 153–156, 158, 159, 164, 167, 169, 170, 172, 175, 178–181, 183, 184, 187, 188, 193–195, 198, 202, 204, 205, 207, 211, 212, 217, 220, 222, 224, 227, 235, 237, 240–243, 245–248, 250, 252, 254, 255, 258, 260, 261, 263–267, 269–283, 285, 286, 288, 290–294, 300, 310, 314, 315, 317, 323, 324, 326, 328, 332, 333, 335–337, 340, 342, 344, 345, 348, 349, 351–353, 355, 358–360, 362–368, 374, 377, 384, 385, 387–390, 393, 394, 397, 400, 401, 403, 404, 410, 412, 415, 416, 418, 420, 421, 423, 424].

In terms of the health outcomes studied, 270 references (65.7%) included the number of recovered cases. Additionally, 189 articles (46%) reported the number of hospitalizations, while 45 articles (10.9%) incorporated the count of infected cases [30, 60, 69, 73–75, 81, 95, 101, 148, 152, 159, 164, 168, 172, 187, 189, 198, 200, 207–209, 215, 224, 226, 227, 232, 239, 251, 257, 260, 279, 284, 351, 356, 372, 373, 377, 379, 382, 386, 390, 405, 410, 415] (see Table 6).

**Table 6:** Outcomes identified in the included articles by income level of the country

**Outcome**	**High income**		**Upper-middle income**		**Lower-middle income**		**Multi-income**		**Total**	
	***n***	**%**	***n***	**%**	***n***	**%**	***n***	**%**	***n***	**%**
**Deaths**	218	53	41	10.0	26	6.3	28	6.8	313	76.2
**Recovered**	182	44.3	40	9.7	25	6.1	23	5.6	270	65.7
**Hospitalized**	140	34.1	27	6.6	14	3.4	8	1.9	189	46
**Infected**	31	7.5	6	1.5	-	-	8	1.9	45	10.9
**Susceptible**	2	0.5	-	-	-	-	-	-	2	0.5

### Methodological quality of the studies

Methodological evaluation was conducted on 402 articles (97.8%) [16–83, 85–109, 111–121, 123, 126, 128–195, 197–301, 303–400, 402–426]. However, the checklist was not applied to nine articles (2.2%) due to their nature as letters to the editor or comments [83, 87, 106, 113, 115, 119, 188, 204, 351].

Upon using the JBI tool, it was determined that 135 articles (33.6%) were at high risk due to a lack of description regarding the studied population [16–18, 20, 22–24, 30–33, 40, 41, 43–46, 49, 57, 59, 67, 78, 82, 88, 89, 94, 97, 98, 101, 102, 107, 108, 111, 132, 137, 138, 151–154, 162, 163, 165, 166, 172, 175, 176, 178–180, 182, 189, 190, 194, 202, 203, 205, 207, 208, 211, 212, 214, 215, 220, 222, 224, 228, 231, 232, 234–236, 239, 241, 246, 248, 250, 252, 253, 255, 257, 259, 261–264, 267–269, 271–275, 279, 281, 283, 286, 289–292, 295–297, 300, 301, 309, 315, 316, 318, 319, 323, 332, 334, 345, 348, 351, 353, 360, 364, 365, 369, 371, 374, 377, 379, 380, 382, 384, 387, 404, 414, 418]. Conversely, 247 articles (61.4%) were deemed low risk as they met the methodological domains outlined in the quality tool. Additionally, 20 articles (5.0%) [27, 35, 109, 125, 136, 155, 160, 169, 173, 198, 247, 261, 311, 324, 344, 368, 389, 403, 405, 423] were categorized as unclear due to insufficient methodological information.

In applying the PROBAST tool, it was concluded that 65.3% of the articles presented a low risk in the bias risk domain. On the other hand, in the applicability domain, 89.8% reported a low risk. Articles classified as high risk in the bias risk domain often lacked information about the population studied. In contrast, those classified as unclear in the applicability domain lacked sufficient methodological details for evaluation. Many of these articles also lacked information on the methods used to control for third variables or analyze predictive outcomes (Supplement N°. 7).

## Discussion

In this review, most models were developed in countries classified as high income, and the SEIR model was more frequently used. This research identified a considerable increase in these articles in the years 2021 (1650%) and 2022 (618.8%) compared to the initial year of the pandemic, a fact that reflects the unavailability of vaccines until the end of 2020.

High-income countries have used these strategies extensively to advise on health decision-making. In this sense, it is striking that only Singapore, the United Arab Emirates, South Korea, Japan, Italy, and Canada are among the countries that exceeded 80% of the population vaccinated [427]. The USA, where the largest number of identified models were developed, has one of the highest crude mortality rates from the disease, even after deploying vaccinations, suggesting a disconnect between the developers of the models and their actual implementation and articulation with public health.

This systematic review provided insight into the main features that have been incorporated into mathematical epidemiological models worldwide, allowing for a clear picture of the different advantages and disadvantages of the multiple analytical approaches to describe COVID-19 behavior and vaccine effectiveness. For governmental decision-making and depending on the public policy question to be answered, this research is a comprehensive compilation that provides sufficient information on the different options for scientific teams to choose from. Depending on the health outcomes to be predicted, the socio-demographic variables to be included, among other aspects, there is an amalgam of algorithms to carry out the mathematical epidemiological modeling.

One point to keep in mind during mathematical modeling in COVID-19 is that the inputs used should ideally come from reliable sources close to the context of the community in which the model is being applied. Hence the importance of an effective surveillance system in capturing cases and outcomes of the disease. Many high-income countries opted to use new technologies to analyze patterns of cases, to strengthen their surveillance system [428].

Among the main advantages of mathematical modeling in infectious diseases and in the context of public health are the multiple representations of scenarios, which can predict essential health outcomes at lower costs [429]. However, the realization of these models often implies the need to financially support multidisciplinary groups carrying out such processes. Unfortunately, not all institutions and governments can afford this. In our systematic review, this gap becomes visible in the scientific production of high-income countries compared to lower-middle and low-income countries.

One limitation of this study is that the predictive capacity of the analytical models was not reviewed, given the impossibility of measuring this characteristic homogeneously. In addition, unfortunately, most of the articles did not present their open programming codes, making it impossible to replicate and review their computational developments. Another limitation of this research was that it was not possible to take into account predictive models used (by some governments) that were useful for decision-making, because some governments considered it a national security issue and never shared these advances with society.

Although the most widely used model (SEIR, in its classic version) has limitations [430], such as the absence of a defined case model, discrepancies in the information available at the population level compared to the individual [431], a lack of incorporation of individual behavior and social influence, and the lack of flexibility to incorporate new evidence [432], it is a very useful and practical model for different purposes in environments with high uncertainty. Therefore, it is necessary to dynamically adjust the models to the reality of the evolution of the pandemic.

Based on our exhaustive compilation, we consider it good international practice for future analytical modeling of infectious diseases to (i) use as many parameters as possible from real-world evidence from the area being studied; (ii) be clear about the mathematical structure of the model being applied (annexes can provide such details of the document); (iii) show all the parameters with which the basis of the exercise is modeled; and (iv) where possible leave the programming code open or available on request to the reader.

Likewise, one of the significant challenges at present is to know the impact of vaccination (effectiveness) in the long term, whether hybrid protection (natural and vaccine immunity) is better, and what vaccination schemes (one, two, three, or four doses) or what type of heterologous schemes produce a greater response. Mathematical modeling is of great help in epidemiology and public health; however, increasing the number of parameters can make the analysis, calibration, implementation, and interpretation of results difficult.

Epidemiological mathematical models are a tool that allows us to predict the behavior of the virus, with certain limitations, providing information for decision-making on public health control measures. The development of more and better epidemiological mathematical models in public health serves as a tool to mitigate negative scenarios and to aid policymakers in navigating through uncertain contexts. It will be essential to standardize the methods used for epidemiological modeling to guarantee high-quality results. Similarly, it will be vital to ensure human and financial resources so that these models are made in the best possible way, with accurate data and in real time. Thus, health policies must be based on evidence to generate the best results in the population.

## Supplementary Information

Supplementary Material N°. 1. Search strategies.

Supplementary Material N°. 2. Excluded studies and reasons for exclusion.

Supplementary Material N°. 3. Variables’ operational definition.

Supplementary Material N°. 4. Checklist - Methodological quality.

Supplementary Material N°. 5. PRISMA flow chart.

Supplementary Material N°. 6. List of included studies.

Supplementary Material Supplementary material N°. 7. Quality assessment (Johanna Briggs Institute Checklist, PROBAST Checklist).

## Data Availability

All data generated or analyzed during this study are included in this published article (and its supplementary information files).
