# Supplementary material for: Predictive models for health outcomes due to SARS-CoV-2, including the effect of vaccination: a systematic review
Source: Syst Rev. 2024 Jan 16;13:30. doi: 10.1186/s13643-023-02411-1 (PMC10790449; doi:10.1186/s13643-023-02411-1)
Supplement: Supplementary file 1 — Supplementary Material N°. 1. Search strategies. [file 13643_2023_2411_MOESM1_ESM.docx]

# Supplementary material N°. 1. Search strategies

## PubMed

| Line | Search Terms |
| --- | --- |
| **1** | covid-19[MeSH Terms] |
| **2** | SARS-CoV-2[Mesh] |
| **3** | (#1) OR (#2) |
| **4** | (((covid-19[MeSH Terms]) OR (SARS-CoV-2[Mesh])) OR ('2019 novel coronavirus disease' or '2019 novel coronavirus disease' or '2019 novel coronavirus epidemic' or '2019 novel coronavirus infection' or '2019-ncov disease' or '2019-ncov infection' or 'covid' or 'covid 19' or 'covid 19 induced pneumonia' or 'covid 2019' or 'covid-10' or 'covid-19' or 'covid-19 induced pneumonia' or 'covid-19 pneumonia' or 'covid19' or 'sars coronavirus 2 infection' or 'sars coronavirus 2 pneumonia' or 'sars-cov-2 disease' or 'sars-cov-2 infection' or 'sars-cov-2 pneumonia' or 'sars-cov2 disease' or 'sars-cov2 infection' or 'sarscov2 disease' or 'sarscov2 infection' or 'wuhan coronavirus disease' or 'wuhan coronavirus infection' or 'coronavirus disease 2' or 'coronavirus disease 2010' or 'coronavirus disease 2019' or 'coronavirus disease 2019 pneumonia' or 'coronavirus disease-19' or 'coronavirus infection 2019' or 'ncov 2019 disease' or 'ncov 2019 infection' or 'new coronavirus pneumonia' or 'novel coronavirus 2019 disease' or 'novel coronavirus 2019 infection' or 'novel coronavirus disease 2019' or 'novel coronavirus infected pneumonia' or 'novel coronavirus infection 2019' or 'novel coronavirus pneumonia' or 'severe acute respiratory syndrome 2' or 'severe acute respiratory syndrome 2 pneumonia' or 'severe acute respiratory syndrome cov-2 infection' or 'severe acute respiratory syndrome coronavirus 2 infection' or 'severe acute respiratory syndrome coronavirus 2019 infection' |
| **5** | Vaccin* |
| **6** | ((Statistical OR Probabilistic OR Polynomial OR "Two-Parameter" OR Binomial OR Epidemiological OR "Communicable Disease Models" OR SIR OR "Susceptible Infected Recovered" OR SIS OR Compartmental OR Mathematic* OR Linear OR Linear Regression OR Log Linear OR Log-Linear OR Logistic OR Logistic Regression* OR Logit Model* OR Cox OR Cox Proportional Hazards OR Hazard OR Proportional Hazard OR Polynomial OR Computational Neural Network* OR Computer Neural Network* OR Connectionist Model* OR Perceptron OR Econometric OR Chaos Theor* OR Non linear Dynamics OR Non linear OR Nonlinear OR Non-linear) AND model* ) |
| **7** | (((#3) AND (#4)) AND (#5)) AND (#6) |

## JSTOR

Search string: covid 19 AND vaccin* AND epidemiological model

Results: 154 documents

## EconLit

Search string: covid 19 AND vaccin* AND model

Results: 17 documents

## MedRxiv

Search string: "(covid 19) AND vaccin* AND (epidemiological model)"

First 200 documents

## LiLacs

Search string: ("COVID 19") AND (vaccin*) AND ((model* AND (statistical OR epidemiologi*)))

Results: 1.341 documents

## Google Scholar

Search string: (covid 19) AND vaccin* AND (epidemiological model)

Results: 197 documents

## IEEE Transactions on Software Engineering

Search string: (covid) AND (vaccin*) AND (model*)

Results: 290 documents

## ACM Transactions on Software Engineering Methodology (TOSEM)

Search string: [All: covid] AND [All: model*] AND [All: vaccin*]

Results: 484 documents

## Empirical Software Engineering Journal

Search string: (covid) AND (vaccin*) AND (model*)

Results: one document

## Journal of Systems and Software

Search string: (covid) AND (vaccin) AND (model)

Results: no documents

## Information and Software Technology

Search string: (covid) AND (vaccin) AND (model)

Results: no documents
