# Supplementary material for: Predictive models for health outcomes due to SARS-CoV-2, including the effect of vaccination: a systematic review
Source: Syst Rev. 2024 Jan 16;13:30. doi: 10.1186/s13643-023-02411-1 (PMC10790449; doi:10.1186/s13643-023-02411-1)
Supplement: Supplementary file 2 — Supplementary Material N°. 2. Excluded studies and reasons for exclusion. [file 13643_2023_2411_MOESM2_ESM.docx]

# Supplementary material N°. 2. Excluded studies and reasons for exclusion

| **Author** | **Title** | **Reason for exclusion** |
| --- | --- | --- |
| Abernethy et al. | Optimal COVID-19 lockdown strategies in an age-structured SEIR model of Northern Ireland | The effectiveness is not modelled |
| Afzal et al. | Merits and limitations of mathematical modeling and computational simulations in mitigation of COVID-19 pandemic: a comprehensive review | The effectiveness is not modelled |
| Ahmad et al. | Mathematical analysis for the effect of voluntary vaccination on the propagation of Corona virus pandemic | The effectiveness is not modelled |
| Akman et al. | The hard lessons and shifting modeling trends of COVID-19 dynamics: multiresolution modeling approach | There is no real-world application |
| Al-Shaery et al. | Evaluating COVID-19 control measures in mass gathering events with vaccine inequalities | There is no real-world application |
| Alanazi et al. | Measuring and preventing COVID-19 using the SIR model and machine learning in smart health care | The effectiveness is not modelled |
| Albani et al. | COVID-19 underreporting and its impact on vaccination strategies | The effectiveness is not modelled |
| Ali et al. | Countering the potential re-emergence of a deadly infectious disease - Information warfare, identifying strategic threats, launching countermeasures | The effectiveness is not modelled |
| Alkhammash et al. | Novel prediction model for COVID-19 in Saudi Arabia based on an LSTM algorithm | The effectiveness is not modelled |
| Alrasheed et al. | COVID-19 spread in Saudi Arabia: modeling, simulation and analysis | The effectiveness is not modelled |
| Alvarez et al. | Modeling vaccination strategies in an Excel spreadsheet: increasing the rate of vaccination is more effective than increasing the vaccination coverage for containing COVID-19 | The effectiveness is not modelled |
| Atkeson et al. | Behavior and the dynamics of epidemics | The effectiveness is not modelled |
| Awad et al. | Implementation of a vaccination program based on epidemic geospatial attributes: COVID-19 pandemic in Ohio as a case study and proof of concept | The effectiveness is not modelled |
| Badfar et al. | Design a robust sliding mode controller based on the state and parameter estimation for the nonlinear epidemiological model of COVID-19 | The effectiveness is not modelled |
| Balisacan et al. | Two new compartmental epidemiological models and their equilibria | There is no real-world application |
| Baniasad et al. | COVID-19 in Asia: transmission factors, re-opening policies, and vaccination simulation | The effectiveness is not modelled |
| Barlow et al. | Optimal shutdown strategies for COVID-19 with economic and mortality costs: British Columbia as a case study | The effectiveness is not modelled |
| Barreiro et al. | Strategies for COVID-19 vaccination under a shortage scenario: a geo-stochastic modelling approach | The effectiveness is not modelled |
| Batista et al. | Minimizing disease spread on a quarantined cruise ship: a model of COVID-19 with asymptomatic infections | The effectiveness is not modelled |
| Beams et al. | Will SARS-CoV-2 become just another seasonal coronavirus? | The effectiveness is not modelled |
| Beira et al. | A differential equations model-fitting analysis of COVID-19 epidemiological data to explain multi-wave dynamics | The effectiveness is not modelled |
| Bekiros et al. | SBDiEM: a new mathematical model of infectious disease dynamics | The effectiveness is not modelled |
| Berger et al. | An SEIR infectious disease model with testing and conditional quarantine | The effectiveness is not modelled |
| Betti et al. | COVID-19 vaccination and healthcare demand | The effectiveness is not modelled |
| Betti et al. | Integrated vaccination and non-pharmaceutical interventions based strategies in Ontario, Canada, as a case study: a mathematical modelling study | The effectiveness is not modelled |
| Bicher et al. | Model based estimation of the SARS-CoV-2 immunization level in Austria and consequences for herd immunity effects | The effectiveness is not modelled |
| Bienstock et al. | A flexible COVID-19 model to assess mitigation, reopening, virus mutation and other changes | The effectiveness is not modelled |
| Bokharaie et al. | A study on the effects of containment policies and vaccination on the spread of SARS-CoV-2 | The effectiveness is not modelled |
| Bongiorno et al. | A multi-layer network model to assess school opening policies during a vaccination campaign: a case study on COVID-19 in France | The effectiveness is not modelled |
| Bonsall et al. | Optimal time to return to normality: parallel use of COVID-19 vaccines and circuit breakers | The effectiveness is not modelled |
| Bordehore et al. | Understanding COVID-19 spreading through simulation modeling and scenarios comparison: preliminary results | The effectiveness is not modelled |
| Borovsky et al. | Model of epidemic kinetics with a source on the example of Moscow | The effectiveness is not modelled |
| Böttcher et al. | Decisive conditions for strategic vaccination against SARS-CoV-2 | The effectiveness is not modelled |
| Braun et al. | COVID-19: predictions for SARS-CoV-2 vaccination on the course of the pandemic and critical occupancy of intensive-care facilities in Germany | The effectiveness is not modelled |
| Brennan et al. | Estimating the effect of timetabling decisions on the spread of SARS-CoV-2 in medium-to-large engineering schools in Canada: an agent-based modelling study | There is no real-world application |
| Brook et al. | Optimizing COVID-19 control with asymptomatic surveillance testing in a university environment | The effectiveness is not modelled |
| Bushman et al. | Population impact of SARS-CoV-2 variants with enhanced transmissibility and/or partial immune escape | There is no real-world application |
| Butler et al. | The effect of population flow on epidemic spread: analysis and control | The effectiveness is not modelled |
| Caldwell et al. | Vaccines and variants: modelling insights into emerging issues in COVID-19 epidemiology | The effectiveness is not modelled |
| Cameron et al. | Preventing global catastrophic biological risks: lessons and recommendations from a tabletop exercise held at the 2020 Munich security conference | There is no real-world application |
| Capobianco et al. | Agent-Based Markov modeling for improved COVID-19 mitigation policies | The effectiveness is not modelled |
| Cartocci et al. | A compartment modeling approach to reconstruct and analyze gender and age-grouped COVID-19 Italian data for decision-making strategies | The effectiveness is not modelled |
| Castonguay et al. | Spatial allocation of scarce COVID-19 vaccines | There is no real-world application |
| Català et al. | The impact of prioritization and dosing intervals on the effects of COVID-19 vaccination in Europe: an agent-based cohort model | There is no real-world application |
| Chalkiadakis et al. | Infection rate models for COVID-19: model risk and public health news sentiment exposure adjustments | The effectiveness is not modelled |
| Chapman et al. | Unexposed populations and potential COVID-19 hospitalisations and deaths in European countries as per data up to 21 November 2021 | The effectiveness is not modelled |
| Chattopadhyay et al. | Infection kinetics of COVID-19 and containment strategy | The effectiveness is not modelled |
| Chaturvedi et al. | Predictive analysis of COVID-19 eradication with vaccination in India, Brazil, and U.S.A | The effectiveness is not modelled |
| Chen et al. | Control of COVID-19 pandemic: vaccination strategies simulation under probabilistic Node-Level model | There is no real-world application |
| Cherednik et al. | Modeling the waves of COVID-19 | The effectiveness is not modelled |
| Choi et al. | Optimal strategies for vaccination and social distancing in a game-theoretic epidemiologic model | There is no real-world application |
| Chrin et al. | Analysis and prediction of COVID-19 data using machine learning models | The effectiveness is not modelled |
| Chu et al. | Dynamics analysis and countermeasures of COVID-19 epidemic | There is no real-world application |
| Colomer et al. | Modeling of vaccination and contact tracing as tools to control the COVID-19 outbreak in Spain | The effectiveness is not modelled |
| Comito et al. | Predicting COVID-19 with AI techniques: current research and future directions | There is no real-world application |
| Cot et al. | Impact of US vaccination strategy on COVID-19 wave dynamics | The effectiveness is not modelled |
| Daghriri et al. | Evolution of select epidemiological modeling and the rise of population sentiment analysis: a literature review and COVID-19 sentiment illustration | The effectiveness is not modelled |
| Damone et al. | Decision-making algorithm and predictive model to assess the impact of infectious disease epidemics on the healthcare system: the COVID-19 case study in Italy | The effectiveness is not modelled |
| Dash et al. | Effects of vaccination decisions and peer influence on epidemic dynamics: a network perspective | There is no real-world application |
| Dashtbali et al. | A compartmental model that predicts the effect of social distancing and vaccination on controlling COVID-19 | The effectiveness is not modelled |
| Davids et al. | Social learning in a network model of COVID-19 | The effectiveness is not modelled |
| Debrabant et al. | The cost-effectiveness of a COVID-19 vaccine in a Danish context | The effectiveness is not modelled |
| Deng et al. | Joint impacts of media, vaccination and treatment on an epidemic Filippov model with application to COVID-19 | The effectiveness is not modelled |
| Djaafara et al. | Using syndromic measures of mortality to capture the dynamics of COVID-19 in Java, Indonesia, in the context of vaccination rollout | The effectiveness is not modelled |
| Du et al. | Comparative cost-effectiveness of SARS-CoV-2 testing strategies in the USA: a modelling study | The effectiveness is not modelled |
| Elgazzar et al. | Simple mathematical models for controlling COVID-19 transmission through social distancing and community awareness | The effectiveness is not modelled |
| Engebretsen et al. | Regional probabilistic situational awareness and forecasting of COVID-19 | The effectiveness is not modelled |
| Estiri et al. | Individualized prediction of COVID-19 adverse outcomes with MLHO | The effectiveness is not modelled |
| Estrada et al. | COVID-19 and SARS-CoV-2. Modeling the present, looking at the future | The effectiveness is not modelled |
| Farooq et al. | A novel adaptive deep learning model of COVID-19 with focus on mortality reduction strategies | The effectiveness is not modelled |
| Faucher et al. | Reactive vaccination of workplaces and schools against COVID-19 | There is no real-world application |
| Flor et al. | Interactive simulation of disease contagion in dynamic crowds | There is no real-world application |
| Forde et al. | Modeling the influence of vaccine administration on COVID-19 testing strategies | There is no real-world application |
| Fosdick et al. | Model-based evaluation of continued COVID-19 risk at long term care facilities | There is no real-world application |
| Friston et al. | How vaccination and contact isolation might interact to suppress transmission of COVID-19: a DCM study | There is no real-world application |
| Fu et al. | Effect of evidence updates on key determinants of measles vaccination impact: a DynaMICE modelling study in ten high-burden countries | There is no real-world application |
| Fu et al. | Optimal lockdown policy for vaccination during COVID-19 pandemic | The effectiveness is not modelled |
| Fudolig et al. | The local stability of a modified multi-strain SIR model for emerging viral strains | The effectiveness is not modelled |
| Fujimoto et al. | Significance of SARS-CoV-2 specific antibody testing during COVID-19 vaccine allocation | There is no real-world application |
| Gandolfi et al. | A new threshold reveals the uncertainty about the effect of school opening on diffusion of COVID-19 | The effectiveness is not modelled |
| Gandon et al. | Targeted vaccination and the speed of SARS-CoV-2 adaptation | There is no real-world application |
| García-Cremades et al. | Improving prediction of COVID-19 evolution by fusing epidemiological and mobility data | The effectiveness is not modelled |
| Gerli et al. | Forecasting COVID-19 infection trends and new hospital admissions in England due to SARS-CoV-2 variant of concern Omicron | The effectiveness is not modelled |
| Gerli et al. | Forecasting COVID-19 infection trends in the EU-27 countries, the UK and Switzerland due to SARS-CoV-2 variant of concern Omicron | The effectiveness is not modelled |
| Getz et al. | A runtime alterable epidemic model with genetic drift, waning immunity and vaccinations | The effectiveness is not modelled |
| Gevertz et al. | A novel COVID-19 epidemiological model with explicit susceptible and asymptomatic isolation compartments reveals unexpected consequences of timing social distancing | The effectiveness is not modelled |
| Giamberardino et al. | What if vaccinated individuals could be infected? | There is no real-world application |
| Giordano et al. | Modeling vaccination rollouts, SARS-CoV-2 variants and the requirement for non-pharmaceutical interventions in Italy | The effectiveness is not modelled |
| Gog et al. | Vaccine escape in a heterogeneous population: insights for SARS-CoV-2 from a simple model | There is no real-world application |
| Goldenbogen et al. | Optimality in COVID-19 vaccination strategies determined by heterogeneity in human-human interaction networks | The effectiveness is not modelled |
| Goldsztejn et al. | Public policy and economic dynamics of COVID-19 spread: a mathematical modeling study | The effectiveness is not modelled |
| Gomes et al. | Frailty variation models for susceptibility and exposure to SARS-CoV-2 | The effectiveness is not modelled |
| Gram et al. | Vaccine effectiveness against SARS-CoV-2 infection, hospitalization, and death when combining a first dose ChAdOx1 vaccine with a subsequent mRNA vaccine in Denmark: a nationwide population-based cohort study | There is no real-world application |
| Grauer et al. | Strategic spatiotemporal vaccine distribution increases the survival rate in an infectious disease like COVID-19 | The effectiveness is not modelled |
| Gumel et al. | A primer on using mathematics to understand COVID-19 dynamics: Modeling, analysis and simulations | The effectiveness is not modelled |
| Hametner et al. | Intensive care unit occupancy predictions in the COVID-19 pandemic based on age-structured modelling and differential flatness | The effectiveness is not modelled |
| Hamou et al. | On dynamics of fractional incommensurate model of COVID-19 with nonlinear saturated incidence rate | The effectiveness is not modelled |
| Han et al. | Modeling of suppression and mitigation interventions in the COVID-19 epidemics | The effectiveness is not modelled |
| Hartono et al. | Forecasting vaccination growth for COVID-19 using machine learning | The effectiveness is not modelled |
| Hazra et al. | The INDSCI-SIM model for COVID-19 in India | The effectiveness is not modelled |
| Houdroge et al. | Predicting the unpredictable: how dynamic COVID-19 policies and restrictions challenge model forecasts | The effectiveness is not modelled |
| Huang et al. | Modeling of the long-term epidemic Dynamics of COVID-19 in the United States | The effectiveness is not modelled |
| Huang et al. | A model for the spread of infectious diseases compatible with case data | The effectiveness is not modelled |
| Huen et al. | A model of endemic coronavirus infections | The effectiveness is not modelled |
| Huntingford et al. | Optimal COVID-19 vaccine sharing between two nations that also have extensive travel exchanges | The effectiveness is not modelled |
| Hurt et al. | Informing university COVID-19 decisions using simple compartmental models | There is no real-world application |
| Ilyin et al. | A recursive model of the spread of COVID-19: modelling study | The effectiveness is not modelled |
| Inthamoussou et al. | Extended SEIR model for health policies assessment against the COVID-19 pandemic: the case of Argentina | The effectiveness is not modelled |
| Reguly et al. | Microsimulation based quantitative analysis of COVID-19 management strategies | There is no real-world application |
| Jiang et al. | Mathematical models for devising the optimal SARS-CoV-2 strategy for eradication in China, South Korea, and Italy | The effectiveness is not modelled |
| Jiang et al. | Forecasting trend of coronavirus disease 2019 using multi-task weighted TSK fuzzy system | There is no real-world application |
| Jithesh et al. | A model based on cellular automata for investigating the impact of lockdown, migration and vaccination on COVID-19 dynamics | The effectiveness is not modelled |
| John et al. | A Survey on mathematical, machine learning and deep learning models for COVID-19 transmission and diagnosis | There is no real-world application |
| Johnson et al. | COVID-19 and computation for policy | There is no real-world application |
| Joshi et al. | Comparative performance of between-population allocation strategies for SARS-CoV-2 vaccines | There is no real-world application |
| Jovanovic et al. | Modelling voluntary general population vaccination strategies during COVID-19 outbreak: influence of disease prevalence | The effectiveness is not modelled |
| Kalkowska et al. | Updated characterization of poliovirus transmission in Pakistan and Afghanistan and the impacts of different outbreak response vaccine options | The effectiveness is not modelled |
| Kaszowska-Mojsa et al. | Immunity in the ABM-DSGE framework for preventing and controlling epidemics-validation of results | There is no real-world application |
| Kemp et al. | Modelling COVID-19 dynamics and potential for herd immunity by vaccination in Austria, Luxembourg and Sweden | The effectiveness is not modelled |
| Khan et al. | Effect of high and low risk susceptibles in the transmission dynamics of COVID-19 and control strategies | There is no real-world application |
| Khan et al. | A mathematical model for the dynamics of SARS-CoV-2 virus using the Caputo-Fabrizio operator | The effectiveness is not modelled |
| Ko et al. | COVID-19 vaccine priority strategy using a heterogenous transmission model based on maximum likelihood estimation in the Republic of Korea | The effectiveness is not modelled |
| Konishi et al. | COVID-19 epidemics monitored through the logarithmic growth rate and SIR model | The effectiveness is not modelled |
| Krebs et al. | COVID-19 scenarios for comparing the effectiveness of age-specific vaccination regimes, exemplified for the city of Aschaffenburg (Germany) | The effectiveness is not modelled |
| Kreck et al. | Back to the roots: a discrete Kermack-McKendrick model adapted to COVID-19 | The effectiveness is not modelled |
| Kuldeep et al. | 6 Predictor System for tracing COVID-19 spread | There is no real-world application |
| Kuzmenko et al. | Impact of vaccination on the COVID-19 pandemic: bibliometric analysis and cross-country forecasting by Fourier series | The effectiveness is not modelled |
| Larese et al. | Incidence of COVID-19 infection in hospital workers from March 1, 2020 to May 31, 2021 routinely tested, before and after vaccination with BNT162B2 | The effectiveness is not modelled |
| Lefèvre et al. | A chain binomial epidemic with asymptomatic motivated by COVID-19 modelling | There is no real-world application |
| Li et al, | Modeling the impact of mass influenza vaccination and public health interventions on COVID-19 epidemics with limited detection capability | The effectiveness is not modelled |
| Li et al. | Effects of vaccination and non-pharmaceutical interventions and their lag times on the COVID-19 pandemic: comparison of eight countries | The effectiveness is not modelled |
| Liang et al. | Statistical analysis on COVID-19 based on SIR model | There is no real-world application |
| Liao et al. | SIRVD-DL: a COVID-19 deep learning prediction model based on time-dependent SIRVD | The effectiveness is not modelled |
| Libotte et al. | Determination of an optimal control strategy for vaccine administration in COVID-19 pandemic treatment | The effectiveness is not modelled |
| Lin et al. | Effectiveness of non-pharmaceutical interventions and vaccine for containing the spread of COVID-19: three illustrations before and after vaccination periods | The effectiveness is not modelled |
| Lu et al. | Cure and death play a role in understanding dynamics for COVID-19: data-driven competing risk compartmental models, with and without vaccination | The effectiveness is not modelled |
| Madewell et al. | Challenges of evaluating and modelling vaccination in emerging infectious diseases | The effectiveness is not modelled |
| Mai et al. | EpiPolicy: a tool for combating epidemics | There is no real-world application |
| Medeiros et al. | Short-term COVID-19 forecast for latecomers | The effectiveness is not modelled |
| Miller et al. | Assessing the risk of vaccine-driven virulence evolution in SARS-CoV-2 | There is no real-world application |
| Nakamoto et al. | Evaluation of the effects of vaccination regimes on the transmission dynamics of COVID-19 pandemic | There is no real-world application |
| Nakata et al. | Feedback balancing between vaccination and quarantine for disease control: a simulation for COVID-19 in Japan | The effectiveness is not modelled |
| Nana-Kyere et al. | Global analysis and optimal control model of COVID-19 | There is no real-world application |
| Navascués et al. | Disease control as an optimization problem | There is no real-world application |
| Nourbakhsh et al. | A wastewater-based epidemic model for SARS-CoV-2 with application to three Canadian cities | The effectiveness is not modelled |
| O’Dea et al. | A semi-parametric, state-space compartmental model with time-dependent parameters for forecasting COVID-19 cases, hospitalizations, and deaths | The effectiveness is not modelled |
| Olivares et al. | Optimal control-based vaccination and testing strategies for COVID-19 | There is no real-world application |
| Olivares et al. | Uncertainty quantification of a mathematical model of COVID-19 transmission dynamics with mass vaccination strategy | There is no real-world application |
| Paetzold et al. | Impacts of rapid mass vaccination against SARS-CoV2 in an early variant of concern hotspot | The effectiveness is not modelled |
| Paltiel et al. | Clinical outcomes of a COVID-19 vaccine: implementation over efficacy | There is no real-world application |
| Penney et al. | Hot-spotting to improve vaccine allocation by harnessing digital contact tracing technology: an application of percolation theory | The effectiveness is not modelled |
| Rahman et al. | A review of COVID-19 modelling strategies in three countries to develop a research framework for regional areas | There is no real-world application |
| Rajaei et al. | State estimation-based control of COVID-19 epidemic before and after vaccine development | There is no real-world application |
| Ram et al. | A modified age-structured SIR model for COVID-19 type viruses | The effectiveness is not modelled |
| Rao et al. | Optimal allocation of limited vaccine to minimize the effective reproduction number | There is no real-world application |
| Rella et al. | Rates of SARS-CoV-2 transmission and vaccination impact the fate of vaccine-resistant strains | There is no real-world application |
| Roberts et al. | Estimating COVID-19 vaccination effectiveness using electronic health records of an academic medical center in Michigan | The effectiveness is not modelled |
| Robinson et al. | Comprehensive compartmental model and calibration algorithm for the study of clinical implications of the population-level spread of COVID-19: a study protocol | There is no real-world application |
| Robles-Fontan et al. | Time-varying effectiveness of the mRNA-1273, BNT162b2 and Ad26.COV2.S vaccines against SARS-CoV-2 infections and COVID-19 hospitalizations and deaths: an analysis based on observational data from Puerto Rico | The effectiveness is not modelled |
| Rochman et al. | Substantial impact of post-vaccination contacts on cumulative infections during viral epidemics | The effectiveness is not modelled |
| Routledge et al. | Using sero-epidemiology to monitor disparities in vaccination and infection with SARS-CoV-2 | The effectiveness is not modelled |
| Rustagi et al. | Analyzing the effect of vaccination over COVID cases and deaths in Asian countries using machine learning models | The effectiveness is not modelled |
| Saad-Roy et al | Trajectory of individual immunity and vaccination required for SARS-CoV-2 community immunity: a conceptual investigation | The effectiveness is not modelled |
| Saad-Roy et al. | Vaccine breakthrough and the invasion dynamics of SARS-CoV-2 variants | There is no real-world application |
| Saad-Roy et al. | Epidemiological and evolutionary considerations of SARS-CoV-2 vaccine dosing regimes | The effectiveness is not modelled |
| Sagar et al. | Trend analysis and comparison of COVID – 19 cases before and after administration of vaccine using machine learning | There is no real-world application |
| Salvadore et al. | Integro-differential approach for modeling the COVID-19 dynamics – Impact of confinement measures in Italy | The effectiveness is not modelled |
| Sararat et al. | Community vaccination can shorten the COVID-19 isolation period: an individual-based modeling approach | There is no real-world application |
| Saunders et al. | COVID-19 vaccination strategies depend on the underlying network of social interactions | There is no real-world application |
| Schwarzendahl et al. | Mutation induced infection waves in diseases like COVID-19 | The effectiveness is not modelled |
| Shirin et al. | Data-driven optimized control of the COVID-19 epidemics | The effectiveness is not modelled |
| Smid et al. | SEIR filter: a stochastic model of epidemic | The effectiveness is not modelled |
| Sokolov et al. | Monitoring and forecasting the COVID-19 epidemic in Moscow: model selection by balanced identification technology – version: September 2021 | The effectiveness is not modelled |
| Southall et al. | Prospects for detecting early warning signals in discrete event sequence data: application to epidemiological incidence data | The effectiveness is not modelled |
| Spinella et al. | Simulation of the impact of people mobility, vaccination rate, and virus variants on the evolution of COVID-19 outbreak in Italy | The effectiveness is not modelled |
| Stoddard et al. | Beyond the new normal: assessing the feasibility of vaccine-based suppression of SARS-CoV-2 | There is no real-world application |
| Stoddard et al. | Individually optimal choices can be collectively disastrous in COVID-19 disease control | There is no real-world application |
| Topîrceanu et al. | A novel geo-hierarchical population mobility model for spatial spreading of resurgent epidemics | The effectiveness is not modelled |
| Treibert et al | A nonstandard finite difference scheme for the SVICDR model to predict COVID-19 dynamics | The effectiveness is not modelled |
| Tutsoy et al. | Pharmacological, non-pharmacological policies and mutation: an artificial intelligence based multi-dimensional policy making algorithm for controlling the casualties of the pandemic diseases | The effectiveness is not modelled |
| Van Egeren et al. | Rapid relaxation of pandemic restrictions after vaccine rollout favors growth of SARS-CoV-2 variants: a model-based analysis | The effectiveness is not modelled |
| Volpert et al. | Epidemic progression and vaccination in a heterogeneous population. Application to the COVID-19 epidemic | The effectiveness is not modelled |
| Wagner et al. | Vaccine nationalism and the dynamics and control of SARS-CoV-2 | The effectiveness is not modelled |
| Wang et al. | Predicting the epidemics trend of COVID-19 using epidemiological-based generative adversarial networks | There is no real-world application |
| Wang et al. | Dynamic assessment of population by using SIRI model | There is no real-world application |
| Xiao et al. | Optimal reopening pathways with COVID-19 vaccine rollout and emerging variants of concern | The effectiveness is not modelled |
| Xu et al. | A continuous Markov-Chain model for the simulation of COVID-19 epidemic dynamics | The effectiveness is not modelled |
| Xu et al. | Control strategies for COVID-19 epidemic with vaccination, shield immunity and quarantine: a metric temporal logic approach | The effectiveness is not modelled |
| Yang et al. | Assessing vaccination priorities for different ages and age-specific vaccination strategies of COVID-19 using an SEIR modelling approach | The effectiveness is not modelled |
| Zhang et al. | An integrated framework for building trustworthy data-driven epidemiological models: application to the COVID-19 outbreak in New York City | The effectiveness is not modelled |
| Zhang et al. | A prognostic dynamic model applicable to infectious diseases providing easily visualized guides: a case study of COVID-19 in the UK | The effectiveness is not modelled |
| Zhao et al. | The impact of awareness diffusion on the spread of COVID-19 based on a two-layer SEIR/V-UA epidemic model | The effectiveness is not modelled |
| Zuo et al. | Analyzing COVID-19 vaccination behavior using an SEIRM/V epidemic model with awareness decay | The effectiveness is not modelled |
| Zuo et al. | Analyzing the COVID-19 vaccination behavior based on epidemic model with awareness-information | The effectiveness is not modelled |
