# Supplementary material for: Predictive models for health outcomes due to SARS-CoV-2, including the effect of vaccination: a systematic review
Source: Syst Rev. 2024 Jan 16;13:30. doi: 10.1186/s13643-023-02411-1 (PMC10790449; doi:10.1186/s13643-023-02411-1)
Supplement: Supplementary file 3 — Supplementary Material N°. 3. Variables’ operational definition. [file 13643_2023_2411_MOESM3_ESM.zip › 12903_2024_5066_MOESM3_ESM/Supp. N°. 3B.docx]

# Supplementary material N°. 3. Variables’ operational definition

| **Variables** | **Definition** |
| --- | --- |
| Authors | The names and surnames of the articles’ authors. |
| Year | The year in which the article was published. |
| Title | The name of the article. |
| Journal | The journal in which the article was published. |
| Abstract | The abstract provided in the article. |
| Keywords | A list of keywords reported in the article |
| DOI | Unique identifier for the article. |
| Language | The language in which the article is written. |
| Macro model | Provides a general indication of the type of model developed in the article. |
| Compartments | A list of compartments or states used in the model, if included. |
| Parameters | A list of parameters used in the model. |
| Location | The place for which the model is estimated. |
| Country | The country for which the model is estimated. |
| Country income | Classification of countries based on their income level according to the World Bank. |
| The model is developed | Indicates whether there is a mathematical development of the model in the article and/or supplements. |
| Software | Indicates the software used by the authors for model estimation when available. |
| Programming code available | Indicates whether the model estimation code is open access and provides the web address if applicable. |
| Dashboard | Denotes the presence of a results dashboard, with the web address provided if applicable. |
| Temporary unit | Reflects the periodicity with which the model results are presented. |
| Includes comorbidities | Indicates whether the estimated model accounts for comorbidities. |
| No. types of vaccine | Specific the number of vaccines against SARS-CoV-2 used in the model. |
| The difference in effectiveness between vaccines | Indicates, for the case when two or more vaccines are modelled, whether different efficacies are considered for each vaccine. |
| Number of doses | Presents the number of doses per vaccine implemented in the model. |
| The difference in days between doses | Indicates if the model considers differentiation in the number of days between vaccine doses. |
| Effectiveness/Efficacy of the vaccine(s) | Presents the effectiveness/efficacy value of the vaccine(s) used in the model. |
| Heterologous vaccination | Indicate whether the model considers combined vaccine platforms. |
| Studied the possibility of reinfection | Indicates whether the model accounts for the reinfection of individuals. |
| Projects or replicates observed results | Indicates whether model results are projections, estimates (replications of observed periods), or both. |
| Strains | A list of strains considered in the model if indicated in the article. |
| Differentiation by age | Indicates if model results are differentiated by age and provides age groups of analysis if applicable. |
| Differentiation by sex | Indicates if model results are differentiated by sex. |
| Differentiation by race/ethnicity | Indicates if model results are differentiated by race/ethnicity. |
| Geographic area differentiation | Indicates whether model results are differentiated by geographical area within the same study country. |
| Non-pharmaceutical interventions | A list of non-pharmaceutical interventions considered in the model. |
| Prioritize by occupation | Indicates whether there is a prioritization of vaccination based on occupation. |
| Socioeconomic characteristics of the population | Indicate whether sociodemographic characteristics are considered for modelling and lists these characteristics if applicable. |
| Inclusion of migrants | Indicates whether migrants are included in the model. |
| Health outcomes | A list of health outcomes for which results are obtained through modeling. |
