# Supplementary material for: Predictive models for health outcomes due to SARS-CoV-2, including the effect of vaccination: a systematic review
Source: Syst Rev. 2024 Jan 16;13:30. doi: 10.1186/s13643-023-02411-1 (PMC10790449; doi:10.1186/s13643-023-02411-1)
Supplement: Supplementary file 4 — Supplementary Material N°. 4. Checklist - Methodological quality. [file 13643_2023_2411_MOESM4_ESM.docx]

# Supplementary material N°. 4. Checklist - Methodological quality

**Joanna Briggs Institute – Cross-sectional studies**


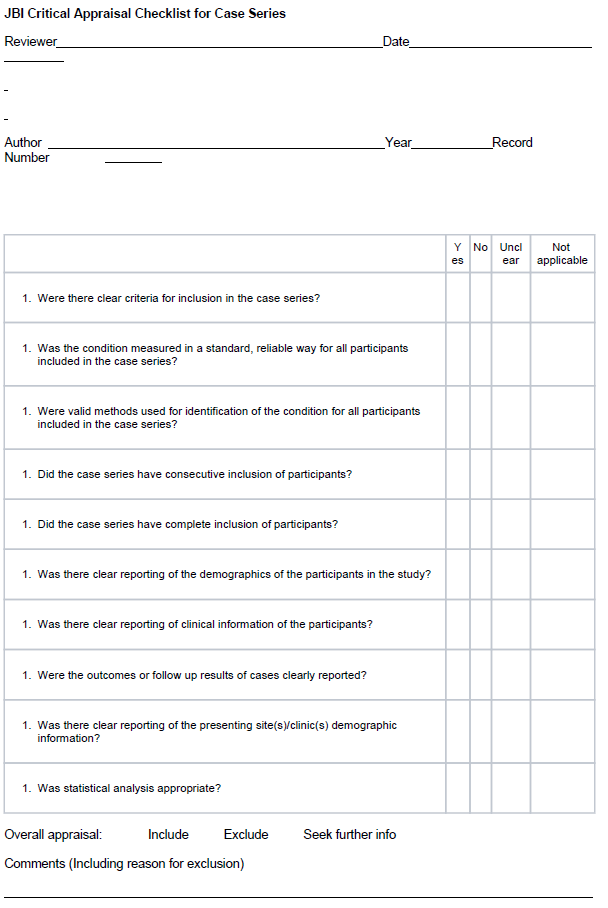


Source: taken from Joanna Briggs Institute (76)

**Joanna** **Briggs Institute – Cohort studies**


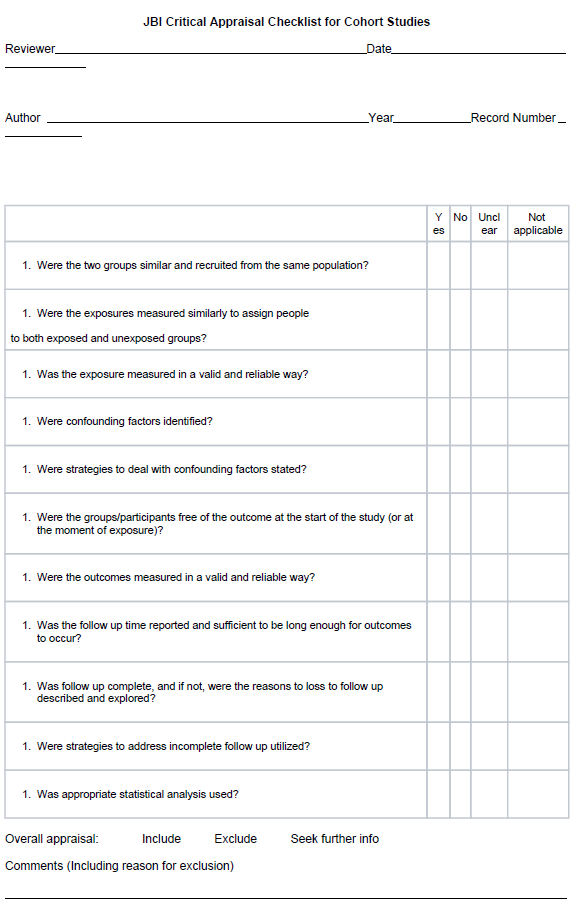


Source: taken from Joanna Briggs Institute (76)

**Joanna** **Briggs Institute – Case-control studies**


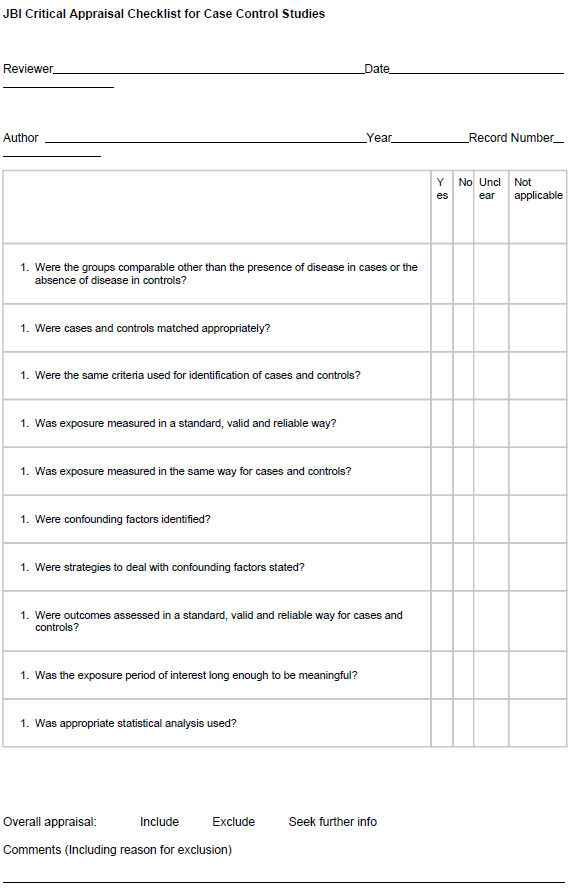


Source: taken from Joanna Briggs Institute (76)

**Tool PROBAST**


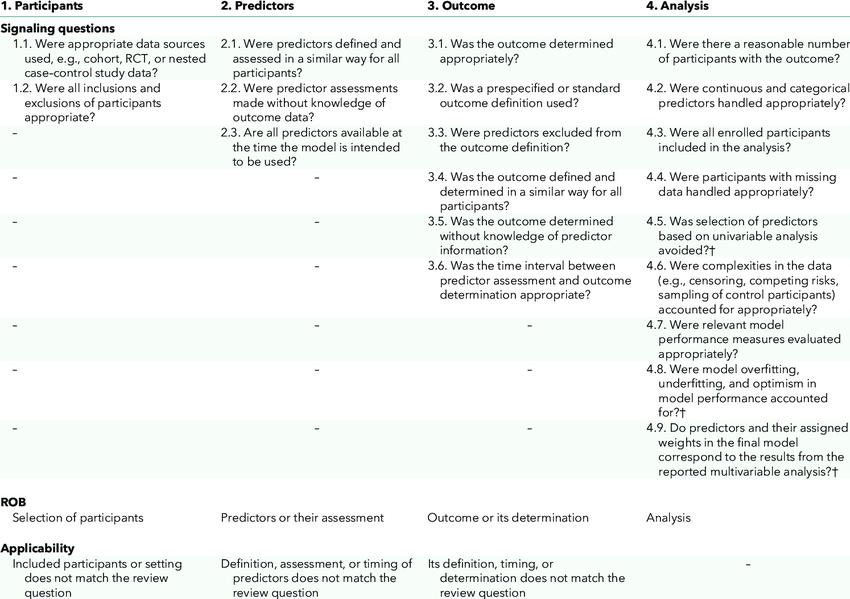


Source: taken from PROBAST (77)
