# Supplementary material for: Predictive models for health outcomes due to SARS-CoV-2, including the effect of vaccination: a systematic review
Source: Syst Rev. 2024 Jan 16;13:30. doi: 10.1186/s13643-023-02411-1 (PMC10790449; doi:10.1186/s13643-023-02411-1)
Supplement: Supplementary file 5 — Supplementary Material N°. 5. PRISMA flow chart. [file 13643_2023_2411_MOESM5_ESM.docx]

# Supplementary material N°. 5. PRISMA flow chart

**Identified results from databases**

(n = 5.646)

**Results found from other sources**

(n = 197)

**Identification**

**Results**

(n = 5.843)

**Duplicated references**

(n = 2.362)

**Excluded references**

(n = 3.083)

**Title and abstract screened references**

(n = 3.481)

**Excluded references:**

Out of the research question scope (n = 139)

No real-world application (n = 57)

**Screening**

**Full-text screening assesment**

(n = 398)

**References added using snow-balling method**

(n = 209)

**Included references**

(n = 202)

**Studies included into this review**

(n = 411)

**Included**
