# Supplementary material for: Predictive models for health outcomes due to SARS-CoV-2, including the effect of vaccination: a systematic review
Source: Syst Rev. 2024 Jan 16;13:30. doi: 10.1186/s13643-023-02411-1 (PMC10790449; doi:10.1186/s13643-023-02411-1)
Supplement: Supplementary file 6 — Supplementary Material N°. 6. List of included studies. [file 13643_2023_2411_MOESM6_ESM.docx]

# Supplementary material N°. 6. List of included studies

| **Authors** | **Year** | | **Title** | | **DOI** |
| --- | --- | --- | --- | --- | --- |
| Acuña-Zegarra et al. | 2021 | | COVID-19 optimal vaccination policies: a modeling study on efficacy, natural and vaccine-induced immunity responses | | 10.1016/j.mbs.2021.108614 |
| Adibi et al. | 2021 | | Continuing COVID-19 vaccination of front-line workers in british columbia with the astrazeneca vaccine: benefits in the face of increased risk for prothrombotic thrombocytopenia | | 10.1101/2021.04.11.21255138 |
| Adiga et al. | 2021 | | Strategies to mitigate COVID-19 resurgence assuming immunity waning: a study for Karnataka, India | | 10.1101/2021.05.26.21257836 |
| Agarwal et al. | 2021 | | The trade-off between prioritization and vaccination speed depends on mitigation measures | | 10.1101/2021.02.24.21252352 |
| Aguas et al. | 2021 | | Potential global impacts of alternative dosing regimen and rollout options for the ChAdOx1 nCoV-19 vaccine | | 10.1038/s41467-021-26449-8 |
| Aguiar et al. | 2021 | | The role of mild and asymptomatic infections on COVID-19 vaccines performance: a modeling study | | 10.1016/j.jare.2021.10.012 |
| Aguilar-Canto et al. | 2022 | | Sensitivity theorems of a model of multiple imperfect vaccines for COVID-19 | | [10.1016/j.chaos.2022.111844](https://doi.org/10.1016/j.chaos.2022.111844) |
| Aguilar-Canto et al. | 2021 | | SIR-based model with multiple imperfect vaccines | | [10.1101/2021.05.07.21256860](https://doi.org/10.1101/2021.05.07.21256860) |
| Ainslie et al. | 2021 | | The impact of vaccinating adolescents and children on COVID-19 disease outcomes | | [10.1101/2021.10.21.21265318](https://doi.org/10.1101/2021.10.21.21265318) |
| Alagoz et al. | 2021 | | The impact of vaccination to control COVID-19 burden in the United States: a simulation modeling approach | | 10.1371/journal.pone.0254456 |
| Albani et al. | 2021 | | COVID-19 underreporting and its impact on vaccination strategies | | 10.1186/s12879-021-06780-7 |
| Albani et al. | 2021 | | The impact of COVID-19 vaccination delay: a data-driven modeling analysis for Chicago and New York City | | 10.1016/j.vaccine.2021.08.098 |
| Aldila et al. | 2021 | | Impact of early detection and vaccination strategy in COVID-19 eradication program in Jakarta, Indonesia | | 10.1186/s13104-021-05540-9 |
| Almajose et al. | 2021 | | A 2SIR-VD model for optimizing geographical COVID-19 vaccine distribution in the Philippines | | 10.1101/2021.05.20.21257556 |
| Amaku et al. | 2021 | | Modelling the impact of delaying vaccination against SARS-CoV-2 assuming unlimited vaccine supply | | 10.1186/s12976-021-00143-0 |
| Amaral et al. | 2021 | | Simulating immunization campaigns and vaccine protection against COVID-19 pandemic in Brazil | | 10.1109/ACCESS.2021.3112036 |
| Amouch et al. | 2021 | | Modeling the dynamic of COVID-19 with different types of transmissions | | 10.1016/j.chaos.2021.111188 |
| Antonini et al. | 2021 | | A modeling study on vaccination and spread of SARS-CoV-2 variants in Italy | | 10.3390/vaccines9080915 |
| Are et al. | 2021 | | COVID-19 endgame: from pandemic to endemic? vaccination, reopening and evolution in a well-vaccinated population | | 10.1101/2021.12.18.21268002 |
| Arslan et al. | 2021 | | COVID-19 alert stages, healthcare projections and mortality patterns in Austin, Texas, may 2021 | | - |
| Arslan et al. | 2021 | | Projections for Austin’s COVID-19 staged alert sytem, incorporating reported cases as additional indicator | | [covid-19.tacc.utexas.edu/media/filer_public/f9/58/f95850cc-1428-4966-8873-601bae0dc0e6/projections_for_austins_covid-19_staged_alert_system_incorporating_reported_cases_as_additional_indicator.pdf](https://covid-19.tacc.utexas.edu/media/filer_public/f9/58/f95850cc-1428-4966-8873-601bae0dc0e6/projections_for_austins_covid-19_staged_alert_system_incorporating_reported_cases_as_additional_indicator.pdf) |
| Aruffo et al. | 2021 | | Mathematical modeling of vaccination rollout and npis lifting on COVID-19 transmission with voc: a case study in Toronto, Canada | | [10.1101/2021.08.11.21261932](https://doi.org/10.1101/2021.08.11.21261932) |
| Aruffo et al. | 2021 | | Community structured model for vaccine strategies to control covid19 spread: a mathematical study | | 10.1101/2021.01.25.21250505 |
| Avila-Ponce de León et al. | 2022 | | Modeling the transmission of the SARS-CoV-2 Delta variant in a partially vaccinated population | | 10.3390/v14010158 |
| Ayoub et al. | 2021 | | Epidemiological impact of prioritising SARS-CoV-2 vaccination by antibody status: mathematical modelling analyses | | 10.1136/bmjinnov-2021-000677 |
| Ayoub et al. | 2022 | | Estimating protection afforded by prior infection in preventing reinfection: applying the test-negative study design | | [10.1101/2022.01.02.22268622](https://doi.org/10.1101/2022.01.02.22268622) |
| Bablani et al. | 2021 | | Can a vaccine-led approach end the NSW outbreak in 100 days, or at least substantially reduce morbidity and mortality? | | 10.1101/2021.08.18.21262252 |
| Babus et al. | 2020 | | The optimal allocation of COVID-19 vaccines | | [10.1101/2020.07.22.20160143](https://doi.org/10.1101/2020.07.22.20160143) |
| Badfar et al. | 2021 | | Design a robust sliding mode controller based on the state and parameter estimation for the nonlinear epidemiological model of COVID-19 | | 10.1007/s11071-021-07036-4 |
| Barlow et al. | 2021 | | Optimal shutdown strategies for COVID-19 with economic and mortality costs: British Columbia as a case study | | 10.1098/rsos.202255 |
| Barmpounakis et al. | 2022 | | Evaluating the effects of second-dose vaccine-delay policies in european countries: a simulation study based on data from Greece | | [10.1371/journal.pone.0263977](https://doi.org/10.1371/journal.pone.0263977) |
| Barnard et al. | 2021 | | Projected epidemiological consequences of the Omicron SARS-CoV-2 variant in England, December 2021 to April 2022 | | 10.1101/2021.12.15.21267858 |
| Barnard et al. | 2021 | | Behaviour, booster vaccines and waning vaccine protection: modelling the medium-term dynamics of SARS-CoV-2 transmission in England | | 10.1101/2021.11.22.21266584 |
| Barreiro et al. | 2021 | | Modelling the interplay of SARS-CoV-2 variants in the United Kingdom | | [10.1101/2021.11.26.21266485](https://doi.org/10.1101/2021.11.26.21266485) |
| Bartsch et al. | 2021 | | Lives and costs saved by expanding and expediting Coronavirus disease 2019 vaccination | | 10.1093/infdis/jiab233 |
| Bartsch et al. | 2021 | | The benefits of vaccinating with the first available COVID-19 Coronavirus vaccine | | 10.1016/j.amepre.2021.01.001 |
| Bartsch et al. | 2020 | | Vaccine efficacy needed for a COVID-19 coronavirus vaccine to prevent or stop an epidemic as the sole intervention | | [10.1016/j.amepre.2020.06.011](https://doi.org/10.1016/j.amepre.2020.06.011) |
| Bauer et al. | 2021 | | Relaxing restrictions at the pace of vaccination increases freedom and guards against further COVID-19 waves | | 10.1371/journal.pcbi.1009288 |
| Bautista et al. | 2021 | | COVID-19 immunization threshold(s): an analysis | | 10.1101/2021.01.02.20248596 |
| Below et al. | 2021 | | The impact of vaccination on the spread patterns of the covid epidemic | | [10.1101/2021.04.29.21256322](https://doi.org/10.1101/2021.04.29.21256322) |
| Berec et al. | 2022 | | Importance of vaccine action and availability and epidemic severity for delaying the second vaccine dose | | [10.1038/s41598-022-11250-4](https://doi.org/10.1038/s41598-022-11250-4) |
| Berkane et al. | 2021 | | Modeling the effect of population-wide vaccination on the evolution of COVID-19 epidemic in Canada | | [10.1101/2021.02.05.21250572](https://doi.org/10.1101/2021.02.05.21250572) |
| Bertsimas et al. | 2021 | | Where to locate COVID-19 mass vaccination facilities? | | 10.1002/nav.22007 |
| Bhattacharya et al. | 2021 | | AI-driven agent-based models to study the role of vaccine acceptance in controlling COVID-9 spread in the US | | 10.1109/BigData52589.2021.9671811 |
| Bianchin et al. | 2021 | | When can we safely return to normal? A novel method for identifying safe levels of NPIs in the context of COVID-19 vaccinations | | [10.1101/2021.04.20.21255350](https://doi.org/10.1101/2021.04.20.21255350) |
| Bilinski et al. | 2021 | | Passing the test: a model-based analysis of safe school-reopening strategies | | 10.7326/M21-0600 |
| Bilinski et al. | 2021 | | SARS-CoV-2 testing strategies to contain school-associated transmission: model-based analysis of impact and cost of diagnostic testing, screening, and surveillance | | 10.1101/2021.05.12.21257131 |
| Booton et al. | 2021 | | Modelling the effect of COVID-19 mass vaccination on acute admissions in a major English healthcare system | | 10.1101/2021.10.10.21264821 |
| Borchering et al. | 2022 | | Impact of SARS-CoV-2 vaccination of children ages 5-11 years on COVID-19 disease burden and resilience to new variants in the United States, November 2021-March 2022: a multi-model study | | 10.1101/2022.03.08.22271905 |
| Borchering et al. | 2021 | | Modeling of future COVID-19 cases, hospitalizations, and deaths, by vaccination rates and nonpharmaceutical intervention scenarios: United States, April-September 2021 | | 10.15585/mmwr.mm7019e3 |
| Bosetti et al. | 2021 | | A race between SARS-CoV-2 variants and vaccination: the case of the B.1.1.7 variant in France | | pasteur-03149525 |
| Bosetti et al. | 2022 | | Epidemiology and control of SARS-CoV-2 epidemics in partially vaccinated populations: a modeling study applied to France | | 10.1186/s12916-022-02235-1 |
| Bosetti et al. | 2022 | | Impact of booster vaccination on the control of COVID-19 Delta wave in the context of waning immunity: application to France in the winter 2021/22 | | 10.2807/1560-7917.ES.2022.27.1.2101125 |
| Bousquet et al. | 2022 | | Deep learning forecasting using time-varying parameters of the SIRD model for COVID-19 | | 10.1038/s41598-022-06992-0 |
| Bowie et al. | 2021 | | A twelve-month projection to september 2022 of the COVID-19 epidemic in the UK using a Dynamic Causal Model | | 10.1101/2021.10.04.21262827 |
| Bracis et al. | 2022 | | Improving vaccination coverage and offering vaccine to all school-age children allowed uninterrupted in-person schooling in King County, WA: modeling analysis | | 10.3934/mbe.2022266 |
| Braun et al. | 2020 | | COVID-19 pandemic predictions using the modified Bateman SIZ model and observational data for Heidelberg, Germany: effect of vaccination with a SARS-CoV-2 vaccine, coronavirus testing and application of the Corona-Warn-App | | 10.5414/CP203846 |
| Brown et al. | 2021 | | A simple model for control of COVID-19 infections on an urban campus | | 10.1073/pnas.2105292118 |
| Brüningk et al. | 2022 | | Determinants of SARS-CoV-2 transmission to guide vaccination strategy in a city | | 10.1093/ve/veac002 |
| Bu et al. | 2021 | | Forecast of the development of COVID-19 based on the small-world network | | 10.1145/3507548.3507575 |
| Bubar et al. | 2021 | | Model-informed COVID-19 vaccine prioritization strategies by age and serostatus | | 10.1126/science.abe6959 |
| Buchwald et al. | 2021 | | Colorado COVID-19 mathematical model documentation prepared by the Colorado COVID-19 modeling group | | - |
| Buckner et al. | 2021 | | Dynamic prioritization of COVID-19 vaccines when social distancing is limited for essential workers | | 10.1073/pnas.2025786118 |
| Bugalia et al. | 2022 | | Mutations make pandemics worse or better: modeling SARS-CoV-2 variants and imperfect vaccination | | [arxiv.org/abs/2201.06285](https://arxiv.org/abs/2201.06285) |
| Buhat et al. | 2021 | | Using constrained optimization for the allocation of COVID-19 vaccines in the Philippines | | [10.1007/s40258-021-00667-z](https://doi.org/10.1007/s40258-021-00667-z) |
| Buonomo et al. | 2022 | | A behavioural modelling approach to assess the impact of COVID-19 vaccine hesitancy | | 10.1016/j.jtbi.2021.110973 |
| Caetano et al. | 2021 | | Measuring the impact of COVID-19 vaccination and immunity waning: a modelling study for Portugal | | [10.1101/2021.12.10.21267618](https://doi.org/10.1101/2021.12.10.21267618) |
| Cai et al. | 2022 | | Assessing the transition of COVID-19 burden towards the young population while vaccines are rolled out in China | | 10.1080/22221751.2022.2063073 |
| Campos et al. | 2021 | | Multi-generational SIR modeling: determination of parameters, epidemiological forecasting and age-dependent vaccination policies | | 10.1016/j.idm.2021.05.003 |
| Canga et al. | 2022 | | Modelling the effect of the interaction between vaccination and non-pharmaceutical measures on COVID-19 incidence | | 10.1101/2021.11.29.21266986 |
| Catalá et al. | 2021 | | The impact of rioritization and dosing intervals on the effects of COVID-19 vaccination in Europe: an agent-based cohort model | | 10.1038/s41598-021-98216-0 |
| Cazelles et al. | 2021 | | Dynamics of the COVID-19 epidemic in Ireland under mitigation | | 10.1186/s12879-021-06433-9 |
| Chang et al. | 2021 | | Nowcasting transmission and suppression of the delta variant of SARS-CoV-2 in Australia | | 10.21203/rs.3.rs-757351/v1 |
| Chapman et al. | 2022 | | Risk factor targeting for vaccine prioritization during the COVID-19 pandemic | | 10.1038/s41598-022-06971-5 |
| Chapman et al. | 2022 | | Unexposed populations and potential COVID-19 hospitalisations and deaths in rioriti countries as per data up to 21 November 2021 | | [10.2807/1560-7917.ES.2022.27.1.2101038](https://doi.org/10.2807/1560-7917.ES.2022.27.1.2101038) |
| Chen et al. | 2021 | | Fitting and predicting trend of COVID-19 by SVEPIUHDR dynamic model | | 10.3760/cma.j.cn112338-20210225-00147 |
| Chen et al. | 2022 | | Impact of vaccination on the COVID-19 pandemic in U.S. states | | 10.1038/s41598-022-05498-z |
| Chen et al. | 2021 | | Impacts of vaccination and Severe Acute Respiratory Syndrome Coronavirus 2 variants Alpha and Delta on Coronavirus Disease 2019 transmission dynamics in the 15 most populous metropolitan statistical areas in the United States | | 10.1101/2021.10.19.21265223 |
| Chen et al. | 2021 | | Prioritizing allocation of COVID-19 vaccines based on social contacts increases vaccination effectiveness | | 10.1101/2021.02.04.21251012 |
| Chen et al. | 2021 | | Age-stratified COVID-19 spread analysis and vaccination: a multitype random network approach | | 10.1109/TNSE.2021.3075222 |
| Chen et al. | 2020 | | Allocation of COVID-19 vaccines under limited supply | | 10.1101/2020.08.23.20179820 |
| Chen et al. | 2021 | | An optimization framework to study the balance between expected fatalities due to COVID-19 and the reopening of U.S. communities | | 10.1109/TASE.2021.3119930 |
| Childs et al. | 2021 | | Modeling waning and boosting of COVID-19 in Canada with vaccination | | 10.1101/2021.05.18.21257426 |
| Chinazzi et al. | 2020 | | Estimating the effect of cooperative versus uncooperative strategies of COVID-19 vaccine allocation: a modeling study | | - |
| Chinchilla et al. | 2021 | | A tale of two doses: model identification and optimal vaccination for COVID-19 | | 10.1109/CDC45484.2021.9683311 |
| Choi et al. | 2021 | | Vaccination prioritization strategies for COVID-19 in Korea: a mathematical modeling approach | | 10.3390/ijerph18084240 |
| Choi et al. | 2021 | | Vaccine effects on susceptibility and symptomatology can change the optimal allocation of COVID-19 vaccines: South Korea as an example | | 10.3390/jcm10132813 |
| Chopra et al. | 2021 | | DeepABM: scalable and efficient agent-based simulations via geometric learning frameworks: a case study for COVID-19 spread and interventions | | 10.1109/WSC52266.2021.9715507 |
| Chu et al. | 2021 | | What vaccination rate(s) rioriti total societal costs after ‘Opening Up’ to COVID-19? Age-Structured SIRM results for the Delta Variant in Australia (New South Wales, Victoria and Western Australia) | | dx.10.2139/ssrn.3944437 |
| Chun et al. | 2021 | | COVID-19 vaccine prioritisation in Japan and South Korea | | 10.1101/2021.04.16.21255649 |
| Chun et al. | 2022 | | Age-varying susceptibility to the delta variant (B16172) of SARS-CoV-2 | | 10.1001/jamanetworkopen.2022.3064 |
| Cipriano et al. | 2021 | | Mitigating the 4^th^ wave of the COVID-19 pandemic in Ontario | | 10.1101/2021.09.02.21263000 |
| Cohen et al. | 2022 | | The changing impact of vaccines in the COVID-19 pandemic | | 10.1101/2022.03.10.22272222 |
| Colomer et al. | 2021 | | Modelling the SARS-CoV-2 outbreak: assessing the usefulness of protective measures to reduce the pandemic at population level | | 10.1016/j.scitotenv.2021.147816 |
| Colosi et al. | 2022 | | Screening and vaccination against COVID-19 to minimize school closure | | 10.1016/S1473-3099(22)00138-4 |
| Colosi et al. | 2022 | | Minimizing school disruption under high incidence conditions due to the Omicron variant in early 2022 | | 10.1101/2022.02.04.22270473 |
| Conn et al. | 2021 | | Mechanistic model calibration and the dynamics of the COVID-19 epidemic in the UK (the past, the present and the future) | | 10.1101/2021.05.18.21257384 |
| Contreras et al. | 2022 | | Impact of the representation of contact data on the evaluation of interventions in infectious diseases simulations | | 10.1101/2022.02.28.22271600 |
| Contreras et al. | 2021 | | Low case numbers enable long-term stable pandemic control without lockdowns | | 10.1126/sciadv.abg2243 |
| Contreras et al. | 2021 | | The winter dilemma | | 10.48550/arXiv.2110.01554 |
| Cook et al. | 2021 | | Impact of vaccination by priority group on UK deaths, hospital admissions and intensive care admissions from COVID-19 | | 10.1111/anae.15442 |
| Coudeville et al. | 2021 | | Potential impact of introducing vaccines against COVID-19 under supply and uptake constraints in France: a modelling study | | 10.1371/journal.pone.0250797 |
| Cruz et al. | 2021 | | Simulation-based evaluation of school reopening strategies during COVID-19: a case study of São Paulo, Brazil | | [10.1017/S0950268821001059](https://doi.org/10.1017/S0950268821001059) |
| Cuesta-Lazaro et al. | 2021 | | Vaccinations or non-pharmaceutical interventions: safe reopening of schools in England | | 10.1101/2021.09.07.21263223 |
| Dagpunar et al. | 2021 | | A prototype vaccination model for endemic COVID-19 under waning immunity and imperfect vaccine take-up | | 10.1101/2021.11.06.21266002 |
| Darapaneni et al. | 2021 | | Forecasting vaccination drive in India for Herd immunity using SIR and prophet model | | [10.1109/AIIoT52608.2021.9454186](https://doi.org/10.1109/AIIoT52608.2021.9454186) |
| David et al. | 2022 | | Non-pharmaceutical intervention levels to reduce the COVID-19 attack ratio among children | | 10.1098/rsos.211863 |
| Davies et al. | 2021 | | Estimated transmissibility and impact of SARS-CoV-2 lineage B.1.1.7 in England | | [10.1126/science.abg3055](https://doi.org/10.1126/science.abg3055) |
| de Cellès et al. | 2022 | | Immunological heterogeneity informs estimation of the durability of COVID-19 vaccine protection | | 10.1098/rsif.2022.0070 |
| de La Sen et al. | 2021 | | On a discrete seir epidemic model with two-doses delayed feedback vaccination control on the susceptible | | 10.3390/vaccines9040398 |
| de La Sen et al. | 2021 | | On a new SEIRDE o I o epidemic model eventually initiated from outside with delayed re-susceptibility and vaccination and treatment feedback controls | | 10.1088/1402-4896/ac018c |
| Avila-Ponce de León et al. | 2022 | | Modeling COVID-19 dynamic using a two-strain model with vaccination | | 10.1016/j.chaos.2022.111927 |
| de Lima et al. | 2021 | | Reopening California: seeking robust, non-dominated COVID-19 exit strategies | | 10.1371/journal.pone.0259166 |
| De Visscher et al. | 2021 | | Second-wave dynamics of COVID-19: impact of behavioral changes, immunity loss, new strains, and vaccination | | 10.21203/rs.3.rs-195879/v1 |
| De-Leon et al. | 2021 | | What pushed Israel out of herd immunity? Modeling COVID-19 spread of Delta and Waning immunity | | 10.1101/2021.09.12.21263451 |
| Debrabant et al. | 2021 | | The cost-effectiveness of a COVID-19 vaccine in a Danish context | | 10.2139/ssrn.3773381 |
| Demongeot et al. | 2022 | | Modeling vaccine efficacy for COVID-19 outbreak in New York city | | [10.3390/biology11030345](https://doi.org/10.3390/biology11030345) |
| DeWitt | 2021 | | Rapid impact analysis of B 1.1.7 variant on the spread of SARS-CoV-2 in North Carolina | | 10.1101/2021.02.07.21251291 |
| Di Domenico et al. | 2021 | | Impact of January 2021 social distancing measures on SARS-CoV-2 b117 circulation in France | | 10.1101/2021.02.14.21251708 |
| Di Domenico et al. | 2021 | | Adherence and sustainability of interventions informing optimal control against COVID-19 pandemic | | 10.1038/s43856-021-00057-5 |
| Di Fusco et al. | 2022 | | Public health impact of the Pfizer-BioNTech COVID-19 vaccine (BNT162b2) in the first year of rollout in the United States | | 10.1080/13696998.2022.2071427 |
| Diagne et al. | 2021 | | A mathematical model of COVID-19 with vaccination and treatment | | [10.1155/2021/1250129](https://doi.org/10.1155/2021/1250129) |
| Diarra et al. | 2022 | | Non-pharmaceutical interventions and COVID-19 vaccination strategies in Senegal: a modelling study | | 10.1136/ bmjgh-2021-007236 |
| Dick et al. | 2021 | | Fall 2021 resurgence and COVID-19 seroprevalence in Canada: modelling waning and boosting COVID-19 immunity in canada, a canadian immunization research network study | | 10.1101/2021.08.17.21262188 |
| Dimeglio et al. | 2021 | | Estimating the impact of public health strategies on the spread of SARS-CoV-2: epidemiological modelling for Toulouse, France | | [10.1002/rmv.2224](https://doi.org/10.1002/rmv.2224) |
| Dimeglio et al. | 2021 | | Influence of SARS-CoV-2 Variant B.1.1.7, vaccination, and public health measures on the spread of SARS-CoV-2 | | [10.3390/v13050898](https://doi.org/10.3390/v13050898) |
| Dönges et al. | 2022 | | Interplay between risk perception, behavior, and COVID-19 spread | | 10.48550/arXiv.2112.12062 |
| Du et al. | 2022 | | Modeling comparative cost-effectiveness of SARS-CoV-2 vaccine dose fractionation in India | | 10.1038/s41591-022-01736-z |
| Du et al. | 2022 | | Cost-effective proactive testing strategies during COVID-19 mass vaccination: a modelling study | | [10.1016/j.lana.2021.100182](https://doi.org/10.1016/j.lana.2021.100182) |
| Dyson et al. | 2021 | | Possible future waves of SARS-CoV-2 infection generated by variants of concern with a range of characteristics | | 10.1038/s41467-021-25915-7 |
| España et al. | 2021 | | The potential impact of Delta variant of SARS-CoV-2 in the context of limited vaccination coverage and increasing social mixing in Bogota, Colombia | | 10.1101/2021.08.06.21261734 |
| Estadilla et al. | 2021 | | Impact of vaccine supplies and delays on optimal control of the COVID-19 pandemic: mapping interventions for the Philippines | | 10.1186/s40249-021-00886-5 |
| Expósito et al. | 2022 | | Impact of vaccination against COVID-19 in children aged 5-11 years: a mathematical model | | PMID: 35185149 |
| Faranda et al. | 2021 | | Interrupting vaccination policies can greatly spread SARS-CoV-2 and enhance mortality from COVID-19 disease: the AstraZeneca case for France and Italy | | [10.1063/5.0050887](https://doi.org/10.1063/5.0050887) |
| Faucher et al. | 2022 | | Agent-based modelling of reactive vaccination of workplaces and schools against COVID-19 | | 10.1038/s41467-022-29015-y |
| Fawaz et al. | 2021 | | Development of a robust mathematical model to estimate COVID-19 cases in Lebanon based on SEIRDV modified model | | [10.1109/ICABME53305.2021.9604824](https://doi.org/10.1109/ICABME53305.2021.9604824) |
| Feng et al. | 2021 | | Mechanism of optimal time-course COVID-19 vaccine prioritization based on non-markovian steady-state prediction | | 10.1101/2021.10.11.21264836 |
| Feng et al. | 2022 | | Modelling COVID-19 vaccine breakthrough infections in highly vaccinated Israel: the effects of waning immunity and third vaccination dose | | 10.1101/2022.01.08.22268950 |
| Ferranna et al. | 2021 | | COVID-19 vaccine allocation: modeling health outcomes and equity implications of alternative strategies | | [10.1016/j.eng.2021.03.014](https://doi.org/10.1016/j.eng.2021.03.014) |
| Ferreira et al. | 2021 | | Modelling optimal vaccination strategies against COVID-19 in a context of Gamma variant predominance in Brazil | | 10.1101/2021.11.19.21266590 |
| Fierro et al. | 2022 | | Vaccination and variants: retrospective model for the evolution of COVID-19 in Italy | | 10.1101/2022.02.27.22271593 |
| Flores-Arguedas et al. | 2021 | | Estimating the impact of non-pharmaceutical interventions and vaccination on the progress of the COVID-19 epidemic in Mexico: a mathematical approach | | 10.48550/arXiv.2102.11071 |
| Foy et al. | 2021 | | Comparing COVID-19 vaccine allocation strategies in India: a mathematical modelling study | | 10.1016/j.ijid.2020.12.075 |
| Frazier et al. | 2022 | | Modeling for COVID-19 college reopening decisions: Cornell, a case study | | 10.1073/pnas.2112532119 |
| Frieswijk et al. | 2021 | | Modelling the effect of vaccination and human behaviour on the spread of epidemic diseases on temporal networks | | 10.48550/arXiv.2111.05590 |
| Fuady et al. | 2021 | | Targeted vaccine allocation could increase the COVID-19 vaccine benefits amidst Its Lack of availability: a mathematical modeling study in Indonesia | | 10.3390/vaccines9050462 |
| Fujii et al. | 2021 | | COVID-19 and output in Japan | | 10.1007/s42973-021-00098-4 |
| Furuse | 2021 | | Simulation of future COVID-19 epidemic by vaccination coverage scenarios in Japan | | 10.7189/jogh.11.05025 |
| Gabriele-Rivet et al. | 2021 | | Modelling the impact of age-stratified public health measures on SARS-CoV-2 transmission in Canada | | 10.1098/rsos.210834 |
| Galanti et al. | 2021 | | Non-pharmaceutical interventions and inoculation rate shape SARS-CoV-2 vaccination campaign success | | 10.1101/2021.02.22.21252240 |
| Galloway et al. | 2021 | | Emergence of SARS-CoV-2 b117 lineage — United States, December 29, 2020 – January 12, 2021 | | 10.15585/mmwr.mm7003e2 |
| Gandjour | 2022 | | Benefits, risks, and cost-effectiveness of COVID-19 self-tests from a consumer's perspective | | 10.1186/s12913-021-07277-4 |
| Gandjour | 2022 | | Cost-effectiveness of future lockdown policies against the COVID-19 pandemic | | 10.1177/09514848221080687 |
| Ganesan et al. | 2021 | | Ensemble forecast of COVID-19 for vulnerability assessment and policy interventions | | 10.21203/rs.3.rs-931186/v1 |
| García et al. | 2022 | | Projecting the impact of COVID-19 variants and vaccination strategies in disease transmission using a multilayer network model in Costa Rica | | 10.1038/s41598-022-06236-1 |
| Gavish et al. | 2022 | | Optimal vaccination at high reproductive numbers: sharp transitions and counter-intuitive allocations | | 10.48550/arXiv.2202.03909 |
| Gavish et al. | 2022 | | The role of childrens vaccination for COVID-19: Pareto-optimal allocations of vaccines | | 10.1371/journal.pcbi.1009872 |
| Genari et al. | 2022 | | Quantifying protocols for safe school activities | | 10.48550/arXiv.2204.07148 |
| Ghafari et al. | 2022 | | A framework for reconstructing SARS-CoV-2 transmission dynamics using excess mortality data | | 10.1101/2021.10.04.21264540 |
| Ghosh et al. | 2020 | | COVID-19: risks of re-emergence, re-infection, and control measures: a long term modelling study | | 10.1101/2020.09.19.20198051 |
| Giacopelli | 2020 | | A full-scale agent-based model of lombardy COVID-19 dynamics to explore social networks connectivity and vaccine impact on epidemic | | 10.1101/2020.09.13.20193599 |
| Di Giamberardino et al. | 2021 | | Optimal exit strategy design for COVID-19 | | [10.1109/ICSTCC52150.2021.9607220](https://doi.org/10.1109/ICSTCC52150.2021.9607220) |
| Giardina et al. | 2022 | | Model-estimated association between simulated US elementary school-related SARS-CoV-2 transmission, mitigation interventions, and vaccine coverage across local incidence levels | | 10.1001/jamanetworkopen.2021.47827 |
| Glover et al | 2020 | | Health versus wealth: on the distributional effects of controlling a pandemic | | 10.3386/w27046 |
| Glover et al. | 2021 | | Optimal age-based vaccination and economic mitigation policies for the second phase of the COVID-19 | | 10.18651/RWP2021-15 |
| Goldstein et al. | 2021 | | Vaccinating the oldest against COVID-19 saves both the most lives and most years of life | | [10.1073/pnas.2026322118](https://doi.org/10.1073/pnas.2026322118) |
| Gomes et al. | 2022 | | Individual variation in susceptibility or exposure to SARS-CoV-2 lowers the herd immunity threshold | | 10.1016/j.jtbi.2022.111063 |
| González-Parra et al. | 2022 | | Mathematical modeling to study optimal allocation of vaccines against COVID-19 using an age-structured population | | 10.3390/axioms11030109 |
| Gonzalez-Parra | 2021 | | Analysis of delayed vaccination regimens: a mathematical modeling approach | | 10.3390/epidemiologia2030021 |
| Good et al. | 2020 | | The interaction of natural and vaccine-induced immunity with social distancing predicts the evolution of the COVID-19 Pandemic | | 10.1128/mBio.02617-20 |
| Gozzi et al. | 2022 | | Preliminary modeling estimates of the relative transmissibility and immune escape of the Omicron SARS-CoV-2 variant of concern in South Africa | | 10.1101/2022.01.04.22268721 |
| Gozzi et al. | 2021 | | The importance of non-pharmaceutical interventions during the COVID-19 vaccine rollout | | 10.1371/journal.pcbi.1009346 |
| Guerstein et al. | 2020 | | Optimal strategies for combining vaccine prioritization and social distancing to reduce hospitalizations and mitigate COVID19 progression | | 10.1101/2020.12.22.20248622 |
| Gumel et al. | 2021 | | Toward achieving a vaccine-derived herd immunity threshold for COVID-19 in the U.S. | | 10.3389/fpubh.2021.709369 |
| Gutiérrez-Jara et al. | 2022 | | Risk perception influence on vaccination program on COVID-19 in Chile: a mathematical model | | 10.3390/ijerph19042022 |
| Guzmán-Merino et al. | 2021 | | Assessing population-sampling strategies for reducing the COVID-19 incidence | | 10.1016/j.compbiomed.2021.104938 |
| Hagens et al. | 2021 | | COVID-19 vaccination scenarios: a cost-effectiveness analysis for Turkey | | [10.3390/vaccines9040399](https://dx.doi.org/10.3390%2Fvaccines9040399) |
| Hammoumi et al. | 2021 | | Impact of booster COVID-19 vaccine for Moroccan adults: a discrete age-structured model approach | | 10.1101/2021.03.14.21253555 |
| Han et al. | 2021 | | Time-varying optimization of COVID-19 vaccine prioritization in the context of limited vaccination capacity | | 10.1038/s41467-021-24872-5 |
| Hanly et al. | 2022 | | The impact of re-opening the international border on COVID-19 hospitalisations in Australia: a modelling study | | 10.5694/mja2.51291 |
| Hartnett et al. | 2021 | | Modelling the impact of social distancing and targeted vaccination on the spread of COVID-19 through a real city-scale contact network | | [10.1093/comnet/cnab042](https://doi.org/10.1093/comnet/cnab042) |
| Hawkes et al. | 2022 | | Vaccinating children against COVID-19: commentary and mathematical modelling | | 10.1101/2022.01.05.22268820 |
| Hinch et al. | 2021 | | OpenABM-Covid19-an agent-based model for non-pharmaceutical interventions against COVID-19 including contact tracing | | 10.1371/journal.pcbi.1009146 |
| Hjorleifsson et al. | 2022 | | Reconstruction of a large-scale outbreak of SARS-CoV-2 infection in Iceland informs vaccination strategies | | 10.1016/j.cmi.2022.02.012 |
| Hladish et al. | 2022 | | Updated projections for COVID-19 Omicron wave in Florida | | 10.1101/2022.01.06.22268849 |
| Hoertel et al. | 2021 | | Optimizing SARS-CoV-2 vaccination strategies in France: results from a stochastic agent-based model | | 10.21203/rs.3.rs-149457/v1 |
| Hogan et al. | 2021 | | Within-country age-based rioritization, global allocation, and public health impact of a vaccine against SARS-CoV-2: a mathematical modelling analysis | | 10.1016/j.vaccine.2021.04.002 |
| Hohenegger et al. | 2021 | | Effective mathematical modelling of health passes during a pandemic | | 10.21203/rs.3.rs-1031016/v1 |
| Holmdahl et al. | 2021 | | Modeling the impact of vaccination strategies for nursing homes in the context of increased SARS-CoV-2 community transmission and variants | | 10.1101/2021.10.25.21265493 |
| Huang et al. | 2021 | | Integrated vaccination and physical distancing interventions to prevent future COVID-19 waves in Chinese cities | | 10.1038/s41562-021-01063-2 |
| Hupert et al. | 2022 | | Heterologous vaccination interventions to reduce pandemic morbidity and mortality: modeling the US winter 2020 COVID-19 wave | | 10.1073/pnas.2025448119 |
| Iboi et al. | 2020 | | Will an imperfect vaccine curtail the COVID-19 pandemic in the U.S.? | | 10.1016/j.idm.2020.07.006 |
| Jablonska et al. | 2021 | | The real-life impact of vaccination on COVID-19 mortality in Europe and Israel | | 10.1016/j.puhe.2021.07.037 |
| Jayasundara et al. | 2021 | | Sustaining effective COVID-19 control in Malaysia through large-scale vaccination | | 10.1016/j.epidem.2021.100517 |
| Jentsch et al. | 2021 | | Prioritising COVID-19 vaccination in changing social and epidemiological landscapes | | 10.1016/S1473-3099(21)00057-8 |
| Jiménez-Rodríguez et al. | 2021 | | A population structure-sensitive mathematical model assessing the effects of vaccination during the third surge of COVID-19 in Italy | | 10.1016/j.jmaa.2021.125975 |
| Kahn et al. | 2022 | | Mathematical modeling to inform vaccination strategies and testing approaches for Coronavirus disease 2019 (COVID-19) in nursing homes | | 10.1093/cid/ciab517 |
| Karabay et al. | 2021 | | COVID-19 vaccination strategies considering hesitancy using particle-based epidemic simulation | | [10.1109/EMBC46164.2021.9630797](https://doi.org/10.1109/EMBC46164.2021.9630797) |
| Karabay et al. | 2021 | | A vaccination simulator for COVID-19: effective and sterilizing immunization cases | | 10.1109/JBHI.2021.3114180 |
| Kassa et al. | 2021 | | Modelling COVID-19 mitigation and control strategies in the presence of migration and vaccination: the case of South Africa | | 10.1007/s13370-021-00900-x |
| Keeling et al. | 2022 | | Fitting to the UK COVID-19 outbreak, short-term forecasts and estimating the reproductive number | | 10.1177/09622802211070257 |
| Keeling et al. | 2021 | | Short-term projections based on early omicron variant dynamics in England | | 10.1101/2021.12.30.21268307 |
| Kemp et al. | 2021 | | Modelling COVID-19 dynamics and potential for herd immunity by vaccination in Austria, Luxembourg and Sweden | | [10.1016/j.jtbi.2021.110874](https://doi.org/10.1016/j.jtbi.2021.110874) |
| Kerr et al. | 2021 | | Covasim: an agent-based model of COVID-19 dynamics and interventions | | 10.1371/journal.pcbi.1009149 |
| Khan et al. | 2021 | | Modeling the dynamics of the SARS-CoV-2 virus in a population with asymptomatic and symptomatic infected individuals and vaccination | | 10.1088/1402-4896/ac0e00 |
| Kim et al. | 2021 | | Resource allocation for different types of vaccines against COVID-19: tradeoffs and synergies between efficacy and reach | | 10.1016/j.vaccine.2021.10.025 |
| Kirwin et al. | 2021 | | A net benefit approach for the optimal allocation of a COVID-19 vaccine | | 10.1007/s40273-021-01037-2 |
| Kitano et al. | 2021 | | The incremental burden of invasive pneumococcal disease associated with a decline in childhood vaccination using a dynamic transmission model in Japan: a secondary impact of COVID-19 | | 10.1016/j.compbiomed.2021.104429 |
| Ko et al. | 2022 | | Multi-faceted analysis of COVID-19 epidemic in the Republic of Korea considering Omicron variant: mathematical modeling-based study | | 10.1101/2022.04.15.22273907 |
| Ko et al. | 2021 | | Risk of COVID-19 transmission in heterogeneous age groups and effective vaccination strategy in Korea: a mathematical modeling study | | 10.4178/epih.e2021059 |
| Kou et al. | 2021 | | A multi-scale agent-based model of infectious disease transmission to assess the impact of vaccination and non-pharmaceutical interventions: the COVID-19 case | | 10.1016/j.jnlssr.2021.08.005 |
| Kraay et al. | 2021 | | Modeling the use of SARS-CoV-2 vaccination to safely relax non-pharmaceutical interventions | | 10.1101/2021.03.12.21253481 |
| Lasser et al. | 2022 | | Assessing the impact of SARS-CoV-2 prevention measures in Austrian schools using agent-based simulations and cluster tracing data | | 10.1038/s41467-022-28170-6 |
| Lasser et al. | 2022 | | Assessment of the effectiveness of Omicron transmission mitigation strategies for European universities using an agent-based network model | | 10.48550/arXiv.2201.08850 |
| Latkowski et al. | 2021 | | An agent-based COVID-19 simulator: extending Covasim to the polish context | | 10.1016/j.procs.2021.09.134 |
| Lau et al. | 2021 | | Risk-benefit analysis of the AstraZeneca COVID-19 vaccine in Australia using a rioriti network modelling framework | | 10.1016/j.vaccine.2021.10.079 |
| Layton et al. | 2022 | | Understanding the dynamics of SARS-CoV-2 variants of concern in Ontario, Canada: a modeling study | | 10.1038/s41598-022-06159-x |
| Lee et al. | 2021 | | Strategies for vaccine prioritization and mass dispensing | | 10.3390/vaccines9050506 |
| Lemaitre et al. | 2021 | | Optimizing the spatio-temporal allocation of COVID-19 vaccines: Italy as a case study | | 10.1101/2021.05.06.21256732 |
| León et al. | 2021 | | Nonpharmaceutical interventions remain essential to reducing coronavirus disease 2019 burden even in a well-vaccinated society: a modeling study | | 10.1093/ofid/ofab415 |
| Leung et al. | 2021 | | Effects of adjusting public health, travel, and social measures during the roll-out of COVID-19 vaccination: a modelling study | | 10.1016/S2468-2667(21)00167-5 |
| Li et al. | 2021 | | Toward the impact of non-pharmaceutical interventions and vaccination on the COVID-19 pandemic with time-dependent SEIR model | | 10.3389/frai.2021.648579 |
| Li et al. | 2022 | | COVID-19 epidemic in New York City: development of an age group-specific mathematical model to predict the outcome of various vaccination strategies | | 10.1186/s12985-022-01771-9 |
| Li et al. | 2021 | | Evaluating the impact of SARS-CoV-2 variants on the COVID-19 epidemic and social restoration in the United States: a mathematical modelling study | | 10.3389/fpubh.2021.801763 |
| Li et al. | 2021 | | Feasibility of booster vaccination in high-risk populations for controlling coronavirus variants — China, 2021 | | 10.46234/ccdcw2021.259 |
| Li et al. | 2021 | | Impact of non-pharmacological interventions on COVID-19 boosting vaccine prioritization and vaccine-induced herd immunity: a population-stratified modelling study | | 10.1101/2021.10.27.21265522 |
| Li et al. | 2021 | | Prioritizing vaccination by age and social activity to advance societal health benefits in Norway: a modelling study | | 10.1016/j.lanepe.2021.100200 |
| Li et al. | 2021 | | Returning to a normal life via COVID-19 vaccines in the United States: a large-scale agent-based simulation study | | 10.2196/27419 |
| Li et al. | 2021 | | Transmission dynamics, heterogeneity and controllability of SARS-CoV-2: a rural-urban comparison | | 10.3390/ijerph18105221 |
| Lin et al. | 2022 | | Multiple COVID-19 waves and vaccination effectiveness in the United States | | 10.3390/ijerph19042282 |
| Lin et al. | 2022 | | Two waves of coivd-19 in brazilian cities and vaccination impact | | 10.3934/mbe.2022216 |
| Liu et al. | 2022 | | Dosing interval strategies for two-dose COVID-19 vaccination in 13 middle-income countries of europe: health impact modelling and benefit-risk analysis | | 10.1016/j.lanepe.2022.100381 |
| Liu et al. | 2022 | | Investigating vaccine-induced immunity and its effect in mitigating SARS-CoV-2 epidemics in China | | 10.1186/s12916-022-02243-1 |
| Liu et al. | 2022 | | Return to normal pre-COVID-19 life is delayed by inequitable vaccine allocation and SARS-CoV-2 variants | | 10.1017/S0950268822000139 |
| Luo et al. | 2021 | | Evaluating the impact of vaccination on COVID-19 pandemic used a hierarchical weighted contact network model | | [10.1109/EMBC46164.2021.9630797](https://doi.org/10.1109/EMBC46164.2021.9630797) |
| Machado et al. | 2022 | | The impact of vaccination on the evolution of COVID-19 in Portugal | | 10.3934/mbe.2022043 |
| Mahmoud et al. | 2021 | | The impact of COVID-19 vaccination campaigns accounting for antibody-dependent enhancement | | 10.1371/journal.pone.0245417 |
| Maier et al. | 2021 | | Potential benefits of delaying the second mRNA COVID-19 vaccine dose | | 10.48550/arXiv.2102.13600 |
| Mairanowski et al. | 2021 | | Functional dependence of COVID-19 growth rate on lockdown conditions and rate of vaccination | | 10.1101/2021.06.06.21258425 |
| Mairanowski et al. | 2021 | | The age-stratified analytical model for the spread of the COVID-19 epidemic | | 10.1101/2021.07.13.21260459 |
| Majumder et al. | 2022 | | COVID-19 disease dynamics with vaccination: the effect of uncertainty | | 10.1101/2022.01.10.22269006 |
| Makhoul et al. | 2021 | | Epidemiological differences in the impact of COVID-19 vaccination in the United States and China | | 10.3390/vaccines9030223 |
| Makhoul et al. | 2020 | | Epidemiological impact of SARS-CoV-2 vaccination: mathematical modeling analyses | | 10.3390/vaccines8040668 |
| Mallela et al. | 2022 | | Bayesian inference of state-level COVID-19 basic reproduction numbers across the United States | | [10.3390/v14010157](https://doi.org/10.3390/v14010157) |
| Mandal et al. | 2021 | | India’s pragmatic vaccination strategy against COVID-19: a mathematical modelling-based analysis | | 10.1136/bmjopen-2021-048874 |
| Mandal et al. | 2021 | | Plausibility of a third wave of COVID-19 in India: a mathematical modelling based analysis | | 10.4103/ijmr.ijmr_1627_21 |
| Marín-Hernández et al. | 2021 | | Anticipated reduction in COVID-19 mortality due to population-wide BCG vaccination: evidence from Germany | | 10.1080/21645515.2021.1872344 |
| Martínez-Rodríguez et al. | 2021 | | Analysis of key factors of a SARS-CoV-2 vaccination program: a mathematical modeling approach | | 10.3390/epidemiologia2020012 |
| Marziano et al. | 2021 | | The effect of COVID-19 vaccination in Italy and perspectives for living with the virus | | 10.1038/s41467-021-27532-w |
| Massonnaud et al. | 2021 | | Evaluating COVID-19 booster vaccination strategies in a partially vaccinated population: a modeling study | | 10.1101/2021.12.01.21267122 |
| Mathiot et al. | 2021 | | Highlighting the impact of social relationships on the propagation of respiratory viruses using percolation theory | | 10.1038/s41598-021-03812-9 |
| Matrajt et al. | 2021 | | Optimizing vaccine allocation for COVID-19 vaccines shows the potential role of single-dose vaccination | | 10.1101/2020.12.31.20249099 |
| Matrajt et al. | 2021 | | Quantifying the impact of lifting community nonpharmaceutical interventions for COVID-19 during vaccination rollout in the United States | | 10.1093/ofid/ofab341 |
| Matrajt et al. | 2020 | | Vaccine optimization for COVID-19: Who to vaccinate first? | | 10.1101/2020.08.14.20175257 |
| Matrajt et al. | 2022 | | Could widespread use of antiviral treatment curb the COVID-19 pandemic? A modeling study | | 10.1101/2021.11.10.21266139 |
| McBryde et al. | 2021 | | Modelling direct and herd protection effects of vaccination against the SARS-CoV-2 Delta variant in Australia | | 10.5694/mja2.51263 |
| Michael et al. | 2021 | | Projections and management of the COVID-19 emergency in India | | 10.21203/rs.3.rs-575258/v1 |
| Milne et al. | 2022 | | Mitigating the SARS-CoV-2 Delta disease burden in Australia by non-pharmaceutical interventions and vaccinating children: a modelling analysis | | 10.1186/s12916-022-02241-3 |
| Min et al. | 2021 | | Dynamics of the COVID-19 epidemic in the post-vaccination period in Korea: a rapid assessment | | 10.4178/epih.e2021040 |
| Miura et al. | 2021 | | Optimal vaccine allocation for COVID-19 in the Netherlands: a data-driven prioritization | | 10.1371/journal.pcbi.1009697 |
| Moghadas et al. | 2021 | | Evaluation of COVID-19 vaccination strategies with a delayed second dose | | 10.1371/journal.pbio.3001211 |
| Moghadas et al. | 2021 | | Simulated identification of silent COVID-19 infections among children and estimated future infection rates with vaccination | | 10.1101/2021.01.06.21249349 |
| Moghadas et al. | 2021 | | The Impact of vaccination on Coronavirus disease 2019 (COVID-19) outbreaks in the United States | | 10.1093/cid/ciab079 |
| Moghadas et al. | 2021 | | Can the USA return to pre-COVID-19 normal by July 4? | | 10.1016/S1473-3099(21)00324-8 |
| Moldokmatova et al. | 2022 | | Mathematical modelling of COVID-19 vaccination strategies in Kyrgyzstan | | 10.1101/2021.12.21.21268200 |
| Moore et al. | 2021 | | Modelling optimal vaccination strategy for SARS-CoV-2 in the UK | | 10.1371/journal.pcbi.1008849 |
| Moore et al. | 2021 | | Vaccination and non-pharmaceutical interventions for COVID-19: a mathematical modelling study | | 10.1016/S1473-3099(21)00143-2 |
| Morales-Zamora et al. | 2022 | | Cost-effectiveness analysis of strategies of COVID-19 vaccination in colombia: comparison of high-risk prioritization and no prioritization strategies with the absence of a vaccination plan | | [https://10.1016/j.vhri.2022.04.004](https://doi.org/10.1016/j.vhri.2022.04.004) |
| Mukandavire et al. | 2020 | | Quantifying early COVID-19 outbreak transmission in South Africa and exploring vaccine efficacy scenarios | | 10.1371/journal.pone.0236003 |
| Mumtaz et al. | 2021 | | Modeling the impact of COVID-19 vaccination in Lebanon: a call to speed-up vaccine roll out | | 10.3390/vaccines9070697 |
| Musa et al. | 2020 | | Assessment of the impacts of pharmaceutical and non-pharmaceutical intervention on COVID-19 in South Africa using mathematical model | | 10.1101/2020.11.13.20231159 |
| Nakhaeizadeh et al. | 2022 | | Estimating the number of COVID-19 cases and impact of new COVID-19 variants and vaccination on the population in Kerman, Iran: a mathematical modeling study | | 10.1155/2022/6624471 |
| Nam et al. | 2021 | | Modelling the impact of extending dose intervals for COVID-19 vaccines in Canada | | 10.1101/2021.04.07.21255094 |
| Nichita et al. | 2021 | | Modeling COVID-19 transmission using idsim, an epidemiological-modelling desktop app with multi-level immunization capabilities | | 10.48550/arXiv.2112.15252 |
| Nixon et al. | 2021 | | Impacts of vaccination and asymptomatic testing on SARS-CoV-2 transmission dynamics in a university setting | | 10.1101/2021.11.22.21266565 |
| Nuraini et al. | 2021 | | Mathematical models for assessing vaccination scenarios in several provinces in Indonesia | | 10.1016/j.idm.2021.09.002 |
| Olmedo et al. | 2022 | | Predictions of three mathematical models related with the COVID-19 vaccination Strategy in Spain. June 2021 | | PMID: 35179148 |
| Oloniiju et al. | 2022 | | Investigating the impact of vaccination and non-pharmaceutical measures in curbing COVID-19 spread: a South Africa perspective | | 10.3934/mbe.2022049 |
| Omae et al. | 2022 | | SIRVVD model-based verification of the effect of first and second doses of COVID-19/SARS-CoV-2 vaccination in Japan | | 10.3934/mbe.2022047 |
| Omae et al. | 2022 | | Theoretical analysis of the SIRVVD model for insights into the target rate of COVID-19/SARS-CoV-2 vaccination in Japan | | [10.1109/ACCESS.2022.3168985](https://doi.org/10.1109/ACCESS.2022.3168985) |
| Omar et al. | 2021 | | COVID-19 deterministic and stochastic modelling with optimized daily vaccinations in Saudi Arabia | | 10.1016/j.rinp.2021.104629 |
| Omar et al. | 2022 | | Fractional stochastic modelling of COVID-19 under wide spread of vaccinations: Egyptian case study | | 10.1016/j.aej.2022.02.002 |
| Padula et al. | 2021 | | Economic value of vaccines to address the COVID-19 pandemic: a U.S. cost-effectiveness and budget impact analysis | | 10.1080/13696998.2021.1965732 |
| Pae | 2021 | | Calibrating an SIR model for South Korea COVID-19 infections and predicting vaccination impact | | 10.1101/2021.09.27.21264172 |
| Pageaud et al. | 2021 | | Adapting french COVID-19 vaccination campaign duration to variant dissemination | | 10.1101/2021.03.17.21253739 |
| Paltiel et al. | 2021 | | Assessing COVID-19 prevention strategies to permit the safe opening of residential colleges in fall 2021 | | 10.7326/M21-2965 |
| Pan et al. | 2022 | | Vaccination as an alternative to non-drug interventions to prevent local resurgence of COVID-19 | | [https://10.1186/s40249-022-00960-6](https://doi.org/10.1186/s40249-022-00960-6) |
| Parino et al. | 2021 | | A model predictive control approach to optimally devise a two‐dose vaccination rollout: a case study on COVID‐19 in Italy | | 10.1002/rnc.5728 |
| Parolini et al. | 2022 | | Modelling the COVID-19 epidemic and the vaccination campaign in Italy by the SUIHTER model | | 10.1016/j.idm.2022.03.002 |
| Patel et al. | 2021 | | Association of simulated COVID-19 vaccination and nonpharmaceutical interventions with infections, hospitalizations, and mortality | | 10.1001/jamanetworkopen.2021.10782 |
| Pearson et al. | 2021 | | COVID-19 vaccination in Sindh Province, Pakistan: a modelling study of health impact and cost-effectiveness | | 10.1371/journal.pmed.1003815 |
| Pérez et al. | 2021 | | An extended SEIARD model for COVID-19 vaccination in Mexico: analysis and forecast | | 10.1101/2021.04.06.21255039 |
| Rabiu et al. | 2022 | Assessing the potential impact of immunity waning on the dynamics of COVID-19 in South Africa: an endemic model of COVID-19 | | 10.1007/s11071-022-07225-9 | |
| Rachaniotis et al. | 2021 | | A two-phase stochastic dynamic model for COVID-19 mid-term policy recommendations in Greece: a pathway towards mass vaccination | | 10.3390/ijerph18052497 |
| Rahmandad et al. | 2021 | | Behavioral dynamics of COVID-19: estimating under-reporting, multiple waves, and adherence fatigue across 92 nations | | 10.1002/sdr.1673 |
| Raina et al. | 2021 | | The use of face masks during vaccine roll-out in New York City and impact on epidemic control | | 10.1016/j.vaccine.2021.08.102 |
| Rajakaruna et al. | 2022 | | Dynamical regulations on mobility and vaccinations for controlling COVID-19 spread | | 10.1038/s41598-022-07371-5 |
| Rajapaksha et al. | 2021 | | An extended Susceptible-Exposed-Infected-Recovered (SEIR) model with vaccination for predicting the COVID-19 pandemic in Sri Lanka | | 10.1101/2021.06.17.21258837 |
| Rajput et al. | 2021 | | Optimal control strategies on COVID-19 infection to bolster the efficacy of vaccination in India | | 10.1038/s41598-021-99088-0 |
| Rana et al. | 2022 | | The modeling and analysis of the COVID-19 pandemic with vaccination and treatment control: a case study of Maharashtra, Delhi, Uttarakhand, Sikkim, and Russia in the light of pharmaceutical and non-pharmaceutical approaches | | [https://10.1140/epjs/s11734-022-00534-5](https://doi.org/10.1140/epjs/s11734-022-00534-5) |
| Rao et al. | 2021 | | Optimal allocation of limited vaccine to control an infectious disease: simple analytical conditions | | 10.1016/j.mbs.2021.108621 |
| Reddy et al. | 2021 | | Clinical outcomes and cost-effectiveness of COVID-19 vaccination in South Africa | | 10.1038/s41467-021-26557-5 |
| Reyné et al. | 2022 | | Non-markovian modelling highlights the importance of age structure on COVID-19 epidemiological dynamics | | 10.1051/mmnp/2022008 |
| Robles-Fontán et al. | 2022 | | Effectiveness estimates of three COVID-19 vaccines based on observational data from Puerto Rico | | 10.1016/j.lana.2022.100212 |
| Rocha et al. | 2021 | | A data-driven model for COVID-19 pandemic: evolution of the attack rate and prognosis for Brazil | | 10.1016/j.chaos.2021.111359 |
| Rodríguez et al. | 2021 | | COVID-19 vaccination rate and protection attitudes can determine the best prioritisation strategy to reduce fatalities | | 10.1101/2020.10.12.20211094 |
| Romero-Brufau et al. | 2021 | | The public health impact of delaying a second dose of the BNT-162b2 or MRNA-1273 COVID-19 vaccine | | [https://10.1101/2021.02.23.21252299](https://doi.org/10.1101/2021.02.23.21252299) |
| Roy et al. | 2021 | | Modeling of COVID-19 transmission dynamics on US population: inter-transfer infection in age groups, mutant variants, and vaccination strategies | | 10.1101/2021.09.25.21264118 |
| Roy et al. | 2021 | | The efficacy of vaccines in the context of COVID-19 and its variants: role of spatio-temporal boundary | | 10.1101/2021.07.19.21260758 |
| Sadarangani et al. | 2021 | | Importance of COVID-19 vaccine efficacy in older age groups | | [10.1016/j.vaccine.2021.03.020](https://doi.org/10.1016/j.vaccine.2021.03.020) |
| Sah et al. | 2021 | | Accelerated vaccine rollout is imperative to mitigate highly transmissible COVID-19 variants | | 10.1016/j.eclinm.2021.100865 |
| Saldaña et al. | 2022 | | Influence of heterogeneous age-group contact patterns on critical vaccination rates for herd immunity to SARS-CoV-2 | | 10.1038/s41598-022-06477-0 |
| Sandmann et al. | 2021 | | The potential health and economic value of SARS-CoV-2 vaccination alongside physical distancing in the UK: a transmission model-based future scenario analysis and economic evaluation | | 10.1016/S1473-3099(21)00079-7 |
| Sanz-Leon et al. | 2022 | | Risk of sustained SARS-CoV-2 transmission in Queensland, Australia | | [https://10.1038/s41598-022-10349-y](https://doi.org/10.1038/s41598-022-10349-y) |
| Savinkina et al. | 2022 | | Model-based estimates of deaths averted and cost per life saved by scaling-up mRNA COVID-19 vaccination in low and lower-middle income countries in the COVID-19 Omicron variant era | | 10.1101/2022.02.08.22270465 |
| Scarabaggio et al. | 2021 | | Modeling, estimation, and optimal control of anti-COVID-19 multi-dose vaccine administration | | 10.1109/CASE49439.2021.9551418 |
| Schneider et al. | 2022 | | Predicting the impact of COVID-19 vaccination campaigns – a flexible age-dependent, spatially-stratified predictive model, accounting for multiple viral variants and vaccines | | 10.1101/2021.12.29.21268526 |
| Schulenburg et al. | 2022 | | Effects of infection fatality ratio and social contact matrices on vaccine prioritization strategies | | 10.48550/arXiv.2201.02869 |
| Shadi et al. | 2022 | | Mathematical modeling of the novel Coronavirus pandemic in Iran: a model with vaccination | | [10.1109/ICCIA54998.2022.9737192](https://doi.org/10.1109/ICCIA54998.2022.9737192) |
| Shen et al. | 2021 | | Projected COVID-19 epidemic in the United States in the context of the effectiveness of a potential vaccine and implications for social distancing and face mask use | | 10.1016/j.vaccine.2021.02.056 |
| Shim | 2021 | | Optimal allocation of the limited COVID-19 vaccine supply in South Korea | | 10.3390/jcm10040591 |
| Shim | 2021 | | Projecting the impact of SARS-CoV-2 Variants and the vaccination program on the fourth wave of the COVID-19 pandemic in South Korea | | 10.3390/ijerph18147578 |
| Silva et al. | 2021 | | Optimized delay of the second COVID-19 vaccine dose reduces ICU admissions | | 10.1073/pnas.2104640118 |
| Castro e Silva et al. | 2022 | | Successive pandemic waves with different virulent strains and the effects of vaccination for SARS-CoV-2 | | 10.3390/vaccines10030343 |
| Sivadas et al. | 2021 | | Control strategies for the third wave of COVID-19 infection in India: a mathematical model incorporating vaccine effectiveness | | 10.1101/2021.12.27.21268464 |
| Somekh et al. | 2022 | | Quantifying the population-level effect of the COVID-19 mass vaccination campaign in Israel: a modeling study | | 10.1093/ofid/ofac087 |
| Sonabend et al. | 2021 | | Non-pharmaceutical interventions, vaccination, and the SARS-CoV-2 delta variant in England: a mathematical modelling study | | 10.1016/S0140-6736(21)02276-5 |
| Song et al. | 2021 | | The second wave of COVID-19 in South and Southeast Asia and the effects of vaccination | | 10.3389/fmed.2021.773110 |
| Song et al. | 2021 | | Vaccination against COVID-19 and society’s return to normality in England: a modelling study of impacts of different types of naturally acquired and vaccine-induced immunity | | 10.1136/bmjopen-2021-053507 |
| Souto et al. | 2022 | | Assessing the best time interval between doses in a two-dose vaccination regimen to reduce the number of deaths in an ongoing epidemic of SARS-CoV-2 | | 10.1371/journal.pcbi.1009978 M |
| Stanojevic et al. | 2021 | | Simulation and prediction of spread of COVID-19 in The Republic of Serbia by SEAIHRDS model of disease transmission | | 10.1016/j.mran.2021.100161 |
| Stapelberg et al. | 2021 | | A Discrete-Event, Simulated Social Agent-Based Network Transmission (DESSABNeT) model for communicable diseases: method and validation using SARS-CoV-2 data in three large Australian cities | | 10.1371/journal.pone.0251737 |
| Stevenson et al. | 2021 | | Modelling of hypothetical SARS-CoV-2 point of care tests for routine testing in residential care homes: rapid cost-effectiveness analysis | | [https://10.3310/hta25390](https://doi.org/10.3310/hta25390) |
| Steyn et al. | 2022 | | A COVID-19 vaccination model for Aotearoa New Zealand | | 10.1038/s41598-022-06707-5 |
| Stollenwerk et al. | 2021 | | Modeling COVID-19 vaccine efficacy and coverage towards herd-immunity in the Basque Country, Spain | | [10.1101/2021.07.12.21260390](https://doi.org/10.1101/2021.07.12.21260390) |
| Storlie et al. | 2021 | | Quantifying the importance of COVID-19 vaccination to our future outlook | | 10.1016/j.mayocp.2021.04.012 |
| Sulis et al. | 2021 | | An agent-based decision support for a vaccination campaign | | 10.1007/s10916-021-01772-1 |
| Sun et al. | 2021 | | Analysis on action tracking reports of COVID-19 informs control strategies and vaccine delivery in post-pandemic era | | 10.1101/2021.04.08.21254953 |
| Sunohara et al. | 2021 | | Effective vaccine allocation strategies, balancing economy with infection control against COVID-19 in Japan | | 10.1371/journal.pone.0257107 |
| Suphanchaimat et al. | 2022 | | Forecasted trends of the new COVID-19 epidemic due to the Omicron variant in Thailand, 2022 | | 10.1101/2022.01.24.477479 |
| Suphanchaimat et al. | 2021 | | Predicted impact of vaccination and active case finding measures to control epidemic of Coronavirus disease 2019 in a migrant-populated area in Thailand | | [10.2147/rmhp.s318012](https://doi.org/10.2147/rmhp.s318012) |
| Suphanchaimat et al. | 2021 | | Prioritization of the target population for Coronavirus disease 2019 (COVID-19) vaccination program in Thailand | | 10.3390/ijerph182010803 |
| Swan et al. | 2021 | | COVID-19 vaccines that reduce symptoms but do not block infection need higher coverage and faster rollout to achieve population impact | | 10.1038/s41598-021-94719-y |
| Swan et al. | 2021 | | Mathematical modeling of vaccines that prevent SARS-CoV-2 transmission | | 10.3390/v13101921 |
| Swan et al. | 2020 | | Vaccines that prevent SARS-CoV-2 transmission may prevent or dampen a Spring wave of COVID-19 cases and deaths in 2021 | | 10.1101/2020.12.13.20248120 |
| Tan et al. | 2022 | | COVID-19 vaccination and estimated public health impact in California | | 10.1001/jamanetworkopen.2022.8526 |
| Tang et al. | 2021 | | The minimal COVID-19 vaccination coverage and efficacy to compensate for potential increase of transmission contacts, and increased transmission probability of the emerging strains | | 10.21203/rs.3.rs-140717/v1 |
| Tang et al. | 2021 | | A new prediction method of COVID-19 epidemic | | [10.1109/PRAI53619.2021.9551052](https://doi.org/10.1109/PRAI53619.2021.9551052) |
| Tang et al. | 2021 | | Controlling multiple COVID-19 epidemic waves: an insight from a multi-scale model linking the behavior change dynamics to the disease transmission dynamics | | 10.1101/2021.04.07.21255056 |
| Tatapudi et al. | 2021 | | Impact of vaccine prioritization strategies on mitigating COVID-19: an agent-based simulation study using an urban region in the United States | | 10.1186/s12874-021-01458-9 |
| Teslya et al. | 2021 | | The importance of sustained compliance with physical distancing during COVID-19 vaccination rollout | | 10.1101/2021.09.22.21263944 |
| Tetteh et al. | 2021 | | Network models to evaluate vaccine strategies towards herd immunity in COVID-19 | | 10.1016/j.jtbi.2021.110894 |
| Thompson et al. | 2021 | | Estimating the impact of interventions against COVID-19: from lockdown to vaccination | | 10.1371/journal.pone.0261330 |
| Tokuda et al. | 2021 | | Potential impact of alternative vaccination strategies on COVID‐19 cases, hospitalization, and mortality in Japan during 2021–2022 | | 10.1002/jgf2.493 |
| Tonkens et al. | 2022 | | Optimizing vaccine allocation strategies in pandemic outbreaks: an optimal control approach | | [10.48550/arXiv.2112.11908](https://doi.org/10.48550/arXiv.2112.11908) |
| Topîrceanu | 2021 | | Immunization using a heterogeneous geo-spatial population model: a qualitative perspective on COVID-19 vaccination strategies | | [10.1016/j.procs.2021.08.217](https://doi.org/10.1016/j.procs.2021.08.217) |
| Torku et al. | 2021 | | Deep-data-driven neural networks for COVID-19 vaccine efficacy | | 10.3390/epidemiologia2040039 |
| Tran et al. | 2021 | | Optimal SARS-CoV-2 vaccine allocation using real-time attack-rate estimates in Rhode Island and Massachusetts | | 10.1186/s12916-021-02038-w |
| Truszkowska et al. | 2021 | | Designing the safe reopening of US towns through high‐resolution agent‐based modeling | | [10.1002/adts.202100157](https://doi.org/10.1002/adts.202100157) |
| Truszkowska et al. | 2022 | | Predicting the effects of waning vaccine immunity against COVID‐19 through high‐resolution agent‐based modeling | | [10.1002/adts.202100521](https://doi.org/10.1002/adts.202100521) |
| Usherwood et al. | 2021 | | A model and predictions for COVID-19 considering population behavior and vaccination | | 10.1038/s41598-021-91514-7 |
| Utamura et al. | 2021 | | A novel deterministic epidemic model considering mass vaccination and lockdown against COVID-19 spread in Israel: numerical study | | 10.1101/2021.05.15.21257264 |
| Van Gordon et al. | 2021 | | Regional comparisons of COVID reporting rates, burden, and mortality age-structure using auxiliary data sources | | 10.1101/2021.08.18.21262248 |
| Van Heusden et al. | 2021 | | Pandemic policy design via feedback: a modelling story | | 10.1101/2021.09.23.21263924 |
| Van Egeren et al. | 2021 | | Rapid relaxation of pandemic restrictions after vaccine rollout favors growth of SARS-CoV-2 variants: a model-based analysis | | 10.1371/journal.pone.0258997 |
| Gómez et al. | 2022 | | Testing and vaccination to reduce the impact of COVID-19 in nursing homes: an agent-based approach | | 10.1186/s12879-022-07385-4 |
| Viana et al. | 2021 | | Controlling the pandemic during the SARS-CoV-2 vaccination rollout | | 10.1038/s41467-021-23938-8 |
| Vignals et al. | 2021 | | Barrier gesture relaxation during vaccination campaign in France: modelling impact of waning immunity | | 10.1101/2021.08.29.21262788 |
| Vilches et al. | 2022 | | COVID-19 hospitalizations and deaths averted under an accelerated vaccination program in northeastern and southern regions of the USA | | 10.1016/j.lana.2021.100147 |
| Vilches et al. | 2022 | | Estimating COVID-19 infections, hospitalizations, and deaths following the US vaccination campaigns during the pandemic | | 10.1001/jamanetworkopen.2021.42725 |
| Vilches et al. | 2021 | | Importance of non-pharmaceutical interventions in the COVID-19 vaccination era: a case study of the Seychelles | | 10.7189/jogh.11.03104 |
| Vilches et al. | 2021 | | Multifaceted strategies for the control of COVID-19 outbreaks in long-term care facilities in Ontario, Canada | | 10.1101/2020.12.04.20244194 |
| Vilches et al. | 2021 | | Projecting the impact of a two-dose COVID-19 vaccination campaign in Ontario, Canada | | 10.1016/j.vaccine.2021.03.058 |
| Vilches et al. | 2021 | | Vaccination efforts in Brazil: scenarios and perspectives under a mathematical modeling approach | | [10.1101/2021.02.22.21252208](https://doi.org/10.1101/2021.02.22.21252208) |
| Volodymyrovych et al. | 2021 | | Pharmaco economics analysis of COVID-19 vaccines in Ukraine | | 10.9734/jpri/2021/v33i32A31727 |
| Walker et al. | 2022 | | Modeling strategies for the allocation of SARS-CoV-2 vaccines in the United States | | [10.1016/j.vaccine.2022.02.015](https://doi.org/10.1016/j.vaccine.2022.02.015) |
| Wang et al. | 2021 | | Economic evaluation for mass vaccination against COVID-19 | | 10.1016/j.jfma.2021.05.020 |
| Wang et al. | 2022 | | From policy to prediction: forecasting COVID-19 dynamics under imperfect vaccination | | 2201.0593 |
| Wang et al. | 2021 | | Asymptomatic SARS-CoV-2 infection and the demography of COVID-19 | | 10.1101/2021.09.03.21262757 |
| Webb | 2021 | | A COVID-19 epidemic model predicting the effectiveness of vaccination in the US | | [10.3390/idr13030062](https://doi.org/10.3390%2Fidr13030062) |
| Więcek et al. | 2022 | | Testing fractional doses of COVID-19 vaccines | | 10.1073/pnas.2116932119 |
| Wiȩcek et al. | 2021 | | Could vaccine dose stretching reduce COVID-19 deaths? | | 10.3386/w29018 |
| Wieland et al. | 2021 | | Simulation of SARS-CoV-2 pandemic in Germany with ordinary differential equations in MATLAB | | [10.1109/ICSTCC52150.2021.9607181](https://doi.org/10.1109/ICSTCC52150.2021.9607181) |
| Wirtz | 2021 | | Decline in mitigation readiness facilitated second waves of SARS-CoV-2 | | 10.1101/2021.02.10.21251523 |
| Wong et al. | 2021 | | SIR Simulation of COVID-19 Pandemic in Malaysia: Will the vaccination program be effective? | | 10.48550/arXiv.2101.07494 |
| Xavier et al. | 2022 | | Timing the race of vaccination, new variants, and relaxing restrictions during COVID-19 pandemic | | [10.1016/j.jocs.2022.101660](https://doi.org/10.1016/j.jocs.2022.101660) |
| Xiong et al. | 2022 | | Economic value of vaccines to address the COVID-19 pandemic in Hong Kong: a cost-effectiveness analysis | | [10.3390/vaccines10040495](https://doi.org/10.3390/vaccines10040495) |
| Yang et al. | 2021 | | COVID-19 pandemic dynamics in India, the SARS-CoV-2 Delta variant, and implications for vaccination | | 10.1101/2021.06.21.21259268 |
| Yang et al. | 2022 | | COVID-19 pandemic dynamics in South Africa and epidemiological characteristics of three variants of concern (Beta, Delta, and Omicron) | | 10.1101/2021.12.19.21268073 |
| Yang et al. | 2021 | | Despite vaccination, China needs non-pharmaceutical interventions to prevent widespread outbreaks of COVID-19 in 2021 | | 10.1038/s41562-021-01155-z |
| Yang et al. | 2021 | | Epidemiological characteristics of three SARS-CoV-2 variants of concern and implications for future COVID-19 pandemic outcomes | | 10.1101/2021.05.19.21257476 |
| Yang et al. | 2022 | | Estimating data-driven coronavirus disease 2019 mitigation strategies for safe university reopening | | 10.1098/rsif.2021.0920 |
| Yang et al. | 2021 | | Simulating the impact of different vaccination policies on the COVID-19 pandemic in New York City | | 10.1101/2021.01.21.21250228 |
| Yang et al. | 2021 | | Assessing vaccination priorities for different ages and age-specific vaccination strategies of COVID-19 using an SEIR modelling approach | | .https://10.1371/journal.pone.0261236 |
| Young et al. | 2021 | | Interplay between COVID-19 vaccines and social measures for ending the SARS-CoV-2 pandemic | | 10.12688/f1000research.54729.1 |
| Yu et al. | 2021 | | Dynamic optimization of COVID-19 vaccine prioritization in the context of limited supply | | 10.21203/rs.3.rs-257573/v1 |
| Yuan et al. | 2022 | | Projections of the transmission of the Omicron variant for Toronto, Ontario, and Canada using surveillance data following recent changes in testing policies | | 10.1016/j.idm.2022.03.004 |
| Zachreson et al. | 2022 | | COVID-19 in low-tolerance border quarantine systems: impact of the Delta variant of SARS-CoV-2 | | 10.1126/sciadv.abm3624 |
| Zhang et al. | 2021 | | Mathematical assessment of constant and time-dependent control measures on the dynamics of the novel coronavirus: an application of optimal control theory | | 10.1016/j.rinp.2021.104971 |
| Zhang et al. | 2021 | | An extended COVID-19 epidemiological model with vaccination and multiple interventions for controlling COVID-19 outbreaks in the UK | | 10.1101/2021.03.10.21252748 |
| Zhang et al. | 2022 | | Modeling vaccinations, virus variants and lockdown: early guidance for SARS-CoV-2 health policies in India | | 10.1101/2022.02.02.22270353 |
| Zhao et al. | 2021 | | Stringent nonpharmaceutical interventions are crucial for curbing COVID-19 transmission in the course of vaccination: a case study of South and Southeast Asian countries | | 10.3390/healthcare9101292 |
| Zhao et al. | 2021 | | The optimal vaccination strategy to control COVID-19: a modeling study based on the transmission scenario in Wuhan City, China | | 10.1186/s40249-021-00922-4 |
| Zhao et al. | 2021 | | Threshold analyses on combinations of testing, population size, and vaccine coverage for COVID-19 control in a university setting | | 10.1371/journal.pone.0255864 |
| Zhao et al. | 2021 | | Will the large-scale vaccination succeed in containing the COVID-19 epidemic and how soon? | | 10.1101/2021.04.16.21255543 |
| Zhao et al. | 2021 | | Analysis of the vaccine effect on infectious diseases by system dynamics model | | 10.1145/3469678.3469696 |
| Zhou et al. | 2021 | | The resurgence risk of COVID-19 in the presence of immunity waning and ADE effect: a mathematical modelling study | | 10.1101/2021.08.25.21262601 |
| Suan Zhu et al. | 2022 | | Estimation of the reproduction number for COVID-19 based on latest vaccination results and the timing for herd-immunity: prospect for 2021 | | 10.26502/fjhs.051 |
| Zia et al. | 2021 | | Why a globally fair COVID-19 vaccination? An analysis based on agent-based simulation | | 10.1101/2021.10.03.21264494 |
| Zou et al. | 2021 | | Critical timing and extent of public health interventions to control outbreaks dominated by SARS-CoV-2 variants in Australia: a mathematical modelling study | | 10.1016/j.ijid.2021.11.024 |
| Zou et al. | 2022 | | Vaccination and quarantine effect on COVID-19 transmission dynamics incorporating Chinese-Spring-Festival travel rush: modeling and simulations | | 10.1007/s11538-021-00958-5 |
