# Supplementary material for: Predictive models for health outcomes due to SARS-CoV-2, including the effect of vaccination: a systematic review
Source: Syst Rev. 2024 Jan 16;13:30. doi: 10.1186/s13643-023-02411-1 (PMC10790449; doi:10.1186/s13643-023-02411-1)
Supplement: Supplementary file 7 — Supplementary Material Supplementary material N°. 7. Quality assessment (Johanna Briggs Institute Checklist, PROBAST Checklist). [file 13643_2023_2411_MOESM7_ESM.zip › 12903_2024_5066_MOESM7_ESM/Supp. N°. 7 JBI.docx]

**Supplementary material N°. 7. Quality assessment**

**Johanna Briggs Institute Checklist**

| **Domains** | **Lasser et al. (2022)** | **Acuña et al. (2021)** | **Adibi et al. (2021)** | **Mahmoud et al. (2021)** | **Agarwal et al. (2021)** |
| --- | --- | --- | --- | --- | --- |
|  | **02-06-22** | **05-06-22** | **05-06-22** | **05-06-22** | **05-06-22** |
| 1. Were the criteria for inclusion in the sample clearly defined? | Yes | Unclear | Unclear | No | No |
| 2. Were the study subjects and the setting described in detail? | Yes | No | No | No | No |
| 3. Was the exposure measured in a valid and reliable way? | Yes | Yes | Yes | Unclear | Unclear |
| 4. Were objective, standard criteria used for measurement of the condition? | Yes | Unclear | Unclear | Yes | Yes |
| 5. Were confounding factors identified? | Unclear | Unclear | Unclear | Unclear | Unclear |
| 6. Were strategies to deal with confounding factors stated? | Yes | Unclear | Unclear | Unclear | Unclear |
| 7. Were the outcomes measured in a valid and reliable way? | Yes | Yes | Yes | Yes | Yes |
| 8. Was appropriate statistical analysis used? | Yes | Yes | Yes | Yes | Yes |
| **conclusion** | Low risk | High risk | High risk | High risk | High risk |
| **Reason for risk assigned** | - | This study was based on the analysis of a database but information on the population of origin is not detailed | This study was based on the analysis of a database but information on the population of origin is not detailed | This study lacks details related to the population used. These are based on simulations | This study lacks details related to the population used. These are based on simulations |
|  |  |  |  |  |  |
|  |  |  |  |  |  |
| **Domains** | **Aguas et al. (2021)** | **Aguiar et al. (2021)** | **Aguilar-Canto et al. (2021) (2)** | **Aguilar-Canto et al. (2022) (1)** | **Ainslie et al. (2021)** |
|  | **05-06-22** | **05-06-22** | **05-06-22** | **05-06-22** | **05-06-22** |
| 1. Were the criteria for inclusion in the sample clearly defined? | No | No | Unclear | No | Unclear |
| 2. Were the study subjects and setting described in detail? | No | No | Unclear | No | No |
| 3. Was the exposure measured in a valid and reliable way? | Unclear | Unclear | Unclear | Unclear | Yes |
| 4. Were objective and standard criteria used to measure the condition? | Yes | Yes | Yes | Yes | Yes |
| 5. Were confounding factors identified? | Unclear | Unclear | Unclear | Unclear | Yes |
| 6. Were strategies established to deal with confounding factors? | Unclear | Unclear | Unclear | Unclear | Yes |
| 7. Were the outcomes measured in a valid and reliable way? | Yes | Yes | Yes | Yes | Yes |
| 8. Was an appropriate statistical analysis used? | Yes | Yes | Yes | Yes | Yes |
| **Conclusion** | High risk | High risk | High risk | High risk | High risk |
| **Justification of the assigned risk** | This study lacks details related to the population used. These are based on simulations | This study lacks details related to the population used. These are based on simulations | In this study, the origin of the data is not clear, despite the fact that various sources are mentioned in the different countries included | This study lacks details related to the population used. These are based on simulations | This study is based on data from another matrix |
|  |  |  |  |  |  |
|  |  |  |  |  |  |
| **Domains** | **Alagoz et al. (2021)** | **Albani et al. (2021) (1)** | **Albani et al. (2021) (2)** | **Aldila et al. (2021)** | **Almajose et al. (2021)** |
|  | **05-06-22** | **05-06-22** | **06-06-22** | **07-06-22** | **08-06-22** |
| 1. Were the criteria for inclusion in the sample clearly defined? | No | No | Yes | No | Yes |
| 2. Were the study subjects and setting described in detail? | No | No | Yes | No | Unclear |
| 3. Was the exposure measured in a valid and reliable way? | Unclear | Unclear | Yes | Unclear | Yes |
| 4. Were objective and standard criteria used to measure the condition? | Yes | Yes | Yes | Yes | Yes |
| 5. Were confounding factors identified? | Unclear | Unclear | Yes | Unclear | Yes |
| 6. Were strategies established to deal with confounding factors? | Unclear | Unclear | Yes | Unclear | Yes |
| 7. Were the outcomes measured in a valid and reliable way? | Yes | Yes | Yes | Yes | Yes |
| 8. Was an appropriate statistical analysis used? | Yes | Yes | Yes | Yes | Yes |
| **Conclusion** | High risk | High risk | Low risk | High risk | Low risk |
| **Justification of the assigned risk** | This study lacks details related to the population used. These are based on simulations | This study lacks details related to the population used. These are based on simulations | - | This study lacks details related to the population used. These are based on simulations | - |
|  |  |  |  |  |  |
|  |  |  |  |  |  |
| **Domains** | **Amaku et al. (2021)** | **Amaral et al. (2021)** | **Amouch et al. (2021)** | **Antonini et al. (2021)** | **Are et al. (2021)** |
|  | **05-06-22** | **05-06-22** | **05-06-22** | **05-06-22** | **05-06-22** |
| 1. Were the criteria for inclusion in the sample clearly defined? | No | Unclear | No | Yes | Unclear |
| 2. Were the study subjects and setting described in detail? | No | Unclear | No | Unclear | Unclear |
| 3. Was the exposure measured in a valid and reliable way? | Unclear | Yes | Unclear | Yes | Yes |
| 4. Were objective and standard criteria used to measure the condition? | Yes | Yes | Yes | Yes | Yes |
| 5. Were confounding factors identified? | Unclear | Unclear | Unclear | Yes | Unclear |
| 6. Were strategies established to deal with confounding factors? | Unclear | Unclear | Unclear | Yes | Unclear |
| 7. Were the outcomes measured in a valid and reliable way? | Yes | Yes | Yes | Yes | Yes |
| 8. Was an appropriate statistical analysis used? | Yes | Yes | Yes | Yes | Yes |
| **Cconclusion** | High risk | High risk | High risk | Low risk | High risk |
| **Justification of the assigned risk** | This study lacks details related to the population used. These are based on simulations | This study was based on the analysis of a database but information on the population of origin is not detailed | This study lacks details related to the population used. These are based on simulations | - | This study was based on the analysis of a database but information on the population of origin is not detailed |
|  |  |  |  |  |  |
|  |  |  |  |  |  |
| **Domains** | **Arslan et al. (2021) (1)** | **Arslan et al. (2021) (2)** | **Aruffo et al. (2021) (1)** | **Aruffo et al. (2021) (2)** | **Adiga et al. (2021)** |
|  | **05-06-22** | **05-06-22** | **05-06-22** | **05-06-22** | **05-06-22** |
| 1. Were the criteria for inclusion in the sample clearly defined? | Yes | Yes | Yes | Yes | Yes |
| 2. Were the study subjects and setting described in detail? | Unclear | Unclear | Unclear | Unclear | Unclear |
| 3. Was the exposure measured in a valid and reliable way? | Yes | Yes | Yes | Yes | Yes |
| 4. Were objective and standard criteria used to measure the condition? | Yes | Yes | Yes | Yes | Yes |
| 5. Were confounding factors identified? | Yes | Yes | Yes | Yes | Yes |
| 6. Were strategies established to deal with confounding factors? | Yes | Yes | Yes | Yes | Yes |
| 7. Were the outcomes measured in a valid and reliable way? | Yes | Yes | Yes | Yes | Yes |
| 8. Was an appropriate statistical analysis used? | Yes | Yes | Yes | Yes | Yes |
| **Conclusion** | Low risk | Low risk | Low risk | Low risk | Low risk |
| **Justification of the assigned risk** | - | - | - | - | - |
|  |  |  |  |  |  |
|  |  |  |  |  |  |
| **Domains** | **Avila-Ponce de León et al. (2021)** | **Ayoub et al. (2021)** | **Ayoub et al. (2022)** | **Bablani et al. (2021)** | **Babus et al. (2020)** |
|  | **05-06-22** | **11-06-22** | **11-06-22** | **11-06-22** | **11-06-22** |
| 1. Were the criteria for inclusion in the sample clearly defined? | No | Yes | Yes | No | No |
| 2. Were the study subjects and setting described in detail? | No | Unclear | Unclear | No | No |
| 3. Was the exposure measured in a valid and reliable way? | Unclear | Yes | Yes | Unclear | Unclear |
| 4. Were objective and standard criteria used to measure the condition? | Yes | Yes | Yes | Yes | Yes |
| 5. Were confounding factors identified? | Unclear | Yes | Yes | Unclear | Unclear |
| 6. Were strategies established to deal with confounding factors? | Unclear | Yes | Yes | Unclear | Unclear |
| 7. Were the outcomes measured in a valid and reliable way? | Yes | Yes | Yes | Yes | Yes |
| 8. Was an appropriate statistical analysis used? | Yes | Yes | Yes | Yes | Yes |
| **Conclusion** | High risk | Low risk | Low risk | High risk | High risk |
| **Justification of the assigned risk** | This study lacks details related to the population used. These are based on simulations | - | - | This study lacks details related to the population used. These are based on simulations | This study lacks details related to the population used. These are based on simulations |
|  |  |  |  |  |  |
|  |  |  |  |  |  |
| **Domains** | **Badfar et al. (2021)** | **Bautista et al. (2021)** | **Barlow et al. (2021)** | **Barmpounakis et al. (2022)** | **Barnard et al. (2021)** |
|  | **11-06-22** | **11-06-22** | **11-06-22** | **11-06-22** | **11-06-22** |
| 1. Were the criteria for inclusion in the sample clearly defined? | No | Yes | Yes | Yes | No |
| 2. Were the study subjects and setting described in detail? | No | Unclear | Unclear | Unclear | No |
| 3. Was the exposure measured in a valid and reliable way? | Unclear | Yes | Yes | Yes | Unclear |
| 4. Were objective and standard criteria used to measure the condition? | Yes | Yes | Yes | Yes | Yes |
| 5. Were confounding factors identified? | Unclear | Yes | Yes | Yes | Unclear |
| 6. Were strategies established to deal with confounding factors? | Unclear | Yes | Yes | Yes | Unclear |
| 7. Were the outcomes measured in a valid and reliable way? | Yes | Yes | Yes | Yes | Yes |
| 8. Was an appropriate statistical analysis used? | Yes | Yes | Yes | Yes | Yes |
| **Conclusion** | High risk | Low risk | Low risk | Low risk | High risk |
| **Justification of the assigned risk** | This study lacks details related to the population used. These are based on simulations | - | - | - | This study lacks details related to the population used. These are based on simulations |
|  |  |  |  |  |  |
|  |  |  |  |  |  |
| **Domains** | **Barnard et al. (2021)** | **Barreiro et al. (2021)** | **Bartsch et al. (2020)** | **Bartsch et al. (2021)** | **Bartsch et al. (2021)** |
|  | **11-06-22** | **11-06-22** | **11-06-22** | **11-06-22** | **11-06-22** |
| 1. Were the criteria for inclusion in the sample clearly defined? | No | No | No | No | No |
| 2. Were the study subjects and setting described in detail? | No | No | No | No | No |
| 3. Was the exposure measured in a valid and reliable way? | Unclear | Unclear | Unclear | Unclear | Unclear |
| 4. Were objective and standard criteria used to measure the condition? | Yes | Yes | Yes | Yes | Yes |
| 5. Were confounding factors identified? | Unclear | Unclear | Unclear | Unclear | Unclear |
| 6. Were strategies established to deal with confounding factors? | Unclear | Unclear | Unclear | Unclear | Unclear |
| 7. Were the outcomes measured in a valid and reliable way? | Yes | Yes | Yes | Yes | Yes |
| 8. Was an appropriate statistical analysis used? | Yes | Yes | Yes | Yes | Yes |
| **Conclusion** | High risk | High risk | High risk | High risk | High risk |
| **Justification of the assigned risk** | This study lacks details related to the population used. These are based on simulations | This study lacks details related to the population used. These are based on simulations | This study lacks details related to the population used. These are based on simulations | This study lacks details related to the population used. These are based on simulations | This study lacks details related to the population used. These are based on simulations |
|  |  |  |  |  |  |
|  |  |  |  |  |  |
| **Domains** | **Bauer et al. (2021)** | **Below et al. (2021)** | **Berec et al. (2022)** | **Berkane et al. (2021)** | **Bertsimas et al. (2021)** |
|  | **11-06-22** | **11-06-22** | **11-06-22** | **11-06-22** | **11-06-22** |
| 1. Were the criteria for inclusion in the sample clearly defined? | No | No | Yes | Yes | No |
| 2. Were the study subjects and setting described in detail? | No | No | Unclear | Unclear | Unclear |
| 3. Was the exposure measured in a valid and reliable way? | Yes | Unclear | Yes | Yes | Unclear |
| 4. Were objective and standard criteria used to measure the condition? | Yes | Yes | Yes | Yes | Unclear |
| 5. Were confounding factors identified? | Yes | Unclear | Yes | Yes | No |
| 6. Were strategies established to deal with confounding factors? | Yes | Unclear | Yes | Yes | No |
| 7. Were the outcomes measured in a valid and reliable way? | Yes | Yes | Yes | Yes | Yes |
| 8. Was an appropriate statistical analysis used? | Yes | Yes | Yes | Yes | Yes |
| **Conclusion** | Low risk | High risk | Low risk | Low risk | High risk |
| **Justification of the assigned risk** | - | This study lacks details related to the population used. These are based on simulations | - | - | This study lacks methodological details related to population and exposure |
|  |  |  |  |  |  |
|  |  |  |  |  |  |
| **Domains** | **Bhattacharya et al. (2021)** | **Bianchin et al. (2021)** | **Bilinski et al. (2021) (1)** | **Bilinski et al. (2021) (2)** | **Booton et al. (2021)** |
|  | **11-06-22** | **11-06-22** | **11-06-22** | **11-06-22** | **11-06-22** |
| 1. Were the criteria for inclusion in the sample clearly defined? | No | Yes | Unclear | Unclear | No |
| 2. Were the study subjects and setting described in detail? | No | Unclear | Yes | Yes | No |
| 3. Was the exposure measured in a valid and reliable way? | Yes | Yes | Yes | Yes | Unclear |
| 4. Were objective and standard criteria used to measure the condition? | Yes | Yes | Yes | Yes | Yes |
| 5. Were confounding factors identified? | Yes | Yes | Yes | Yes | Unclear |
| 6. Were strategies established to deal with confounding factors? | Yes | Yes | Yes | Yes | Unclear |
| 7. Were the outcomes measured in a valid and reliable way? | Yes | Yes | Yes | Yes | Yes |
| 8. Was an appropriate statistical analysis used? | Yes | Yes | Yes | Yes | Yes |
| **Conclusion** | Low risk | Low risk | Low risk | Low risk | Unclear |
| **Justification of the assigned risk** | - | - | - | - | This study does not have a well-defined population and the model does not provide enough detail in its measurements |
|  |  |  |  |  |  |
|  |  |  |  |  |  |
| **Domains** | **Borchering et al. (2021)** | **Borchering et al. (2022)** | **Bosseti et al. (2022) (1)** | **Bosseti et al. (2021) (2)** | **Bosseti et al. (2022)** |
|  | **11-06-22** | **11-06-22** | **11-06-22** | **11-06-22** | **11-06-22** |
| 1. Were the criteria for inclusion in the sample clearly defined? | No | No | No | No | No |
| 2. Were the study subjects and setting described in detail? | No | No | Unclear | Unclear | Unclear |
| 3. Was the exposure measured in a valid and reliable way? | Unclear | Unclear | Yes | Yes | Yes |
| 4. Were objective and standard criteria used to measure the condition? | Yes | Yes | Yes | Yes | Yes |
| 5. Were confounding factors identified? | Unclear | Unclear | Yes | Yes | Yes |
| 6. Were strategies established to deal with confounding factors? | Unclear | Unclear | Yes | Yes | Yes |
| 7. Were the outcomes measured in a valid and reliable way? | Yes | Yes | Yes | Yes | Yes |
| 8. Was an appropriate statistical analysis used? | Yes | Yes | Yes | Yes | Yes |
| **Conclusion** | High risk | High risk | Low risk | Low risk | Low risk |
| **Justification of the assigned risk** | Insufficient methodological details are found in this study | Insufficient methodological details are found in this study | - | - | - |
|  |  |  |  |  |  |
|  |  |  |  |  |  |
| **Domains** | **Bousquet et al. (2022)** | **Bowie et al. (2021)** | **Bracis et al. (2022)** | **Braun et al. (2020)** | **Brown et al. (2021)** |
|  | **11-06-22** | **11-06-22** | **11-06-22** | **11-06-22** | **11-06-22** |
| 1. Were the criteria for inclusion in the sample clearly defined? | Unclear | No | Yes | No | Unclear |
| 2. Were the study subjects and setting described in detail? | Unclear | No | Unclear | No | Unclear |
| 3. Was the exposure measured in a valid and reliable way? | Yes | Yes | Yes | Yes | Yes |
| 4. Were objective and standard criteria used to measure the condition? | Yes | Yes | Yes | Yes | Yes |
| 5. Were confounding factors identified? | Yes | Yes | Yes | Yes | Yes |
| 6. Were strategies established to deal with confounding factors? | Yes | Yes | Yes | Yes | Yes |
| 7. Were the outcomes measured in a valid and reliable way? | Yes | Yes | Yes | Yes | Yes |
| 8. Was an appropriate statistical analysis used? | Yes | Yes | Yes | Yes | Yes |
| **Conclusion** | Unclear | High risk | Low risk | High risk | Unclear |
| **Justification of the assigned risk** | This study does not define the population very well in terms of criteria, but it shows the databases used | There is not enough detail about the population used | - | There is no information on the source of the data | There is not enough information on the population used |
|  |  |  |  |  |  |
|  |  |  |  |  |  |
| **Domains** | **Brüningk et al. (2022)** | **Bu et al. (2021)** | **Bubar et al. (2021)** | **Buckner et al. (2021)** | **Bugalia et al. (2022)** |
|  | **11-06-22** | **11-06-22** | **11-06-22** | **11-06-22** | **11-06-22** |
| 1. Were the criteria for inclusion in the sample clearly defined? | Yes | No | No | No | Unclear |
| 2. Were the study subjects and setting described in detail? | Yes | No | No | No | Yes |
| 3. Was the exposure measured in a valid and reliable way? | Yes | Yes | Yes | Yes | Yes |
| 4. Were objective and standard criteria used to measure the condition? | Yes | Yes | Yes | Yes | Yes |
| 5. Were confounding factors identified? | Yes | Yes | Yes | Yes | Yes |
| 6. Were strategies established to deal with confounding factors? | Yes | Yes | Yes | Yes | Yes |
| 7. Were the outcomes measured in a valid and reliable way? | Yes | Yes | Yes | Yes | Yes |
| 8. Was an appropriate statistical analysis used? | Yes | Yes | Yes | Yes | Yes |
| **Conclusion** | Low risk | High risk | High risk | High risk | Low risk |
| **Justification of the assigned risk** | - | There is no information on the source of the data | There is no information on the source of the data | There is no information on the source of the data | - |
|  |  |  |  |  |  |
| **Domains** | **Buhat et al. (2021)** | **Buonomo et al. (2021)** | **Hohenegger et al. (2021)** | **Caetano et al. (2021)** | **Cai et al. (2022)** |
|  | **11-06-22** | **11-06-22** | **11-06-22** | **12-06-22** | **12-06-22** |
| 1. Were the criteria for inclusion in the sample clearly defined? | Yes | Unclear | Yes | Yes | Yes |
| 2. Were the study subjects and setting described in detail? | Unclear | Yes | Unclear | Unclear | Unclear |
| 3. Was the exposure measured in a valid and reliable way? | Yes | Yes | Yes | Yes | Yes |
| 4. Were objective and standard criteria used to measure the condition? | Yes | Yes | Yes | Yes | Yes |
| 5. Were confounding factors identified? | Yes | Yes | Yes | Yes | Yes |
| 6. Were strategies established to deal with confounding factors? | Yes | Yes | Yes | Yes | Yes |
| 7. Were the outcomes measured in a valid and reliable way? | Yes | Yes | Yes | Yes | Yes |
| 8. Was an appropriate statistical analysis used? | Yes | Yes | Yes | Yes | Yes |
| **Conclusion** | Low risk | Low risk | Low risk | Low risk | Low risk |
| **Justification of the assigned risk** | - | Although the sources are not so defined, a low risk is taken because their reliability is explained. | - | - | - |
|  |  |  |  |  |  |
| **Domains** | **Campos et al. (2021)** | **Canga et al. (2022)** | **Buchwald et al. (2021)** | **Catalá et al. (2021)** | **Cazelles et al. (2021)** |
|  | **12-06-22** | **12-06-22** | **12-06-22** | **12-06-22** | **12-06-22** |
| 1. Were the criteria for inclusion in the sample clearly defined? | No | Unclear | Yes | Yes | No |
| 2. Were the study subjects and setting described in detail? | No | No | Unclear | Unclear | No |
| 3. Was the exposure measured in a valid and reliable way? | Yes | Yes | Yes | Yes | Yes |
| 4. Were objective and standard criteria used to measure the condition? | Yes | Yes | Yes | Yes | Yes |
| 5. Were confounding factors identified? | Yes | Yes | Yes | Yes | Yes |
| 6. Were strategies established to deal with confounding factors? | Yes | Yes | Yes | Yes | Yes |
| 7. Were the outcomes measured in a valid and reliable way? | Yes | Yes | Yes | Yes | Yes |
| 8. Was an appropriate statistical analysis used? | Yes | Yes | Yes | Yes | Yes |
| **Conclusion** | High risk | Unclear | Low risk | Low risk | High risk |
| **Justification of the assigned risk** | There is no information on the source of the data | There are no data referring to the population used. There are unamplified data that can help inferring information | - | - | There is no information on the source of the data |
|  |  |  |  |  |  |
|  |  |  |  |  |  |
| **Domains** | **Chang et al. (2021)** | **Chapman et al. (2022) (1)** | **Chapman et al. (2022) (2)** | **Chen et al. (2020)** | **Chen et al. (2021) (1)** |
|  | **12-06-22** | **12-06-22** | **12-06-22** | **12-06-22** | **12-06-22** |
| 1. Were the criteria for inclusion in the sample clearly defined? | No | Yes | Yes | No | No |
| 2. Were the study subjects and setting described in detail? | No | Yes | Yes | No | No |
| 3. Was the exposure measured in a valid and reliable way? | Yes | Yes | Yes | Yes | Yes |
| 4. Were objective and standard criteria used to measure the condition? | Yes | Yes | Yes | Yes | Yes |
| 5. Were confounding factors identified? | Yes | Yes | Yes | Yes | Yes |
| 6. Were strategies established to deal with confounding factors? | Yes | Yes | Yes | Yes | Yes |
| 7. Were the outcomes measured in a valid and reliable way? | Yes | Yes | Yes | Yes | Yes |
| 8. Was an appropriate statistical analysis used? | Yes | Yes | Yes | Yes | Yes |
| **Conclusion** | High risk | Low risk | Low risk | High risk | High risk |
| **Justification of the assigned risk** | There is no information on the source of the data | - | - | There is no information on the source of the data | There is no information on the source of the data |
|  |  |  |  |  |  |
| **Domains** | **Chen et al. (2021) (2)** | **Chen et al. (2021) (3)** | **Chen et al. (2021) (4)** | **Chen et al. (2022)** | **Chen et al. (2021)** |
|  | **12-06-22** | **12-06-22** | **12-06-22** | **12-06-22** | **12-06-22** |
| 1. Were the criteria for inclusion in the sample clearly defined? | No | No | Yes | Yes | Yes |
| 2. Were the study subjects and setting described in detail? | No | No | Unclear | Yes | Unclear |
| 3. Was the exposure measured in a valid and reliable way? | Yes | Yes | Yes | Yes | Yes |
| 4. Were objective and standard criteria used to measure the condition? | Yes | Yes | Yes | Yes | Yes |
| 5. Were confounding factors identified? | Yes | Yes | Yes | Yes | Yes |
| 6. Were strategies established to deal with confounding factors? | Yes | Yes | Yes | Yes | Yes |
| 7. Were the outcomes measured in a valid and reliable way? | Yes | Yes | Yes | Yes | Yes |
| 8. Was an appropriate statistical analysis used? | Yes | Yes | Yes | Yes | Yes |
| **Conclusion** | High risk | High risk | Low risk | Low risk | Low risk |
| **Justification of the assigned risk** | There is no information on the source of the data | There is no information on the source of the data | - | - | - |
|  |  |  |  |  |  |
| **Domains** | **Childs et al. (2021)** | **Chinazzi et al. (2020)** | **Chinchilla et al. (2021)** | **Choi et al. (2021)** | **Choi et al. (2021)** |
|  | **12-06-22** | **12-06-22** | **12-06-22** | **12-06-22** | **12-06-22** |
| 1. Were the criteria for inclusion in the sample clearly defined? | No | No | Yes | No | No |
| 2. Were the study subjects and setting described in detail? | No | No | No | No | No |
| 3. Was the exposure measured in a valid and reliable way? | Unclear | Yes | Yes | Yes | Yes |
| 4. Were objective and standard criteria used to measure the condition? | No | Yes | Yes | Yes | Yes |
| 5. Were confounding factors identified? | Unclear | Yes | Yes | Yes | Yes |
| 6. Were strategies established to deal with confounding factors? | Unclear | Unclear | Yes | Yes | Yes |
| 7. Were the outcomes measured in a valid and reliable way? | Yes | Yes | Yes | Yes | Yes |
| 8. Was an appropriate statistical analysis used? | Yes | Yes | Yes | Yes | Yes |
| **Conclusion** | High risk | High risk | Low risk | High risk | High risk |
| **Justification of the assigned risk** | This study poorly describes the population, there is no mention of criteria to consider the disease | This study does not have detailed information on the information of the origin of the data | - | No details of the population included | No details of the population included are found, despite the fact that its origin is mentioned |
|  |  |  |  |  |  |
| **Domains** | **Chopra et al. (2021)** | **Chu et al. (2021)** | **Chun et al. (2021)** | **Chun et al. (2022)** | **Cipriano et al. (2021)** |
|  | **12-06-22** | **12-06-22** | **12-06-22** | **12-06-22** | **12-06-22** |
| 1. Were the criteria for inclusion in the sample clearly defined? | No | Unclear | No | Yes | Yes |
| 2. Were the study subjects and setting described in detail? | No | Unclear | Unclear | No | No |
| 3. Was the exposure measured in a valid and reliable way? | Yes | Yes | Yes | Yes | Yes |
| 4. Were objective and standard criteria used to measure the condition? | Yes | Yes | Yes | Yes | Yes |
| 5. Were confounding factors identified? | Yes | Unclear | NO | Yes | Yes |
| 6. Were strategies established to deal with confounding factors? | Yes | Unclear | No | Yes | Yes |
| 7. Were the outcomes measured in a valid and reliable way? | Yes | Yes | Yes | Yes | Yes |
| 8. Was an appropriate statistical analysis used? | Yes | Yes | Yes | Yes | Yes |
| **Conclusion** | High risk | High risk | High risk | Low risk | Low risk |
| **Justification of the assigned risk** | There are no details of the origin of the population data | The provenance of the data could be assumed, but it is not explicitly mentioned. | There are no details of the population and data as confounding variables or their analysis | - | - |
|  |  |  |  |  |  |
| **Domains** | **Cohen et al. (2021)** | **Colomer et al. (2021)** | **Colosi et al. (2022)** | **Colosi et al. (2022)** | **Contreras et al. (2020)** |
|  | **12-06-22** | **12-06-22** | **12-06-22** | **12-06-22** | **12-06-22** |
| 1. Were the criteria for inclusion in the sample clearly defined? | Yes | Yes | Yes | No | Unclear |
| 2. Were the study subjects and setting described in detail? | No | No | No | No | Unclear |
| 3. Was the exposure measured in a valid and reliable way? | Yes | Yes | Yes | Unclear | Yes |
| 4. Were objective and standard criteria used to measure the condition? | Yes | Yes | Yes | Unclear | Yes |
| 5. Were confounding factors identified? | Unclear | Yes | Yes | Unclear | Unclear |
| 6. Were strategies established to deal with confounding factors? | Unclear | Yes | Yes | Unclear | Unclear |
| 7. Were the outcomes measured in a valid and reliable way? | Yes | Yes | Yes | Unclear | Yes |
| 8. Was an appropriate statistical analysis used? | Yes | Yes | Yes | Yes | Yes |
| **Conclusion** | High risk | Low risk | Low risk | High risk | High risk |
| **Justification of the assigned risk** | The inclusion criteria in the population are not very well defined, nor is there clear evidence of confounding control or possible variables | - | - | This study does not clearly show the origin of the data and the way in which the model was developed | Provenance data not adequately described |
|  |  |  |  |  |  |
|  |  |  |  |  |  |
| **Domains** | **Contreras et al. (2021)** | **Contreras et al. (2022)** | **Cook et al. (2021)** | **Coudeville et al. (2021)** |  |
|  | **13-06-22** | **13-06-22** | **13-06-22** | **13-06-22** |  |
| 1. Were the criteria for inclusion in the sample clearly defined? | Yes | Yes | Yes | No |  |
| 2. Were the study subjects and setting described in detail? | No | No | Yes | No |  |
| 3. Was the exposure measured in a valid and reliable way? | Yes | Yes | Yes | Yes |  |
| 4. Were objective and standard criteria used to measure the condition? | Yes | Yes | Yes | Yes |  |
| 5. Were confounding factors identified? | Yes | Yes | Yes | Yes |  |
| 6. Were strategies established to deal with confounding factors? | Yes | Yes | Yes | Yes |  |
| 7. Were the outcomes measured in a valid and reliable way? | Yes | Yes | Yes | Yes |  |
| 8. Was an appropriate statistical analysis used? | Yes | Yes | Yes | Yes |  |
| **Conclusion** | Low risk | Low risk | Low risk | High risk |  |
| **Justification of the assigned risk** | - | - | - | Provenance data not adequately described |  |
|  |  |  |  |  |  |
| **Domains** | **Cruz et al. (2021)** | **Cuesta-Lazaro et al. (2021)** | **Dagpunar et al. (2021)** | **Darapaneni et al. (2021)** | **David et al. (2022)** |
|  | **13-06-22** | **14-06-22** | **14-06-22** | **15-06-22** | **15-06-22** |
| 1. Were the criteria for inclusion in the sample clearly defined? | Yes | Yes | No | Yes | Yes |
| 2. Were the study subjects and setting described in detail? | No | No | No | No | Yes |
| 3. Was the exposure measured in a valid and reliable way? | Yes | Yes | Yes | Yes | Yes |
| 4. Were objective and standard criteria used to measure the condition? | Yes | Yes | Yes | Yes | Yes |
| 5. Were confounding factors identified? | Yes | Yes | Yes | Yes | Yes |
| 6. Were strategies established to deal with confounding factors? | Yes | Yes | Yes | Yes | Yes |
| 7. Were the outcomes measured in a valid and reliable way? | Yes | Yes | Yes | Yes | Yes |
| 8. Was an appropriate statistical analysis used? | Yes | Yes | Yes | Yes | Yes |
| **Conclusion** | Low risk | Low risk | High risk | Low risk | Low risk |
| **Justification of the assigned risk** | - | - | Provenance data not adequately described | - | - |
|  |  |  |  |  |  |
| **Domains** | **Davies et al. (2021)** | **de Cellès et al. (2021)** | **de La Sen et al. (2021)** | **de La Sen et al. (2021)** | **Avila-Ponce de Leon et al. (2022)** |
|  | **15-06-22** | **17-06-22** | **17-06-22** | **17-06-22** | **18-06-22** |
| 1. Were the criteria for inclusion in the sample clearly defined? | Yes | Yes | No | No | No |
| 2. Were the study subjects and setting described in detail? | Yes | No | No | No | No |
| 3. Was the exposure measured in a valid and reliable way? | Yes | Yes | Yes | Yes | Yes |
| 4. Were objective and standard criteria used to measure the condition? | Yes | Yes | Yes | Yes | Yes |
| 5. Were confounding factors identified? | Yes | Yes | Yes | Yes | Yes |
| 6. Were strategies established to deal with confounding factors? | Yes | Yes | Yes | Yes | Yes |
| 7. Were the outcomes measured in a valid and reliable way? | Yes | Yes | Yes | Yes | Yes |
| 8. Was an appropriate statistical analysis used? | Yes | Yes | Yes | Yes | Yes |
| **Conclusion** | Low risk | Low risk | High risk | High risk | High risk |
| **Justification of the assigned risk** | - | - | Provenance data not adequately described | The origin of the data is not described | Provenance data not adequately described |
|  |  |  |  |  |  |
| **Domains** | **de Lima et al. (2021)** | **De-Leon et al. (2021)** | **Debrabant et al. (2021)** | **Demongeot et al. (2022)** | **DeWitt et al. (2021)** |
|  | **18-06-22** | **18-06-22** | **18-06-22** | **18-06-22** | **18-06-22** |
| 1. Were the criteria for inclusion in the sample clearly defined? | No | No | Yes | Yes | No |
| 2. Were the study subjects and setting described in detail? | No | No | No | No | No |
| 3. Was the exposure measured in a valid and reliable way? | Yes | Yes | Yes | Yes | Yes |
| 4. Were objective and standard criteria used to measure the condition? | Yes | Yes | Yes | Yes | Yes |
| 5. Were confounding factors identified? | Yes | Yes | Yes | Yes | Yes |
| 6. Were strategies established to deal with confounding factors? | Yes | Yes | Yes | Yes | Yes |
| 7. Were the outcomes measured in a valid and reliable way? | Yes | Yes | Yes | Yes | Yes |
| 8. Was an appropriate statistical analysis used? | Yes | Yes | Yes | Yes | Yes |
| **Conclusion** | High risk | High risk | Low risk | Low risk | High risk |
| **Justification of the assigned risk** | Provenance data not adequately described | Provenance data not adequately described | - | - | The origin of the data is not adequately described |
|  |  |  |  |  |  |
| **Domains** | **Di Domenico et al. (2021)** | **Di Fusco et al. (2022)** | **Diagne et al. (2021)** | **Diarra et al. (2022)** | **Dick et al. (2021)** |
|  | **18-06-22** | **18-06-22** | **18-06-22** | **18-06-22** | **18-06-22** |
| 1. Were the criteria for inclusion in the sample clearly defined? | No | Yes | Yes | No | Unclear |
| 2. Were the study subjects and setting described in detail? | No | Unclear | No | No | Unclear |
| 3. Was the exposure measured in a valid and reliable way? | Yes | Yes | Yes | Yes | Yes |
| 4. Were objective and standard criteria used to measure the condition? | Yes | Yes | Yes | Yes | Yes |
| 5. Were confounding factors identified? | Unclear | Unclear | Yes | Yes | Unclear |
| 6. Were strategies established to deal with confounding factors? | Yes | Yes | Yes | Yes | Yes |
| 7. Were the outcomes measured in a valid and reliable way? | Yes | Yes | Yes | Yes | Yes |
| 8. Was an appropriate statistical analysis used? | Yes | Yes | Yes | Yes | Yes |
| **Conclusion** | High risk | Low risk | Low risk | High risk | High risk |
| **Justification of the assigned risk** | Provenance data not adequately described | - | - | Provenance data not adequately described | Provenance data not adequately described |
|  |  |  |  |  |  |
| **Domains** | **Dimeglio et al. (2021) (1)** | **Dimeglio et al. (2021) (2)** | **Di Ddomenico et al. (2021) (2)** | **Dönges et al. (2021)** |  |
|  | **18-06-22** | **18-06-22** | **18-06-22** | **18-06-22** |  |
| 1. Were the criteria for inclusion in the sample clearly defined? | Yes | Yes | Yes | Yes |  |
| 2. Were the study subjects and setting described in detail? | No | No | Yes | Unclear |  |
| 3. Was the exposure measured in a valid and reliable way? | Yes | Yes | Yes | Yes |  |
| 4. Were objective and standard criteria used to measure the condition? | Yes | Yes | Yes | Yes |  |
| 5. Were confounding factors identified? | Yes | Yes | Yes | Yes |  |
| 6. Were strategies established to deal with confounding factors? | Yes | Yes | Yes | Yes |  |
| 7. Were the outcomes measured in a valid and reliable way? | Yes | Yes | Yes | Yes |  |
| 8. Was an appropriate statistical analysis used? | Yes | Yes | Yes | Yes |  |
| **Conclusion** | Low risk | Low risk | Low risk | Low risk |  |
| **Justification of the assigned risk** |  |  |  |  |  |
|  |  |  |  |  |  |
| **Domains** | **Du et al. (2022) (1)** | **Du et al. (2022) (2)** | **Dyson et al. (2021)** | **Españaet al. (2021)** | **Estadilla et al. (2021)** |
|  | **18-06-22** | **18-06-22** | **18-06-22** | **18-06-22** | **18-06-22** |
| 1. Were the criteria for inclusion in the sample clearly defined? | Yes | Yes | Yes | Yes | Yes |
| 2. Were the study subjects and setting described in detail? | Yes | Unclear | Unclear | Unclear | No |
| 3. Was the exposure measured in a valid and reliable way? | Yes | Yes | Yes | Yes | Yes |
| 4. Were objective and standard criteria used to measure the condition? | Yes | Yes | Yes | Yes | Yes |
| 5. Were confounding factors identified? | Yes | Yes | Yes | Yes | Yes |
| 6. Were strategies established to deal with confounding factors? | Yes | Yes | Yes | Yes | Yes |
| 7. Were the outcomes measured in a valid and reliable way? | Yes | Yes | Yes | Yes | Yes |
| 8. Was an appropriate statistical analysis used? | Yes | Yes | Yes | Yes | Yes |
| **Conclusion** | Low risk | Low risk | Low risk | Low risk | Low risk |
| **Justification of the assigned risk** | - | - | - | - | - |
|  |  |  |  |  |  |
| **Domains** | **Expósito et al. (2022)** | **Faranda et al. (2021)** | **Faucher et al. (2022)** | **Fawaz et al. (2021)** | **Feng et al. (2021)** |
|  | **18-06-22** | **18-06-22** | **18-06-22** | **18-06-22** | **18-06-22** |
| 1. Were the criteria for inclusion in the sample clearly defined? | No | Yes | Yes | Yes | No |
| 2. Were the study subjects and setting described in detail? | No | Unclear | Yes | Unclear | No |
| 3. Was the exposure measured in a valid and reliable way? | Yes | Yes | Yes | Yes | Unclear |
| 4. Were objective and standard criteria used to measure the condition? | Yes | Yes | Yes | Yes | Yes |
| 5. Were confounding factors identified? | Unclear | Yes | Yes | Yes | Unclear |
| 6. Were strategies established to deal with confounding factors? | Unclear | Yes | Yes | Yes | Unclear |
| 7. Were the outcomes measured in a valid and reliable way? | Unclear | Yes | Yes | Yes | Yes |
| 8. Was an appropriate statistical analysis used? | Yes | Yes | Yes | Yes | Yes |
| **Conclusion** | High risk | Low risk | Low risk | Low risk | High risk |
| **Justification of the assigned risk** | There are no details of the population of origin. As it is a simulation, the variables used or classified as confusion are not taken into account | - | - | - | This study lacks many methodological details |
|  |  |  |  |  |  |
| **Domains** | **Feng et al. (2022)** | **Ferrana et al. (2021)** | **Ferreria et al. (2021)** | **Souto et al. (2022)** | **Fierro et al. (2022)** |
|  | **18-06-22** | **18-06-22** | **18-06-22** | **18-06-22** | **18-06-22** |
| 1. Were the criteria for inclusion in the sample clearly defined? | Yes | Yes | Yes | Unclear | Yes |
| 2. Were the study subjects and setting described in detail? | No | No | Unclear | Unclear | No |
| 3. Was the exposure measured in a valid and reliable way? | Yes | Yes | Yes | Yes | Yes |
| 4. Were objective and standard criteria used to measure the condition? | Yes | Yes | Yes | Yes | Yes |
| 5. Were confounding factors identified? | Yes | Yes | Yes | Yes | Yes |
| 6. Were strategies established to deal with confounding factors? | Yes | Yes | Yes | Yes | Yes |
| 7. Were the outcomes measured in a valid and reliable way? | Yes | Yes | Yes | Yes | Yes |
| 8. Was an appropriate statistical analysis used? | Yes | Yes | Yes | Yes | Yes |
| **Conclusion** | Low risk | Low risk | Low risk | Unclear | Low risk |
| **Justification of the assigned risk** | - | - | - | There is no detail of the population used. They are simulations | - |
|  |  |  |  |  |  |
|  |  |  |  |  |  |
| **Domains** | **Flores-Arguedas et al. (2021)** | **Foy et al. (2021)** | **Frazier et al. (2022)** | **Frieswijk et al. (2021)** | **Fuady et al. (2021)** |
|  | **18-06-22** | **18-06-22** | **18-06-22** | **18-06-22** | **18-06-22** |
| 1. Were the criteria for inclusion in the sample clearly defined? | Unclear | Yes | Yes | Yes | Yes |
| 2. Were the study subjects and setting described in detail? | Unclear | Unclear | Unclear | Unclear | Yes |
| 3. Was the exposure measured in a valid and reliable way? | Yes | Yes | Yes | Yes | Yes |
| 4. Were objective and standard criteria used to measure the condition? | Yes | Yes | Yes | Yes | Yes |
| 5. Were confounding factors identified? | Yes | Yes | Yes | Yes | Yes |
| 6. Were strategies established to deal with confounding factors? | Yes | Yes | Yes | Yes | Yes |
| 7. Were the outcomes measured in a valid and reliable way? | Yes | Yes | Yes | Yes | Yes |
| 8. Was an appropriate statistical analysis used? | Yes | Yes | Yes | Yes | Yes |
| **Conclusion** | Unclear | Low risk | Low risk | Low risk | Low risk |
| **Justification of the assigned risk** | There is no detail of the population used. They are simulations | - | - | - | - |
|  |  |  |  |  |  |
|  |  |  |  |  |  |
| **Domains** | **Fujii et al. (2021)** | **Furuse (2021)** | **Gabriele-Rivet et al. (2021)** | **Galanti et al. (2021)** | **Galloway et al. (2021)** |
|  | **18-06-22** | **18-06-22** | **18-06-22** | **19-06-22** | **19-06-22** |
| 1. Were the criteria for inclusion in the sample clearly defined? | Yes | No | No | Yes | No |
| 2. Were the study subjects and setting described in detail? | Unclear | No | No | Unclear | No |
| 3. Was the exposure measured in a valid and reliable way? | Yes | Unclear | Unclear | Yes | Unclear |
| 4. Were objective and standard criteria used to measure the condition? | Yes | Yes | Yes | Yes | Yes |
| 5. Were confounding factors identified? | Yes | Unclear | Unclear | Yes | Unclear |
| 6. Were strategies established to deal with confounding factors? | Yes | Unclear | Unclear | Yes | Unclear |
| 7. Were the outcomes measured in a valid and reliable way? | Yes | Yes | Yes | Yes | Yes |
| 8. Was an appropriate statistical analysis used? | Yes | Yes | Yes | Yes | Yes |
| **Conclusion** | Low risk | High risk | High risk | Low risk | High risk |
| **Justification of the assigned risk** | - | This study lacks many methodological details | This study lacks many methodological details | - | This study lacks many methodological details |
|  |  |  |  |  |  |
|  |  |  |  |  |  |
| **Domains** | **Gandjour (2022) (1)** | **Gandjour (2022) (2)** | **Ganesan et al. (2021)** | **García et al. (2022)** | **Gavish et al. (2022) (1)** |
|  | **20/06/22** | **20/06/22** | **21-06-22** | **21-06-22** | **22-06-22** |
| 1. Were the criteria for inclusion in the sample clearly defined? | No | Yes | No | Yes | Yes |
| 2. Were the study subjects and setting described in detail? | No | Unclear | No | Unclear | Unclear |
| 3. Was the exposure measured in a valid and reliable way? | Yes | Yes | Yes | Yes | Yes |
| 4. Were objective and standard criteria used to measure the condition? | Yes | Yes | Yes | Yes | Yes |
| 5. Were confounding factors identified? | Yes | Yes | Yes | Yes | Yes |
| 6. Were strategies established to deal with confounding factors? | Yes | Yes | Yes | Yes | Yes |
| 7. Were the outcomes measured in a valid and reliable way? | Yes | Yes | Yes | Yes | Yes |
| 8. Was an appropriate statistical analysis used? | Yes | Yes | Yes | Yes | Yes |
| **Conclusion** | High risk | Low risk | High risk | Low risk | Low risk |
| **Justification of the assigned risk** | This study lacks many methodological details | - | This study lacks many methodological details | - | - |
|  |  |  |  |  |  |
|  |  |  |  |  |  |
| **Domains** | **Gavish et al. (2022) (2)** | **Genari et al. (2022)** | **Ghafari et al. (2022)** | **Ghosh et al. (2020)** | **Giacopelli (2020)** |
|  | **22-06-22** | **25-06-22** | **25-06-22** | **25-06-22** | **25-06-22** |
| 1. Were the criteria for inclusion in the sample clearly defined? | Yes | Yes | No | Yes | No |
| 2. Were the study subjects and setting described in detail? | Unclear | Unclear | No | Unclear | No |
| 3. Was the exposure measured in a valid and reliable way? | Yes | Yes | Yes | Yes | Unclear |
| 4. Were objective and standard criteria used to measure the condition? | Yes | Yes | Yes | Yes | Yes |
| 5. Were confounding factors identified? | Yes | Yes | Yes | Yes | Unclear |
| 6. Were strategies established to deal with confounding factors? | Yes | Yes | Yes | Yes | Unclear |
| 7. Were the outcomes measured in a valid and reliable way? | Yes | Yes | Yes | Yes | Yes |
| 8. Was an appropriate statistical analysis used? | Yes | Yes | Yes | Yes | Yes |
| **Conclusion** | Low risk | Low risk | High risk | Low risk | High risk |
| **Justification of the assigned risk** | - | - | This study lacks many methodological details, especially from the population | - | This study lacks many methodological details |
|  |  |  |  |  |  |
|  |  |  |  |  |  |
| **Domains** | **Di Giamberardino et al. (2021)** | **Giardina et al. (2022)** | **Glover et al. (2020)** | **Glover et al. (2021)** | **Goldstein et al. (2021)** |
|  | **25-06-22** | **25-06-22** | **25-06-22** | **25-06-22** | **25-06-22** |
| 1. Were the criteria for inclusion in the sample clearly defined? | No | Yes | Yes | Yes | Yes |
| 2. Were the study subjects and setting described in detail? | No | Yes | Unclear | Unclear | Unclear |
| 3. Was the exposure measured in a valid and reliable way? | Yes | Yes | Yes | Yes | Yes |
| 4. Were objective and standard criteria used to measure the condition? | Yes | Yes | Yes | Yes | Yes |
| 5. Were confounding factors identified? | Yes | Unclear | Yes | Yes | Yes |
| 6. Were strategies established to deal with confounding factors? | Yes | Yes | Yes | Yes | Yes |
| 7. Were the outcomes measured in a valid and reliable way? | Yes | Yes | Yes | Yes | Yes |
| 8. Was an appropriate statistical analysis used? | Yes | Yes | Yes | Yes | Yes |
| **Conclusion** | High risk | Low risk | Low risk | Low risk | Low risk |
| **Justification of the assigned risk** | This study has no data related to the origin of the database | - | - | - | - |
|  |  |  |  |  |  |
|  |  |  |  |  |  |
| **Domains** | **Gomes et al. (2022)** | **González-Parra et al. (2021)** | **Martínez-Rodríguez et al. (2021)** | **Gonzalez-Parra et al. (2022)** | **Good et al. (2020)** |
|  | **25-06-22** | **25-06-22** | **25-06-22** | **25-06-22** | **25-06-22** |
| 1. Were the criteria for inclusion in the sample clearly defined? | Yes | No | No | No | No |
| 2. Were the study subjects and setting described in detail? | Unclear | No | No | No | No |
| 3. Was the exposure measured in a valid and reliable way? | Yes | Yes | Yes | Yes | Yes |
| 4. Were objective and standard criteria used to measure the condition? | Yes | Yes | Yes | Yes | Yes |
| 5. Were confounding factors identified? | Yes | Unclear | Unclear | Unclear | Yes |
| 6. Were strategies established to deal with confounding factors? | Yes | Yes | Yes | Yes | Yes |
| 7. Were the outcomes measured in a valid and reliable way? | Yes | Yes | Yes | Yes | Yes |
| 8. Was an appropriate statistical analysis used? | Yes | Yes | Yes | Yes | Yes |
| **Conclusion** | Low risk | High risk | High risk | High risk | High risk |
| **Justification of the assigned risk** | - | Provenance data not adequately described. They are simulations | Provenance data not adequately described. They are simulations | Provenance data not adequately described | This study has no data related to the origin of the database |
|  |  |  |  |  |  |
|  |  |  |  |  |  |
| **Domains** | **Gozzi et al. (2021)** | **Gozzi et al. (2022)** | **Guerstein et al. (2020)** | **Gumel et al. (2021)** | **Gutiérrez-Jara et al. (2022)** |
|  | **25-06-22** | **25-06-22** | **25-06-22** | **25-06-22** | **25-06-22** |
| 1. Were the criteria for inclusion in the sample clearly defined? | Yes | Yes | No | Yes | No |
| 2. Were the study subjects and setting described in detail? | Unclear | Unclear | No | Unclear | No |
| 3. Was the exposure measured in a valid and reliable way? | Yes | Yes | Yes | Yes | Yes |
| 4. Were objective and standard criteria used to measure the condition? | Yes | Yes | Yes | Yes | Yes |
| 5. Were confounding factors identified? | Yes | Yes | Yes | Yes | Yes |
| 6. Were strategies established to deal with confounding factors? | Yes | Yes | Yes | Yes | Yes |
| 7. Were the outcomes measured in a valid and reliable way? | Yes | Yes | Yes | Yes | Yes |
| 8. Was an appropriate statistical analysis used? | Yes | Yes | Yes | Yes | Yes |
| **Conclusion** | Low risk | Low risk | High risk | Low risk | High risk |
| **Justification of the assigned risk** | - | - | This study has no data related to the origin of the database | - | This study has no data related to the origin of the database |
|  |  |  |  |  |  |
| **Domains** | **Guzmán-Merino et al. (2021)** | **Hagens et al. (2021)** | **Hammouni et al. (2021)** | **Han et al. (2021)** | **Hanly et al. (2022)** |
|  | **25-06-22** | **25-06-22** | **25-06-22** | **25-06-22** | **25-06-22** |
| 1. Were the criteria for inclusion in the sample clearly defined? | Yes | Yes | Yes | Yes | Yes |
| 2. Were the study subjects and setting described in detail? | Unclear | Unclear | Unclear | Unclear | Unclear |
| 3. Was the exposure measured in a valid and reliable way? | Yes | Yes | Yes | Yes | Yes |
| 4. Were objective and standard criteria used to measure the condition? | Yes | Yes | Yes | Yes | Yes |
| 5. Were confounding factors identified? | Yes | Yes | Yes | Yes | Yes |
| 6. Were strategies established to deal with confounding factors? | Yes | Yes | Yes | Yes | Yes |
| 7. Were the outcomes measured in a valid and reliable way? | Yes | Yes | Yes | Yes | Yes |
| 8. Was an appropriate statistical analysis used? | Yes | Yes | Yes | Yes | Yes |
| **Conclusion** | Low risk | Low risk | Low risk | Low risk | Low risk |
| **Justification of the assigned risk** | - | - | - | - | - |
|  |  |  |  |  |  |
|  |  |  |  |  |  |
| **Domains** | **Hartnertt et al. (2021)** | **Hawkes et al. (2022)** | **Hinch et al. (2021)** | **Hjorleifsson et al. (2022)** | **Hladish et al. (2022)** |
|  | **25-06-22** | **25-06-22** | **25-06-22** | **25-06-22** | **26-06-22** |
| 1. Were the criteria for inclusion in the sample clearly defined? | No | Yes | Yes | No | No |
| 2. Were the study subjects and setting described in detail? | No | Unclear | Unclear | No | No |
| 3. Was the exposure measured in a valid and reliable way? | Yes | Yes | Yes | Yes | Yes |
| 4. Were objective and standard criteria used to measure the condition? | Yes | Yes | Yes | Yes | Yes |
| 5. Were confounding factors identified? | Yes | Yes | Yes | Yes | Yes |
| 6. Were strategies established to deal with confounding factors? | Yes | Yes | Yes | Yes | Yes |
| 7. Were the outcomes measured in a valid and reliable way? | Yes | Yes | Yes | Yes | Yes |
| 8. Was an appropriate statistical analysis used? | Yes | Yes | Yes | Yes | Yes |
| **Conclusion** | High risk | Low risk | Low risk | High risk | High risk |
| **Justification of the assigned risk** | This study has no data related to the origin of the database | - | - | This study has no data related to the origin of the database | This study has no data related to the origin of the database |
|  |  |  |  |  |  |
| **Domains** | **Hoertel et al. (2021)** | **Hogan et al. (2021)** | **Holmdahl et al. (2021)** | **Huang et al. (2021)** | **Hupert et al. (2021)** |
|  | **26-06-22** | **26-06-22** | **26-06-22** | **26-06-22** | **26-06-22** |
| 1. Were the criteria for inclusion in the sample clearly defined? | Unclear | Yes | Yes | Yes | Yes |
| 2. Were the study subjects and setting described in detail? | Unclear | Unclear | Yes | Unclear | No |
| 3. Was the exposure measured in a valid and reliable way? | Yes | Yes | Yes | Yes | Yes |
| 4. Were objective and standard criteria used to measure the condition? | Yes | Yes | Yes | Yes | Yes |
| 5. Were confounding factors identified? | Yes | Yes | Yes | Yes | Yes |
| 6. Were strategies established to deal with confounding factors? | Yes | Yes | Yes | Yes | Yes |
| 7. Were the outcomes measured in a valid and reliable way? | Yes | Yes | Yes | Yes | Yes |
| 8. Was an appropriate statistical analysis used? | Yes | Yes | Yes | Yes | Yes |
| **Conclusion** | High risk | Low risk | Low risk | Low risk | High risk |
| **Justification of the assigned risk** | This study presents the origin of the data, but without any details | - | - | - | This study presents the origin of the data, but without any details |
|  |  |  |  |  |  |
| **Domains** | **Iboi et al. (2020)** | **Jablonska et al. (2021)** | **Jayasundara et al. (2021)** | **Jentsch et al. (2021)** | **Jiménez-Rodríguez et al. (2021)** |
|  | **01-07-22** | **01-07-22** | **01-07-22** | **01-07-22** | **01-07-22** |
| 1. Were the criteria for inclusion in the sample clearly defined? | Unclear | Yes | Yes | Yes | Yes |
| 2. Were the study subjects and setting described in detail? | No | Unclear | Unclear | Unclear | Unclear |
| 3. Was the exposure measured in a valid and reliable way? | Yes | Yes | Yes | Yes | Yes |
| 4. Were objective and standard criteria used to measure the condition? | Yes | Yes | Yes | Yes | Yes |
| 5. Were confounding factors identified? | Yes | Yes | Yes | Yes | Yes |
| 6. Were strategies established to deal with confounding factors? | Yes | Yes | Yes | Yes | Yes |
| 7. Were the outcomes measured in a valid and reliable way? | Yes | Yes | Yes | Yes | Yes |
| 8. Was an appropriate statistical analysis used? | Yes | Yes | Yes | Yes | Yes |
| **Conclusion** | High risk | Low risk | Low risk | Low risk | Low risk |
| **Justification of the assigned risk** | There is not enough information about the population | - | - | - | - |
|  |  |  |  |  |  |
|  |  |  |  |  |  |
| **Domains** | **Kahn et al. (2022)** | **Karabay et al. (2021) (1)** | **Karabay et al. (2021) (2)** | **Kassa et al. (2021)** | **Keeling et al. (2021)** |
|  | **01-07-22** | **01-07-22** | **01-07-22** | **01-07-22** | **01-07-22** |
| 1. Were the criteria for inclusion in the sample clearly defined? | Yes | Yes | Yes | Yes | Yes |
| 2. Were the study subjects and setting described in detail? | Yes | Unclear | Unclear | Unclear | Yes |
| 3. Was the exposure measured in a valid and reliable way? | Yes | Yes | Yes | Yes | Yes |
| 4. Were objective and standard criteria used to measure the condition? | Yes | Yes | Yes | Yes | Yes |
| 5. Were confounding factors identified? | Yes | Yes | Yes | Yes | Yes |
| 6. Were strategies established to deal with confounding factors? | Yes | Yes | Yes | Yes | Yes |
| 7. Were the outcomes measured in a valid and reliable way? | Yes | Yes | Yes | Yes | Yes |
| 8. Was an appropriate statistical analysis used? | Yes | Yes | Yes | Yes | Yes |
| **Conclusion** | Low risk | Low risk | Low risk | Low risk | Low risk |
| **Justification of the assigned risk** | - | - | - | - | - |
|  |  |  |  |  |  |
| **Domains** | **Keeling et al. (2022)** | **Kemp et al. (2021)** | **Kerr et al. (2021)** | **Khan et al. (2021)** | **Kim et al. (2021)** |
|  | **01-07-22** | **02-07-22** | **02-07-22** | **02-07-22** | **02-07-22** |
| 1. Were the criteria for inclusion in the sample clearly defined? | Yes | Yes | Yes | Yes | Yes |
| 2. Were the study subjects and setting described in detail? | Yes | Unclear | Yes | Unclear | Unclear |
| 3. Was the exposure measured in a valid and reliable way? | Yes | Yes | Yes | Yes | Yes |
| 4. Were objective and standard criteria used to measure the condition? | Yes | Yes | Yes | Yes | Yes |
| 5. Were confounding factors identified? | Yes | Yes | Yes | Yes | Yes |
| 6. Were strategies established to deal with confounding factors? | Yes | Yes | Yes | Yes | Yes |
| 7. Were the outcomes measured in a valid and reliable way? | Yes | Yes | Yes | Yes | Yes |
| 8. Was an appropriate statistical analysis used? | Yes | Yes | Yes | Yes | Yes |
| **Conclusion** | Low risk | Low risk | Low risk | Low risk | Low risk |
| **Justification of the assigned risk** | - | - | - | - | - |
|  |  |  |  |  |  |
| **Domains** | **Kirwin et al. (2021)** | **Kitano et al. (2021)** | **Ko et al. (2021)** | **Ko et al. (2022)** | **Kou et al. (2021)** |
|  | **02-07-22** | **02-07-22** | **02-07-22** | **02-07-22** | **02-07-22** |
| 1. Were the criteria for inclusion in the sample clearly defined? | Yes | Yes | Yes | Unclear | Yes |
| 2. Were the study subjects and setting described in detail? | Unclear | Yes | Unclear | Unclear | Unclear |
| 3. Was the exposure measured in a valid and reliable way? | Yes | Yes | Yes | Yes | Yes |
| 4. Were objective and standard criteria used to measure the condition? | Yes | Yes | Yes | Yes | Yes |
| 5. Were confounding factors identified? | Yes | Yes | Yes | Unclear | Yes |
| 6. Were strategies established to deal with confounding factors? | Yes | Yes | Yes | Yes | Yes |
| 7. Were the outcomes measured in a valid and reliable way? | Yes | Yes | Yes | Yes | Yes |
| 8. Was an appropriate statistical analysis used? | Yes | Yes | Yes | Yes | Yes |
| **Conclusion** | Low risk | Low risk | Low risk | High risk | Low risk |
| **Justification of the assigned risk** | - | - | - | The study is not clear in methodological aspects | - |
|  |  |  |  |  |  |
| **Domains** | **Kraay et al. (2021)** | **Lasser et al. (2022)** | **Lau et al. (2021)** | **Layton et al. (2022)** | **Lee et al. (2021)** |
|  | **02-07-22** | **02-07-22** | **02-07-22** | **02-07-22** | **02-07-22** |
| 1. Were the criteria for inclusion in the sample clearly defined? | Unclear | Yes | Yes | Unclear | Yes |
| 2. Were the study subjects and setting described in detail? | Unclear | Yes | Unclear | Unclear | Unclear |
| 3. Was the exposure measured in a valid and reliable way? | Yes | Yes | Yes | Yes | Yes |
| 4. Were objective and standard criteria used to measure the condition? | Yes | Yes | Yes | Yes | Yes |
| 5. Were confounding factors identified? | Unclear | Unclear | Yes | Unclear | Yes |
| 6. Were strategies established to deal with confounding factors? | Yes | Yes | Yes | Yes | Yes |
| 7. Were the outcomes measured in a valid and reliable way? | Yes | Yes | Yes | Yes | Yes |
| 8. Was an appropriate statistical analysis used? | Yes | Yes | Yes | Yes | Yes |
| **Conclusion** | High risk | Low risk | Low risk | High risk | Low risk |
| **Justification of the assigned risk** | The study is not clear in methodological aspects | - | - | The study is not clear in methodological aspects | - |
|  |  |  |  |  |  |
|  |  |  |  |  |  |
| **Domains** | **Lemaitre et al. (2021)** | **Leung et al. (2021)** | **Li et al. (2021) (1)** | **Li et al. (2021) (2)** | **Li et al. (2021) (3)** |
|  | **02-07-22** | **02-07-22** | **02-07-22** | **02-07-22** | **02-07-22** |
| 1. Were the criteria for inclusion in the sample clearly defined? | Yes | Unclear | Unclear | Unclear | Yes |
| 2. Were the study subjects and setting described in detail? | Unclear | Unclear | Unclear | Unclear | Unclear |
| 3. Was the exposure measured in a valid and reliable way? | Yes | Yes | Yes | Yes | Yes |
| 4. Were objective and standard criteria used to measure the condition? | Yes | Yes | Yes | Yes | Yes |
| 5. Were confounding factors identified? | Yes | Unclear | Unclear | Unclear | Yes |
| 6. Were strategies established to deal with confounding factors? | Yes | Yes | Yes | Yes | Yes |
| 7. Were the outcomes measured in a valid and reliable way? | Yes | Yes | Unclear | Unclear | Yes |
| 8. Was an appropriate statistical analysis used? | Yes | Yes | Yes | Yes | Yes |
| **Conclusion** | Low risk | High risk | High risk | High risk | Low risk |
| **Justification of the assigned risk** | - | The study is not clear in methodological aspects | The study is not clear in methodological aspects, the confounding variables as such are not defined | The study is not clear in methodological aspects, the confounding variables as such are not defined | - |
|  |  |  |  |  |  |
| **Domains** | **Li et al. (2021) (4)** | **Li et al. (2021) (5)** | **Li et al. (2021) (6)** | **Li et al. (2022)** | **Li et al. (2021) (7)** |
|  | **02-07-22** | **02-07-22** | **02-07-22** | **02-07-22** | **02-07-22** |
| 1. Were the criteria for inclusion in the sample clearly defined? | Yes | Yes | Yes | Yes | Yes |
| 2. Were the study subjects and setting described in detail? | Unclear | Unclear | Unclear | Unclear | Unclear |
| 3. Was the exposure measured in a valid and reliable way? | Yes | Yes | Yes | Yes | Yes |
| 4. Were objective and standard criteria used to measure the condition? | Yes | Yes | Yes | Yes | Yes |
| 5. Were confounding factors identified? | Yes | Yes | Yes | Yes | Yes |
| 6. Were strategies established to deal with confounding factors? | Yes | Yes | Yes | Yes | Yes |
| 7. Were the outcomes measured in a valid and reliable way? | Yes | Yes | Yes | Yes | Yes |
| 8. Was an appropriate statistical analysis used? | Yes | Yes | Yes | Yes | Yes |
| **Conclusion** | Low risk | Low risk | Low risk | Low risk | Low risk |
| **Justification of the assigned risk** | - | - | - | - | - |
|  |  |  |  |  |  |
| **Domains** | **Lin et al. (2022) (1)** | **Lin et al. (2022) (2)** | **Liu et al. (2022) (1)** | **Liu et al. (2022) (2)** | **Liu et al. (2022) (3)** |
|  | **08-07-22** | **08-07-22** | **08-07-22** | **08-07-22** | **08-07-22** |
| 1. Were the criteria for inclusion in the sample clearly defined? | Yes | Yes | Yes | Yes | Yes |
| 2. Were the study subjects and setting described in detail? | Unclear | Unclear | Unclear | Unclear | Unclear |
| 3. Was the exposure measured in a valid and reliable way? | Yes | Yes | Yes | Yes | Yes |
| 4. Were objective and standard criteria used to measure the condition? | Yes | Yes | Yes | Yes | Yes |
| 5. Were confounding factors identified? | Yes | Yes | Yes | Yes | Yes |
| 6. Were strategies established to deal with confounding factors? | Yes | Yes | Yes | Yes | Yes |
| 7. Were the outcomes measured in a valid and reliable way? | Yes | Yes | Yes | Yes | Yes |
| 8. Was an appropriate statistical analysis used? | Yes | Yes | Yes | Yes | Yes |
| **Conclusion** | Low risk | Low risk | Low risk | Low risk | Low risk |
| **Justification of the assigned risk** | - | - | - | - | - |
|  |  |  |  |  |  |
|  |  |  |  |  |  |
| **Domains** | **Luo et al. (2021)** | **Machado et al. (2022)** | **Maier et al. (2021)** | **Mairanowski et al. (2021) (1)** | **Mairanowski et al. (2021) (2)** |
|  | **08-07-22** | **08-07-22** | **08-07-22** | **08-07-22** | **08-07-22** |
| 1. Were the criteria for inclusion in the sample clearly defined? | No | Yes | Yes | No | No |
| 2. Were the study subjects and setting described in detail? | No | Unclear | Unclear | No | No |
| 3. Was the exposure measured in a valid and reliable way? | Yes | Yes | Yes | Yes | Yes |
| 4. Were objective and standard criteria used to measure the condition? | Yes | Yes | Yes | Yes | Yes |
| 5. Were confounding factors identified? | Yes | Yes | Yes | Yes | Yes |
| 6. Were strategies established to deal with confounding factors? | Yes | Yes | Yes | Yes | Yes |
| 7. Were the outcomes measured in a valid and reliable way? | Yes | Yes | Yes | Yes | Yes |
| 8. Was an appropriate statistical analysis used? | Yes | Yes | Yes | Yes | Yes |
| **Conclusion** | High risk | Low risk | Low risk | High risk | High risk |
| **Justification of the assigned risk** | This study has no data related to the origin of the database | - | - | This study has no data related to the origin of the database | This study has no data related to the origin of the database |
|  |  |  |  |  |  |
|  |  |  |  |  |  |
| **Domains** | **Majumder et al. (2022)** | **Makhoul et al. (2020)** | **Makhoul et al. (2021)** | **Mallela et al. (2022)** | **Mandal et al. (2021) (1)** |
|  | **08-07-22** | **08-07-22** | **08-07-22** | **08-07-22** | **08-07-22** |
| 1. Were the criteria for inclusion in the sample clearly defined? | No | Yes | Yes | Yes | Yes |
| 2. Were the study subjects and setting described in detail? | No | Unclear | Unclear | Unclear | Unclear |
| 3. Was the exposure measured in a valid and reliable way? | Yes | Yes | Yes | Yes | Yes |
| 4. Were objective and standard criteria used to measure the condition? | Yes | Yes | Yes | Yes | Yes |
| 5. Were confounding factors identified? | Yes | Yes | Yes | Yes | Yes |
| 6. Were strategies established to deal with confounding factors? | Yes | Yes | Yes | Yes | Yes |
| 7. Were the outcomes measured in a valid and reliable way? | Yes | Yes | Yes | Yes | Yes |
| 8. Was an appropriate statistical analysis used? | Yes | Yes | Yes | Yes | Yes |
| **Conclusion** | High risk | Low risk | Low risk | Low risk | Low risk |
| **Justification of the assigned risk** | This study has no data related to the origin of the database | - | - | - | - |
|  |  |  |  |  |  |
|  |  |  |  |  |  |
| **Domains** | **Mandal et al. (2021) (2)** | **Marziano et al. (2021)** | **Massonnaud et al. (2021)** | **Mathiot et al. (2021)** | **Matrajt et al. (2020)** |
|  | **08-07-22** | **08-07-22** | **08-07-22** | **08-07-22** | **08-07-22** |
| 1. Were the criteria for inclusion in the sample clearly defined? | Yes | No | Unclear | Yes | Yes |
| 2. Were the study subjects and setting described in detail? | Unclear | No | Unclear | Unclear | Unclear |
| 3. Was the exposure measured in a valid and reliable way? | Yes | Yes | Yes | Yes | Yes |
| 4. Were objective and standard criteria used to measure the condition? | Yes | Yes | Yes | Yes | Yes |
| 5. Were confounding factors identified? | Yes | Yes | Yes | Yes | Yes |
| 6. Were strategies established to deal with confounding factors? | Yes | Yes | Yes | Yes | Yes |
| 7. Were the outcomes measured in a valid and reliable way? | Yes | Yes | Yes | Yes | Yes |
| 8. Was an appropriate statistical analysis used? | Yes | Yes | Yes | Yes | Yes |
| **Conclusion** | Low risk | High risk | Unclear | Low risk | Low risk |
| **Justification of the assigned risk** | - | This study has no data related to the origin of the database | Details of the subjects included are unclear | - | - |
|  |  |  |  |  |  |
|  |  |  |  |  |  |
| **Domains** | **Matrajt et al. (2021)** | **Matrajt et al. (2022)** | **McBryde et al. (2021)** | **Milne et al. (2022)** | **Min et al. (2021)** |
|  | **08-07-22** | **08-07-22** | **08-07-22** | **08-07-22** | **08-07-22** |
| 1. Were the criteria for inclusion in the sample clearly defined? | Yes | Yes | Yes | Yes | Yes |
| 2. Were the study subjects and setting described in detail? | Unclear | Unclear | Unclear | No | No |
| 3. Was the exposure measured in a valid and reliable way? | Yes | Yes | Yes | Yes | Yes |
| 4. Were objective and standard criteria used to measure the condition? | Yes | Yes | Yes | Yes | Yes |
| 5. Were confounding factors identified? | Yes | Yes | Yes | Yes | Yes |
| 6. Were strategies established to deal with confounding factors? | Yes | Yes | Yes | Yes | Yes |
| 7. Were the outcomes measured in a valid and reliable way? | Yes | Yes | Yes | Yes | Yes |
| 8. Was an appropriate statistical analysis used? | Yes | Yes | Yes | Yes | Yes |
| **Conclusion** | Low risk | Low risk | Low risk | Low risk | Low risk |
| **Justification of the assigned risk** | - | - | - | - | - |
|  |  |  |  |  |  |
|  |  |  |  |  |  |
| **Domains** | **Miura et al. (2021)** | **Moghadas et al. (2021) (1)** | **Moghadas et al. (2021) (2)** | **Moghadas et al. (2021) (3)** | **Moldokmatova et al. (2021)** |
|  | **08-07-22** | **08-07-22** | **08-07-22** | **08-07-22** | **08-07-22** |
| 1. Were the criteria for inclusion in the sample clearly defined? | Yes | Unclear | Yes | Yes | Yes |
| 2. Were the study subjects and setting described in detail? | No | Unclear | No | No | No |
| 3. Was the exposure measured in a valid and reliable way? | Yes | Yes | Yes | Yes | Yes |
| 4. Were objective and standard criteria used to measure the condition? | Yes | Yes | Yes | Yes | Yes |
| 5. Were confounding factors identified? | Yes | Yes | Yes | Yes | Yes |
| 6. Were strategies established to deal with confounding factors? | Yes | Yes | Yes | Yes | Yes |
| 7. Were the outcomes measured in a valid and reliable way? | Yes | Yes | Yes | Yes | Yes |
| 8. Was an appropriate statistical analysis used? | Yes | Yes | Yes | Yes | Yes |
| **Conclusion** | Low risk | Unclear | Low risk | Low risk | Low risk |
| **Justification of the assigned risk** | - | Details of the subjects included are unclear | - | - | - |
|  |  |  |  |  |  |
|  |  |  |  |  |  |
| **Domains** | **Moore et al. (2021)** | **Moore et al. (2021)** | **Morales-Zamora et al. (2022)** | **Mukandavire et al. (2020)** | **Mumtaz et al. (2021)** |
|  | **09-07-22** | **09-07-22** | **09-07-22** | **09-07-22** | **09-07-22** |
| 1. Were the criteria for inclusion in the sample clearly defined? | Yes | Yes | Yes | Unclear | Unclear |
| 2. Were the study subjects and setting described in detail? | Yes | Unclear | Unclear | Unclear | Unclear |
| 3. Was the exposure measured in a valid and reliable way? | Yes | Yes | Yes | Yes | Yes |
| 4. Were objective and standard criteria used to measure the condition? | Yes | Yes | Yes | Yes | Yes |
| 5. Were confounding factors identified? | Yes | Yes | Yes | Yes | Yes |
| 6. Were strategies established to deal with confounding factors? | Yes | Yes | Yes | Yes | Yes |
| 7. Were the outcomes measured in a valid and reliable way? | Yes | Yes | Yes | Yes | Yes |
| 8. Was an appropriate statistical analysis used? | Yes | Yes | Yes | Yes | Yes |
| **Conclusion** | Low risk | Low risk | Low risk | Unclear | Unclear |
| **Justification of the assigned risk** | - | - | - | Details of the subjects included are unclear | Details of the subjects included are unclear. Although they mention that a cohort is established |
|  |  |  |  |  |  |
|  |  |  |  |  |  |
| **Domains** | **Musa et al. (2020)** | **Rabiu et al. (2021)** | **Nakhaeizadeh et al. (2022)** | **Nam et al. (2021)** | **Nichita et al. (2021)** |
|  | **09-07-22** | **09-07-22** | **09-07-22** | **09-07-22** | **09-07-22** |
| 1. Were the criteria for inclusion in the sample clearly defined? | Yes | Yes | No | Yes | Yes |
| 2. Were the study subjects and setting described in detail? | Unclear | Unclear | Unclear | Unclear | Unclear |
| 3. Was the exposure measured in a valid and reliable way? | Yes | Yes | Yes | Yes | Yes |
| 4. Were objective and standard criteria used to measure the condition? | Yes | Yes | Yes | Yes | Yes |
| 5. Were confounding factors identified? | Yes | Yes | Yes | Yes | Yes |
| 6. Were strategies established to deal with confounding factors? | Yes | Yes | Yes | Yes | Yes |
| 7. Were the outcomes measured in a valid and reliable way? | Yes | Yes | Yes | Yes | Yes |
| 8. Was an appropriate statistical analysis used? | Yes | Yes | Yes | Yes | Yes |
| **Conclusion** | Low risk | Low risk | High risk | Low risk | Low risk |
| **Justification of the assigned risk** | - | - | This study does not present specific data on the origin of the data | - | - |
|  |  |  |  |  |  |
|  |  |  |  |  |  |
| **Domains** | **Nixon et al. (2021)** | **Nuraini et al. (2021)** | **Olmedo et al. (2022)** | **Oloniiju et al. (2022)** | **Omae et al. (2022) (1)** |
|  | **09-07-22** | **09-07-22** | **09-07-22** | **09-07-22** | **09-07-22** |
| 1. Were the criteria for inclusion in the sample clearly defined? | Yes | Yes | Unclear | Yes | Yes |
| 2. Were the study subjects and setting described in detail? | Unclear | Unclear | Unclear | Unclear | Unclear |
| 3. Was the exposure measured in a valid and reliable way? | Yes | Yes | Yes | Yes | Yes |
| 4. Were objective and standard criteria used to measure the condition? | Yes | Yes | Yes | Yes | Yes |
| 5. Were confounding factors identified? | Yes | Yes | Yes | Yes | Yes |
| 6. Were strategies established to deal with confounding factors? | Yes | Yes | Yes | Yes | Yes |
| 7. Were the outcomes measured in a valid and reliable way? | Yes | Yes | Yes | Yes | Yes |
| 8. Was an appropriate statistical analysis used? | Yes | Yes | Yes | Yes | Yes |
| **Conclusion** | Low risk | Low risk | Unclear | Low risk | Low risk |
| **Justification of the assigned risk** | - | - | Details of the subjects included are unclear. Although they mention that a cohort is established | - | - |
|  |  |  |  |  |  |
|  |  |  |  |  |  |
| **Domains** | **Omae et al. (2022) (2)** | **Omar et al. (2021)** | **Omar et al. (2022)** | **Padula et al. (2021)** | **Pae (2021)** |
|  | **09-07-22** | **09-07-22** | **09-07-22** | **09-07-22** | **09-07-22** |
| 1. Were the criteria for inclusion in the sample clearly defined? | Yes | Yes | Yes | Yes | No |
| 2. Were the study subjects and setting described in detail? | Unclear | Unclear | Unclear | Unclear | Unclear |
| 3. Was the exposure measured in a valid and reliable way? | Yes | Yes | Yes | Yes | Yes |
| 4. Were objective and standard criteria used to measure the condition? | Yes | Yes | Yes | Yes | Unclear |
| 5. Were confounding factors identified? | Yes | Yes | Yes | Yes | Yes |
| 6. Were strategies established to deal with confounding factors? | Yes | Yes | Yes | Yes | Yes |
| 7. Were the outcomes measured in a valid and reliable way? | Yes | Yes | Yes | Yes | Yes |
| 8. Was an appropriate statistical analysis used? | Yes | Yes | Yes | Yes | Yes |
| **Conclusion** | Low risk | Low risk | Low risk | Low risk | High risk |
| **Justification of the assigned risk** | - | - | - | - | There are no specific data on the selection criteria of the population included, however, the origin of the database is mentioned |
|  |  |  |  |  |  |
|  |  |  |  |  |  |
| **Domains** | **Pageaud et al. (2021)** | **Paltiel et al. (2021)** | **Pan et al. (2022)** | **Parino et al. (2021)** | **Parolini et al. (2022)** |
|  | **09-07-22** | **09-07-22** | **09-07-22** | **09-07-22** | **09-07-22** |
| 1. Were the criteria for inclusion in the sample clearly defined? | Yes | Yes | Yes | Unclear | Unclear |
| 2. Were the study subjects and setting described in detail? | Unclear | No | No | Unclear | Unclear |
| 3. Was the exposure measured in a valid and reliable way? | Yes | Yes | Yes | Yes | Yes |
| 4. Were objective and standard criteria used to measure the condition? | Yes | Yes | Yes | Yes | Yes |
| 5. Were confounding factors identified? | Yes | Yes | Yes | Yes | Yes |
| 6. Were strategies established to deal with confounding factors? | Yes | Yes | Yes | Yes | Yes |
| 7. Were the outcomes measured in a valid and reliable way? | Yes | Yes | Yes | Yes | Yes |
| 8. Was an appropriate statistical analysis used? | Yes | Yes | Yes | Yes | Yes |
| **Conclusion** | Low risk | Low risk | Low risk | Unclear | Unclear |
| **Justification of the assigned risk** | - | - | - | Details of the subjects included are unclear. Although they mention that a cohort is established | Details of the subjects included are unclear. Although they mention that a cohort is established |
|  |  |  |  |  |  |
| **Domains** | **Patel et al. (2021)** | **Pearson et al. (2021)** | **Pérez et al. (2021)** | **Rachaniotis et al. (2021)** | **Rahmandan et al. (2021)** |
|  | **09-07-22** | **16-07-22** | **16-07-22** | **16-07-22** | **16-07-22** |
| 1. Were the criteria for inclusion in the sample clearly defined? | No | No | Yes | Unclear | Yes |
| 2. Were the study subjects and setting described in detail? | No | No | No | Unclear | Unclear |
| 3. Was the exposure measured in a valid and reliable way? | Yes | Yes | Yes | Yes | Yes |
| 4. Were objective and standard criteria used to measure the condition? | Yes | Yes | Yes | Yes | Yes |
| 5. Were confounding factors identified? | Unclear | Unclear | Yes | Yes | Yes |
| 6. Were strategies established to deal with confounding factors? | Yes | Yes | Yes | Yes | Yes |
| 7. Were the outcomes measured in a valid and reliable way? | Yes | Yes | Yes | Yes | Yes |
| 8. Was an appropriate statistical analysis used? | Yes | Yes | Yes | Yes | Yes |
| **Conclusion** | High risk | High risk | Low risk | Low risk | Low risk |
| **Justification of the assigned risk** | There are no established selection criteria for the inclusion of patients in the study. A database is taken as a base. At the same time, there are no evidence of possible effects that cause confusion | There are no established selection criteria for the inclusion of patients in the study. A database is taken as a base. At the same time, there are no evidence of possible effects that cause confusion | - | Although there are no clear criteria, the simulation was done based on a specific population. So it is considered low risk. | Although there are no clear criteria, the simulation was done based on a specific population. So it is considered low risk. |
|  |  |  |  |  |  |
|  |  |  |  |  |  |
| **Domains** | **Raina et al. (2021)** | **Rajakaruna et al. (2022)** | **Rajapaksha et al. (2021)** | **Rajput et al. (2021)** | **Rana et al. (2022)** |
|  | **16-07-22** | **16-07-22** | **16-07-22** | **16-07-22** | **16-07-22** |
| 1. Were the criteria for inclusion in the sample clearly defined? | Yes | Yes | Yes | Yes | Yes |
| 2. Were the study subjects and setting described in detail? | Unclear | Yes | Unclear | Unclear | Unclear |
| 3. Was the exposure measured in a valid and reliable way? | Yes | Yes | Yes | Yes | Yes |
| 4. Were objective and standard criteria used to measure the condition? | Yes | Yes | Yes | Yes | Yes |
| 5. Were confounding factors identified? | Yes | Yes | Yes | Yes | Yes |
| 6. Were strategies established to deal with confounding factors? | Yes | Yes | Yes | Yes | Yes |
| 7. Were the outcomes measured in a valid and reliable way? | Yes | Yes | Yes | Yes | Yes |
| 8. Was an appropriate statistical analysis used? | Yes | Yes | Yes | Yes | Yes |
| **Conclusion** | Low risk | Low risk | Low risk | Low risk | Low risk |
| **Justification of the assigned risk** | - | - | - | - | - |
|  |  |  |  |  |  |
|  |  |  |  |  |  |
| **Domains** | **Rao et al. (2021)** | **Reddy et al. (2021)** | **Reyné et al. (2022)** | **Robles-Fontán et al. (2020)** | **Rocha et al. (2021)** |
|  | **16-07-22** | **16-07-22** | **16-07-22** | **16-07-22** | **16-07-22** |
| 1. Were the criteria for inclusion in the sample clearly defined? | Yes | No | Unclear | No | Yes |
| 2. Were the study subjects and setting described in detail? | Unclear | Unclear | Unclear | Unclear | Yes |
| 3. Was the exposure measured in a valid and reliable way? | Yes | Yes | Yes | Yes | Yes |
| 4. Were objective and standard criteria used to measure the condition? | Yes | Yes | Yes | Yes | Yes |
| 5. Were confounding factors identified? | Yes | Yes | Yes | Yes | Yes |
| 6. Were strategies established to deal with confounding factors? | Yes | Yes | Yes | Yes | Yes |
| 7. Were the outcomes measured in a valid and reliable way? | Yes | Yes | Yes | Yes | Yes |
| 8. Was an appropriate statistical analysis used? | Yes | Yes | Yes | Yes | Yes |
| **Conclusion** | Low risk | Low risk | Unclear | Low risk | Low risk |
| **Justification of the assigned risk** | - | - | Details of the subjects included are unclear. | - | - |
|  |  |  |  |  |  |
| **Domains** | **Rodríguez et al. (2021)** | **Romero-Brufau et al. (2021)** | **Roy et al. (2021) (1)** | **Roy et al. (2021) (2)** | **Sadarangani et al. (2021)** |
|  | **16-07-22** | **16-07-22** | **16-07-22** | **16-07-22** | **16-07-22** |
| 1. Were the criteria for inclusion in the sample clearly defined? | Unclear | Yes | No | No | Unclear |
| 2. Were the study subjects and setting described in detail? | Yes | No | No | No | No |
| 3. Was the exposure measured in a valid and reliable way? | Yes | Yes | Yes | Yes | Yes |
| 4. Were objective and standard criteria used to measure the condition? | Yes | Yes | Yes | Yes | Yes |
| 5. Were confounding factors identified? | Yes | Unclear | Unclear | Unclear | Yes |
| 6. Were strategies established to deal with confounding factors? | Yes | Unclear | Unclear | Unclear | Yes |
| 7. Were the outcomes measured in a valid and reliable way? | Yes | Yes | Yes | Unclear | Yes |
| 8. Was an appropriate statistical analysis used? | Yes | Yes | Yes | Yes | Yes |
| **Conclusion** | Low risk | High risk | High risk | High risk | High risk |
| **Justification of the assigned risk** | Although the study establishes very well the parameters that were taken into account for the population, its origin is not clear | The criteria to be taken into account in the population were presented, but the conditions for the evaluation of confounding variables were not clear | The data corresponding to the population are not found | There are no methodological data in this study. The criteria that were taken into account for the generation of the compartments are mentioned, but there are no details | Details of the source of the population are not mentioned |
|  |  |  |  |  |  |
| **Domains** | **Sah et al. (2021)** | **Saldaña et al. (2022)** | **Sandmann et al. (2021)** | **Sanz-Leon et al. (2022)** | **Savinkina et al. (2022)** |
|  | **16-07-22** | **16-07-22** | **16-07-22** | **16-07-22** | **16-07-22** |
| 1. Were the criteria for inclusion in the sample clearly defined? | Unclear | Yes | Yes | Yes | Yes |
| 2. Were the study subjects and setting described in detail? | No | Unclear | Unclear | Unclear | Unclear |
| 3. Was the exposure measured in a valid and reliable way? | Yes | Yes | Yes | Yes | Yes |
| 4. Were objective and standard criteria used to measure the condition? | Yes | Yes | Yes | Yes | Yes |
| 5. Were confounding factors identified? | Yes | Yes | Yes | Yes | Yes |
| 6. Were strategies established to deal with confounding factors? | Yes | Yes | Yes | Yes | Yes |
| 7. Were the outcomes measured in a valid and reliable way? | Yes | Yes | Yes | Yes | Yes |
| 8. Was an appropriate statistical analysis used? | Yes | Yes | Yes | Yes | Yes |
| **Conclusion** | Low risk | Low risk | Low risk | Low risk | Low risk |
| **Justification of the assigned risk** | - | - | - | - | - |
|  |  |  |  |  |  |
|  |  |  |  |  |  |
| **Domains** | **Scarabaggio et al. (2021)** | **Schneider et al. (2022)** | **Schulenburg et al. (2022)** | **Shadi et al. (2022)** | **Shen et al. (2021)** |
|  | **16-07-22** | **16-07-22** | **16-07-22** | **16-07-22** | **16-07-22** |
| 1. Were the criteria for inclusion in the sample clearly defined? | Yes | Unclear | No | No | Yes |
| 2. Were the study subjects and setting described in detail? | Yes | Unclear | No | No | Yes |
| 3. Was the exposure measured in a valid and reliable way? | Yes | Yes | Yes | Yes | Yes |
| 4. Were objective and standard criteria used to measure the condition? | Yes | Yes | Yes | Yes | Yes |
| 5. Were confounding factors identified? | Yes | Yes | Yes | Unclear | Yes |
| 6. Were strategies established to deal with confounding factors? | Yes | Yes | Yes | Unclear | Yes |
| 7. Were the outcomes measured in a valid and reliable way? | Yes | Yes | Yes | Unclear | Yes |
| 8. Was an appropriate statistical analysis used? | Yes | Yes | Yes | Yes | Yes |
| **Conclusion** | Low risk | Unclear | High risk | High risk | Low risk |
| **Justification of the assigned risk** | - | Details of the included population are not clear | Details of the included population are not clear | The criteria that were taken into account for the generation of the compartments are mentioned, but there are no details | - |
|  |  |  |  |  |  |
|  |  |  |  |  |  |
| **Domains** | **Shim (2021) (1)** | **Shim (2021) (2)** | **Silva et al. (2021)** | **Castro e Silva et al. (2022)** | **Sivadas et al. (2021)** |
|  | **16-07-22** | **16-07-22** | **16-07-22** | **17-07-22** | **17-07-22** |
| 1. Were the criteria for inclusion in the sample clearly defined? | Yes | Unclear | Unclear | Unclear | Unclear |
| 2. Were the study subjects and setting described in detail? | Yes | Unclear | No | No | No |
| 3. Was the exposure measured in a valid and reliable way? | Yes | Yes | Yes | Yes | Yes |
| 4. Were objective and standard criteria used to measure the condition? | Yes | Yes | Yes | Yes | Yes |
| 5. Were confounding factors identified? | Yes | Yes | Yes | Yes | Yes |
| 6. Were strategies established to deal with confounding factors? | Yes | Yes | Yes | Yes | Yes |
| 7. Were the outcomes measured in a valid and reliable way? | Yes | Yes | Yes | Yes | Yes |
| 8. Was an appropriate statistical analysis used? | Yes | Yes | Yes | Yes | Yes |
| **Conclusion** | Low risk | Unclear | High risk | High risk | High risk |
| **Justification of the assigned risk** | - | Details of the included population are not clear. | Details of the source of the population are not mentioned | Details of the source of the population are not mentioned | Details of the source of the population are not mentioned |
|  |  |  |  |  |  |
|  |  |  |  |  |  |
| **Domains** | **Somekh et al. (2022)** | **Sonabend et al. (2021)** | **Song et al. (2021) (1)** | **Song et al. (2021) (2)** | **Stanojevic et al. (2021)** |
|  | **17-07-22** | **17-07-22** | **17-07-22** | **17-07-22** | **17-07-22** |
| 1. Were the criteria for inclusion in the sample clearly defined? | Yes | Yes | Unclear | Unclear | Yes |
| 2. Were the study subjects and setting described in detail? | Yes | Unclear | No | No | Unclear |
| 3. Was the exposure measured in a valid and reliable way? | Yes | Yes | Yes | Yes | Yes |
| 4. Were objective and standard criteria used to measure the condition? | Yes | Yes | Yes | Yes | Yes |
| 5. Were confounding factors identified? | Yes | Yes | Yes | Yes | Yes |
| 6. Were strategies established to deal with confounding factors? | Yes | Yes | Yes | Yes | Yes |
| 7. Were the outcomes measured in a valid and reliable way? | Yes | Yes | Yes | Yes | Yes |
| 8. Was an appropriate statistical analysis used? | Yes | Yes | Yes | Yes | Yes |
| **Conclusion** | Low risk | Low risk | High risk | High risk | Low risk |
| **Justification of the assigned risk** | - | - | Details of the source of the population are not mentioned | Details of the source of the population are not mentioned | - |
|  |  |  |  |  |  |
|  |  |  |  |  |  |
| **Domains** | **Stapelberg et al. (2021)** | **Stevenson et al. (2021)** | **Steyn et al. (2022)** | **Stollenwerk et al. (2021)** | **Storlie et al. (2021)** |
|  | **17-07-22** | **17-07-22** | **17-07-22** | **17-07-22** | **17-07-22** |
| 1. Were the criteria for inclusion in the sample clearly defined? | Unclear | Yes | Unclear | Yes | Unclear |
| 2. Were the study subjects and setting described in detail? | Unclear | Unclear | Unclear | Unclear | No |
| 3. Was the exposure measured in a valid and reliable way? | Yes | Yes | Yes | Yes | Yes |
| 4. Were objective and standard criteria used to measure the condition? | Yes | Yes | Yes | Yes | Yes |
| 5. Were confounding factors identified? | Yes | Yes | Yes | Yes | Yes |
| 6. Were strategies established to deal with confounding factors? | Yes | Yes | Yes | Yes | Yes |
| 7. Were the outcomes measured in a valid and reliable way? | Yes | Yes | Yes | Yes | Yes |
| 8. Was an appropriate statistical analysis used? | Yes | Yes | Yes | Yes | Yes |
| **Conclusion** | Unclear | Low risk | Unclear | Low risk | High risk |
| **Justification of the assigned risk** | Details of the subjects included are unclear. Although they mention that a cohort is established | - | Details of the subjects included are unclear. Although they mention that a cohort is established | - | Details of the source of the population are not mentioned |
|  |  |  |  |  |  |
|  |  |  |  |  |  |
| **Domains** | **Sulis et al. (2021)** | **Sun et al. (2021)** | **Sunohara et al. (2021)** | **Suphanchaimat et al. (2021) (1)** | **Suphanchaimat et al. (2021) (2)** |
|  | **17-07-22** | **17-07-22** | **17-07-22** | **17-07-22** | **18-07-22** |
| 1. Were the criteria for inclusion in the sample clearly defined? | Unclear | Yes | Unclear | Unclear | Yes |
| 2. Were the study subjects and setting described in detail? | Unclear | Unclear | No | No | Unclear |
| 3. Was the exposure measured in a valid and reliable way? | Yes | Yes | Yes | Yes | Yes |
| 4. Were objective and standard criteria used to measure the condition? | Yes | Yes | Yes | Yes | Yes |
| 5. Were confounding factors identified? | Yes | Yes | Yes | Yes | Yes |
| 6. Were strategies established to deal with confounding factors? | Yes | Yes | Yes | Yes | Yes |
| 7. Were the outcomes measured in a valid and reliable way? | Yes | Yes | Yes | Yes | Yes |
| 8. Was an appropriate statistical analysis used? | Yes | Yes | Yes | Yes | Yes |
| **Conclusion** | Unclear | Low risk | High risk | High risk | Low risk |
| **Justification of the assigned risk** | Details of the subjects included are unclear. Although they mention that a cohort is established | - | Details of the source of the population are not mentioned | Details of the source of the population are not mentioned | - |
|  |  |  |  |  |  |
|  |  |  |  |  |  |
| **Domains** | **Suphanchaimat et al. (2022)** | **Swan et al. (2020)** | **Swan et al. (2021) (1)** | **Swan et al. (2021) (2)** | **Tan et al. (2022)** |
|  | **18-07-22** | **18-07-22** | **18-07-22** | **18-07-22** | **18-07-22** |
| 1. Were the criteria for inclusion in the sample clearly defined? | Yes | Yes | Unclear | Unclear | Yes |
| 2. Were the study subjects and setting described in detail? | Unclear | Unclear | No | No | Yes |
| 3. Was the exposure measured in a valid and reliable way? | Yes | Yes | Yes | Yes | Yes |
| 4. Were objective and standard criteria used to measure the condition? | Yes | Yes | Yes | Yes | Yes |
| 5. Were confounding factors identified? | Yes | Yes | Yes | Yes | Yes |
| 6. Were strategies established to deal with confounding factors? | Yes | Yes | Yes | Yes | Yes |
| 7. Were the outcomes measured in a valid and reliable way? | Yes | Yes | Yes | Yes | Yes |
| 8. Was an appropriate statistical analysis used? | Yes | Yes | Yes | Yes | Yes |
| **Conclusion** | Low risk | Low risk | High risk | High risk | Low risk |
| **Justification of the assigned risk** | - | - | Details of the source of the population are not mentioned | Details of the source of the population are not mentioned | - |
|  |  |  |  |  |  |
|  |  |  |  |  |  |
| **Domains** | **Tang et al. (2021) (1)** | **Tang et al. (2021) (2)** | **Tang et al. (2021) (3)** | **Tatapudi et al. (2021)** | **Teslya et al. (2021)** |
|  | **18-07-22** | **18-07-22** | **06-08-22** | **06-08-22** | **06-08-22** |
| 1. Were the criteria for inclusion in the sample clearly defined? | Yes | Yes | No | Yes | Yes |
| 2. Were the study subjects and setting described in detail? | Yes | Yes | Unclear | Unclear | Unclear |
| 3. Was the exposure measured in a valid and reliable way? | Yes | Yes | Yes | Yes | Yes |
| 4. Were objective and standard criteria used to measure the condition? | Yes | Yes | Yes | Yes | Yes |
| 5. Were confounding factors identified? | Yes | Yes | Yes | Yes | Yes |
| 6. Were strategies established to deal with confounding factors? | Yes | Yes | Yes | Yes | Yes |
| 7. Were the outcomes measured in a valid and reliable way? | Yes | Yes | Yes | Yes | Yes |
| 8. Was an appropriate statistical analysis used? | Yes | Yes | Yes | Yes | Yes |
| **Conclusion** | Low risk | Low risk | High risk | Low risk | Low risk |
| **Justification of the assigned risk** | - | - | Details of the source of the population are not mentioned | - | - |
|  |  |  |  |  |  |
|  |  |  |  |  |  |
| **Domains** | **Tetteh et al. (2021)** | **Thompson et al. (2021)** | **Tonkens et al. (2021)** | **Topîrceanu (2021)** | **Torku et al. (2021)** |
|  | **06-08-22** | **06-08-22** | **06-08-22** | **06-08-22** | **06-08-22** |
| 1. Were the criteria for inclusion in the sample clearly defined? | Yes | Yes | Unclear | Unclear | Yes |
| 2. Were the study subjects and setting described in detail? | Unclear | Yes | No | Unclear | Yes |
| 3. Was the exposure measured in a valid and reliable way? | Yes | Yes | Yes | Yes | Yes |
| 4. Were objective and standard criteria used to measure the condition? | Yes | Yes | Yes | Yes | Yes |
| 5. Were confounding factors identified? | Yes | Yes | Yes | Yes | Yes |
| 6. Were strategies established to deal with confounding factors? | Yes | Yes | Yes | Yes | Yes |
| 7. Were the outcomes measured in a valid and reliable way? | Yes | Yes | Yes | Yes | Yes |
| 8. Was an appropriate statistical analysis used? | Yes | Yes | Yes | Yes | Yes |
| **Conclusion** | Low risk | Low risk | High risk | High risk | Low risk |
| **Justification of the assigned risk** | - | - | Details of the source of the population are not mentioned | Details of the source of the population are not mentioned | - |
|  |  |  |  |  |  |
|  |  |  |  |  |  |
| **Domains** | **Tran et al. (2021)** | **Truszkowska et al. (2021)** | **Truszkowska et al. (2022)** | **Usherwood et al. (2021)** | **Utamura et al. (2021)** |
|  | **06-08-22** | **06-08-22** | **08-08-22** | **08-08-22** | **08-08-22** |
| 1. Were the criteria for inclusion in the sample clearly defined? | Yes | Yes | Yes | Unclear | Yes |
| 2. Were the study subjects and setting described in detail? | Yes | Unclear | Unclear | Unclear | Yes |
| 3. Was the exposure measured in a valid and reliable way? | Yes | Yes | Yes | Yes | Yes |
| 4. Were objective and standard criteria used to measure the condition? | Yes | Yes | Yes | Yes | Yes |
| 5. Were confounding factors identified? | Yes | Yes | Yes | Yes | Yes |
| 6. Were strategies established to deal with confounding factors? | Yes | Yes | Yes | Yes | Yes |
| 7. Were the outcomes measured in a valid and reliable way? | Yes | Yes | Yes | Yes | Yes |
| 8. Was an appropriate statistical analysis used? | Yes | Yes | Yes | Yes | Yes |
| **Conclusion** | Low risk | Low risk | Low risk | High risk | Low risk |
| **Justification of the assigned risk** | - | - | - | Details of the source of the population are not mentioned | - |
|  |  |  |  |  |  |
|  |  |  |  |  |  |
| **Domains** | **Van Egeren et al. (2021)** | **Van Gordon et al. (2021)** | **Van Heusden et al. (2021)** | **Gómezet al. (2022)** | **Viana, et al. (2021)** |
|  | **08-08-22** | **08-08-22** | **08-08-22** | **08-08-22** | **08-08-22** |
| 1. Were the criteria for inclusion in the sample clearly defined? | Unclear | Yes | Yes | Yes | Yes |
| 2. Were the study subjects and setting described in detail? | Unclear | Unclear | Unclear | Unclear | Yes |
| 3. Was the exposure measured in a valid and reliable way? | Yes | Yes | Yes | Yes | Yes |
| 4. Were objective and standard criteria used to measure the condition? | Yes | Yes | Yes | Yes | Yes |
| 5. Were confounding factors identified? | Yes | Yes | Yes | Yes | Yes |
| 6. Were strategies established to deal with confounding factors? | Yes | Yes | Yes | Yes | Yes |
| 7. Were the outcomes measured in a valid and reliable way? | Yes | Yes | Yes | Yes | Yes |
| 8. Was an appropriate statistical analysis used? | Yes | Yes | Yes | Yes | Yes |
| **Conclusion** | Unclear | Low risk | Low risk | Low risk | Low risk |
| **Justification of the assigned risk** | The information is not clear | - | - | - | - |
|  |  |  |  |  |  |
|  |  |  |  |  |  |
| **Domains** | **Vignals et al. (2021)** | **Vilches et al. (2021) (1)** | **Vilches et al. (2021) (2)** | **Vilches et al. (2021) (3)** | **Vilches et al. (2022)** |
|  | **08-08-22** | **08-08-22** | **08-08-22** | **08-08-22** | **08-08-22** |
| 1. Were the criteria for inclusion in the sample clearly defined? | Yes | Yes | Unclear | Unclear | Yes |
| 2. Were the study subjects and setting described in detail? | Unclear | Unclear | Unclear | Unclear | Unclear |
| 3. Was the exposure measured in a valid and reliable way? | Yes | Yes | Yes | Yes | Yes |
| 4. Were objective and standard criteria used to measure the condition? | Yes | Yes | Yes | Yes | Yes |
| 5. Were confounding factors identified? | Yes | Unclear | Yes | Yes | Unclear |
| 6. Were strategies established to deal with confounding factors? | Yes | Unclear | Yes | Yes | Unclear |
| 7. Were the outcomes measured in a valid and reliable way? | Yes | Yes | Yes | Yes | Yes |
| 8. Was an appropriate statistical analysis used? | Yes | Yes | Yes | Yes | Yes |
| **Conclusion** | Low risk | High risk | High risk | High risk | High risk |
| **Justification of the assigned risk** | - | Although there is information about the population, it does not have information related to the modeling | There is no clear information on the population | There is no clear information on the population | Although there is information about the population, it does not have information related to the modeling |
|  |  |  |  |  |  |
|  |  |  |  |  |  |
| **Domains** | **De Visscher et al. (2021)** | **Volodymyrovych et al. (2021)** | **Walker et al. (2022)** | **Wang et al. (2021)** | **Wang et al. (2021)** |
|  | **08-08-22** | **08-08-22** | **08-08-22** | **08-08-22** | **09-08-22** |
| 1. Were the criteria for inclusion in the sample clearly defined? | Yes | Yes | Yes | Yes | Yes |
| 2. Were the study subjects and setting described in detail? | Unclear | Unclear | Unclear | Unclear | Unclear |
| 3. Was the exposure measured in a valid and reliable way? | Yes | Yes | Yes | Yes | Yes |
| 4. Were objective and standard criteria used to measure the condition? | Yes | Yes | Yes | Yes | Yes |
| 5. Were confounding factors identified? | Yes | Yes | Yes | Unclear | Unclear |
| 6. Were strategies established to deal with confounding factors? | Yes | Yes | Yes | Unclear | Unclear |
| 7. Were the outcomes measured in a valid and reliable way? | Yes | Yes | Yes | Yes | Yes |
| 8. Was an appropriate statistical analysis used? | Yes | Yes | Yes | Yes | Yes |
| **Conclusion** | Low risk | Low risk | Low risk | High risk | High risk |
| **Justification of the assigned risk** | - | - | - | Although there is information about the population, it does not have information related to the modeling | Although there is information about the population, it does not have information related to the modeling |
|  |  |  |  |  |  |
|  |  |  |  |  |  |
| **Domains** | **Wang et al. (2022)** | **Webb (2021)** | **Więcek et al. (2021)** | **Więcek et al. (2022)** | **Wieland et al. (2021)** |
|  | **09-08-22** | **09-08-22** | **09-08-22** | **09-08-22** | **09-08-22** |
| 1. Were the criteria for inclusion in the sample clearly defined? | Yes | Yes | Yes | Yes | Yes |
| 2. Were the study subjects and setting described in detail? | Unclear | Unclear | Yes | Unclear | Unclear |
| 3. Was the exposure measured in a valid and reliable way? | Yes | Yes | Yes | Yes | Yes |
| 4. Were objective and standard criteria used to measure the condition? | Yes | Yes | Yes | Yes | Yes |
| 5. Were confounding factors identified? | Yes | Yes | Yes | Yes | Unclear |
| 6. Were strategies established to deal with confounding factors? | Yes | Yes | Yes | Yes | Unclear |
| 7. Were the outcomes measured in a valid and reliable way? | Yes | Yes | Yes | Yes | Yes |
| 8. Was an appropriate statistical analysis used? | Yes | Yes | Yes | Yes | Yes |
| **Conclusion** | Low risk | Low risk | Low risk | Low risk | High risk |
| **Justification of the assigned risk** | - | - | - | - | Although there is information about the population, the information related to the modeling is not clear |
|  |  |  |  |  |  |
|  |  |  |  |  |  |
| **Domains** | **Conn et al. (2021)** | **Wirtz (2021)** | **Wong et al. (2021)** | **Xavier et al. (2022)** | **Xiong et al. (2022)** |
|  | **09-08-22** | **09-08-22** | **09-08-22** | **09-08-22** | **09-08-22** |
| 1. Were the criteria for inclusion in the sample clearly defined? | Yes | Yes | No | Yes | Yes |
| 2. Were the study subjects and setting described in detail? | Unclear | Unclear | No | Unclear | Unclear |
| 3. Was the exposure measured in a valid and reliable way? | Yes | Yes | Yes | Yes | Yes |
| 4. Were objective and standard criteria used to measure the condition? | Yes | Yes | Yes | Yes | Yes |
| 5. Were confounding factors identified? | Yes | Yes | Unclear | Yes | Yes |
| 6. Were strategies established to deal with confounding factors? | Yes | Yes | Unclear | Yes | Yes |
| 7. Were the outcomes measured in a valid and reliable way? | Yes | Yes | Yes | Yes | Yes |
| 8. Was an appropriate statistical analysis used? | Yes | Yes | Yes | Yes | Yes |
| **Conclusion** | Low risk | Low risk | High risk | Low risk | Low risk |
| **Justification of the assigned risk** | - | - | There is no explicit information on the origin of the data. There are also methodological gaps | - | - |
|  |  |  |  |  |  |
|  |  |  |  |  |  |
| **Domains** | **Yang et al. (2021) (1)** | **Yang et al. (2021) (2)** | **Yang et al. (2021) (3)** | **Yang et al. (2021) (4)** | **Yang et al. (2022) (1)** |
|  | **09-08-22** | **09-08-22** | **09-08-22** | **09-08-22** | **09-08-22** |
| 1. Were the criteria for inclusion in the sample clearly defined? | Yes | Yes | Yes | No | Yes |
| 2. Were the study subjects and setting described in detail? | Unclear | Unclear | Unclear | No | Unclear |
| 3. Was the exposure measured in a valid and reliable way? | Yes | Yes | Yes | Yes | Yes |
| 4. Were objective and standard criteria used to measure the condition? | Yes | Yes | Yes | Yes | Yes |
| 5. Were confounding factors identified? | Yes | Yes | Yes | Unclear | Yes |
| 6. Were strategies established to deal with confounding factors? | Yes | Yes | Yes | Unclear | Yes |
| 7. Were the outcomes measured in a valid and reliable way? | Yes | Yes | Yes | Yes | Yes |
| 8. Was an appropriate statistical analysis used? | Yes | Yes | Yes | Yes | Yes |
| **Conclusion** | Low risk | Low risk | Low risk | High risk | Low risk |
| **Justification of the assigned risk** | - | - | - | There is no explicit information on the origin of the data. There are also methodological gaps | - |
|  |  |  |  |  |  |
|  |  |  |  |  |  |
| **Domains** | **Yang et al. (2022) (2)** | **Yang et al. (2021) (5)** | **Young et al. (2021)** | **Yu et al. (2021)** | **Yuan et al. (2022)** |
|  | **09-08-22** | **09-08-22** | **09-08-22** | **10-08-22** | **10-08-22** |
| 1. Were the criteria for inclusion in the sample clearly defined? | Yes | Yes | No | Yes | Yes |
| 2. Were the study subjects and setting described in detail? | Yes | Unclear | No | Yes | Unclear |
| 3. Was the exposure measured in a valid and reliable way? | Yes | Yes | Yes | Yes | Yes |
| 4. Were objective and standard criteria used to measure the condition? | Yes | Yes | Yes | Yes | Yes |
| 5. Were confounding factors identified? | Yes | Yes | Yes | Yes | Yes |
| 6. Were strategies established to deal with confounding factors? | Yes | Yes | Yes | Yes | Yes |
| 7. Were the outcomes measured in a valid and reliable way? | Yes | Yes | Yes | Yes | Yes |
| 8. Was an appropriate statistical analysis used? | Yes | Yes | Yes | Yes | Yes |
| **Conclusion** | Low risk | Low risk | High risk | Low risk | Low risk |
| **Justification of the assigned risk** | - | - | There is no information related to the origin of the data | - | - |
|  |  |  |  |  |  |
|  |  |  |  |  |  |
| **Domains** | **Zachreson et al. (2022)** | **Zhang et al. (2021) (1)** | **Zhang et al. (2021) (2)** | **Zhang et al. (2022)** | **Zhao et al. (2021) (1)** |
|  | **10-08-22** | **10-08-22** | **10-08-22** | **10-08-22** | **10-08-22** |
| 1. Were the criteria for inclusion in the sample clearly defined? | Unclear | Yes | Unclear | Yes | Unclear |
| 2. Were the study subjects and setting described in detail? | Unclear | Unclear | Unclear | Unclear | Unclear |
| 3. Was the exposure measured in a valid and reliable way? | Yes | Yes | Yes | Yes | Yes |
| 4. Were objective and standard criteria used to measure the condition? | Yes | Yes | Yes | Yes | Yes |
| 5. Were confounding factors identified? | Yes | Yes | Yes | Yes | Yes |
| 6. Were strategies established to deal with confounding factors? | Yes | Yes | Yes | Yes | Yes |
| 7. Were the outcomes measured in a valid and reliable way? | Yes | Yes | Yes | Yes | Yes |
| 8. Was an appropriate statistical analysis used? | Yes | Yes | Yes | Yes | Yes |
| **Conclusion** | High risk | Low risk | High risk | Low risk | High risk |
| **Justification of the assigned risk** | There is no clear information on the population | - | There is no clear information on the population | - | The information related to the origin of the data is not clear |
|  |  |  |  |  |  |
|  |  |  |  |  |  |
| **Domains** | **Zhao et al. (2021) (2)** | **Zhao et al. (2021) (3)** | **Zhao et al. (2021) (4)** | **Zhao et al. (2021) (5)** | **Zhou et al. (2021)** |
|  | **10-08-22** | **10-08-22** | **10-08-22** | **10-08-22** | **10-08-22** |
| 1. Were the criteria for inclusion in the sample clearly defined? | Yes | Yes | Yes | Yes | Yes |
| 2. Were the study subjects and setting described in detail? | Unclear | Unclear | Unclear | Unclear | Unclear |
| 3. Was the exposure measured in a valid and reliable way? | Yes | Yes | Yes | Yes | Yes |
| 4. Were objective and standard criteria used to measure the condition? | Yes | Yes | Yes | Yes | Yes |
| 5. Were confounding factors identified? | Yes | Yes | Yes | Yes | Yes |
| 6. Were strategies established to deal with confounding factors? | Yes | Yes | Yes | Yes | Yes |
| 7. Were the outcomes measured in a valid and reliable way? | Yes | Yes | Yes | Yes | Yes |
| 8. Was an appropriate statistical analysis used? | Yes | Yes | Yes | Yes | Yes |
| **Conclusion** | Low risk | Low risk | Low risk | Low risk | Low risk |
| **Justification of the assigned risk** | - | - | - | - | - |
|  |  |  |  |  |  |
|  |  |  |  |  |  |
| **Domains** | **Suan Zhu et al. (2022)** | **Zia et al. (2021)** | **Zou et al. (2021)** | **Zou et al. (2022)** |  |
|  | **10-08-22** | **10-08-22** | **10-08-22** | **10-08-22** |  |
| 1. Were the criteria for inclusion in the sample clearly defined? | Yes | Unclear | Unclear | Yes |  |
| 2. Were the study subjects and setting described in detail? | Unclear | Unclear | Unclear | Unclear |  |
| 3. Was the exposure measured in a valid and reliable way? | Yes | Yes | Yes | Yes |  |
| 4. Were objective and standard criteria used to measure the condition? | Yes | Yes | Yes | Yes |  |
| 5. Were confounding factors identified? | Yes | Yes | Yes | Yes |  |
| 6. Were strategies established to deal with confounding factors? | Yes | Yes | Yes | Yes |  |
| 7. Were the outcomes measured in a valid and reliable way? | Yes | Yes | Yes | Yes |  |
| 8. Was an appropriate statistical analysis used? | Yes | Yes | Yes | Yes |  |
| **Conclusion** | Low risk | High risk | High risk | Low risk |  |
| **Justification of the assigned risk** | - | The information related to the origin of the data is not clear | The information related to the origin of the data is not clear | - |  |
