# Supplementary material for: Predictive models for health outcomes due to SARS-CoV-2, including the effect of vaccination: a systematic review
Source: Syst Rev. 2024 Jan 16;13:30. doi: 10.1186/s13643-023-02411-1 (PMC10790449; doi:10.1186/s13643-023-02411-1)
Supplement: Supplementary file 7 — Supplementary Material Supplementary material N°. 7. Quality assessment (Johanna Briggs Institute Checklist, PROBAST Checklist). [file 13643_2023_2411_MOESM7_ESM.zip › 12903_2024_5066_MOESM7_ESM/Supp. N°. 7 PROBAST.docx]

**Supplementary material N°. 7. Quality assessment**

**PROBAST Checklist**

| **Domain 1** | **Lasser et al. (2022)** | **Acuña et al. (2021)** | **Adibi et al. (2021)** | **Mahmoud et al. (2021)** | **Agarwal et al. (2021)** |
| --- | --- | --- | --- | --- | --- |
| **A. Risk of bias** | **02-06-22** | **05-06-22** | **05-06-22** | **05-06-22** | **05-06-22** |
| 1.1 Were appropriate data sources used, eg cohort, RCT or nested case-control study data? | Yes | No | No | No | No |
| 1.2 Were all inclusions and exclusions of participants appropriate? | Yes | No | No | No | No |
| Risk of bias introduced by selection of participants (low/ high/ unclear) | Low | High | High | High | High |
| **B. Applicability** |  |  |  |  |  |
| Concern that the included participants and setting do not match the review question (low/ high/ unclear) | Low | Unclear | Unclear | Unclear | Unclear |
| **Domain 2** |  |  |  |  |  |
| **A. Riks of bias - Predictors** |  |  |  |  |  |
| 2.1 Were predictors defined and assessed in a similar way for all participants? | Yes | Yes | Yes | Yes | Yes |
| 2.2 Were predictor assessments made without knowledge of outcome data? | Yes | Yes | Yes | Yes | Yes |
| 2.3 Are all predictors available at the time the model is intended to be used? | Yes | Yes | Yes | Yes | Yes |
| Risk of bias introduced by predictors or their assessment (low/ high/ unclear) | Low | Low | Low | Low | Low |
| **B. Applicability** |  |  |  |  |  |
| Concern that the definition, assessment or timing of predictors in the model do not match the review question (low/ high/ unclear) | Low | Low | Low | Low | Low |
| **Domain 3** |  |  |  |  |  |
| **A. Riks of bias - outcome** |  |  |  |  |  |
| Was the outcome determined appropriately? | Yes | Yes | Yes | Yes | Yes |
| Was a pre-specified or standard outcome definition used? | Yes | Yes | Yes | Yes | Yes |
| Were predictors excluded from the outcome definition? | Yes | Yes | Yes | Yes | Yes |
| Was the outcome defined and determined in a similar way for all participants? | Yes | Yes | Yes | Yes | Yes |
| Was the outcome determined without knowledge of predictor information? | Yes | Yes | Yes | Yes | Yes |
| Was the time interval between predictor assessment and outcome determination appropriate? | Yes | Yes | Yes | Yes | Yes |
| **Risk of bias introduced by the outcome or its determination (low/ high/ Unclear)** | Low | Low | Low | Low | Low |
| **B. Applicability** |  |  |  |  |  |
| If a composite outcome was used, describe the relative frequency/distribution of each contributing outcome: | NA | NA | NA | NA | NA |
| Concern that the outcome, its definition, timing or determination do not match the review question (low/ high/ unclear) | Low | Low | Low | Low | Low |
| **Domain 4** |  |  |  |  |  |
| **A. Risk of bias** |  |  |  |  |  |
| Describe numbers of participants, number of candidate predictors, outcome events and events per candidate predictor: | Yes | Yes | Yes | Yes | Yes |
| Describe how the model was developed (for example in regards to modeling technique (eg survival or logistic modelling), predictor selection, and risk group definition): | Yes | Yes | Yes | Yes | Yes |
| Describe whether and how the model was validated, either internally (eg bootstrapping, cross validation, random split sample) or externally (eg temporal validation, geographical validation, different setting, different type of participants): | Yes | Yes | Yes | Yes | Yes |
| Describe the performance measures of the model, eg (re)calibration, discrimination, (re)classification, net benefit, and whether they were adjusted for optimism: | Yes | Yes | Yes | Yes | Yes |
| Describe any participants who were excluded from the analysis: | Yes | Yes | Yes | Yes | Yes |
| Describe missing data on predictors and outcomes as well as methods used for missing data: | No | Yes | Yes | Yes | Yes |
| 4.1 Were there a reasonable number of participants with the outcome? | Yes | Yes | Yes | Yes | Yes |
| 4.2 Were continuous and categorical predictors handled appropriately? | Yes | Yes | Yes | Yes | Yes |
| 4.3 Were all enrolled participants included in the analysis? | Yes | Yes | Yes | Yes | Yes |
| 4.4 Were participants with missing data handled appropriately? | Yes | Yes | Yes | Yes | Yes |
| 4.5 Was selection of predictors based on univariate analysis avoided? | Yes | Yes | Yes | Yes | Yes |
| 4.6 Were complexities in the data (eg censoring, competing risks, sampling of controls) accounted for appropriately? | Yes | Yes | Yes | Yes | Yes |
| 4.7 Are relevant model performance measures evaluated appropriately? | Yes | Yes | Yes | Yes | Yes |
| 4.8 Were model overfitting and optimism in model performance accounted for? | Yes | Yes | Yes | Yes | Yes |
| 4.9 Do predictors and their assigned weights in the final model correspond to the results from multivariable analysis? | Yes | Yes | Yes | Yes | Yes |
| **Risk of bias introduced by the analysis (low/ high/ Unclear)** | Low | Low | Low | Low | Low |
| **Step 4: Overall assessment. Use the following tables to reach overall judgments about risk of bias and concerns regarding applicability of the prediction model evaluation (development and/or validation) across all assessed domains. Complete for each evaluation of a distinct model.** | **-** | There is not enough information related to the chosen population, so there is a risk of introducing bias in the selection | There is not enough information related to the chosen population, so there is a risk of introducing bias in the selection | There is not enough information related to the chosen population, so there is a risk of introducing bias in the selection | There is not enough information related to the chosen population, so there is a risk of introducing bias in the selection |
| **Overall judgment of risk of bias** | Low risk | High risk | High risk | High risk | High risk |
| **Overall judgment of applicability** | Low risk | Low risk | Low risk | Low risk | Low risk |
|  |  |  |  |  |  |
|  |  |  |  |  |  |
| **Domain 1** | **Aguas et al. (2021)** | **Aguiar et al. (2021)** | **Aguilar-Canto et al. (2021)** | **Aguilar-Canto et al. (2022)** | **Ainslie et al. (2021)** |
| **A. Risk of bias** | **05-06-22** | **05-06-22** | **05-06-22** | **05-06-22** | **05-06-22** |
| 1.1 Were appropriate data sources used, e.g. data from cohort studies, RCTs or nested case-controls? | No | No | Unclear | No | No |
| 1.2 Were all inclusions and exclusions of participants appropriate? | No | No | No | No | No |
| Risk of bias introduced by participant selection (low/high/unclear) | High | High | High | High | High |
| **B. Applicability** |  |  |  |  |  |
| Concern that included participants and setting do not match review question (low/high/unclear) | Unclear | Unclear | Unclear | Unclear | Unclear |
| **Domain 2** |  |  |  |  |  |
| **A. Risk of bias - predictors** |  |  |  |  |  |
| 2.1 Were predictors defined and evaluated in a similar way for all participants? | Yes | Yes | Yes | Yes | Yes |
| 2.2 Were predictor evaluations performed without knowing the outcome data? | Yes | Yes | Yes | Yes | Yes |
| 2.3 Are all the predictors available at the time the model is intended to be used? | Yes | Yes | Yes | Yes | Yes |
| Risk of bias introduced by the predictors or their assessment (low/high/unclear) | Low | Low | Low | Low | Low |
| **B. Applicability** |  |  |  |  |  |
| Concern that the definition, evaluation, or timing of predictors in the model do not match the review question (low/high/unclear) | Low | Low | Low | Low | Low |
| **Domain 3** |  |  |  |  |  |
| **A. Risk of bias - outcome** |  |  |  |  |  |
| Was the result properly determined? | Yes | Yes | Yes | Yes | Yes |
| Was a standard or prespecified outcome definition used? | Yes | Yes | Yes | Yes | Yes |
| Were predictors excluded from the outcome definition? | Yes | Yes | Yes | Yes | Yes |
| Was the outcome similarly defined and determined for all participants? | Yes | Yes | Yes | Yes | Yes |
| Was the outcome determined without knowing the predictor information? | Yes | Yes | Yes | Yes | Yes |
| Was the time interval between the evaluation of the predictor and the determination of the result adequate? | Yes | Yes | Yes | Yes | Yes |
| Risk of bias introduced by the result or its determination (low/high/unclear) | Low | Low | Low | Low | Low |
| **B. Applicability** |  |  |  |  |  |
| If a composite outcome was used, please describe the relative frequency/distribution of each contributing outcome: | NA | NA | NA | NA | NA |
| Concern that the outcome, its definition, timing, or determination does not match the review question (low/high/unclear) | Low | Low | Low | Low | Low |
| **Domain 4** |  |  |  |  |  |
| **A. Risk of bias** |  |  |  |  |  |
| Describe the number of participants, number of candidate predictors, outcome events, and events per candidate predictor: | Yes | Yes | Yes | Yes | Yes |
| Describe how the model was developed (for example, with respect to modeling technique (eg, survival or logistic modeling), selection of predictors, and definition of risk groups): | Yes | Yes | Yes | Yes | Yes |
| Describe if and how the model was validated, either internally (eg, bootstrapping, cross-validation, split random sample) or externally (eg, temporal validation, geographic validation, different setting, different type of participants): | Yes | Yes | Yes | Yes | Yes |
| Describe the performance measures of the model, e.g. (re)calibration, discrimination, (re)classification, net profit and if adjusted for optimism: | Yes | Yes | Yes | Yes | Yes |
| Describe the participants who were excluded from the analysis: | Yes | Yes | Yes | Yes | Yes |
| Describe missing data on predictors and outcomes, as well as methods used for missing data: | Yes | Yes | Yes | Yes | Yes |
| 4.1 Was there a reasonable number of participants with the result? | Yes | Yes | Yes | Yes | Yes |
| 4.2 Were continuous and categorical predictors handled correctly? | Yes | Yes | Yes | Yes | Yes |
| 4.3 Were all enrolled participants included in the analysis? | Yes | Yes | Yes | Yes | Yes |
| 4.4 Were participants with missing data treated appropriately? | Yes | Yes | Yes | Yes | Yes |
| 4.5 Was selection of predictors based on univariate analysis avoided? | Yes | Yes | Yes | Yes | Yes |
| 4.6 Were the complexities of the data (eg censoring, competing risks, sampling of controls) adequately accounted for? | Yes | Yes | Yes | Yes | Yes |
| 4.7 Were relevant model performance measures adequately evaluated? | Yes | Yes | Yes | Yes | Yes |
| 4.8 Was model overfitting and optimism factored into model performance? | Yes | Yes | Yes | Yes | Yes |
| 4.9 Do the predictors and their assigned weights in the final model correspond to the results of the multivariate analysis? | Yes | Yes | Yes | Yes | Yes |
| **Risk of bias introduced by the analysis (low/high/Unclear)** | Low | Low | Low | Low | Low |
| **Step 4: General evaluation** | There is not enough information related to the chosen population, so there is a risk of introducing bias in the selection | There is not enough information related to the chosen population, so there is a risk of introducing bias in the selection | There is not enough information related to the chosen population, so there is a risk of introducing bias in the selection | There is not enough information related to the chosen population, so there is a risk of introducing bias in the selection | There is not enough information related to the chosen population, so there is a risk of introducing bias in the selection |
| **Overall assessment of risk of bias** | High risk | High risk | High risk | High risk | High risk |
| **General judgment of applicability** | Low risk | Low risk | Low risk | Low risk | Low risk |
|  |  |  |  |  |  |
|  |  |  |  |  |  |
| **Domain 1** | **Alagoz et al. (2021)** | **Albani et al. (2021) (1)** | **Albani et al. (2021) (2)** | **Aldila et al. (2021)** | **Almajose et al. (2021)** |
| **A. Risk of bias** | **05-06-22** | **05-06-22** | **06-06-22** | **07-06-22** | **08-06-22** |
| 1.1 Were appropriate data sources used, e.g. data from cohort studies, RCTs or nested case-controls? | No | No | Yes | No | Yes |
| 1.2 Were all inclusions and exclusions of participants appropriate? | No | No | Yes | No | Yes |
| Risk of bias introduced by participant selection (low/high/unclear) | High | High | Low | High | Low |
| **B. Applicability** |  |  |  |  |  |
| Concern that included participants and setting do not match review question (low/high/unclear) | Unclear | Unclear | Low | Unclear | Low |
| **Domain 2** |  |  |  |  |  |
| **A. Risk of bias - predictors** |  |  |  |  |  |
| 2.1 Were predictors defined and evaluated in a similar way for all participants? | Yes | Yes | Yes | Yes | Yes |
| 2.2 Were predictor evaluations performed without knowing the outcome data? | Yes | Yes | Yes | Yes | Yes |
| 2.3 Are all the predictors available at the time the model is intended to be used? | Yes | Yes | Yes | Yes | Yes |
| Risk of bias introduced by the predictors or their assessment (low/high/unclear) | Low | Low | Low | Low | Low |
| **B. Applicability** |  |  |  |  |  |
| Concern that the definition, evaluation, or timing of predictors in the model do not match the review question (low/high/unclear) | Low | Low | Low | Low | Low |
| **Domain 3** |  |  |  |  |  |
| **A. Risk of bias - outcome** |  |  |  |  |  |
| Was the result properly determined? | Yes | Yes | Yes | Yes | Yes |
| Was a standard or prespecified outcome definition used? | Yes | Yes | Yes | Yes | Yes |
| Were predictors excluded from the outcome definition? | Yes | Yes | Yes | Yes | Yes |
| Was the outcome similarly defined and determined for all participants? | Yes | Yes | Yes | Yes | Yes |
| Was the outcome determined without knowing the predictor information? | Yes | Yes | Yes | Yes | Yes |
| Was the time interval between the evaluation of the predictor and the determination of the result adequate? | Yes | Yes | Yes | Yes | Yes |
| Risk of bias introduced by the result or its determination (low/high/unclear) | Low | Low | Low | Low | Low |
| **B. Applicability** |  |  |  |  |  |
| If a composite outcome was used, please describe the relative frequency/distribution of each contributing outcome: | Yes | NA | NA | NA | NA |
| Concern that the outcome, its definition, timing, or determination does not match the review question (low/high/unclear) | Low | Low | Low | Low | Low |
| **Domain 4** |  |  |  |  |  |
| **A. Risk of bias** |  |  |  |  |  |
| Describe the number of participants, number of candidate predictors, outcome events, and events per candidate predictor: | Yes | Yes | Yes | Yes | Yes |
| Describe how the model was developed (for example, with respect to modeling technique (eg, survival or logistic modeling), selection of predictors, and definition of risk groups): | Yes | Yes | Yes | Yes | Yes |
| Describe if and how the model was validated, either internally (eg, bootstrapping, cross-validation, split random sample) or externally (eg, temporal validation, geographic validation, different setting, different type of participants): | Yes | Yes | Yes | Yes | Yes |
| Describe the performance measures of the model, e.g. (re)calibration, discrimination, (re)classification, net profit and if adjusted for optimism: | Yes | Yes | Yes | Yes | Yes |
| Describe the participants who were excluded from the analysis: | Yes | Yes | Yes | Yes | Yes |
| Describe missing data on predictors and outcomes, as well as methods used for missing data: | Yes | Yes | Yes | Yes | Yes |
| 4.1 Was there a reasonable number of participants with the result? | Yes | Yes | Yes | Yes | Yes |
| 4.2 Were continuous and categorical predictors handled correctly? | Yes | Yes | Yes | Yes | Yes |
| 4.3 Were all enrolled participants included in the analysis? | Yes | Yes | Yes | Yes | Yes |
| 4.4 Were participants with missing data treated appropriately? | Yes | Yes | Yes | Yes | Yes |
| 4.5 Was selection of predictors based on univariate analysis avoided? | Yes | Yes | Yes | Yes | Yes |
| 4.6 Were the complexities of the data (eg censoring, competing risks, sampling of controls) adequately accounted for? | Yes | Yes | Yes | Yes | Yes |
| 4.7 Were relevant model performance measures adequately evaluated? | Yes | Yes | Yes | Yes | Yes |
| 4.8 Was model overfitting and optimism factored into model performance? | Yes | Yes | Yes | Yes | Yes |
| 4.9 Do the predictors and their assigned weights in the final model correspond to the results of the multivariate analysis? | Yes | Yes | Yes | Yes | Yes |
| **Risk of bias introduced by the analysis (low/high/Unclear)** | Low | Low | Low | Low | Low |
| **Step 4: General evaluation** | There is not enough information related to the chosen population, so there is a risk of introducing bias in the selection | There is not enough information related to the chosen population, so there is a risk of introducing bias in the selection | - | There is not enough information related to the chosen population, so there is a risk of introducing bias in the selection | - |
| **Overall assessment of risk of bias** | High risk | High risk | Low risk | High risk | Low risk |
| **General judgment of applicability** | Low risk | Low risk | Low risk | Low risk | Low risk |
|  |  |  |  |  |  |
|  |  |  |  |  |  |
| **Domain 1** | **Amaku et al. (2021)** | **Amaral et al. (2021)** | **Amouch et al. (2021)** | **Antonini et al. (2021)** | **Are et al. (2021)** |
| **A. Risk of bias** | **05-06-22** | **05-06-22** | **05-06-22** | **05-06-22** | **05-06-22** |
| 1.1 Were appropriate data sources used, e.g. data from cohort studies, RCTs or nested case-controls? | No | No | No | Yes | No |
| 1.2 Were all inclusions and exclusions of participants appropriate? | No | No | No | Unclear | No |
| Risk of bias introduced by participant selection (low/high/unclear) | High | High | High | Low | High |
| **B. Applicability** |  |  |  |  |  |
| Concern that included participants and setting do not match review question (low/high/unclear) | Unclear | Unclear | Unclear | Low | Unclear |
| **Domain 2** |  |  |  |  |  |
| **A. Risk of bias - predictors** |  |  |  |  |  |
| 2.1 Were predictors defined and evaluated in a similar way for all participants? | Yes | Yes | Yes | Yes | Yes |
| 2.2 Were predictor evaluations performed without knowing the outcome data? | Yes | Yes | Yes | Yes | Yes |
| 2.3 Are all the predictors available at the time the model is intended to be used? | Yes | Yes | Yes | Yes | Yes |
| Risk of bias introduced by the predictors or their assessment (low/high/unclear) | Low | Low | Low | Low | Low |
| **B. Applicability** |  |  |  |  |  |
| Concern that the definition, evaluation, or timing of predictors in the model do not match the review question (low/high/unclear) | Low | Low | Low | Low | Low |
| **Domain 3** |  |  |  |  |  |
| **A. Risk of bias - outcome** |  |  |  |  |  |
| Was the result properly determined? | Yes | Yes | Yes | Yes | Yes |
| Was a standard or prespecified outcome definition used? | Yes | Yes | Yes | Yes | Yes |
| Were predictors excluded from the outcome definition? | Yes | Yes | Yes | Yes | Yes |
| Was the outcome similarly defined and determined for all participants? | Yes | Yes | Yes | Yes | Yes |
| Was the outcome determined without knowing the predictor information? | Yes | Yes | Yes | Yes | Yes |
| Was the time interval between the evaluation of the predictor and the determination of the result adequate? | Yes | Yes | Yes | Yes | Yes |
| Risk of bias introduced by the result or its determination (low/high/unclear) | Low | Low | Low | Low | Low |
| **B. Applicability** |  |  |  |  |  |
| If a composite outcome was used, please describe the relative frequency/distribution of each contributing outcome: | NA | NA | NA | NA | NA |
| Concern that the outcome, its definition, timing, or determination does not match the review question (low/high/unclear) | Low | Low | Low | Low | Low |
| **Domain 4** |  |  |  |  |  |
| **A. Risk of bias** |  |  |  |  |  |
| Describe the number of participants, number of candidate predictors, outcome events, and events per candidate predictor: | Yes | Yes | Yes | Yes | Yes |
| Describe how the model was developed (for example, with respect to modeling technique (eg, survival or logistic modeling), selection of predictors, and definition of risk groups): | Yes | Yes | Yes | Yes | Yes |
| Describe if and how the model was validated, either internally (eg, bootstrapping, cross-validation, split random sample) or externally (eg, temporal validation, geographic validation, different setting, different type of participants): | Yes | Yes | Yes | Yes | Yes |
| Describe the performance measures of the model, e.g. (re)calibration, discrimination, (re)classification, net profit and if adjusted for optimism: | Yes | Yes | Yes | Yes | Yes |
| Describe the participants who were excluded from the analysis: | Yes | Yes | Yes | Yes | Yes |
| Describe missing data on predictors and outcomes, as well as methods used for missing data: | Yes | Yes | Yes | Yes | Yes |
| 4.1 Was there a reasonable number of participants with the result? | Yes | Yes | Yes | Yes | Yes |
| 4.2 Were continuous and categorical predictors handled correctly? | Yes | Yes | Yes | Yes | Yes |
| 4.3 Were all enrolled participants included in the analysis? | Yes | Yes | Yes | Yes | Yes |
| 4.4 Were participants with missing data treated appropriately? | Yes | Yes | Yes | Yes | Yes |
| 4.5 Was selection of predictors based on univariate analysis avoided? | Yes | Yes | Yes | Yes | Yes |
| 4.6 Were the complexities of the data (eg censoring, competing risks, sampling of controls) adequately accounted for? | Yes | Yes | Yes | Yes | Yes |
| 4.7 Were relevant model performance measures adequately evaluated? | Yes | Yes | Yes | Yes | Yes |
| 4.8 Was model overfitting and optimism factored into model performance? | Yes | Yes | Yes | Yes | Yes |
| 4.9 Do the predictors and their assigned weights in the final model correspond to the results of the multivariate analysis? | Yes | Yes | Yes | Yes | Yes |
| **Risk of bias introduced by the analysis (low/high/Unclear)** | Low | Low | Low | Low | Low |
| **Step 4: General evaluation** | There is not enough information related to the chosen population, so there is a risk of introducing bias in the selection | There is not enough information related to the chosen population, so there is a risk of introducing bias in the selection | There is not enough information related to the chosen population, so there is a risk of introducing bias in the selection | - | There is not enough information related to the chosen population, so there is a risk of introducing bias in the selection |
| **Overall assessment of risk of bias** | High | High | High | Low | High |
| **General judgment of applicability** | Low | Low | Low | Low | Low |
|  |  |  |  |  |  |
|  |  |  |  |  |  |
| **Domain 1** | **Arslan et al. (2021) (1)** | **Arslan et al. (2021) (2)** | **Aruffo et al. (2021) (1)** | **Aruffo et al. (2021) (2)** | **Adiga et al. (2021)** |
| **A. Risk of bias** | **05-06-22** | **05-06-22** | **05-06-22** | **05-06-22** | **05-06-22** |
| 1.1 Were appropriate data sources used, e.g. data from cohort studies, RCTs or nested case-controls? | Yes | Yes | Yes | Yes | Yes |
| 1.2 Were all inclusions and exclusions of participants appropriate? | Unclear | Unclear | Unclear | Unclear | Unclear |
| Risk of bias introduced by participant selection (low/high/unclear) | Low | Low | Low | Low | Low |
| **B. Applicability** |  |  |  |  |  |
| Concern that included participants and setting do not match review question (low/high/unclear) | Low | Low | Low | Low | Low |
| **Domain 2** |  |  |  |  |  |
| **A. Risk of bias - predictors** |  |  |  |  |  |
| 2.1 Were predictors defined and evaluated in a similar way for all participants? | Yes | Yes | Yes | Yes | Yes |
| 2.2 Were predictor evaluations performed without knowing the outcome data? | Yes | Yes | Yes | Yes | Yes |
| 2.3 Are all the predictors available at the time the model is intended to be used? | Yes | Yes | Yes | Yes | Yes |
| Risk of bias introduced by the predictors or their assessment (low/high/unclear) | Low | Low | Low | Low | Low |
| **B. Applicability** |  |  |  |  |  |
| Concern that the definition, evaluation, or timing of predictors in the model do not match the review question (low/high/unclear) | Low | Low | Low | Low | Low |
| **Domain 3** |  |  |  |  |  |
| **A. Risk of bias - outcome** |  |  |  |  |  |
| Was the result properly determined? | Yes | Yes | Yes | Yes | Yes |
| Was a standard or prespecified outcome definition used? | Yes | Yes | Yes | Yes | Yes |
| Were predictors excluded from the outcome definition? | Yes | Yes | Yes | Yes | Yes |
| Was the outcome similarly defined and determined for all participants? | Yes | Yes | Yes | Yes | Yes |
| Was the outcome determined without knowing the predictor information? | Yes | Yes | Yes | Yes | Yes |
| Was the time interval between the evaluation of the predictor and the determination of the result adequate? | Yes | Yes | Yes | Yes | Yes |
| Risk of bias introduced by the result or its determination (low/high/unclear) | Low | Low | Low | Low | Low |
| **B. Applicability** |  |  |  |  |  |
| If a composite outcome was used, please describe the relative frequency/distribution of each contributing outcome: | NA | NA | NA | NA | NA |
| Concern that the outcome, its definition, timing, or determination does not match the review question (low/high/unclear) | Low | Low | Low | Low | Low |
| **Domain 4** |  |  |  |  |  |
| **A. Risk of bias** |  |  |  |  |  |
| Describe the number of participants, number of candidate predictors, outcome events, and events per candidate predictor: | Yes | Yes | Yes | Yes | Yes |
| Describe how the model was developed (for example, with respect to modeling technique (eg, survival or logistic modeling), selection of predictors, and definition of risk groups): | Yes | Yes | Yes | Yes | Yes |
| Describe if and how the model was validated, either internally (eg, bootstrapping, cross-validation, split random sample) or externally (eg, temporal validation, geographic validation, different setting, different type of participants): | Yes | Yes | Yes | Yes | Yes |
| Describe the performance measures of the model, e.g. (re)calibration, discrimination, (re)classification, net profit and if adjusted for optimism: | Yes | Yes | Yes | Yes | Yes |
| Describe the participants who were excluded from the analysis: | Yes | Yes | Yes | Yes | Yes |
| Describe missing data on predictors and outcomes, as well as methods used for missing data: | Yes | Yes | Yes | Yes | Yes |
| 4.1 Was there a reasonable number of participants with the result? | Yes | Yes | Yes | Yes | Yes |
| 4.2 Were continuous and categorical predictors handled correctly? | Yes | Yes | Yes | Yes | Yes |
| 4.3 Were all enrolled participants included in the analysis? | Yes | Yes | Yes | Yes | Yes |
| 4.4 Were participants with missing data treated appropriately? | Yes | Yes | Yes | Yes | Yes |
| 4.5 Was selection of predictors based on univariate analysis avoided? | Yes | Yes | Yes | Yes | Yes |
| 4.6 Were the complexities of the data (eg censoring, competing risks, sampling of controls) adequately accounted for? | Yes | Yes | Yes | Yes | Yes |
| 4.7 Were relevant model performance measures adequately evaluated? | Yes | Yes | Yes | Yes | Yes |
| 4.8 Was model overfitting and optimism factored into model performance? | Yes | Yes | Yes | Yes | Yes |
| 4.9 Do the predictors and their assigned weights in the final model correspond to the results of the multivariate analysis? | Yes | Yes | Yes | Yes | Yes |
| **Risk of bias introduced by the analysis (low/high/Unclear)** | Low | Low | Low | Low | Low |
| **Step 4: General evaluation** | **-** | **-** | **-** | **-** | **-** |
| **Overall assessment of risk of bias** | Low | Low | Low | Low | Low |
| **General judgment of applicability** | Low | Low | Low | Low | Low |
|  |  |  |  |  |  |
|  |  |  |  |  |  |
| **Domain 1** | **Ávila-Ponce de León et al. (2021)** | **Ayoub et al. (2021)** | **Ayoub et al. (2022)** | **Bablani et al. (2021)** | **Babus et al. (2020)** |
| **A. Risk of bias** | **05-06-22** | **11-06-22** | **11-06-22** | **11-06-22** | **11-06-22** |
| 1.1 Were appropriate data sources used, e.g. data from cohort studies, RCTs or nested case-controls? | No | Yes | Yes | No | No |
| 1.2 Were all inclusions and exclusions of participants appropriate? | No | Unclear | Unclear | No | No |
| Risk of bias introduced by participant selection (low/high/unclear) | High | Low | Low | High | High |
| **B. Applicability** |  |  |  |  |  |
| Concern that included participants and setting do not match review question (low/high/unclear) | Unclear | Low | Low | Unclear | Unclear |
| **Domain 2** |  |  |  |  |  |
| **A. Risk of bias - predictors** |  |  |  |  |  |
| 2.1 Were predictors defined and evaluated in a similar way for all participants? | Yes | Yes | Yes | Yes | Yes |
| 2.2 Were predictor evaluations performed without knowing the outcome data? | Yes | Yes | Yes | Yes | Yes |
| 2.3 Are all the predictors available at the time the model is intended to be used? | Yes | Yes | Yes | Yes | Yes |
| Risk of bias introduced by the predictors or their assessment (low/high/unclear) | Low | Low | Low | Low | Low |
| **B. Applicability** |  |  |  |  |  |
| Concern that the definition, evaluation, or timing of predictors in the model do not match the review question (low/high/unclear) | Low | Low | Low | Low | Low |
| **Domain 3** |  |  |  |  |  |
| **A. Risk of bias - outcome** |  |  |  |  |  |
| Was the result properly determined? | Yes | Yes | Yes | Yes | Yes |
| Was a standard or prespecified outcome definition used? | Yes | Yes | Yes | Yes | Yes |
| Were predictors excluded from the outcome definition? | Yes | Yes | Yes | Yes | Yes |
| Was the outcome similarly defined and determined for all participants? | Yes | Yes | Yes | Yes | Yes |
| Was the outcome determined without knowing the predictor information? | Yes | Yes | Yes | Yes | Yes |
| Was the time interval between the evaluation of the predictor and the determination of the result adequate? | Yes | Yes | Yes | Yes | Yes |
| Risk of bias introduced by the result or its determination (low/high/unclear) | Low | Low | Low | Low | Low |
| **B. Applicability** |  |  |  |  |  |
| If a composite outcome was used, please describe the relative frequency/distribution of each contributing outcome: | NA | NA | NA | NA | NA |
| Concern that the outcome, its definition, timing, or determination does not match the review question (low/high/unclear) | Low | Low | Low | Low | Low |
| **Domain 4** |  |  |  |  |  |
| **A. Risk of bias** |  |  |  |  |  |
| Describe the number of participants, number of candidate predictors, outcome events, and events per candidate predictor: | Yes | Yes | Yes | Yes | Yes |
| Describe how the model was developed (for example, with respect to modeling technique (eg, survival or logistic modeling), selection of predictors, and definition of risk groups): | Yes | Yes | Yes | Yes | Yes |
| Describe if and how the model was validated, either internally (eg, bootstrapping, cross-validation, split random sample) or externally (eg, temporal validation, geographic validation, different setting, different type of participants): | Yes | Yes | Yes | Yes | Yes |
| Describe the performance measures of the model, e.g. (re)calibration, discrimination, (re)classification, net profit and if adjusted for optimism: | Yes | Yes | Yes | Yes | Yes |
| Describe the participants who were excluded from the analysis: | Yes | Yes | Yes | Yes | Yes |
| Describe missing data on predictors and outcomes, as well as methods used for missing data: | Yes | Yes | Yes | Yes | Yes |
| 4.1 Was there a reasonable number of participants with the result? | Yes | Yes | Yes | Yes | Yes |
| 4.2 Were continuous and categorical predictors handled correctly? | Yes | Yes | Yes | Yes | Yes |
| 4.3 Were all enrolled participants included in the analysis? | Yes | Yes | Yes | Yes | Yes |
| 4.4 Were participants with missing data treated appropriately? | Yes | Yes | Yes | Yes | Yes |
| 4.5 Was selection of predictors based on univariate analysis avoided? | Yes | Yes | Yes | Yes | Yes |
| 4.6 Were the complexities of the data (eg censoring, competing risks, sampling of controls) adequately accounted for? | Yes | Yes | Yes | Yes | Yes |
| 4.7 Were relevant model performance measures adequately evaluated? | Yes | Yes | Yes | Yes | Yes |
| 4.8 Was model overfitting and optimism factored into model performance? | Yes | Yes | Yes | Yes | Yes |
| 4.9 Do the predictors and their assigned weights in the final model correspond to the results of the multivariate analysis? | Yes | Yes | Yes | Yes | Yes |
| **Risk of bias introduced by the analysis (low/high/Unclear)** | Low | Low | Low | Low | Low |
| **Step 4: General evaluation** | There is not enough information related to the chosen population, so there is a risk of introducing bias in the selection | - | - | There is not enough information related to the chosen population, so there is a risk of introducing bias in the selection | There is not enough information related to the chosen population, so there is a risk of introducing bias in the selection |
| **Overall assessment of risk of bias** | High | Low | Low | High | High |
| **General judgment of applicability** | Low | Low | Low | Low | Low |
|  |  |  |  |  |  |
|  |  |  |  |  |  |
| **Domain 1** | **Badfar et al. (2021)** | **Bautista et al. (2021)** | **Barlow et al. (2021)** | **Barmpounakis et al. (2022)** | **Barnard et al. (2021)** |
| **A. Risk of bias** | **11-06-22** | **11-06-22** | **11-06-22** | **11-06-22** | **11-06-22** |
| 1.1 Were appropriate data sources used, e.g. data from cohort studies, RCTs or nested case-controls? | No | Yes | Yes | Yes | No |
| 1.2 Were all inclusions and exclusions of participants appropriate? | No | Unclear | Unclear | Unclear | No |
| Risk of bias introduced by participant selection (low/high/unclear) | High | Low | Low | Low | High |
| **B. Applicability** |  |  |  |  |  |
| Concern that included participants and setting do not match review question (low/high/unclear) | Unclear | Low | Low | Low | Unclear |
| **Domain 2** |  |  |  |  |  |
| **A. Risk of bias - predictors** |  |  |  |  |  |
| 2.1 Were predictors defined and evaluated in a similar way for all participants? | Yes | Yes | Yes | Yes | Yes |
| 2.2 Were predictor evaluations performed without knowing the outcome data? | Yes | Yes | Yes | Yes | Yes |
| 2.3 Are all the predictors available at the time the model is intended to be used? | Yes | Yes | Yes | Yes | Yes |
| Risk of bias introduced by the predictors or their assessment (low/high/unclear) | Low | Low | Low | Low | Low |
| **B. Applicability** |  |  |  |  |  |
| Concern that the definition, evaluation, or timing of predictors in the model do not match the review question (low/high/unclear) | Low | Low | Low | Low | Low |
| **Domain 3** |  |  |  |  |  |
| **A. Risk of bias - outcome** |  |  |  |  |  |
| Was the result properly determined? | Yes | Yes | Yes | Yes | Yes |
| Was a standard or prespecified outcome definition used? | Yes | Yes | Yes | Yes | Yes |
| Were predictors excluded from the outcome definition? | Yes | Yes | Yes | Yes | Yes |
| Was the outcome similarly defined and determined for all participants? | Yes | Yes | Yes | Yes | Yes |
| Was the outcome determined without knowing the predictor information? | Yes | Yes | Yes | Yes | Yes |
| Was the time interval between the evaluation of the predictor and the determination of the result adequate? | Yes | Yes | Yes | Yes | Yes |
| Risk of bias introduced by the result or its determination (low/high/unclear) | Low | Low | Low | Low | Low |
| **B. Applicability** |  |  |  |  |  |
| If a composite outcome was used, please describe the relative frequency/distribution of each contributing outcome: | NA | NA | NA | NA | Yes |
| Concern that the outcome, its definition, timing, or determination does not match the review question (low/high/unclear) | Low | Low | Low | Low | Low |
| **Domain 4** |  |  |  |  |  |
| **A. Risk of bias** |  |  |  |  |  |
| Describe the number of participants, number of candidate predictors, outcome events, and events per candidate predictor: | Yes | Yes | Yes | Yes | Yes |
| Describe how the model was developed (for example, with respect to modeling technique (eg, survival or logistic modeling), selection of predictors, and definition of risk groups): | Yes | Yes | Yes | Yes | Yes |
| Describe if and how the model was validated, either internally (eg, bootstrapping, cross-validation, split random sample) or externally (eg, temporal validation, geographic validation, different setting, different type of participants): | Yes | Yes | Yes | Yes | Yes |
| Describe the performance measures of the model, e.g. (re)calibration, discrimination, (re)classification, net profit and if adjusted for optimism: | Yes | Yes | Yes | Yes | Yes |
| Describe the participants who were excluded from the analysis: | Yes | Yes | Yes | Yes | Yes |
| Describe missing data on predictors and outcomes, as well as methods used for missing data: | Yes | Yes | Yes | Yes | Yes |
| 4.1 Was there a reasonable number of participants with the result? | Yes | Yes | Yes | Yes | Yes |
| 4.2 Were continuous and categorical predictors handled correctly? | Yes | Yes | Yes | Yes | Yes |
| 4.3 Were all enrolled participants included in the analysis? | Yes | Yes | Yes | Yes | Yes |
| 4.4 Were participants with missing data treated appropriately? | Yes | Yes | Yes | Yes | Yes |
| 4.5 Was selection of predictors based on univariate analysis avoided? | Yes | Yes | Yes | Yes | Yes |
| 4.6 Were the complexities of the data (eg censoring, competing risks, sampling of controls) adequately accounted for? | Yes | Yes | Yes | Yes | Yes |
| 4.7 Were relevant model performance measures adequately evaluated? | Yes | Yes | Yes | Yes | Yes |
| 4.8 Was model overfitting and optimism factored into model performance? | Yes | Yes | Yes | Yes | Yes |
| 4.9 Do the predictors and their assigned weights in the final model correspond to the results of the multivariate analysis? | Yes | Yes | Yes | Yes | Yes |
| **Risk of bias introduced by the analysis (low/high/Unclear)** | Low | Low | Low | Low | Low |
| **Step 4: General evaluation** | There is not enough information related to the chosen population, so there is a risk of introducing bias in the selection | - | - | - | There is not enough information related to the chosen population, so there is a risk of introducing bias in the selection |
| **Overall assessment of risk of bias** | High | Low | Low | Low | High |
| **General judgment of applicability** | Low | Low | Low | Low | Low |
|  |  |  |  |  |  |
|  |  |  |  |  |  |
| **Domain 1** | **Barnard et al. (2021)** | **Barreiro et al. (2021)** | **Bartsch et al. (2020)** | **Bartsch et al. (2021) (1)** | **Bartsch et al. (2021) (2)** |
| **A. Risk of bias** | **11-06-22** | **11-06-22** | **11-06-22** | **11-06-22** | **11-06-22** |
| 1.1 Were appropriate data sources used, e.g. data from cohort studies, RCTs or nested case-controls? | No | No | No | No | No |
| 1.2 Were all inclusions and exclusions of participants appropriate? | No | No | No | No | No |
| Risk of bias introduced by participant selection (low/high/unclear) | High | High | High | High | High |
| **B. Applicability** |  |  |  |  |  |
| Concern that included participants and setting do not match review question (low/high/unclear) | Unclear | Unclear | Unclear | Unclear | Unclear |
| **Domain 2** |  |  |  |  |  |
| **A. Risk of bias - predictors** |  |  |  |  |  |
| 2.1 Were predictors defined and evaluated in a similar way for all participants? | Yes | Yes | Yes | Yes | Yes |
| 2.2 Were predictor evaluations performed without knowing the outcome data? | Yes | Yes | Yes | Yes | Yes |
| 2.3 Are all the predictors available at the time the model is intended to be used? | Yes | Yes | Yes | Yes | Yes |
| Risk of bias introduced by the predictors or their assessment (low/high/unclear) | Low | Low | Low | Low | Low |
| **B. Applicability** |  |  |  |  |  |
| Concern that the definition, evaluation, or timing of predictors in the model do not match the review question (low/high/unclear) | Low | Unclear | Low | Low | Low |
| **Domain 3** |  |  |  |  |  |
| **A. Risk of bias - outcome** |  |  |  |  |  |
| Was the result properly determined? | Yes | Yes | Yes | Yes | Yes |
| Was a standard or prespecified outcome definition used? | Yes | Yes | Yes | Yes | Yes |
| Were predictors excluded from the outcome definition? | Yes | Yes | Yes | Yes | Yes |
| Was the outcome similarly defined and determined for all participants? | Yes | Yes | Yes | Yes | Yes |
| Was the outcome determined without knowing the predictor information? | Yes | Yes | Yes | Yes | Yes |
| Was the time interval between the evaluation of the predictor and the determination of the result adequate? | Yes | Yes | Yes | Yes | Yes |
| Risk of bias introduced by the result or its determination (low/high/unclear) | Low | Low | Low | Low | Low |
| **B. Applicability** |  |  |  |  |  |
| If a composite outcome was used, please describe the relative frequency/distribution of each contributing outcome: | Yes | Yes | NA | NA | Yes |
| Concern that the outcome, its definition, timing, or determination does not match the review question (low/high/unclear) | Low | Low | Low | Low | Low |
| **Domain 4** |  |  |  |  |  |
| **A. Risk of bias** |  |  |  |  |  |
| Describe the number of participants, number of candidate predictors, outcome events, and events per candidate predictor: | Yes | Yes | Yes | Yes | Yes |
| Describe how the model was developed (for example, with respect to modeling technique (eg, survival or logistic modeling), selection of predictors, and definition of risk groups): | Yes | Yes | Yes | Yes | Yes |
| Describe if and how the model was validated, either internally (eg, bootstrapping, cross-validation, split random sample) or externally (eg, temporal validation, geographic validation, different setting, different type of participants): | Yes | Yes | Yes | Yes | Yes |
| Describe the performance measures of the model, e.g. (re)calibration, discrimination, (re)classification, net profit and if adjusted for optimism: | Yes | Yes | Yes | Yes | Yes |
| Describe the participants who were excluded from the analysis: | Yes | Yes | Yes | Yes | Yes |
| Describe missing data on predictors and outcomes, as well as methods used for missing data: | Yes | Yes | Yes | Yes | Yes |
| 4.1 Was there a reasonable number of participants with the result? | Yes | Yes | Yes | Yes | Yes |
| 4.2 Were continuous and categorical predictors handled correctly? | Yes | Yes | Yes | Yes | Yes |
| 4.3 Were all enrolled participants included in the analysis? | Yes | Yes | Yes | Yes | Yes |
| 4.4 Were participants with missing data treated appropriately? | Yes | Yes | Yes | Yes | Yes |
| 4.5 Was selection of predictors based on univariate analysis avoided? | Yes | Yes | Yes | Yes | Yes |
| 4.6 Were the complexities of the data (eg censoring, competing risks, sampling of controls) adequately accounted for? | Yes | Yes | Yes | Yes | Yes |
| 4.7 Were relevant model performance measures adequately evaluated? | Yes | Yes | Yes | Yes | Yes |
| 4.8 Was model overfitting and optimism factored into model performance? | Yes | Yes | Yes | Yes | Yes |
| 4.9 Do the predictors and their assigned weights in the final model correspond to the results of the multivariate analysis? | Yes | Yes | Yes | Yes | Yes |
| **Risk of bias introduced by the analysis (low/high/Unclear)** | Low | Low | Low | Low | Low |
| **Step 4: General evaluation** | There is not enough information related to the chosen population, so there is a risk of introducing bias in the selection | There is not enough information related to the chosen population, so there is a risk of introducing bias in the selection | There is not enough information related to the chosen population, so there is a risk of introducing bias in the selection | There is not enough information related to the chosen population, so there is a risk of introducing bias in the selection | There is not enough information related to the chosen population, so there is a risk of introducing bias in the selection |
| **Overall assessment of risk of bias** | High | High | High | High | High |
| **General judgment of applicability** | Low | Unclear | Low | Low | Low |
|  |  |  |  |  |  |
|  |  |  |  |  |  |
| **Domain 1** | **Bauer et al. (2021)** | **Below et al. (2021)** | **Berec et al. (2022)** | **Berkane et al. (2021)** | **Bertsimas et al. (2021)** |
| **A. Risk of bias** | **11-06-22** | **11-06-22** | **11-06-22** | **11-06-22** | **11-06-22** |
| 1.1 Were appropriate data sources used, e.g. data from cohort studies, RCTs or nested case-controls? | No | No | Yes | Yes | No |
| 1.2 Were all inclusions and exclusions of participants appropriate? | No | No | Yes | Yes | No |
| Risk of bias introduced by participant selection (low/high/unclear) | High | High | Low | Low | High |
| **B. Applicability** |  |  |  |  |  |
| Concern that included participants and setting do not match review question (low/high/unclear) | Unclear | Unclear | Low | Low | Unclear |
| **Domain 2** |  |  |  |  |  |
| **A. Risk of bias - predictors** |  |  |  |  |  |
| 2.1 Were predictors defined and evaluated in a similar way for all participants? | Yes | Yes | Yes | Yes | Yes |
| 2.2 Were predictor evaluations performed without knowing the outcome data? | Yes | Yes | Yes | Yes | Yes |
| 2.3 Are all the predictors available at the time the model is intended to be used? | Yes | Yes | Yes | Yes | Yes |
| Risk of bias introduced by the predictors or their assessment (low/high/unclear) | Low | Low | Low | Low | Low |
| **B. Applicability** |  |  |  |  |  |
| Concern that the definition, evaluation, or timing of predictors in the model do not match the review question (low/high/unclear) | Low | Low | Low | Low | Low |
| **Domain 3** |  |  |  |  |  |
| **A. Risk of bias - outcome** |  |  |  |  |  |
| Was the result properly determined? | Yes | Yes | Yes | Yes | Yes |
| Was a standard or prespecified outcome definition used? | Yes | Yes | Yes | Yes | Yes |
| Were predictors excluded from the outcome definition? | Yes | Yes | Yes | Yes | Yes |
| Was the outcome similarly defined and determined for all participants? | Yes | Yes | Yes | Yes | Yes |
| Was the outcome determined without knowing the predictor information? | Yes | Yes | Yes | Yes | Yes |
| Was the time interval between the evaluation of the predictor and the determination of the result adequate? | Yes | Yes | Yes | Yes | Yes |
| Risk of bias introduced by the result or its determination (low/high/unclear) | Low | Low | Low | Low | Low |
| **B. Applicability** |  |  |  |  |  |
| If a composite outcome was used, please describe the relative frequency/distribution of each contributing outcome: | NA | Yes | Yes | Yes | Yes |
| Concern that the outcome, its definition, timing, or determination does not match the review question (low/high/unclear) | Low | Low | Low | Low | Low |
| **Domain 4** |  |  |  |  |  |
| **A. Risk of bias** |  |  |  |  |  |
| Describe the number of participants, number of candidate predictors, outcome events, and events per candidate predictor: | Yes | Yes | Yes | Yes | Yes |
| Describe how the model was developed (for example, with respect to modeling technique (eg, survival or logistic modeling), selection of predictors, and definition of risk groups): | Yes | Yes | Yes | Yes | Yes |
| Describe if and how the model was validated, either internally (eg, bootstrapping, cross-validation, split random sample) or externally (eg, temporal validation, geographic validation, different setting, different type of participants): | Yes | Yes | Yes | Yes | Yes |
| Describe the performance measures of the model, e.g. (re)calibration, discrimination, (re)classification, net profit and if adjusted for optimism: | Yes | Yes | Yes | Yes | Yes |
| Describe the participants who were excluded from the analysis: | Yes | Yes | Yes | Yes | Yes |
| Describe missing data on predictors and outcomes, as well as methods used for missing data: | Yes | Yes | Yes | Yes | Yes |
| 4.1 Was there a reasonable number of participants with the result? | Yes | Yes | Yes | Yes | Yes |
| 4.2 Were continuous and categorical predictors handled correctly? | Yes | Yes | Yes | Yes | Yes |
| 4.3 Were all enrolled participants included in the analysis? | Yes | Yes | Yes | Yes | Yes |
| 4.4 Were participants with missing data treated appropriately? | Yes | Yes | Yes | Yes | Yes |
| 4.5 Was selection of predictors based on univariate analysis avoided? | Yes | Yes | Yes | Yes | Yes |
| 4.6 Were the complexities of the data (eg censoring, competing risks, sampling of controls) adequately accounted for? | Yes | Yes | Yes | Yes | Yes |
| 4.7 Were relevant model performance measures adequately evaluated? | Yes | Yes | Yes | Yes | Yes |
| 4.8 Was model overfitting and optimism factored into model performance? | Yes | Yes | Yes | Yes | Yes |
| 4.9 Do the predictors and their assigned weights in the final model correspond to the results of the multivariate analysis? | Yes | Yes | Yes | Yes | Yes |
| **Risk of bias introduced by the analysis (low/high/Unclear)** | Low | Low | Low | Low | Low |
| **Step 4: General evaluation** | - | There is not enough information related to the chosen population, so there is a risk of introducing bias in the selection | - | - | There is not enough information related to the chosen population, so there is a risk of introducing bias in the selection |
| **Overall assessment of risk of bias** | Low | High | Low | Low | High |
| **General judgment of applicability** | Low | Low | Low | Low | Low |
|  |  |  |  |  |  |
|  |  |  |  |  |  |
| **Domain 1** | **Bhattacharya et al. (2021)** | **Bianchin et al. (2021)** | **Bilinski et al. (2021) (1)** | **Bilinski et al. (2021) (2)** | **Booton et al. (2021)** |
| **A. Risk of bias** | **11-06-22** | **11-06-22** | **11-06-22** | **11-06-22** | **11-06-22** |
| 1.1 Were appropriate data sources used, e.g. data from cohort studies, RCTs or nested case-controls? | Yes | Yes | Yes | Yes | Unclear |
| 1.2 Were all inclusions and exclusions of participants appropriate? | Unclear | Yes | Yes | Yes | No |
| Risk of bias introduced by participant selection (low/high/unclear) | Unclear | Low | Low | Low | High |
| **B. Applicability** |  |  |  |  |  |
| Concern that included participants and setting do not match review question (low/high/unclear) | Low | Low | Low | Low | High |
| **Domain 2** |  |  |  |  |  |
| **A. Risk of bias - predictors** |  |  |  |  |  |
| 2.1 Were predictors defined and evaluated in a similar way for all participants? | Yes | Yes | Yes | Yes | Yes |
| 2.2 Were predictor evaluations performed without knowing the outcome data? | Yes | Yes | Yes | Yes | Yes |
| 2.3 Are all the predictors available at the time the model is intended to be used? | Yes | Yes | Yes | Yes | Yes |
| Risk of bias introduced by the predictors or their assessment (low/high/unclear) | Low | Low | Low | Low | Low |
| **B. Applicability** |  |  |  |  |  |
| Concern that the definition, evaluation, or timing of predictors in the model do not match the review question (low/high/unclear) | Low | Low | Low | Low | Low |
| **Domain 3** |  |  |  |  |  |
| **A. Risk of bias - outcome** |  |  |  |  |  |
| Was the result properly determined? | Yes | Yes | Yes | Yes | Yes |
| Was a standard or prespecified outcome definition used? | Yes | Yes | Yes | Yes | Yes |
| Were predictors excluded from the outcome definition? | Yes | Yes | Yes | Yes | Yes |
| Was the outcome similarly defined and determined for all participants? | Yes | Yes | Yes | Yes | Yes |
| Was the outcome determined without knowing the predictor information? | Yes | Yes | Yes | Yes | Yes |
| Was the time interval between the evaluation of the predictor and the determination of the result adequate? | Yes | Yes | Yes | Yes | Yes |
| Risk of bias introduced by the result or its determination (low/high/unclear) | Low | Low | Low | Low | Low |
| **B. Applicability** |  |  |  |  |  |
| If a composite outcome was used, please describe the relative frequency/distribution of each contributing outcome: | Yes | Yes | Yes | Yes | NA |
| Concern that the outcome, its definition, timing, or determination does not match the review question (low/high/unclear) | Low | Low | Low | Low | Low |
| **Domain 4** |  |  |  |  |  |
| **A. Risk of bias** |  |  |  |  |  |
| Describe the number of participants, number of candidate predictors, outcome events, and events per candidate predictor: | Yes | Yes | Yes | Yes | Yes |
| Describe how the model was developed (for example, with respect to modeling technique (eg, survival or logistic modeling), selection of predictors, and definition of risk groups): | Yes | Yes | Yes | Yes | Yes |
| Describe if and how the model was validated, either internally (eg, bootstrapping, cross-validation, split random sample) or externally (eg, temporal validation, geographic validation, different setting, different type of participants): | Yes | Yes | Yes | Yes | Yes |
| Describe the performance measures of the model, e.g. (re)calibration, discrimination, (re)classification, net profit and if adjusted for optimism: | Yes | Yes | Yes | Yes | Yes |
| Describe the participants who were excluded from the analysis: | Yes | Yes | Yes | Yes | Yes |
| Describe missing data on predictors and outcomes, as well as methods used for missing data: | Yes | Yes | Yes | Yes | Yes |
| 4.1 Was there a reasonable number of participants with the result? | Yes | Yes | Yes | Yes | Yes |
| 4.2 Were continuous and categorical predictors handled correctly? | Yes | Yes | Yes | Yes | Yes |
| 4.3 Were all enrolled participants included in the analysis? | Yes | Yes | Yes | Yes | Yes |
| 4.4 Were participants with missing data treated appropriately? | Yes | Yes | Yes | Yes | Yes |
| 4.5 Was selection of predictors based on univariate analysis avoided? | Yes | Yes | Yes | Yes | Yes |
| 4.6 Were the complexities of the data (eg censoring, competing risks, sampling of controls) adequately accounted for? | Yes | Yes | Yes | Yes | Yes |
| 4.7 Were relevant model performance measures adequately evaluated? | Yes | Yes | Yes | Yes | Yes |
| 4.8 Was model overfitting and optimism factored into model performance? | Yes | Yes | Yes | Yes | Yes |
| 4.9 Do the predictors and their assigned weights in the final model correspond to the results of the multivariate analysis? | Yes | Yes | Yes | Yes | Yes |
| **Risk of bias introduced by the analysis (low/high/Unclear)** | Low | Low | Low | Low | Low |
| **Step 4: General evaluation** | **-** | **-** | **-** | **-** | This article does not give a broad definition of the data used. Likewise, the model used does not show as much detail |
| **Overall assessment of risk of bias** | Low | Low | Low | Low | High |
| **General judgment of applicability** | Low | Low | Low | Low | Low |
|  |  |  |  |  |  |
|  |  |  |  |  |  |
| **Domain 1** | **Borchering et al. (2021)** | **Borchering et al. (2022)** | **Bosseti et al. (2022)** | **Bosseti et al. (2021)** | **Bosseti et al. (2022)** |
| **A. Risk of bias** | **11-06-22** | **11-06-22** | **11-06-22** | **11-06-22** | **11-06-22** |
| 1.1 Were appropriate data sources used, e.g. data from cohort studies, RCTs or nested case-controls? | Unclear | Unclear | No | Unclear | Unclear |
| 1.2 Were all inclusions and exclusions of participants appropriate? | No | No | No | No | No |
| Risk of bias introduced by participant selection (low/high/unclear) | High | High | High | High | High |
| **B. Applicability** |  |  |  |  |  |
| Concern that included participants and setting do not match review question (low/high/unclear) | High | High | Low | Low | Low |
| **Domain 2** |  |  |  |  |  |
| **A. Risk of bias - predictors** |  |  |  |  |  |
| 2.1 Were predictors defined and evaluated in a similar way for all participants? | Yes | Yes | Yes | Yes | Yes |
| 2.2 Were predictor evaluations performed without knowing the outcome data? | Yes | Yes | Yes | Yes | Yes |
| 2.3 Are all the predictors available at the time the model is intended to be used? | Yes | Yes | Yes | Yes | Yes |
| Risk of bias introduced by the predictors or their assessment (low/high/unclear) | Low | Low | Low | Low | Low |
| **B. Applicability** |  |  |  |  |  |
| Concern that the definition, evaluation, or timing of predictors in the model do not match the review question (low/high/unclear) | Low | Low | Low | Low | Low |
| **Domain 3** |  |  |  |  |  |
| **A. Risk of bias - outcome** |  |  |  |  |  |
| Was the result properly determined? | Yes | Yes | Yes | Yes | Yes |
| Was a standard or prespecified outcome definition used? | Yes | Yes | Yes | Yes | Yes |
| Were predictors excluded from the outcome definition? | Yes | Yes | Yes | Yes | Yes |
| Was the outcome similarly defined and determined for all participants? | Yes | Yes | Yes | Yes | Yes |
| Was the outcome determined without knowing the predictor information? | Yes | Yes | Yes | Yes | Yes |
| Was the time interval between the evaluation of the predictor and the determination of the result adequate? | Yes | Yes | Yes | Yes | Yes |
| Risk of bias introduced by the result or its determination (low/high/unclear) | Low | Low | Low | Low | Low |
| **B. Applicability** |  |  |  |  |  |
| If a composite outcome was used, please describe the relative frequency/distribution of each contributing outcome: | NA | NA | NA | NA | NA |
| Concern that the outcome, its definition, timing, or determination does not match the review question (low/high/unclear) | Low | Low | Low | Low | Low |
| **Domain 4** |  |  |  |  |  |
| **A. Risk of bias** |  |  |  |  |  |
| Describe the number of participants, number of candidate predictors, outcome events, and events per candidate predictor: | Yes | Yes | Yes | Yes | Yes |
| Describe how the model was developed (for example, with respect to modeling technique (eg, survival or logistic modeling), selection of predictors, and definition of risk groups): | Yes | Yes | Yes | Yes | Yes |
| Describe if and how the model was validated, either internally (eg, bootstrapping, cross-validation, split random sample) or externally (eg, temporal validation, geographic validation, different setting, different type of participants): | Yes | Yes | Yes | Yes | Yes |
| Describe the performance measures of the model, e.g. (re)calibration, discrimination, (re)classification, net profit and if adjusted for optimism: | Yes | Yes | Yes | Yes | Yes |
| Describe the participants who were excluded from the analysis: | Yes | Yes | Yes | Yes | Yes |
| Describe missing data on predictors and outcomes, as well as methods used for missing data: | Yes | Yes | Yes | Yes | Yes |
| 4.1 Was there a reasonable number of participants with the result? | Yes | Yes | Yes | Yes | Yes |
| 4.2 Were continuous and categorical predictors handled correctly? | Yes | Yes | Yes | Yes | Yes |
| 4.3 Were all enrolled participants included in the analysis? | Yes | Yes | Yes | Yes | Yes |
| 4.4 Were participants with missing data treated appropriately? | Yes | Yes | Yes | Yes | Yes |
| 4.5 Was selection of predictors based on univariate analysis avoided? | Yes | Yes | Yes | Yes | Yes |
| 4.6 Were the complexities of the data (eg censoring, competing risks, sampling of controls) adequately accounted for? | Yes | Yes | Yes | Yes | Yes |
| 4.7 Were relevant model performance measures adequately evaluated? | Yes | Yes | Yes | Yes | Yes |
| 4.8 Was model overfitting and optimism factored into model performance? | Yes | Yes | Yes | Yes | Yes |
| 4.9 Do the predictors and their assigned weights in the final model correspond to the results of the multivariate analysis? | Yes | Yes | Yes | Yes | Yes |
| **Risk of bias introduced by the analysis (low/high/Unclear)** | Low | Low | Low | Low | Low |
| **Step 4: General evaluation** | This article does not give a broad definition of the data used. Likewise, the model used does not show as much detail | This article does not give a broad definition of the data used. Likewise, the model used does not show so much detail | This study does not define the source of the data, although there are some established criteria to introduce in the model | This study does not define the source of the data, although there are some established criteria to introduce in the model | This study does not define the source of the data, although there are some established criteria to introduce in the model |
| **Overall assessment of risk of bias** | High | High | High | High | High |
| **General judgment of applicability** | Low | Low | Low | Low | Low |
|  |  |  |  |  |  |
|  |  |  |  |  |  |
| **Domain 1** | **Bousquet et al. (2022)** | **Bowie et al. (2021)** | **Bracis et al. (2022)** | **Braun et al. (2020)** | **Brown et al. (2021)** |
| **A. Risk of bias** | **11-06-22** | **11-06-22** | **11-06-22** | **11-06-22** | **11-06-22** |
| 1.1 Were appropriate data sources used, e.g. data from cohort studies, RCTs or nested case-controls? | Yes | Yes | Yes | No | No |
| 1.2 Were all inclusions and exclusions of participants appropriate? | Unclear | Unclear | Yes | No | No |
| Risk of bias introduced by participant selection (low/high/unclear) | Unclear | Unclear | Low | High | High |
| **B. Applicability** |  |  |  |  |  |
| Concern that included participants and setting do not match review question (low/high/unclear) | Low | Low | Low | High | High |
| **Domain 2** |  |  |  |  |  |
| **A. Risk of bias - predictors** |  |  |  |  |  |
| 2.1 Were predictors defined and evaluated in a similar way for all participants? | Yes | Yes | Yes | Yes | Yes |
| 2.2 Were predictor evaluations performed without knowing the outcome data? | Yes | Yes | Yes | Yes | Yes |
| 2.3 Are all the predictors available at the time the model is intended to be used? | Yes | Yes | Yes | Yes | Yes |
| Risk of bias introduced by the predictors or their assessment (low/high/unclear) | Low | Low | Low | Low | Low |
| **B. Applicability** |  |  |  |  |  |
| Concern that the definition, evaluation, or timing of predictors in the model do not match the review question (low/high/unclear) | Low | Low | Low | Low | Low |
| **Domain 3** |  |  |  |  |  |
| **A. Risk of bias - outcome** |  |  |  |  |  |
| Was the result properly determined? | Yes | Yes | Yes | Yes | Yes |
| Was a standard or prespecified outcome definition used? | Yes | Yes | Yes | Yes | Yes |
| Were predictors excluded from the outcome definition? | Yes | Yes | Yes | Yes | Yes |
| Was the outcome similarly defined and determined for all participants? | Yes | Yes | Yes | Yes | Yes |
| Was the outcome determined without knowing the predictor information? | Yes | Yes | Yes | Yes | Yes |
| Was the time interval between the evaluation of the predictor and the determination of the result adequate? | Yes | Yes | Yes | Yes | Yes |
| Risk of bias introduced by the result or its determination (low/high/unclear) | Low | Low | Low | Low | Low |
| **B. Applicability** |  |  |  |  |  |
| If a composite outcome was used, please describe the relative frequency/distribution of each contributing outcome: | NA | Yes | Yes | Yes | Yes |
| Concern that the outcome, its definition, timing, or determination does not match the review question (low/high/unclear) | Low | Low | Low | Low | Low |
| **Domain 4** |  |  |  |  |  |
| **A. Risk of bias** |  |  |  |  |  |
| Describe the number of participants, number of candidate predictors, outcome events, and events per candidate predictor: | Yes | Yes | Yes | Yes | Yes |
| Describe how the model was developed (for example, with respect to modeling technique (eg, survival or logistic modeling), selection of predictors, and definition of risk groups): | Yes | Yes | Yes | Yes | Yes |
| Describe if and how the model was validated, either internally (eg, bootstrapping, cross-validation, split random sample) or externally (eg, temporal validation, geographic validation, different setting, different type of participants): | Yes | Yes | Yes | Yes | Yes |
| Describe the performance measures of the model, e.g. (re)calibration, discrimination, (re)classification, net profit and if adjusted for optimism: | Yes | Yes | Yes | Yes | Yes |
| Describe the participants who were excluded from the analysis: | Yes | Yes | Yes | Yes | Yes |
| Describe missing data on predictors and outcomes, as well as methods used for missing data: | Yes | Yes | Yes | Yes | Yes |
| 4.1 Was there a reasonable number of participants with the result? | Yes | Yes | Yes | Yes | Yes |
| 4.2 Were continuous and categorical predictors handled correctly? | Yes | Yes | Yes | Yes | Yes |
| 4.3 Were all enrolled participants included in the analysis? | Yes | Yes | Yes | Yes | Yes |
| 4.4 Were participants with missing data treated appropriately? | Yes | Yes | Yes | Yes | Yes |
| 4.5 Was selection of predictors based on univariate analysis avoided? | Yes | Yes | Yes | Yes | Yes |
| 4.6 Were the complexities of the data (eg censoring, competing risks, sampling of controls) adequately accounted for? | Yes | Yes | Yes | Yes | Yes |
| 4.7 Were relevant model performance measures adequately evaluated? | Yes | Yes | Yes | Yes | Yes |
| 4.8 Was model overfitting and optimism factored into model performance? | Yes | Yes | Yes | Yes | Yes |
| 4.9 Do the predictors and their assigned weights in the final model correspond to the results of the multivariate analysis? | Yes | Yes | Yes | Yes | Yes |
| **Risk of bias introduced by the analysis (low/high/Unclear)** | Low | Low | Low | Low | Low |
| **Step 4: General evaluation** | There is not enough information related to the chosen population, so there is a risk of introducing bias in the selection | The inclusion criteria are not clear | - | Nothing is mentioned about the population or its source | Nothing is mentioned about the population or its source |
| **Overall assessment of risk of bias** | Unclear | Unclear | Low | High | High |
| **General judgment of applicability** | Low | Low | Low | High | High |
|  |  |  |  |  |  |
|  |  |  |  |  |  |
| **Domain 1** | **Brüningk et al. (2022)** | **Bu et al. (2021)** | **Bubar et al. (2021)** | **Buckner et al. (2021)** | **Bugalia et al. (2022)** |
| **A. Risk of bias** | **11-06-22** | **11-06-22** | **11-06-22** | **11-06-22** | **11-06-22** |
| 1.1 Were appropriate data sources used, e.g. data from cohort studies, RCTs or nested case-controls? | Yes | No | No | No | Yes |
| 1.2 Were all inclusions and exclusions of participants appropriate? | Yes | No | No | No | Yes |
| Risk of bias introduced by participant selection (low/high/unclear) | Low | High | High | High | Low |
| **B. Applicability** |  |  |  |  |  |
| Concern that included participants and setting do not match review question (low/high/unclear) | Low | Low | Low | Low | Low |
| **Domain 2** |  |  |  |  |  |
| **A. Risk of bias - predictors** |  |  |  |  |  |
| 2.1 Were predictors defined and evaluated in a similar way for all participants? | Yes | Yes | Yes | Yes | Yes |
| 2.2 Were predictor evaluations performed without knowing the outcome data? | Yes | Yes | Yes | Yes | Yes |
| 2.3 Are all the predictors available at the time the model is intended to be used? | Yes | Yes | Yes | Yes | Yes |
| Risk of bias introduced by the predictors or their assessment (low/high/unclear) | Low | Low | Low | Low | Low |
| **B. Applicability** |  |  |  |  |  |
| Concern that the definition, evaluation, or timing of predictors in the model do not match the review question (low/high/unclear) | Low | Low | Low | Low | Low |
| **Domain 3** |  |  |  |  |  |
| **A. Risk of bias - outcome** |  |  |  |  |  |
| Was the result properly determined? | Yes | Yes | Yes | Yes | Yes |
| Was a standard or prespecified outcome definition used? | Yes | Yes | Yes | Yes | Yes |
| Were predictors excluded from the outcome definition? | Yes | Yes | Yes | Yes | Yes |
| Was the outcome similarly defined and determined for all participants? | Yes | Yes | Yes | Yes | Yes |
| Was the outcome determined without knowing the predictor information? | Yes | Yes | Yes | Yes | Yes |
| Was the time interval between the evaluation of the predictor and the determination of the result adequate? | Yes | Yes | Yes | Yes | Yes |
| Risk of bias introduced by the result or its determination (low/high/unclear) | Low | Low | Low | Low | Low |
| **B. Applicability** |  |  |  |  |  |
| If a composite outcome was used, please describe the relative frequency/distribution of each contributing outcome: | Yes | Yes | Yes | Yes | Yes |
| Concern that the outcome, its definition, timing, or determination does not match the review question (low/high/unclear) | Low | Low | Low | Low | Low |
| **Domain 4** |  |  |  |  |  |
| **A. Risk of bias** |  |  |  |  |  |
| Describe the number of participants, number of candidate predictors, outcome events, and events per candidate predictor: | Yes | Yes | Yes | Yes | Yes |
| Describe how the model was developed (for example, with respect to modeling technique (eg, survival or logistic modeling), selection of predictors, and definition of risk groups): | Yes | Yes | Yes | Yes | Yes |
| Describe if and how the model was validated, either internally (eg, bootstrapping, cross-validation, split random sample) or externally (eg, temporal validation, geographic validation, different setting, different type of participants): | Yes | Yes | Yes | Yes | Yes |
| Describe the performance measures of the model, e.g. (re)calibration, discrimination, (re)classification, net profit and if adjusted for optimism: | Yes | Yes | Yes | Yes | Yes |
| Describe the participants who were excluded from the analysis: | Yes | Yes | Yes | Yes | Yes |
| Describe missing data on predictors and outcomes, as well as methods used for missing data: | Yes | Yes | Yes | Yes | Yes |
| 4.1 Was there a reasonable number of participants with the result? | Yes | Yes | Yes | Yes | Yes |
| 4.2 Were continuous and categorical predictors handled correctly? | Yes | Yes | Yes | Yes | Yes |
| 4.3 Were all enrolled participants included in the analysis? | Yes | Yes | Yes | Yes | Yes |
| 4.4 Were participants with missing data treated appropriately? | Yes | Yes | Yes | Yes | Yes |
| 4.5 Was selection of predictors based on univariate analysis avoided? | Yes | Yes | Yes | Yes | Yes |
| 4.6 Were the complexities of the data (eg censoring, competing risks, sampling of controls) adequately accounted for? | Yes | Yes | Yes | Yes | Yes |
| 4.7 Were relevant model performance measures adequately evaluated? | Yes | Yes | Yes | Yes | Yes |
| 4.8 Was model overfitting and optimism factored into model performance? | Yes | Yes | Yes | Yes | Yes |
| 4.9 Do the predictors and their assigned weights in the final model correspond to the results of the multivariate analysis? | Yes | Yes | Yes | Yes | Yes |
| **Risk of bias introduced by the analysis (low/high/Unclear)** | Low | Low | Low | Low | Low |
| **Step 4: General evaluation** | **-** | Nothing is mentioned about the population or its source | Nothing is mentioned about the population or its source | Nothing is mentioned about the population or its source | **-** |
| **Overall assessment of risk of bias** | Low | High | High | High | Low |
| **General judgment of applicability** | Low | Low | Low | Low | Low |
|  |  |  |  |  |  |
|  |  |  |  |  |  |
| **Domain 1** | **Buhat et al. (2021)** | **Buonomo et al. (2021)** | **Hohenegger et al. (2021)** | **Caetano et al. (2021)** | **Cai et al. (2022)** |
| **A. Risk of bias** | **11-06-22** | **11-06-22** | **11-06-22** | **12-06-22** | **12-06-22** |
| 1.1 Were appropriate data sources used, e.g. data from cohort studies, RCTs or nested case-controls? | Yes | Yes | Yes | Yes | Yes |
| 1.2 Were all inclusions and exclusions of participants appropriate? | Yes | No | Yes | Yes | Yes |
| Risk of bias introduced by participant selection (low/high/unclear) | Low | Low | Low | Low | Low |
| **B. Applicability** |  |  |  |  |  |
| Concern that included participants and setting do not match review question (low/high/unclear) | Low | Low | Low | Low | Low |
| **Domain 2** |  |  |  |  |  |
| **A. Risk of bias - predictors** |  |  |  |  |  |
| 2.1 Were predictors defined and evaluated in a similar way for all participants? | Yes | Yes | Yes | Yes | Yes |
| 2.2 Were predictor evaluations performed without knowing the outcome data? | Yes | Yes | Yes | Yes | Yes |
| 2.3 Are all the predictors available at the time the model is intended to be used? | Yes | Yes | Yes | Yes | Yes |
| Risk of bias introduced by the predictors or their assessment (low/high/unclear) | Low | Low | Low | Low | Low |
| **B. Applicability** |  |  |  |  |  |
| Concern that the definition, evaluation, or timing of predictors in the model do not match the review question (low/high/unclear) | Low | Low | Low | Low | Low |
| **Domain 3** |  |  |  |  |  |
| **A. Risk of bias - outcome** |  |  |  |  |  |
| Was the result properly determined? | Yes | Yes | Yes | Yes | Yes |
| Was a standard or prespecified outcome definition used? | Yes | Yes | Yes | Yes | Yes |
| Were predictors excluded from the outcome definition? | Yes | Yes | Yes | Yes | Yes |
| Was the outcome similarly defined and determined for all participants? | Yes | Yes | Yes | Yes | Yes |
| Was the outcome determined without knowing the predictor information? | Yes | Yes | Yes | Yes | Yes |
| Was the time interval between the evaluation of the predictor and the determination of the result adequate? | Yes | Yes | Yes | Yes | Yes |
| Risk of bias introduced by the result or its determination (low/high/unclear) | Low | Low | Low | Low | Low |
| **B. Applicability** |  |  |  |  |  |
| If a composite outcome was used, please describe the relative frequency/distribution of each contributing outcome: | Yes | NA | Yes | Yes | Yes |
| Concern that the outcome, its definition, timing, or determination does not match the review question (low/high/unclear) | Low | Low | Low | Low | Low |
| **Domain 4** |  |  |  |  |  |
| **A. Risk of bias** |  |  |  |  |  |
| Describe the number of participants, number of candidate predictors, outcome events, and events per candidate predictor: | Yes | Yes | Yes | Yes | Yes |
| Describe how the model was developed (for example, with respect to modeling technique (eg, survival or logistic modeling), selection of predictors, and definition of risk groups): | Yes | Yes | Yes | Yes | Yes |
| Describe if and how the model was validated, either internally (eg, bootstrapping, cross-validation, split random sample) or externally (eg, temporal validation, geographic validation, different setting, different type of participants): | Yes | Yes | Yes | Yes | Yes |
| Describe the performance measures of the model, e.g. (re)calibration, discrimination, (re)classification, net profit and if adjusted for optimism: | Yes | Yes | Yes | Yes | Yes |
| Describe the participants who were excluded from the analysis: | Yes | Yes | Yes | Yes | Yes |
| Describe missing data on predictors and outcomes, as well as methods used for missing data: | Yes | Yes | Yes | Yes | Yes |
| 4.1 Was there a reasonable number of participants with the result? | Yes | Yes | Yes | Yes | Yes |
| 4.2 Were continuous and categorical predictors handled correctly? | Yes | Yes | Yes | Yes | Yes |
| 4.3 Were all enrolled participants included in the analysis? | Yes | Yes | Yes | Yes | Yes |
| 4.4 Were participants with missing data treated appropriately? | Yes | Yes | Yes | Yes | Yes |
| 4.5 Was selection of predictors based on univariate analysis avoided? | Yes | Yes | Yes | Yes | Yes |
| 4.6 Were the complexities of the data (eg censoring, competing risks, sampling of controls) adequately accounted for? | Yes | Yes | Yes | Yes | Yes |
| 4.7 Were relevant model performance measures adequately evaluated? | Yes | Yes | Yes | Yes | Yes |
| 4.8 Was model overfitting and optimism factored into model performance? | Yes | Yes | Yes | Yes | Yes |
| 4.9 Do the predictors and their assigned weights in the final model correspond to the results of the multivariate analysis? | Yes | Yes | Yes | Yes | Yes |
| **Risk of bias introduced by the analysis (low/high/Unclear)** | Low | Low | Low | Low | Low |
| **Step 4: General evaluation** |  |  |  |  |  |
| **Overall assessment of risk of bias** | Low | Low | Low | Low | Low |
| **General judgment of applicability** | Low | Low | Low | Low | Low |
|  |  |  |  |  |  |
|  |  |  |  |  |  |
| **Domain 1** | **Campos et al. (2021)** | **Canga et al. (2022)** | **Buchwald et al. (2021)** | **Catalá et al. (2021)** | **Cazelles et al. (2021)** |
| **A. Risk of bias** | **12-06-22** | **12-06-22** | **12-06-22** | **12-06-22** | **12-06-22** |
| 1.1 Were appropriate data sources used, e.g. data from cohort studies, RCTs or nested case-controls? | No | Unclear | Yes | Yes | No |
| 1.2 Were all inclusions and exclusions of participants appropriate? | No | Unclear | No | Yes | No |
| Risk of bias introduced by participant selection (low/high/unclear) | High | Unclear | Low | Low | High |
| **B. Applicability** |  |  |  |  |  |
| Concern that included participants and setting do not match review question (low/high/unclear) | Low | Low | Low | Low | Low |
| **Domain 2** |  |  |  |  |  |
| **A. Risk of bias - predictors** |  |  |  |  |  |
| 2.1 Were predictors defined and evaluated in a similar way for all participants? | Yes | Yes | Yes | Yes | Yes |
| 2.2 Were predictor evaluations performed without knowing the outcome data? | Yes | Yes | Yes | Yes | Yes |
| 2.3 Are all the predictors available at the time the model is intended to be used? | Yes | Yes | Yes | Yes | Yes |
| Risk of bias introduced by the predictors or their assessment (low/high/unclear) | Low | Low | Low | Low | Low |
| **B. Applicability** |  |  |  |  |  |
| Concern that the definition, evaluation, or timing of predictors in the model do not match the review question (low/high/unclear) | Low | Low | Low | Low | Low |
| **Domain 3** |  |  |  |  |  |
| **A. Risk of bias - outcome** |  |  |  |  |  |
| Was the result properly determined? | Yes | Yes | Yes | Yes | Yes |
| Was a standard or prespecified outcome definition used? | Yes | Yes | Yes | Yes | Yes |
| Were predictors excluded from the outcome definition? | Yes | Yes | Yes | Yes | Yes |
| Was the outcome similarly defined and determined for all participants? | Yes | Yes | Yes | Yes | Yes |
| Was the outcome determined without knowing the predictor information? | Yes | Yes | Yes | Yes | Yes |
| Was the time interval between the evaluation of the predictor and the determination of the result adequate? | Yes | Yes | Yes | Yes | Yes |
| Risk of bias introduced by the result or its determination (low/high/unclear) | Low | Low | Low | Low | Low |
| **B. Applicability** |  |  |  |  |  |
| If a composite outcome was used, please describe the relative frequency/distribution of each contributing outcome: | NA | NA | NA | NA | NA |
| Concern that the outcome, its definition, timing, or determination does not match the review question (low/high/unclear) | Low | Low | Low | Low | Low |
| **Domain 4** |  |  |  |  |  |
| **A. Risk of bias** |  |  |  |  |  |
| Describe the number of participants, number of candidate predictors, outcome events, and events per candidate predictor: | Yes | Yes | Yes | Yes | Yes |
| Describe how the model was developed (for example, with respect to modeling technique (eg, survival or logistic modeling), selection of predictors, and definition of risk groups): | Yes | Yes | Yes | Yes | Yes |
| Describe if and how the model was validated, either internally (eg, bootstrapping, cross-validation, split random sample) or externally (eg, temporal validation, geographic validation, different setting, different type of participants): | Yes | Yes | Yes | Yes | Yes |
| Describe the performance measures of the model, e.g. (re)calibration, discrimination, (re)classification, net profit and if adjusted for optimism: | Yes | Yes | Yes | Yes | Yes |
| Describe the participants who were excluded from the analysis: | Yes | Yes | Yes | Yes | Yes |
| Describe missing data on predictors and outcomes, as well as methods used for missing data: | Yes | Yes | Yes | Yes | Yes |
| 4.1 Was there a reasonable number of participants with the result? | Yes | Yes | Yes | Yes | Yes |
| 4.2 Were continuous and categorical predictors handled correctly? | Yes | Yes | Yes | Yes | Yes |
| 4.3 Were all enrolled participants included in the analysis? | Yes | Yes | Yes | Yes | Yes |
| 4.4 Were participants with missing data treated appropriately? | Yes | Yes | Yes | Yes | Yes |
| 4.5 Was selection of predictors based on univariate analysis avoided? | Yes | Yes | Yes | Yes | Yes |
| 4.6 Were the complexities of the data (eg censoring, competing risks, sampling of controls) adequately accounted for? | Yes | Yes | Yes | Yes | Yes |
| 4.7 Were relevant model performance measures adequately evaluated? | Yes | Yes | Yes | Yes | Yes |
| 4.8 Was model overfitting and optimism factored into model performance? | Yes | Yes | Yes | Yes | Yes |
| 4.9 Do the predictors and their assigned weights in the final model correspond to the results of the multivariate analysis? | Yes | Yes | Yes | Yes | Yes |
| **Risk of bias introduced by the analysis (low/high/Unclear)** | Low | Low | Low | Low | Low |
| **Step 4: General evaluation** | There is not enough information related to the chosen population, so there is a risk of introducing bias in the selection | The population is not clear, related information is found although no information is detailed | - | - | Nothing is mentioned about the population or its source |
| **Overall assessment of risk of bias** | High | Unclear | Low | Low | High |
| **General judgment of applicability** | Low | Low | Low | Low | Low |
|  |  |  |  |  |  |
|  |  |  |  |  |  |
| **Domain 1** | **Chang et al. (2021)** | **Chapman et al. (2022) (1)** | **Chapman et al. (2022) (2)** | **Chen et al. (2020)** |  |
| **A. Risk of bias** | **12-06-22** | **12-06-22** | **12-06-22** | **12-06-22** |  |
| 1.1 Were appropriate data sources used, e.g. data from cohort studies, RCTs or nested case-controls? | No | Yes | Yes | No |  |
| 1.2 Were all inclusions and exclusions of participants appropriate? | No | Yes | Yes | No |  |
| Risk of bias introduced by participant selection (low/high/unclear) | High | Low | Low | High |  |
| **B. Applicability** |  |  |  |  |  |
| Concern that included participants and setting do not match review question (low/high/unclear) | Low | Low | Low | Low |  |
| **Domain 2** |  |  |  |  |  |
| **A. Risk of bias - predictors** |  |  |  |  |  |
| 2.1 Were predictors defined and evaluated in a similar way for all participants? | Yes | Yes | Yes | Yes |  |
| 2.2 Were predictor evaluations performed without knowing the outcome data? | Yes | Yes | Yes | Yes |  |
| 2.3 Are all the predictors available at the time the model is intended to be used? | Yes | Yes | Yes | Yes |  |
| Risk of bias introduced by the predictors or their assessment (low/high/unclear) | Low | Low | Low | Low |  |
| **B. Applicability** |  |  |  |  |  |
| Concern that the definition, evaluation, or timing of predictors in the model do not match the review question (low/high/unclear) | Low | Low | Low | Low |  |
| **Domain 3** |  |  |  |  |  |
| **A. Risk of bias - outcome** |  |  |  |  |  |
| Was the result properly determined? | Yes | Yes | Yes | Yes |  |
| Was a standard or prespecified outcome definition used? | Yes | Yes | Yes | Yes |  |
| Were predictors excluded from the outcome definition? | Yes | Yes | Yes | Yes |  |
| Was the outcome similarly defined and determined for all participants? | Yes | Yes | Yes | Yes |  |
| Was the outcome determined without knowing the predictor information? | Yes | Yes | Yes | Yes |  |
| Was the time interval between the evaluation of the predictor and the determination of the result adequate? | Yes | Yes | Yes | Yes |  |
| Risk of bias introduced by the result or its determination (low/high/unclear) | Low | Low | Low | Low |  |
| **B. Applicability** |  |  |  |  |  |
| If a composite outcome was used, please describe the relative frequency/distribution of each contributing outcome: | NA | NA | NA | NA |  |
| Concern that the outcome, its definition, timing, or determination does not match the review question (low/high/unclear) | Low | Low | Low | Low |  |
| **Domain 4** |  |  |  |  |  |
| **A. Risk of bias** |  |  |  |  |  |
| Describe the number of participants, number of candidate predictors, outcome events, and events per candidate predictor: | Yes | Yes | Yes | Yes |  |
| Describe how the model was developed (for example, with respect to modeling technique (eg, survival or logistic modeling), selection of predictors, and definition of risk groups): | Yes | Yes | Yes | Yes |  |
| Describe if and how the model was validated, either internally (eg, bootstrapping, cross-validation, split random sample) or externally (eg, temporal validation, geographic validation, different setting, different type of participants): | Yes | Yes | Yes | Yes |  |
| Describe the performance measures of the model, e.g. (re)calibration, discrimination, (re)classification, net profit and if adjusted for optimism: | Yes | Yes | Yes | Yes |  |
| Describe the participants who were excluded from the analysis: | Yes | Yes | Yes | Yes |  |
| Describe missing data on predictors and outcomes, as well as methods used for missing data: | Yes | Yes | Yes | Yes |  |
| 4.1 Was there a reasonable number of participants with the result? | Yes | Yes | Yes | Yes |  |
| 4.2 Were continuous and categorical predictors handled correctly? | Yes | Yes | Yes | Yes |  |
| 4.3 Were all enrolled participants included in the analysis? | Yes | Yes | Yes | Yes |  |
| 4.4 Were participants with missing data treated appropriately? | Yes | Yes | Yes | Yes |  |
| 4.5 Was selection of predictors based on univariate analysis avoided? | Yes | Yes | Yes | Yes |  |
| 4.6 Were the complexities of the data (eg censoring, competing risks, sampling of controls) adequately accounted for? | Yes | Yes | Yes | Yes |  |
| 4.7 Were relevant model performance measures adequately evaluated? | Yes | Yes | Yes | Yes |  |
| 4.8 Was model overfitting and optimism factored into model performance? | Yes | Yes | Yes | Yes |  |
| 4.9 Do the predictors and their assigned weights in the final model correspond to the results of the multivariate analysis? | Yes | Yes | Yes | Yes |  |
| **Risk of bias introduced by the analysis (low/high/Unclear)** | Low | Low | Low | Low |  |
| **Step 4: General evaluation** | There is not enough information related to the chosen population, so there is a risk of introducing bias in the selection | - | - | Nothing is mentioned about the population or its source |  |
| **Overall assessment of risk of bias** | High | Low | Low | High |  |
| **General judgment of applicability** | Low | Low | Low | Low |  |
|  |  |  |  |  |  |
|  |  |  |  |  |  |
| **Domain 1** | **Chen et al. (2021) (1)** | **Chen et al. (2021) (2)** | **Chen et al. (2021) (3)** | **Chen et al. (2021) (4)** | **Chen et al. (2022)** |
| **A. Risk of bias** | **12-06-22** | **12-06-22** | **12-06-22** | **12-06-22** | **12-06-22** |
| 1.1 Were appropriate data sources used, e.g. data from cohort studies, RCTs or nested case-controls? | No | No | No | Yes | Yes |
| 1.2 Were all inclusions and exclusions of participants appropriate? | No | No | No | Yes | Yes |
| Risk of bias introduced by participant selection (low/high/unclear) | High | High | High | Low | Low |
| **B. Applicability** |  |  |  |  |  |
| Concern that included participants and setting do not match review question (low/high/unclear) | Low | Low | Low | Low | Low |
| **Domain 2** |  |  |  |  |  |
| **A. Risk of bias - predictors** |  |  |  |  |  |
| 2.1 Were predictors defined and evaluated in a similar way for all participants? | Yes | Yes | Yes | Yes | Yes |
| 2.2 Were predictor evaluations performed without knowing the outcome data? | Yes | Yes | Yes | Yes | Yes |
| 2.3 Are all the predictors available at the time the model is intended to be used? | Yes | Yes | Yes | Yes | Yes |
| Risk of bias introduced by the predictors or their assessment (low/high/unclear) | Low | Low | Low | Low | Low |
| **B. Applicability** |  |  |  |  |  |
| Concern that the definition, evaluation, or timing of predictors in the model do not match the review question (low/high/unclear) | Low | Low | Low | Low | Low |
| **Domain 3** |  |  |  |  |  |
| **A. Risk of bias - outcome** |  |  |  |  |  |
| Was the result properly determined? | Yes | Yes | Yes | Yes | Yes |
| Was a standard or prespecified outcome definition used? | Yes | Yes | Yes | Yes | Yes |
| Were predictors excluded from the outcome definition? | Yes | Yes | Yes | Yes | Yes |
| Was the outcome similarly defined and determined for all participants? | Yes | Yes | Yes | Yes | Yes |
| Was the outcome determined without knowing the predictor information? | Yes | Yes | Yes | Yes | Yes |
| Was the time interval between the evaluation of the predictor and the determination of the result adequate? | Yes | Yes | Yes | Yes | Yes |
| Risk of bias introduced by the result or its determination (low/high/unclear) | Low | Low | Low | Low | Low |
| **B. Applicability** |  |  |  |  |  |
| If a composite outcome was used, please describe the relative frequency/distribution of each contributing outcome: | NA | NA | NA | NA | NA |
| Concern that the outcome, its definition, timing, or determination does not match the review question (low/high/unclear) | Low | Low | Low | Low | Low |
| **Domain 4** |  |  |  |  |  |
| **A. Risk of bias** |  |  |  |  |  |
| Describe the number of participants, number of candidate predictors, outcome events, and events per candidate predictor: | Yes | Yes | Yes | Yes | Yes |
| Describe how the model was developed (for example, with respect to modeling technique (eg, survival or logistic modeling), selection of predictors, and definition of risk groups): | Yes | Yes | Yes | Yes | Yes |
| Describe if and how the model was validated, either internally (eg, bootstrapping, cross-validation, split random sample) or externally (eg, temporal validation, geographic validation, different setting, different type of participants): | Yes | Yes | Yes | Yes | Yes |
| Describe the performance measures of the model, e.g. (re)calibration, discrimination, (re)classification, net profit and if adjusted for optimism: | Yes | Yes | Yes | Yes | Yes |
| Describe the participants who were excluded from the analysis: | Yes | Yes | Yes | Yes | Yes |
| Describe missing data on predictors and outcomes, as well as methods used for missing data: | Yes | Yes | Yes | Yes | Yes |
| 4.1 Was there a reasonable number of participants with the result? | Yes | Yes | Yes | Yes | Yes |
| 4.2 Were continuous and categorical predictors handled correctly? | Yes | Yes | Yes | Yes | Yes |
| 4.3 Were all enrolled participants included in the analysis? | Yes | Yes | Yes | Yes | Yes |
| 4.4 Were participants with missing data treated appropriately? | Yes | Yes | Yes | Yes | Yes |
| 4.5 Was selection of predictors based on univariate analysis avoided? | Yes | Yes | Yes | Yes | Yes |
| 4.6 Were the complexities of the data (eg censoring, competing risks, sampling of controls) adequately accounted for? | Yes | Yes | Yes | Yes | Yes |
| 4.7 Were relevant model performance measures adequately evaluated? | Yes | Yes | Yes | Yes | Yes |
| 4.8 Was model overfitting and optimism factored into model performance? | Yes | Yes | Yes | Yes | Yes |
| 4.9 Do the predictors and their assigned weights in the final model correspond to the results of the multivariate analysis? | Yes | Yes | Yes | Yes | Yes |
| **Risk of bias introduced by the analysis (low/high/Unclear)** | Low | Low | Low | Low | Low |
| **Step 4: General evaluation** | There is not enough information related to the chosen population, so there is a risk of introducing bias in the selection | Nothing is mentioned about the population or its source | Nothing is mentioned about the population or its source | **-** | **-** |
| **Overall assessment of risk of bias** | High | High | High | Low | Low |
| **General judgment of applicability** | Low | Low | Low | Low | Low |
|  |  |  |  |  |  |
|  |  |  |  |  |  |
| **Domain 1** | **Chen et al. (2021) (5)** | **Childs et al. (2021)** | **Chinazzi et al. (2020)** | **Chinchilla et al. (2021)** | **Choi et al. (2021) (1)** |
| **A. Risk of bias** | **12-06-22** | **12-06-22** | **12-06-22** | **12-06-22** | **12-06-22** |
| 1.1 Were appropriate data sources used, e.g. data from cohort studies, RCTs or nested case-controls? | Yes | Yes | Yes | Yes | Yes |
| 1.2 Were all inclusions and exclusions of participants appropriate? | Yes | Unclear | No | Unclear | Yes |
| Risk of bias introduced by participant selection (low/high/unclear) | Low | Unclear | Unclear | Low | Low |
| **B. Applicability** |  |  |  |  |  |
| Concern that included participants and setting do not match review question (low/high/unclear) | Low | Low | Low | Low | Low |
| **Domain 2** |  |  |  |  |  |
| **A. Risk of bias - predictors** |  |  |  |  |  |
| 2.1 Were predictors defined and evaluated in a similar way for all participants? | Yes | Yes | Yes | Yes | Yes |
| 2.2 Were predictor evaluations performed without knowing the outcome data? | Yes | Yes | Yes | Yes | Yes |
| 2.3 Are all the predictors available at the time the model is intended to be used? | Yes | Yes | Yes | Yes | Yes |
| Risk of bias introduced by the predictors or their assessment (low/high/unclear) | Low | Low | Low | Low | Low |
| **B. Applicability** |  |  |  |  |  |
| Concern that the definition, evaluation, or timing of predictors in the model do not match the review question (low/high/unclear) | Low | Low | Low | Low | Low |
| **Domain 3** |  |  |  |  |  |
| **A. Risk of bias - outcome** |  |  |  |  |  |
| Was the result properly determined? | Yes | Yes | Yes | Yes | Yes |
| Was a standard or prespecified outcome definition used? | Yes | Unclear | Unclear | Yes | Yes |
| Were predictors excluded from the outcome definition? | Yes | Yes | Yes | Yes | Yes |
| Was the outcome similarly defined and determined for all participants? | Yes | Yes | Yes | Yes | Yes |
| Was the outcome determined without knowing the predictor information? | Yes | No | Yes | Yes | Yes |
| Was the time interval between the evaluation of the predictor and the determination of the result adequate? | Yes | Yes | Yes | Yes | Yes |
| Risk of bias introduced by the result or its determination (low/high/unclear) | Low | Low | Low | Low | Low |
| **B. Applicability** |  |  |  |  |  |
| If a composite outcome was used, please describe the relative frequency/distribution of each contributing outcome: | NA | NA | NA | NA | NA |
| Concern that the outcome, its definition, timing, or determination does not match the review question (low/high/unclear) | Low | Low | Low | Low | Low |
| **Domain 4** |  |  |  |  |  |
| **A. Risk of bias** |  |  |  |  |  |
| Describe the number of participants, number of candidate predictors, outcome events, and events per candidate predictor: | Yes | No | Yes | Yes | Yes |
| Describe how the model was developed (for example, with respect to modeling technique (eg, survival or logistic modeling), selection of predictors, and definition of risk groups): | Yes | Yes | Yes | Yes | Yes |
| Describe if and how the model was validated, either internally (eg, bootstrapping, cross-validation, split random sample) or externally (eg, temporal validation, geographic validation, different setting, different type of participants): | Yes | Unclear | Yes | Yes | Yes |
| Describe the performance measures of the model, e.g. (re)calibration, discrimination, (re)classification, net profit and if adjusted for optimism: | Yes | Yes | Yes | Yes | Yes |
| Describe the participants who were excluded from the analysis: | Yes | No | Yes | Yes | Yes |
| Describe missing data on predictors and outcomes, as well as methods used for missing data: | Yes | No | Yes | Yes | Yes |
| 4.1 Was there a reasonable number of participants with the result? | Yes | Yes | Yes | Yes | Yes |
| 4.2 Were continuous and categorical predictors handled correctly? | Yes | Yes | Yes | Yes | Yes |
| 4.3 Were all enrolled participants included in the analysis? | Yes | Yes | Yes | Yes | Yes |
| 4.4 Were participants with missing data treated appropriately? | Yes | Unclear | Unclear | Yes | Yes |
| 4.5 Was selection of predictors based on univariate analysis avoided? | Yes | Yes | Yes | Yes | Yes |
| 4.6 Were the complexities of the data (eg censoring, competing risks, sampling of controls) adequately accounted for? | Yes | Yes | Yes | Yes | Yes |
| 4.7 Were relevant model performance measures adequately evaluated? | Yes | Yes | Yes | Yes | Yes |
| 4.8 Was model overfitting and optimism factored into model performance? | Yes | Yes | Yes | Yes | Yes |
| 4.9 Do the predictors and their assigned weights in the final model correspond to the results of the multivariate analysis? | Yes | Yes | Yes | Yes | Yes |
| **Risk of bias introduced by the analysis (low/high/Unclear)** | Low | High | Low | Low | Low |
| **Step 4: General evaluation** | **-** | This study does not have enough information regarding the methodology | There is not much detail of the population used | **-** | **-** |
| **Overall assessment of risk of bias** | Low | High | Unclear | Low | Low |
| **General judgment of applicability** | Low | High | Low | Low | Low |
|  |  |  |  |  |  |
|  |  |  |  |  |  |
| **Domain 1** | **Choi et al. (2021) (2)** | **Chopra et al. (2021)** | **Chu et al. (2021)** | **Chun et al. (2021)** | **Chun et al. (2022)** |
| **A. Risk of bias** | **12-06-22** | **12-06-22** | **12-06-22** | **12-06-22** | **12-06-22** |
| 1.1 Were appropriate data sources used, e.g. data from cohort studies, RCTs or nested case-controls? | Yes | No | No | Yes | Yes |
| 1.2 Were all inclusions and exclusions of participants appropriate? | Yes | No | No | Unclear | No |
| Risk of bias introduced by participant selection (low/high/unclear) | Low | High | High | Low | Low |
| **B. Applicability** |  |  |  |  |  |
| Concern that included participants and setting do not match review question (low/high/unclear) | Low | Low | Low | Low | Low |
| **Domain 2** |  |  |  |  |  |
| **A. Risk of bias - predictors** |  |  |  |  |  |
| 2.1 Were predictors defined and evaluated in a similar way for all participants? | Yes | Yes | Yes | Yes | Yes |
| 2.2 Were predictor evaluations performed without knowing the outcome data? | Yes | Yes | Yes | Yes | Yes |
| 2.3 Are all the predictors available at the time the model is intended to be used? | Yes | Yes | Yes | Yes | Yes |
| Risk of bias introduced by the predictors or their assessment (low/high/unclear) | Low | Low | Low | Low | Low |
| **B. Applicability** |  |  |  |  |  |
| Concern that the definition, evaluation, or timing of predictors in the model do not match the review question (low/high/unclear) | Low | Low | Low | Low | Low |
| **Domain 3** |  |  |  |  |  |
| **A. Risk of bias - outcome** |  |  |  |  |  |
| Was the result properly determined? | Yes | Yes | Yes | Yes | Yes |
| Was a standard or prespecified outcome definition used? | Yes | Unclear | Yes | Yes | Yes |
| Were predictors excluded from the outcome definition? | Yes | Yes | Yes | Yes | Yes |
| Was the outcome similarly defined and determined for all participants? | Yes | Yes | Yes | Yes | Yes |
| Was the outcome determined without knowing the predictor information? | Yes | No | Yes | Yes | Yes |
| Was the time interval between the evaluation of the predictor and the determination of the result adequate? | Yes | Yes | Yes | Yes | Yes |
| Risk of bias introduced by the result or its determination (low/high/unclear) | Low | Low | Low | Low | Low |
| **B. Applicability** |  |  |  |  |  |
| If a composite outcome was used, please describe the relative frequency/distribution of each contributing outcome: | NA | NA | NA | NA | NA |
| Concern that the outcome, its definition, timing, or determination does not match the review question (low/high/unclear) | Low | Low | Low | Low | Low |
| **Domain 4** |  |  |  |  |  |
| **A. Risk of bias** |  |  |  |  |  |
| Describe the number of participants, number of candidate predictors, outcome events, and events per candidate predictor: | Yes | No | Yes | Yes | Yes |
| Describe how the model was developed (for example, with respect to modeling technique (eg, survival or logistic modeling), selection of predictors, and definition of risk groups): | Yes | Yes | Yes | Yes | Yes |
| Describe if and how the model was validated, either internally (eg, bootstrapping, cross-validation, split random sample) or externally (eg, temporal validation, geographic validation, different setting, different type of participants): | Yes | Unclear | Yes | Yes | Yes |
| Describe the performance measures of the model, e.g. (re)calibration, discrimination, (re)classification, net profit and if adjusted for optimism: | Yes | Yes | Yes | Yes | Yes |
| Describe the participants who were excluded from the analysis: | Yes | No | Yes | Yes | Yes |
| Describe missing data on predictors and outcomes, as well as methods used for missing data: | Yes | No | Yes | Yes | Yes |
| 4.1 Was there a reasonable number of participants with the result? | Yes | Yes | Yes | Yes | Yes |
| 4.2 Were continuous and categorical predictors handled correctly? | Yes | Yes | Yes | Yes | Yes |
| 4.3 Were all enrolled participants included in the analysis? | Yes | Yes | Yes | Yes | Yes |
| 4.4 Were participants with missing data treated appropriately? | Yes | Unclear | Yes | Yes | Yes |
| 4.5 Was selection of predictors based on univariate analysis avoided? | Yes | Yes | Yes | Yes | Yes |
| 4.6 Were the complexities of the data (eg censoring, competing risks, sampling of controls) adequately accounted for? | Yes | Yes | Yes | Yes | Yes |
| 4.7 Were relevant model performance measures adequately evaluated? | Yes | Yes | Yes | Yes | Yes |
| 4.8 Was model overfitting and optimism factored into model performance? | Yes | Yes | Yes | Yes | Yes |
| 4.9 Do the predictors and their assigned weights in the final model correspond to the results of the multivariate analysis? | Yes | Yes | Yes | Yes | Yes |
| **Risk of bias introduced by the analysis (low/high/Unclear)** | Low | High | Low | Low | Low |
| **Step 4: General evaluation** | **-** | Information about population is not detailed and there is a lack of information regarding the methodology | Population information is not detailed | **-** | **-** |
| **Overall assessment of risk of bias** | Low | High | High | Low | Low |
| **General judgment of applicability** | Low | High | Low | Low | Low |
|  |  |  |  |  |  |
|  |  |  |  |  |  |
| **Domain 1** | **Cipriano et al. (2021)** | **Cohen et al. (2021)** | **Colomer et al. (2021)** | **Colosi et al. (2022) (1)** |  |
| **A. Risk of bias** | **12-06-22** | **12-06-22** | **12-06-22** | **12-06-22** |  |
| 1.1 Were appropriate data sources used, e.g. data from cohort studies, RCTs or nested case-controls? | Yes | Yes | Yes | Yes |  |
| 1.2 Were all inclusions and exclusions of participants appropriate? | No | Yes | Yes | Yes |  |
| Risk of bias introduced by participant selection (low/high/unclear) | Low | Low | Low | Low |  |
| **B. Applicability** |  |  |  |  |  |
| Concern that included participants and setting do not match review question (low/high/unclear) | Low | Low | Low | Low |  |
| **Domain 2** |  |  |  |  |  |
| **A. Risk of bias - predictors** |  |  |  |  |  |
| 2.1 Were predictors defined and evaluated in a similar way for all participants? | Yes | Yes | Yes | Yes |  |
| 2.2 Were predictor evaluations performed without knowing the outcome data? | Yes | Yes | Yes | Yes |  |
| 2.3 Are all the predictors available at the time the model is intended to be used? | Yes | Yes | Yes | Yes |  |
| Risk of bias introduced by the predictors or their assessment (low/high/unclear) | Low | Low | Low | Low |  |
| **B. Applicability** |  |  |  |  |  |
| Concern that the definition, evaluation, or timing of predictors in the model do not match the review question (low/high/unclear) | Low | Low | Low | Low |  |
| **Domain 3** |  |  |  |  |  |
| **A. Risk of bias - outcome** |  |  |  |  |  |
| Was the result properly determined? | Yes | Yes | Yes | Yes |  |
| Was a standard or prespecified outcome definition used? | Yes | Yes | Yes | Yes |  |
| Were predictors excluded from the outcome definition? | Yes | Yes | Yes | Yes |  |
| Was the outcome similarly defined and determined for all participants? | Yes | Yes | Yes | Yes |  |
| Was the outcome determined without knowing the predictor information? | Yes | Yes | Yes | Yes |  |
| Was the time interval between the evaluation of the predictor and the determination of the result adequate? | Yes | Yes | Yes | Yes |  |
| Risk of bias introduced by the result or its determination (low/high/unclear) | Low | Low | Low | Low |  |
| **B. Applicability** |  |  |  |  |  |
| If a composite outcome was used, please describe the relative frequency/distribution of each contributing outcome: | Yes | Yes | Yes | Yes |  |
| Concern that the outcome, its definition, timing, or determination does not match the review question (low/high/unclear) | Low | Low | Low | Low |  |
| **Domain 4** |  |  |  |  |  |
| **A. Risk of bias** |  |  |  |  |  |
| Describe the number of participants, number of candidate predictors, outcome events, and events per candidate predictor: | Yes | Yes | Yes | Yes |  |
| Describe how the model was developed (for example, with respect to modeling technique (eg, survival or logistic modeling), selection of predictors, and definition of risk groups): | Yes | Yes | Yes | Yes |  |
| Describe if and how the model was validated, either internally (eg, bootstrapping, cross-validation, split random sample) or externally (eg, temporal validation, geographic validation, different setting, different type of participants): | Yes | Yes | Yes | Yes |  |
| Describe the performance measures of the model, e.g. (re)calibration, discrimination, (re)classification, net profit and if adjusted for optimism: | Yes | Yes | Yes | Yes |  |
| Describe the participants who were excluded from the analysis: | Yes | Yes | Yes | Yes |  |
| Describe missing data on predictors and outcomes, as well as methods used for missing data: | Yes | Yes | Yes | Yes |  |
| 4.1 Was there a reasonable number of participants with the result? | Yes | Yes | Yes | Yes |  |
| 4.2 Were continuous and categorical predictors handled correctly? | Yes | Yes | Yes | Yes |  |
| 4.3 Were all enrolled participants included in the analysis? | Yes | Yes | Yes | Yes |  |
| 4.4 Were participants with missing data treated appropriately? | Yes | Yes | Yes | Yes |  |
| 4.5 Was selection of predictors based on univariate analysis avoided? | Yes | Yes | Yes | Yes |  |
| 4.6 Were the complexities of the data (eg censoring, competing risks, sampling of controls) adequately accounted for? | Yes | Yes | Yes | Yes |  |
| 4.7 Were relevant model performance measures adequately evaluated? | Yes | Yes | Yes | Yes |  |
| 4.8 Was model overfitting and optimism factored into model performance? | Yes | Yes | Yes | Yes |  |
| 4.9 Do the predictors and their assigned weights in the final model correspond to the results of the multivariate analysis? | Yes | Yes | Yes | Yes |  |
| **Risk of bias introduced by the analysis (low/high/Unclear)** | Low | Low | Low | Low |  |
| **Step 4: General evaluation** | **-** | **-** | **-** | **-** |  |
| **Overall assessment of risk of bias** | Low | Low | Low | Low |  |
| **General judgment of applicability** | Low | Low | Low | Low |  |
|  |  |  |  |  |  |
|  |  |  |  |  |  |
| **Domain 1** | **Colosi et al. (2022) (2)** | **Contreras et al. (2020)** | **Contreras et al. (2021)** | **Contreras et al. (2022)** |  |
| **A. Risk of bias** | **12-06-22** | **12-06-22** | **13-06-22** | **13-06-22** |  |
| 1.1 Were appropriate data sources used, e.g. data from cohort studies, RCTs or nested case-controls? | No | Yes | Yes | Yes |  |
| 1.2 Were all inclusions and exclusions of participants appropriate? | No | Unclear | Unclear | Unclear |  |
| Risk of bias introduced by participant selection (low/high/unclear) | High | Low | Low | Low |  |
| **B. Applicability** |  |  |  |  |  |
| Concern that included participants and setting do not match review question (low/high/unclear) | Low | Low | Low | Low |  |
| **Domain 2** |  |  |  |  |  |
| **A. Risk of bias - predictors** |  |  |  |  |  |
| 2.1 Were predictors defined and evaluated in a similar way for all participants? | No | Yes | Yes | Yes |  |
| 2.2 Were predictor evaluations performed without knowing the outcome data? | Unclear | Yes | Yes | Yes |  |
| 2.3 Are all the predictors available at the time the model is intended to be used? | Unclear | Yes | Yes | Yes |  |
| Risk of bias introduced by the predictors or their assessment (low/high/unclear) | High | Low | Low | Low |  |
| **B. Applicability** |  |  |  |  |  |
| Concern that the definition, evaluation, or timing of predictors in the model do not match the review question (low/high/unclear) | Unclear | Low | Low | Low |  |
| **Domain 3** |  |  |  |  |  |
| **A. Risk of bias - outcome** |  |  |  |  |  |
| Was the result properly determined? | Yes | Yes | Yes | Yes |  |
| Was a standard or prespecified outcome definition used? | No | Yes | Yes | Yes |  |
| Were predictors excluded from the outcome definition? | Unclear | Yes | Yes | Yes |  |
| Was the outcome similarly defined and determined for all participants? | Unclear | Yes | Yes | Yes |  |
| Was the outcome determined without knowing the predictor information? | Unclear | Yes | Yes | Yes |  |
| Was the time interval between the evaluation of the predictor and the determination of the result adequate? | Yes | Yes | Yes | Yes |  |
| Risk of bias introduced by the result or its determination (low/high/unclear) | High | Low | Low | Low |  |
| **B. Applicability** |  |  |  |  |  |
| If a composite outcome was used, please describe the relative frequency/distribution of each contributing outcome: | Unclear | NA | NA | NA |  |
| Concern that the outcome, its definition, timing, or determination does not match the review question (low/high/unclear) | Unclear | Low | Low | Low |  |
| **Domain 4** |  |  |  |  |  |
| **A. Risk of bias** |  |  |  |  |  |
| Describe the number of participants, number of candidate predictors, outcome events, and events per candidate predictor: | No | Yes | Yes | Yes |  |
| Describe how the model was developed (for example, with respect to modeling technique (eg, survival or logistic modeling), selection of predictors, and definition of risk groups): | Unclear | Yes | Yes | Yes |  |
| Describe if and how the model was validated, either internally (eg, bootstrapping, cross-validation, split random sample) or externally (eg, temporal validation, geographic validation, different setting, different type of participants): | Unclear | Yes | Yes | Yes |  |
| Describe the performance measures of the model, e.g. (re)calibration, discrimination, (re)classification, net profit and if adjusted for optimism: | Unclear | Yes | Yes | Yes |  |
| Describe the participants who were excluded from the analysis: | No | Yes | Yes | Yes |  |
| Describe missing data on predictors and outcomes, as well as methods used for missing data: | No | Yes | Yes | Yes |  |
| 4.1 Was there a reasonable number of participants with the result? | Yes | Yes | Yes | Yes |  |
| 4.2 Were continuous and categorical predictors handled correctly? | Yes | Yes | Yes | Yes |  |
| 4.3 Were all enrolled participants included in the analysis? | Yes | Yes | Yes | Yes |  |
| 4.4 Were participants with missing data treated appropriately? | Yes | Yes | Yes | Yes |  |
| 4.5 Was selection of predictors based on univariate analysis avoided? | Yes | Yes | Yes | Yes |  |
| 4.6 Were the complexities of the data (eg censoring, competing risks, sampling of controls) adequately accounted for? | Yes | Yes | Yes | Yes |  |
| 4.7 Were relevant model performance measures adequately evaluated? | Yes | Yes | Yes | Yes |  |
| 4.8 Was model overfitting and optimism factored into model performance? | Yes | Yes | Yes | Yes |  |
| 4.9 Do the predictors and their assigned weights in the final model correspond to the results of the multivariate analysis? | No | Yes | Yes | Yes |  |
| **Risk of bias introduced by the analysis (low/high/Unclear)** | High | Low | Low | Low |  |
| **Step 4: General evaluation** | This study does not have many details required to define the model | - | **-** | **-** |  |
| **Overall assessment of risk of bias** | High | Low | Low | Low |  |
| **General judgment of applicability** | High | Low | Low | Low |  |
|  |  |  |  |  |  |
|  |  |  |  |  |  |
| **Domain 1** | **Cook et al. (2021)** | **Coudeville et al. (2021)** | **Cruz et al. (2021)** | **Cuesta-Lazaro et al. (2021)** |  |
| **A. Risk of bias** | **13-06-22** | **13-06-22** | **13-06-22** | **14-06-22** |  |
| 1.1 Were appropriate data sources used, e.g. data from cohort studies, RCTs or nested case-controls? | Yes | Yes | Yes | Yes |  |
| 1.2 Were all inclusions and exclusions of participants appropriate? | Yes | Yes | Yes | Yes |  |
| Risk of bias introduced by participant selection (low/high/unclear) | Low | Low | Low | Low |  |
| **B. Applicability** |  |  |  |  |  |
| Concern that included participants and setting do not match review question (low/high/unclear) | Low | Low | Low | Low |  |
| **Domain 2** |  |  |  |  |  |
| **A. Risk of bias - predictors** |  |  |  |  |  |
| 2.1 Were predictors defined and evaluated in a similar way for all participants? | Yes | Yes | Yes | Yes |  |
| 2.2 Were predictor evaluations performed without knowing the outcome data? | Yes | Yes | Yes | Yes |  |
| 2.3 Are all the predictors available at the time the model is intended to be used? | Yes | Yes | Yes | Yes |  |
| Risk of bias introduced by the predictors or their assessment (low/high/unclear) | Low | Low | Low | Low |  |
| **B. Applicability** |  |  |  |  |  |
| Concern that the definition, evaluation, or timing of predictors in the model do not match the review question (low/high/unclear) | Low | Low | Low | Low |  |
| **Domain 3** |  |  |  |  |  |
| **A. Risk of bias - outcome** |  |  |  |  |  |
| Was the result properly determined? | Yes | Yes | Yes | Yes |  |
| Was a standard or prespecified outcome definition used? | Yes | Yes | Yes | Yes |  |
| Were predictors excluded from the outcome definition? | Yes | Yes | Yes | Yes |  |
| Was the outcome similarly defined and determined for all participants? | Yes | Yes | Yes | Yes |  |
| Was the outcome determined without knowing the predictor information? | Yes | Yes | Yes | Yes |  |
| Was the time interval between the evaluation of the predictor and the determination of the result adequate? | Yes | Yes | Yes | Yes |  |
| Risk of bias introduced by the result or its determination (low/high/unclear) | Low | Low | Low | Low |  |
| **B. Applicability** |  |  |  |  |  |
| If a composite outcome was used, please describe the relative frequency/distribution of each contributing outcome: | NA | NA | NA | NA |  |
| Concern that the outcome, its definition, timing, or determination does not match the review question (low/high/unclear) | Low | Low | Low | Low |  |
| **Domain 4** |  |  |  |  |  |
| **A. Risk of bias** |  |  |  |  |  |
| Describe the number of participants, number of candidate predictors, outcome events, and events per candidate predictor: | Yes | Yes | Yes | Yes |  |
| Describe how the model was developed (for example, with respect to modeling technique (eg, survival or logistic modeling), selection of predictors, and definition of risk groups): | Yes | Yes | Yes | Yes |  |
| Describe if and how the model was validated, either internally (eg, bootstrapping, cross-validation, split random sample) or externally (eg, temporal validation, geographic validation, different setting, different type of participants): | Yes | Yes | Yes | Yes |  |
| Describe the performance measures of the model, e.g. (re)calibration, discrimination, (re)classification, net profit and if adjusted for optimism: | Yes | Yes | Yes | Yes |  |
| Describe the participants who were excluded from the analysis: | Yes | Yes | Yes | Yes |  |
| Describe missing data on predictors and outcomes, as well as methods used for missing data: | Yes | Yes | Yes | Yes |  |
| 4.1 Was there a reasonable number of participants with the result? | Yes | Yes | Yes | Yes |  |
| 4.2 Were continuous and categorical predictors handled correctly? | Yes | Yes | Yes | Yes |  |
| 4.3 Were all enrolled participants included in the analysis? | Yes | Yes | Yes | Yes |  |
| 4.4 Were participants with missing data treated appropriately? | Yes | Yes | Yes | Yes |  |
| 4.5 Was selection of predictors based on univariate analysis avoided? | Yes | Yes | Yes | Yes |  |
| 4.6 Were the complexities of the data (eg censoring, competing risks, sampling of controls) adequately accounted for? | Yes | Yes | Yes | Yes |  |
| 4.7 Were relevant model performance measures adequately evaluated? | Yes | Yes | Yes | Yes |  |
| 4.8 Was model overfitting and optimism factored into model performance? | Yes | Yes | Yes | Yes |  |
| 4.9 Do the predictors and their assigned weights in the final model correspond to the results of the multivariate analysis? | Yes | Yes | Yes | Yes |  |
| **Risk of bias introduced by the analysis (low/high/Unclear)** | Low | Low | Low | Low |  |
| **Step 4: General evaluation** |  |  |  |  |  |
| **Overall assessment of risk of bias** | Low | Low | Low | Low |  |
| **General judgment of applicability** | Low | Low | Low | Low |  |
|  |  |  |  |  |  |
|  |  |  |  |  |  |
| **Domain 1** | **Dagpunar et al. (2021)** | **Darapaneni et al. (2021)** | **David et al. (2022)** | **Davies et al. (2021)** | **de Cellès et al. (2021)** |
| **A. Risk of bias** | **14-06-22** | **15-06-22** | **15-06-22** | **15-06-22** | **17-06-22** |
| 1.1 Were appropriate data sources used, e.g. data from cohort studies, RCTs or nested case-controls? | Unclear | Yes | Yes | Yes | Yes |
| 1.2 Were all inclusions and exclusions of participants appropriate? | No | Yes | Yes | Yes | Yes |
| Risk of bias introduced by participant selection (low/high/unclear) | High | Low | Low | Low | Low |
| **B. Applicability** |  |  |  |  |  |
| Concern that included participants and setting do not match review question (low/high/unclear) | Low | Low | Low | Low | Low |
| **Domain 2** |  |  |  |  |  |
| **A. Risk of bias - predictors** |  |  |  |  |  |
| 2.1 Were predictors defined and evaluated in a similar way for all participants? | Yes | Yes | Yes | Yes | Yes |
| 2.2 Were predictor evaluations performed without knowing the outcome data? | Yes | Yes | Yes | Yes | Yes |
| 2.3 Are all the predictors available at the time the model is intended to be used? | Yes | Yes | Yes | Yes | Yes |
| Risk of bias introduced by the predictors or their assessment (low/high/unclear) | Low | Low | Low | Low | Low |
| **B. Applicability** |  |  |  |  |  |
| Concern that the definition, evaluation, or timing of predictors in the model do not match the review question (low/high/unclear) | Low | Low | Low | Low | Low |
| **Domain 3** |  |  |  |  |  |
| **A. Risk of bias - outcome** |  |  |  |  |  |
| Was the result properly determined? | Yes | Yes | Yes | Yes | Yes |
| Was a standard or prespecified outcome definition used? | Yes | Yes | Yes | Yes | Yes |
| Were predictors excluded from the outcome definition? | Yes | Yes | Yes | Yes | Yes |
| Was the outcome similarly defined and determined for all participants? | Yes | Yes | Yes | Yes | Yes |
| Was the outcome determined without knowing the predictor information? | Yes | Yes | Yes | Yes | Yes |
| Was the time interval between the evaluation of the predictor and the determination of the result adequate? | Yes | Yes | Yes | Yes | Yes |
| Risk of bias introduced by the result or its determination (low/high/unclear) | Low | Low | Low | Low | Low |
| **B. Applicability** |  |  |  |  |  |
| If a composite outcome was used, please describe the relative frequency/distribution of each contributing outcome: | NA | NA | NA | NA | NA |
| Concern that the outcome, its definition, timing, or determination does not match the review question (low/high/unclear) | Low | Low | Low | Low | Low |
| **Domain 4** |  |  |  |  |  |
| **A. Risk of bias** |  |  |  |  |  |
| Describe the number of participants, number of candidate predictors, outcome events, and events per candidate predictor: | Yes | Yes | Yes | Yes | Yes |
| Describe how the model was developed (for example, with respect to modeling technique (eg, survival or logistic modeling), selection of predictors, and definition of risk groups): | Yes | Yes | Yes | Yes | Yes |
| Describe if and how the model was validated, either internally (eg, bootstrapping, cross-validation, split random sample) or externally (eg, temporal validation, geographic validation, different setting, different type of participants): | Yes | Yes | Yes | Yes | Yes |
| Describe the performance measures of the model, e.g. (re)calibration, discrimination, (re)classification, net profit and if adjusted for optimism: | Yes | Yes | Yes | Yes | Yes |
| Describe the participants who were excluded from the analysis: | Yes | Yes | Yes | Yes | Yes |
| Describe missing data on predictors and outcomes, as well as methods used for missing data: | Yes | Yes | Yes | Yes | Yes |
| 4.1 Was there a reasonable number of participants with the result? | Yes | Yes | Yes | Yes | Yes |
| 4.2 Were continuous and categorical predictors handled correctly? | Yes | Yes | Yes | Yes | Yes |
| 4.3 Were all enrolled participants included in the analysis? | Yes | Yes | Yes | Yes | Yes |
| 4.4 Were participants with missing data treated appropriately? | Yes | Yes | Yes | Yes | Yes |
| 4.5 Was selection of predictors based on univariate analysis avoided? | Yes | Yes | Yes | Yes | Yes |
| 4.6 Were the complexities of the data (eg censoring, competing risks, sampling of controls) adequately accounted for? | Yes | Yes | Yes | Yes | Yes |
| 4.7 Were relevant model performance measures adequately evaluated? | Yes | Yes | Yes | Yes | Yes |
| 4.8 Was model overfitting and optimism factored into model performance? | Yes | Yes | Yes | Yes | Yes |
| 4.9 Do the predictors and their assigned weights in the final model correspond to the results of the multivariate analysis? | Yes | Yes | Yes | Yes | Yes |
| **Risk of bias introduced by the analysis (low/high/Unclear)** | Low | Low | Low | Low | Low |
| **Step 4: General evaluation** | There is not enough information related to the chosen population, so there is a risk of introducing bias in the selection | There is no description of the origin of the data | There is no description of the origin of the data | There is no description of the origin of the data | There is no description of the origin of the data |
| **Overall assessment of risk of bias** | High | Low | Low | Low | Low |
| **General judgment of applicability** | Low | Low | Low | Low | Low |
|  |  |  |  |  |  |
|  |  |  |  |  |  |
| **Domain 1** | **de La Sen et al. (2021) (1)** | **de La Sen et al. (2021) (2)** | **Avila-Ponce de Leon et al. (2022)** | **de Lima et al. (2021)** | **De-Leon et al. (2021)** |
| **A. Risk of bias** | **17-06-22** | **17-06-22** | **18-06-22** | **18-06-22** | **18-06-22** |
| 1.1 Were appropriate data sources used, e.g. data from cohort studies, RCTs or nested case-controls? | Unclear | Unclear | Unclear | Unclear | Unclear |
| 1.2 Were all inclusions and exclusions of participants appropriate? | No | No | No | No | No |
| Risk of bias introduced by participant selection (low/high/unclear) | High | High | High | High | High |
| **B. Applicability** |  |  |  |  |  |
| Concern that included participants and setting do not match review question (low/high/unclear) | Low | Low | Low | Low | Low |
| **Domain 2** |  |  |  |  |  |
| **A. Risk of bias - predictors** |  |  |  |  |  |
| 2.1 Were predictors defined and evaluated in a similar way for all participants? | Yes | Yes | Yes | Yes | Yes |
| 2.2 Were predictor evaluations performed without knowing the outcome data? | Yes | Yes | Yes | Yes | Yes |
| 2.3 Are all the predictors available at the time the model is intended to be used? | Yes | Yes | Yes | Yes | Yes |
| Risk of bias introduced by the predictors or their assessment (low/high/unclear) | Low | Low | Low | Low | Low |
| **B. Applicability** |  |  |  |  |  |
| Concern that the definition, evaluation, or timing of predictors in the model do not match the review question (low/high/unclear) | Low | Low | Low | Low | Low |
| **Domain 3** |  |  |  |  |  |
| **A. Risk of bias - outcome** |  |  |  |  |  |
| Was the result properly determined? | Yes | Yes | Yes | Yes | Yes |
| Was a standard or prespecified outcome definition used? | Yes | Yes | Yes | Yes | Yes |
| Were predictors excluded from the outcome definition? | Yes | Yes | Yes | Yes | Yes |
| Was the outcome similarly defined and determined for all participants? | Yes | Yes | Yes | Yes | Yes |
| Was the outcome determined without knowing the predictor information? | Yes | Yes | Yes | Yes | Yes |
| Was the time interval between the evaluation of the predictor and the determination of the result adequate? | Yes | Yes | Yes | Yes | Yes |
| Risk of bias introduced by the result or its determination (low/high/unclear) | Low | Low | Low | Low | Low |
| **B. Applicability** |  |  |  |  |  |
| If a composite outcome was used, please describe the relative frequency/distribution of each contributing outcome: | NA | NA | NA | NA | NA |
| Concern that the outcome, its definition, timing, or determination does not match the review question (low/high/unclear) | Low | Low | Low | Low | Low |
| **Domain 4** |  |  |  |  |  |
| **A. Risk of bias** |  |  |  |  |  |
| Describe the number of participants, number of candidate predictors, outcome events, and events per candidate predictor: | Yes | Yes | Yes | Yes | Yes |
| Describe how the model was developed (for example, with respect to modeling technique (eg, survival or logistic modeling), selection of predictors, and definition of risk groups): | Yes | Yes | Yes | Yes | Yes |
| Describe if and how the model was validated, either internally (eg, bootstrapping, cross-validation, split random sample) or externally (eg, temporal validation, geographic validation, different setting, different type of participants): | Yes | Yes | Yes | Yes | Yes |
| Describe the performance measures of the model, e.g. (re)calibration, discrimination, (re)classification, net profit and if adjusted for optimism: | Yes | Yes | Yes | Yes | Yes |
| Describe the participants who were excluded from the analysis: | Yes | Yes | Yes | Yes | Yes |
| Describe missing data on predictors and outcomes, as well as methods used for missing data: | Yes | Yes | Yes | Yes | Yes |
| 4.1 Was there a reasonable number of participants with the result? | Yes | Yes | Yes | Yes | Yes |
| 4.2 Were continuous and categorical predictors handled correctly? | Yes | Yes | Yes | Yes | Yes |
| 4.3 Were all enrolled participants included in the analysis? | Yes | Yes | Yes | Yes | Yes |
| 4.4 Were participants with missing data treated appropriately? | Yes | Yes | Yes | Yes | Yes |
| 4.5 Was selection of predictors based on univariate analysis avoided? | Yes | Yes | Yes | Yes | Yes |
| 4.6 Were the complexities of the data (eg censoring, competing risks, sampling of controls) adequately accounted for? | Yes | Yes | Yes | Yes | Yes |
| 4.7 Were relevant model performance measures adequately evaluated? | Yes | Yes | Yes | Yes | Yes |
| 4.8 Was model overfitting and optimism factored into model performance? | Yes | Yes | Yes | Yes | Yes |
| 4.9 Do the predictors and their assigned weights in the final model correspond to the results of the multivariate analysis? | Yes | Yes | Yes | Yes | Yes |
| **Risk of bias introduced by the analysis (low/high/Unclear)** | Low | Low | Low | Low | Low |
| **Step 4: General evaluation** | There is not enough information related to the chosen population, so there is a risk of introducing bias in the selection | There is no description of the origin of the data | There is no description of the origin of the data | There is no description of the origin of the data | There is no description of the origin of the data |
| **Overall assessment of risk of bias** | High | High | High | High | High |
| **General judgment of applicability** | Low | Low | Low | Low | Low |
|  |  |  |  |  |  |
|  |  |  |  |  |  |
| **Domain 1** | **Debrabant et al. (2021)** | **Demongeot et al. (2022)** | **DeWitt et al. (2021)** | **Di Domenico et al. (2021)** | **Di Fusco et al. (2022)** |
| **A. Risk of bias** | **18-06-22** | **18-06-22** | **18-06-22** | **18-06-22** | **18-06-22** |
| 1.1 Were appropriate data sources used, e.g. data from cohort studies, RCTs or nested case-controls? | Yes | Yes | No | No | Yes |
| 1.2 Were all inclusions and exclusions of participants appropriate? | Yes | Yes | Unclear | Unclear | Yes |
| Risk of bias introduced by participant selection (low/high/unclear) | Low | Low | High | High | Low |
| **B. Applicability** |  |  |  |  |  |
| Concern that included participants and setting do not match review question (low/high/unclear) | Low | Low | Low | Low | Low |
| **Domain 2** |  |  |  |  |  |
| **A. Risk of bias - predictors** |  |  |  |  |  |
| 2.1 Were predictors defined and evaluated in a similar way for all participants? | Yes | Yes | No | No | Yes |
| 2.2 Were predictor evaluations performed without knowing the outcome data? | Yes | Yes | Unclear | Unclear | Yes |
| 2.3 Are all the predictors available at the time the model is intended to be used? | Yes | Yes | Unclear | Unclear | Yes |
| Risk of bias introduced by the predictors or their assessment (low/high/unclear) | Low | Low | High | High | Low |
| **B. Applicability** |  |  |  |  |  |
| Concern that the definition, evaluation, or timing of predictors in the model do not match the review question (low/high/unclear) | Low | Low | Unclear | Unclear | Low |
| **Domain 3** |  |  |  |  |  |
| **A. Risk of bias - outcome** |  |  |  |  |  |
| Was the result properly determined? | Yes | Yes | Yes | Yes | Yes |
| Was a standard or prespecified outcome definition used? | Yes | Yes | No | No | Yes |
| Were predictors excluded from the outcome definition? | Yes | Yes | Unclear | Unclear | Yes |
| Was the outcome similarly defined and determined for all participants? | Yes | Yes | Unclear | Unclear | Yes |
| Was the outcome determined without knowing the predictor information? | Yes | Yes | Unclear | Unclear | Yes |
| Was the time interval between the evaluation of the predictor and the determination of the result adequate? | Yes | Yes | Yes | Yes | Yes |
| Risk of bias introduced by the result or its determination (low/high/unclear) | Low | Low | High | High | Low |
| **B. Applicability** |  |  |  |  |  |
| If a composite outcome was used, please describe the relative frequency/distribution of each contributing outcome: | NA | NA | Unclear | Unclear | NA |
| Concern that the outcome, its definition, timing, or determination does not match the review question (low/high/unclear) | Low | Low | Unclear | Unclear | Low |
| **Domain 4** |  |  |  |  |  |
| **A. Risk of bias** |  |  |  |  |  |
| Describe the number of participants, number of candidate predictors, outcome events, and events per candidate predictor: | Yes | Yes | No | No | Yes |
| Describe how the model was developed (for example, with respect to modeling technique (eg, survival or logistic modeling), selection of predictors, and definition of risk groups): | Yes | Yes | Unclear | Unclear | Yes |
| Describe if and how the model was validated, either internally (eg, bootstrapping, cross-validation, split random sample) or externally (eg, temporal validation, geographic validation, different setting, different type of participants): | Yes | Yes | Unclear | Unclear | Yes |
| Describe the performance measures of the model, e.g. (re)calibration, discrimination, (re)classification, net profit and if adjusted for optimism: | Yes | Yes | Unclear | Unclear | Yes |
| Describe the participants who were excluded from the analysis: | Yes | Yes | No | No | Yes |
| Describe missing data on predictors and outcomes, as well as methods used for missing data: | Yes | Yes | No | No | Yes |
| 4.1 Was there a reasonable number of participants with the result? | Yes | Yes | Yes | Yes | Yes |
| 4.2 Were continuous and categorical predictors handled correctly? | Yes | Yes | Yes | Yes | Yes |
| 4.3 Were all enrolled participants included in the analysis? | Yes | Yes | Yes | Yes | Yes |
| 4.4 Were participants with missing data treated appropriately? | Yes | Yes | Yes | Yes | Yes |
| 4.5 Was selection of predictors based on univariate analysis avoided? | Yes | Yes | Yes | Yes | Yes |
| 4.6 Were the complexities of the data (eg censoring, competing risks, sampling of controls) adequately accounted for? | Yes | Yes | Yes | Yes | Yes |
| 4.7 Were relevant model performance measures adequately evaluated? | Yes | Yes | Yes | Yes | Yes |
| 4.8 Was model overfitting and optimism factored into model performance? | Yes | Yes | Yes | Yes | Yes |
| 4.9 Do the predictors and their assigned weights in the final model correspond to the results of the multivariate analysis? | Yes | Yes | No | No | Yes |
| **Risk of bias introduced by the analysis (low/high/Unclear)** | Low | Low | High | High | Low |
| **Step 4: General evaluation** | **-** | **-** | This study does not have many details required to define the model | This study does not have many details required to define the model | **-** |
| **Overall assessment of risk of bias** | Low | Low | High | High | Low |
| **General judgment of applicability** | Low | Low | High | High | Low |
|  |  |  |  |  |  |
|  |  |  |  |  |  |
| **Domain 1** | **Diagne et al. (2021)** | **Diarra et al. (2022)** | **Dick et al. (2021)** | **Dimeglio et al. (2021) (1)** | **Dimeglio et al. (2021)** |
| **A. Risk of bias** | **18-06-22** | **18-06-22** | **18-06-22** | **18-06-22** | **18-06-22** |
| 1.1 Were appropriate data sources used, e.g. data from cohort studies, RCTs or nested case-controls? | Yes | Yes | No | Yes | Yes |
| 1.2 Were all inclusions and exclusions of participants appropriate? | Yes | Yes | Unclear | No | No |
| Risk of bias introduced by participant selection (low/high/unclear) | Low | Low | High | Low | Low |
| **B. Applicability** |  |  |  |  |  |
| Concern that included participants and setting do not match review question (low/high/unclear) | Low | Low | Low | Low | Low |
| **Domain 2** |  |  |  |  |  |
| **A. Risk of bias - predictors** |  |  |  |  |  |
| 2.1 Were predictors defined and evaluated in a similar way for all participants? | Yes | Yes | No | Yes | Yes |
| 2.2 Were predictor evaluations performed without knowing the outcome data? | Yes | Yes | Unclear | Yes | Yes |
| 2.3 Are all the predictors available at the time the model is intended to be used? | Yes | Yes | Unclear | Yes | Yes |
| Risk of bias introduced by the predictors or their assessment (low/high/unclear) | Low | Low | High | Low | Low |
| **B. Applicability** |  |  |  |  |  |
| Concern that the definition, evaluation, or timing of predictors in the model do not match the review question (low/high/unclear) | Low | Low | Unclear | Low | Low |
| **Domain 3** |  |  |  |  |  |
| **A. Risk of bias - outcome** |  |  |  |  |  |
| Was the result properly determined? | Yes | Yes | Yes | Yes | Yes |
| Was a standard or prespecified outcome definition used? | Yes | Yes | No | Yes | Yes |
| Were predictors excluded from the outcome definition? | Yes | Yes | Unclear | Yes | Yes |
| Was the outcome similarly defined and determined for all participants? | Yes | Yes | Unclear | Yes | Yes |
| Was the outcome determined without knowing the predictor information? | Yes | Yes | Unclear | Yes | Yes |
| Was the time interval between the evaluation of the predictor and the determination of the result adequate? | Yes | Yes | Yes | Yes | Yes |
| Risk of bias introduced by the result or its determination (low/high/unclear) | Low | Low | High | Low | Low |
| **B. Applicability** |  |  |  |  |  |
| If a composite outcome was used, please describe the relative frequency/distribution of each contributing outcome: | NA | NA | Unclear | NA | NA |
| Concern that the outcome, its definition, timing, or determination does not match the review question (low/high/unclear) | Low | Low | Unclear | Low | Low |
| **Domain 4** |  |  |  |  |  |
| **A. Risk of bias** |  |  |  |  |  |
| Describe the number of participants, number of candidate predictors, outcome events, and events per candidate predictor: | Yes | Yes | No | Yes | Yes |
| Describe how the model was developed (for example, with respect to modeling technique (eg, survival or logistic modeling), selection of predictors, and definition of risk groups): | Yes | Yes | Unclear | Yes | Yes |
| Describe if and how the model was validated, either internally (eg, bootstrapping, cross-validation, split random sample) or externally (eg, temporal validation, geographic validation, different setting, different type of participants): | Yes | Yes | Unclear | Yes | Yes |
| Describe the performance measures of the model, e.g. (re)calibration, discrimination, (re)classification, net profit and if adjusted for optimism: | Yes | Yes | Unclear | Yes | Yes |
| Describe the participants who were excluded from the analysis: | Yes | Yes | No | Yes | Yes |
| Describe missing data on predictors and outcomes, as well as methods used for missing data: | Yes | Yes | No | Yes | Yes |
| 4.1 Was there a reasonable number of participants with the result? | Yes | Yes | Yes | Yes | Yes |
| 4.2 Were continuous and categorical predictors handled correctly? | Yes | Yes | Yes | Yes | Yes |
| 4.3 Were all enrolled participants included in the analysis? | Yes | Yes | Yes | Yes | Yes |
| 4.4 Were participants with missing data treated appropriately? | Yes | Yes | Yes | Yes | Yes |
| 4.5 Was selection of predictors based on univariate analysis avoided? | Yes | Yes | Yes | Yes | Yes |
| 4.6 Were the complexities of the data (eg censoring, competing risks, sampling of controls) adequately accounted for? | Yes | Yes | Yes | Yes | Yes |
| 4.7 Were relevant model performance measures adequately evaluated? | Yes | Yes | Yes | Yes | Yes |
| 4.8 Was model overfitting and optimism factored into model performance? | Yes | Yes | Yes | Yes | Yes |
| 4.9 Do the predictors and their assigned weights in the final model correspond to the results of the multivariate analysis? | Yes | Yes | No | Yes | Yes |
| **Risk of bias introduced by the analysis (low/high/Unclear)** | Low | Low | High | Low | Low |
| **Step 4: General evaluation** | **-** | **-** | This study does not have many details required to define the model | **-** | **-** |
| **Overall assessment of risk of bias** | Low | Low | High | Low | Low |
| **General judgment of applicability** | Low | Low | High | Low | Low |
|  |  |  |  |  |  |
|  |  |  |  |  |  |
| **Domain 1** | **Di Domenico et al. (2021)** | **Dönges et al. (2021) ()** | **Du et al. (2022) (1)** | **Du et al. (2022)** |  |
| **A. Risk of bias** | **18-06-22** | **18-06-22** | **18-06-22** | **18-06-22** |  |
| 1.1 Were appropriate data sources used, e.g. data from cohort studies, RCTs or nested case-controls? | Yes | Yes | Yes | Yes |  |
| 1.2 Were all inclusions and exclusions of participants appropriate? | Unclear | No | Unclear | No |  |
| Risk of bias introduced by participant selection (low/high/unclear) | Low | Low | Low | Low |  |
| **B. Applicability** |  |  |  |  |  |
| Concern that included participants and setting do not match review question (low/high/unclear) | Low | Low | Low | Low |  |
| **Domain 2** |  |  |  |  |  |
| **A. Risk of bias - predictors** |  |  |  |  |  |
| 2.1 Were predictors defined and evaluated in a similar way for all participants? | Yes | Yes | Yes | Yes |  |
| 2.2 Were predictor evaluations performed without knowing the outcome data? | Yes | Yes | Yes | Yes |  |
| 2.3 Are all the predictors available at the time the model is intended to be used? | Yes | Yes | Yes | Yes |  |
| Risk of bias introduced by the predictors or their assessment (low/high/unclear) | Low | Low | Low | Low |  |
| **B. Applicability** |  |  |  |  |  |
| Concern that the definition, evaluation, or timing of predictors in the model do not match the review question (low/high/unclear) | Low | Low | Low | Low |  |
| **Domain 3** |  |  |  |  |  |
| **A. Risk of bias - outcome** |  |  |  |  |  |
| Was the result properly determined? | Yes | Yes | Yes | Yes |  |
| Was a standard or prespecified outcome definition used? | Yes | Yes | Unclear | Yes |  |
| Were predictors excluded from the outcome definition? | Yes | Yes | Yes | Yes |  |
| Was the outcome similarly defined and determined for all participants? | Yes | Yes | Yes | Yes |  |
| Was the outcome determined without knowing the predictor information? | Yes | Yes | Yes | Yes |  |
| Was the time interval between the evaluation of the predictor and the determination of the result adequate? | Yes | Yes | Yes | Yes |  |
| Risk of bias introduced by the result or its determination (low/high/unclear) | Low | Low | Low | Low |  |
| **B. Applicability** |  |  |  |  |  |
| If a composite outcome was used, please describe the relative frequency/distribution of each contributing outcome: | NA | NA | Yes | NA |  |
| Concern that the outcome, its definition, timing, or determination does not match the review question (low/high/unclear) | Low | Low | Low | Low |  |
| **Domain 4** |  |  |  |  |  |
| **A. Risk of bias** |  |  |  |  |  |
| Describe the number of participants, number of candidate predictors, outcome events, and events per candidate predictor: | Yes | Yes | Yes | Yes |  |
| Describe how the model was developed (for example, with respect to modeling technique (eg, survival or logistic modeling), selection of predictors, and definition of risk groups): | Yes | Yes | Yes | Yes |  |
| Describe if and how the model was validated, either internally (eg, bootstrapping, cross-validation, split random sample) or externally (eg, temporal validation, geographic validation, different setting, different type of participants): | Yes | Yes | Yes | Yes |  |
| Describe the performance measures of the model, e.g. (re)calibration, discrimination, (re)classification, net profit and if adjusted for optimism: | Yes | Yes | Yes | Yes |  |
| Describe the participants who were excluded from the analysis: | Yes | Yes | Unclear | Yes |  |
| Describe missing data on predictors and outcomes, as well as methods used for missing data: | Yes | Yes | Yes | Yes |  |
| 4.1 Was there a reasonable number of participants with the result? | Yes | Yes | Yes | Yes |  |
| 4.2 Were continuous and categorical predictors handled correctly? | Yes | Yes | Yes | Yes |  |
| 4.3 Were all enrolled participants included in the analysis? | Yes | Yes | Yes | Yes |  |
| 4.4 Were participants with missing data treated appropriately? | Yes | Yes | Yes | Yes |  |
| 4.5 Was selection of predictors based on univariate analysis avoided? | Yes | Yes | Yes | Yes |  |
| 4.6 Were the complexities of the data (eg censoring, competing risks, sampling of controls) adequately accounted for? | Yes | Yes | Yes | Yes |  |
| 4.7 Were relevant model performance measures adequately evaluated? | Yes | Yes | Yes | Yes |  |
| 4.8 Was model overfitting and optimism factored into model performance? | Yes | Yes | Yes | Yes |  |
| 4.9 Do the predictors and their assigned weights in the final model correspond to the results of the multivariate analysis? | Yes | Yes | Yes | Yes |  |
| **Risk of bias introduced by the analysis (low/high/Unclear)** | Low | Low | Low | Low |  |
| **Step 4: General evaluation** | **-** | **-** | **-** | **-** |  |
| **Overall assessment of risk of bias** | Low | Low | Low | Low |  |
| **General judgment of applicability** | Low | Low | Low | Low |  |
|  |  |  |  |  |  |
|  |  |  |  |  |  |
| **Domain 1** | **Dyson et al. (2021)** | **España et al. (2021)** | **Estadilla et al. (2021)** | **Expósito et al. (2022)** |  |
| **A. Risk of bias** | **18-06-22** | **18-06-22** | **18-06-22** | **18-06-22** |  |
| 1.1 Were appropriate data sources used, e.g. data from cohort studies, RCTs or nested case-controls? | Yes | Yes | Yes | No |  |
| 1.2 Were all inclusions and exclusions of participants appropriate? | No | Yes | No | Unclear |  |
| Risk of bias introduced by participant selection (low/high/unclear) | Low | Low | Low | High |  |
| **B. Applicability** |  |  |  |  |  |
| Concern that included participants and setting do not match review question (low/high/unclear) | Low | Low | Unclear | Low |  |
| **Domain 2** |  |  |  |  |  |
| **A. Risk of bias - predictors** |  |  |  |  |  |
| 2.1 Were predictors defined and evaluated in a similar way for all participants? | Yes | Yes | Yes | Yes |  |
| 2.2 Were predictor evaluations performed without knowing the outcome data? | Yes | Yes | Yes | Yes |  |
| 2.3 Are all the predictors available at the time the model is intended to be used? | Yes | Yes | Yes | Yes |  |
| Risk of bias introduced by the predictors or their assessment (low/high/unclear) | Low | Low | Low | Low |  |
| **B. Applicability** |  |  |  |  |  |
| Concern that the definition, evaluation, or timing of predictors in the model do not match the review question (low/high/unclear) | Low | Low | Low | Low |  |
| **Domain 3** |  |  |  |  |  |
| **A. Risk of bias - outcome** |  |  |  |  |  |
| Was the result properly determined? | Yes | Yes | Yes | Yes |  |
| Was a standard or prespecified outcome definition used? | Yes | Yes | Yes | Yes |  |
| Were predictors excluded from the outcome definition? | Yes | Yes | Yes | Yes |  |
| Was the outcome similarly defined and determined for all participants? | Yes | Yes | Yes | Yes |  |
| Was the outcome determined without knowing the predictor information? | Yes | Yes | Yes | Yes |  |
| Was the time interval between the evaluation of the predictor and the determination of the result adequate? | Yes | Yes | Yes | Yes |  |
| Risk of bias introduced by the result or its determination (low/high/unclear) | Low | Low | Low | Low |  |
| **B. Applicability** |  |  |  |  |  |
| If a composite outcome was used, please describe the relative frequency/distribution of each contributing outcome: | NA | NA | Yes | NA |  |
| Concern that the outcome, its definition, timing, or determination does not match the review question (low/high/unclear) | Low | Low | Low | Low |  |
| **Domain 4** |  |  |  |  |  |
| **A. Risk of bias** |  |  |  |  |  |
| Describe the number of participants, number of candidate predictors, outcome events, and events per candidate predictor: | Yes | Yes | Yes | Yes |  |
| Describe how the model was developed (for example, with respect to modeling technique (eg, survival or logistic modeling), selection of predictors, and definition of risk groups): | Yes | Yes | Yes | Yes |  |
| Describe if and how the model was validated, either internally (eg, bootstrapping, cross-validation, split random sample) or externally (eg, temporal validation, geographic validation, different setting, different type of participants): | Yes | Yes | Yes | Unclear |  |
| Describe the performance measures of the model, e.g. (re)calibration, discrimination, (re)classification, net profit and if adjusted for optimism: | Yes | Yes | Yes | Unclear |  |
| Describe the participants who were excluded from the analysis: | Yes | Yes | Yes | No |  |
| Describe missing data on predictors and outcomes, as well as methods used for missing data: | No | No | No | Unclear |  |
| 4.1 Was there a reasonable number of participants with the result? | Yes | Yes | Yes | Unclear |  |
| 4.2 Were continuous and categorical predictors handled correctly? | Yes | Yes | Yes | Yes |  |
| 4.3 Were all enrolled participants included in the analysis? | Yes | Yes | Yes | Yes |  |
| 4.4 Were participants with missing data treated appropriately? | NA | NA | NA | No |  |
| 4.5 Was selection of predictors based on univariate analysis avoided? | Yes | Yes | Yes | Yes |  |
| 4.6 Were the complexities of the data (eg censoring, competing risks, sampling of controls) adequately accounted for? | Yes | Yes | Yes | Yes |  |
| 4.7 Were relevant model performance measures adequately evaluated? | Yes | Yes | Yes | Yes |  |
| 4.8 Was model overfitting and optimism factored into model performance? | Yes | Yes | Yes | Yes |  |
| 4.9 Do the predictors and their assigned weights in the final model correspond to the results of the multivariate analysis? | Yes | Yes | Yes | Yes |  |
| **Risk of bias introduced by the analysis (low/high/Unclear)** | Low | Low | Low | High |  |
| **Step 4: General evaluation** | **-** | **-** | **-** | This article does not have data related to the origin of the same, likewise a clear procedure in the modeling is not explained |  |
| **Overall assessment of risk of bias** | Low | Low | Low | High |  |
| **General judgment of applicability** | Low | Low | Low | High |  |
|  |  |  |  |  |  |
|  |  |  |  |  |  |
| **Domain 1** | **Faranda et al. (2021)** | **Faucher et al. (2022)** | **Fawaz et al. (2021)** | **Feng et al. (2021)** | **Feng et al. (2022)** |
| **A. Risk of bias** | **18-06-22** | **18-06-22** | **18-06-22** | **18-06-22** | **18-06-22** |
| 1.1 Were appropriate data sources used, e.g. data from cohort studies, RCTs or nested case-controls? | Yes | Yes | Yes | Unclear | Yes |
| 1.2 Were all inclusions and exclusions of participants appropriate? | No | Yes | Unclear | No | No |
| Risk of bias introduced by participant selection (low/high/unclear) | Low | Low | Low | High | Low |
| **B. Applicability** |  |  |  |  |  |
| Concern that included participants and setting do not match review question (low/high/unclear) | Low | Low | Low | Low | Low |
| **Domain 2** |  |  |  |  |  |
| **A. Risk of bias - predictors** |  |  |  |  |  |
| 2.1 Were predictors defined and evaluated in a similar way for all participants? | Yes | Yes | Yes | No | Yes |
| 2.2 Were predictor evaluations performed without knowing the outcome data? | Yes | Yes | Yes | Unclear | Yes |
| 2.3 Are all the predictors available at the time the model is intended to be used? | Yes | Yes | Yes | Unclear | Yes |
| Risk of bias introduced by the predictors or their assessment (low/high/unclear) | Low | Low | Low | High | Low |
| **B. Applicability** |  |  |  |  |  |
| Concern that the definition, evaluation, or timing of predictors in the model do not match the review question (low/high/unclear) | Low | Low | Low | Unclear | Low |
| **Domain 3** |  |  |  |  |  |
| **A. Risk of bias - outcome** |  |  |  |  |  |
| Was the result properly determined? | Yes | Yes | Yes | Yes | Yes |
| Was a standard or prespecified outcome definition used? | Yes | Yes | Yes | No | Yes |
| Were predictors excluded from the outcome definition? | Yes | Yes | Yes | Unclear | Yes |
| Was the outcome similarly defined and determined for all participants? | Yes | Yes | Yes | Unclear | Yes |
| Was the outcome determined without knowing the predictor information? | Yes | Yes | Yes | Unclear | Yes |
| Was the time interval between the evaluation of the predictor and the determination of the result adequate? | Yes | Yes | Yes | Yes | Yes |
| Risk of bias introduced by the result or its determination (low/high/unclear) | Low | Low | Low | High | Low |
| **B. Applicability** |  |  |  |  |  |
| If a composite outcome was used, please describe the relative frequency/distribution of each contributing outcome: | Yes | Yes | Yes | Unclear | Yes |
| Concern that the outcome, its definition, timing, or determination does not match the review question (low/high/unclear) | Low | Low | Low | Unclear | Low |
| **Domain 4** |  |  |  |  |  |
| **A. Risk of bias** |  |  |  |  |  |
| Describe the number of participants, number of candidate predictors, outcome events, and events per candidate predictor: | Yes | Yes | Yes | No | Yes |
| Describe how the model was developed (for example, with respect to modeling technique (eg, survival or logistic modeling), selection of predictors, and definition of risk groups): | Yes | Yes | Yes | Unclear | Yes |
| Describe if and how the model was validated, either internally (eg, bootstrapping, cross-validation, split random sample) or externally (eg, temporal validation, geographic validation, different setting, different type of participants): | Yes | Yes | Yes | Unclear | Yes |
| Describe the performance measures of the model, e.g. (re)calibration, discrimination, (re)classification, net profit and if adjusted for optimism: | Yes | Yes | Yes | Unclear | Yes |
| Describe the participants who were excluded from the analysis: | Yes | Yes | Yes | No | Yes |
| Describe missing data on predictors and outcomes, as well as methods used for missing data: | No | No | No | No | No |
| 4.1 Was there a reasonable number of participants with the result? | Yes | Yes | Yes | Yes | Yes |
| 4.2 Were continuous and categorical predictors handled correctly? | Yes | Yes | Yes | Yes | Yes |
| 4.3 Were all enrolled participants included in the analysis? | Yes | Yes | Yes | Yes | Yes |
| 4.4 Were participants with missing data treated appropriately? | No | No | No | Yes | No |
| 4.5 Was selection of predictors based on univariate analysis avoided? | Yes | Yes | Yes | Yes | Yes |
| 4.6 Were the complexities of the data (eg censoring, competing risks, sampling of controls) adequately accounted for? | Yes | Yes | Yes | Yes | Yes |
| 4.7 Were relevant model performance measures adequately evaluated? | Yes | Yes | Yes | Yes | Yes |
| 4.8 Was model overfitting and optimism factored into model performance? | Yes | Yes | Yes | Yes | Yes |
| 4.9 Do the predictors and their assigned weights in the final model correspond to the results of the multivariate analysis? | Yes | Yes | Yes | No | Yes |
| **Risk of bias introduced by the analysis (low/high/Unclear)** | Low | Low | Low | High | Low |
| **Step 4: General evaluation** | **-** | **-** | **-** | This study does not have many details required to define the model | **-** |
| **Overall assessment of risk of bias** | Low | Low | Low | High | Low |
| **General judgment of applicability** | Low | Low | Low | High | Low |
|  |  |  |  |  |  |
|  |  |  |  |  |  |
| **Domain 1** | **Ferrana et al. (2021)** | **Ferreria et al. (2021)** | **Souto et al. (2022)** | **Fierro et al. (2022)** | **Flores-Arguedas et al. (2021)** |
| **A. Risk of bias** | **18-06-22** | **18-06-22** | **18-06-22** | **18-06-22** | **18-06-22** |
| 1.1 Were appropriate data sources used, e.g. data from cohort studies, RCTs or nested case-controls? | Yes | Yes | Unclear | Yes | Unclear |
| 1.2 Were all inclusions and exclusions of participants appropriate? | Yes | Unclear | No | Yes | No |
| Risk of bias introduced by participant selection (low/high/unclear) | Low | Low | High | Low | High |
| **B. Applicability** |  |  |  |  |  |
| Concern that included participants and setting do not match review question (low/high/unclear) | Low | Low | Low | Low | Low |
| **Domain 2** |  |  |  |  |  |
| **A. Risk of bias - predictors** |  |  |  |  |  |
| 2.1 Were predictors defined and evaluated in a similar way for all participants? | Unclear | Yes | Yes | Yes | Yes |
| 2.2 Were predictor evaluations performed without knowing the outcome data? | Unclear | Yes | Yes | Yes | Yes |
| 2.3 Are all the predictors available at the time the model is intended to be used? | Unclear | Yes | Yes | Yes | Yes |
| Risk of bias introduced by the predictors or their assessment (low/high/unclear) | High | Low | Low | Low | Low |
| **B. Applicability** |  |  |  |  |  |
| Concern that the definition, evaluation, or timing of predictors in the model do not match the review question (low/high/unclear) | Low | Low | Low | Low | Low |
| **Domain 3** |  |  |  |  |  |
| **A. Risk of bias - outcome** |  |  |  |  |  |
| Was the result properly determined? | Yes | Yes | Yes | Yes | Yes |
| Was a standard or prespecified outcome definition used? | Yes | Yes | Yes | Yes | Yes |
| Were predictors excluded from the outcome definition? | Yes | Yes | Yes | Yes | Yes |
| Was the outcome similarly defined and determined for all participants? | Yes | Yes | Yes | Yes | Yes |
| Was the outcome determined without knowing the predictor information? | Yes | Yes | Yes | Yes | Yes |
| Was the time interval between the evaluation of the predictor and the determination of the result adequate? | Yes | Yes | Yes | Yes | Yes |
| Risk of bias introduced by the result or its determination (low/high/unclear) | Low | Low | Low | Low | Low |
| **B. Applicability** |  |  |  |  |  |
| If a composite outcome was used, please describe the relative frequency/distribution of each contributing outcome: | Yes | Yes | Yes | Yes | Yes |
| Concern that the outcome, its definition, timing, or determination does not match the review question (low/high/unclear) | Low | Low | Low | Low | Low |
| **Domain 4** |  |  |  |  |  |
| **A. Risk of bias** |  |  |  |  |  |
| Describe the number of participants, number of candidate predictors, outcome events, and events per candidate predictor: | Yes | Yes | Yes | Yes | Yes |
| Describe how the model was developed (for example, with respect to modeling technique (eg, survival or logistic modeling), selection of predictors, and definition of risk groups): | Yes | Yes | Yes | Yes | Yes |
| Describe if and how the model was validated, either internally (eg, bootstrapping, cross-validation, split random sample) or externally (eg, temporal validation, geographic validation, different setting, different type of participants): | Yes | Yes | Yes | Yes | Yes |
| Describe the performance measures of the model, e.g. (re)calibration, discrimination, (re)classification, net profit and if adjusted for optimism: | Yes | Yes | Yes | Yes | Yes |
| Describe the participants who were excluded from the analysis: | Yes | Yes | Yes | Yes | Yes |
| Describe missing data on predictors and outcomes, as well as methods used for missing data: | No | No | No | No | No |
| 4.1 Was there a reasonable number of participants with the result? | Yes | Yes | Yes | Yes | Yes |
| 4.2 Were continuous and categorical predictors handled correctly? | Yes | Yes | Yes | Yes | Yes |
| 4.3 Were all enrolled participants included in the analysis? | Yes | Yes | Yes | Yes | Yes |
| 4.4 Were participants with missing data treated appropriately? | No | No | No | No | No |
| 4.5 Was selection of predictors based on univariate analysis avoided? | Yes | Yes | Yes | Yes | Yes |
| 4.6 Were the complexities of the data (eg censoring, competing risks, sampling of controls) adequately accounted for? | Yes | Yes | Yes | Yes | Yes |
| 4.7 Were relevant model performance measures adequately evaluated? | Yes | Yes | Yes | Yes | Yes |
| 4.8 Was model overfitting and optimism factored into model performance? | Yes | Yes | Yes | Yes | Yes |
| 4.9 Do the predictors and their assigned weights in the final model correspond to the results of the multivariate analysis? | Yes | Yes | Yes | Yes | Yes |
| **Risk of bias introduced by the analysis (low/high/Unclear)** | Low | Low | Low | Low | Low |
| **Step 4: General evaluation** | **-** | **-** | Insufficient data regarding the population | - | Insufficient data regarding the population |
| **Overall assessment of risk of bias** | Low | Low | High | Low | High |
| **General judgment of applicability** | High | Low | Low | Low | Low |
|  |  |  |  |  |  |
|  |  |  |  |  |  |
| **Domain 1** | **Foy et al. (2021)** | **Frazier et al. (2022)** | **Frieswijk et al. (2021)** | **Fuady et al. (2021)** | **Fujii et al. (2021)** |
| **A. Risk of bias** | **18-06-22** | **18-06-22** | **18-06-22** | **18-06-22** | **18-06-22** |
| 1.1 Were appropriate data sources used, e.g. data from cohort studies, RCTs or nested case-controls? | Yes | Yes | Yes | Yes | Yes |
| 1.2 Were all inclusions and exclusions of participants appropriate? | Yes | Yes | Yes | Yes | Yes |
| Risk of bias introduced by participant selection (low/high/unclear) | Low | Low | Low | Low | Low |
| **B. Applicability** |  |  |  |  |  |
| Concern that included participants and setting do not match review question (low/high/unclear) | Low | Low | Low | Low | Low |
| **Domain 2** |  |  |  |  |  |
| **A. Risk of bias - predictors** |  |  |  |  |  |
| 2.1 Were predictors defined and evaluated in a similar way for all participants? | Yes | Yes | Yes | Yes | Yes |
| 2.2 Were predictor evaluations performed without knowing the outcome data? | Yes | Yes | Yes | Yes | Yes |
| 2.3 Are all the predictors available at the time the model is intended to be used? | Yes | Yes | Yes | Yes | Yes |
| Risk of bias introduced by the predictors or their assessment (low/high/unclear) | Low | Low | Low | Low | Low |
| **B. Applicability** |  |  |  |  |  |
| Concern that the definition, evaluation, or timing of predictors in the model do not match the review question (low/high/unclear) | Low | Low | Low | Low | Low |
| **Domain 3** |  |  |  |  |  |
| **A. Risk of bias - outcome** |  |  |  |  |  |
| Was the result properly determined? | Yes | Yes | Yes | Yes | Yes |
| Was a standard or prespecified outcome definition used? | Yes | Yes | Yes | Yes | Yes |
| Were predictors excluded from the outcome definition? | Yes | Yes | Yes | Yes | Yes |
| Was the outcome similarly defined and determined for all participants? | Yes | Yes | Yes | Yes | Yes |
| Was the outcome determined without knowing the predictor information? | Yes | Yes | Yes | Yes | Yes |
| Was the time interval between the evaluation of the predictor and the determination of the result adequate? | Yes | Yes | Yes | Yes | Yes |
| Risk of bias introduced by the result or its determination (low/high/unclear) | Low | Low | Low | Low | Low |
| **B. Applicability** |  |  |  |  |  |
| If a composite outcome was used, please describe the relative frequency/distribution of each contributing outcome: | Yes | Yes | Yes | Yes | Yes |
| Concern that the outcome, its definition, timing, or determination does not match the review question (low/high/unclear) | Low | Low | Low | Low | Low |
| **Domain 4** |  |  |  |  |  |
| **A. Risk of bias** |  |  |  |  |  |
| Describe the number of participants, number of candidate predictors, outcome events, and events per candidate predictor: | Yes | Yes | Yes | Yes | Yes |
| Describe how the model was developed (for example, with respect to modeling technique (eg, survival or logistic modeling), selection of predictors, and definition of risk groups): | Yes | Yes | Yes | Yes | Yes |
| Describe if and how the model was validated, either internally (eg, bootstrapping, cross-validation, split random sample) or externally (eg, temporal validation, geographic validation, different setting, different type of participants): | Yes | Yes | Yes | Yes | Yes |
| Describe the performance measures of the model, e.g. (re)calibration, discrimination, (re)classification, net profit and if adjusted for optimism: | Yes | Yes | Yes | Yes | Yes |
| Describe the participants who were excluded from the analysis: | Yes | Yes | Yes | Yes | Yes |
| Describe missing data on predictors and outcomes, as well as methods used for missing data: | No | No | No | No | No |
| 4.1 Was there a reasonable number of participants with the result? | Yes | Yes | Yes | Yes | Yes |
| 4.2 Were continuous and categorical predictors handled correctly? | Yes | Yes | Yes | Yes | Yes |
| 4.3 Were all enrolled participants included in the analysis? | Yes | Yes | Yes | Yes | Yes |
| 4.4 Were participants with missing data treated appropriately? | No | No | No | No | No |
| 4.5 Was selection of predictors based on univariate analysis avoided? | Yes | Yes | Yes | Yes | Yes |
| 4.6 Were the complexities of the data (eg censoring, competing risks, sampling of controls) adequately accounted for? | Yes | Yes | Yes | Yes | Yes |
| 4.7 Were relevant model performance measures adequately evaluated? | Yes | Yes | Yes | Yes | Yes |
| 4.8 Was model overfitting and optimism factored into model performance? | Yes | Yes | Yes | Yes | Yes |
| 4.9 Do the predictors and their assigned weights in the final model correspond to the results of the multivariate analysis? | Yes | Yes | Yes | Yes | Yes |
| **Risk of bias introduced by the analysis (low/high/Unclear)** | Low | Low | Low | Low | Low |
| **Step 4: General evaluation** |  |  |  |  |  |
| **Overall assessment of risk of bias** | Low | Low | Low | Low | Low |
| **General judgment of applicability** | Low | Low | Low | Low | Low |
|  |  |  |  |  |  |
|  |  |  |  |  |  |
| **Domain 1** | **Furuse (2021)** | **Gabriele-Rivet et al. (2021)** | **Galanti et al. (2021)** | **Galloway et al. (2021)** | **Gandjour (2022) (1)** |
| **A. Risk of bias** | **18-06-22** | **18-06-22** | **19-06-22** | **19-06-22** | **20-06-22** |
| 1.1 Were appropriate data sources used, e.g. data from cohort studies, RCTs or nested case-controls? | Unclear | Unclear | Yes | Unclear | Unclear |
| 1.2 Were all inclusions and exclusions of participants appropriate? | No | No | Yes | No | No |
| Risk of bias introduced by participant selection (low/high/unclear) | High | High | Low | High | High |
| **B. Applicability** |  |  |  |  |  |
| Concern that included participants and setting do not match review question (low/high/unclear) | Low | Low | Low | Low | Low |
| **Domain 2** |  |  |  |  |  |
| **A. Risk of bias - predictors** |  |  |  |  |  |
| 2.1 Were predictors defined and evaluated in a similar way for all participants? | No | No | Yes | No | Yes |
| 2.2 Were predictor evaluations performed without knowing the outcome data? | Unclear | Unclear | Yes | Unclear | Yes |
| 2.3 Are all the predictors available at the time the model is intended to be used? | Unclear | Unclear | Yes | Unclear | Yes |
| Risk of bias introduced by the predictors or their assessment (low/high/unclear) | High | High | Low | High | Low |
| **B. Applicability** |  |  |  |  |  |
| Concern that the definition, evaluation, or timing of predictors in the model do not match the review question (low/high/unclear) | Unclear | Unclear | Low | Unclear | Low |
| **Domain 3** |  |  |  |  |  |
| **A. Risk of bias - outcome** |  |  |  |  |  |
| Was the result properly determined? | Yes | Yes | Yes | Yes | Yes |
| Was a standard or prespecified outcome definition used? | No | No | Yes | No | Yes |
| Were predictors excluded from the outcome definition? | Unclear | Unclear | Yes | Unclear | Yes |
| Was the outcome similarly defined and determined for all participants? | Unclear | Unclear | Yes | Unclear | Yes |
| Was the outcome determined without knowing the predictor information? | Unclear | Unclear | Yes | Unclear | Yes |
| Was the time interval between the evaluation of the predictor and the determination of the result adequate? | Yes | Yes | Yes | Yes | Yes |
| Risk of bias introduced by the result or its determination (low/high/unclear) | High | High | Low | High | Low |
| **B. Applicability** |  |  |  |  |  |
| If a composite outcome was used, please describe the relative frequency/distribution of each contributing outcome: | Unclear | Unclear | Yes | Unclear | Yes |
| Concern that the outcome, its definition, timing, or determination does not match the review question (low/high/unclear) | Unclear | Unclear | Low | Unclear | Low |
| **Domain 4** |  |  |  |  |  |
| **A. Risk of bias** |  |  |  |  |  |
| Describe the number of participants, number of candidate predictors, outcome events, and events per candidate predictor: | No | No | Yes | No | Yes |
| Describe how the model was developed (for example, with respect to modeling technique (eg, survival or logistic modeling), selection of predictors, and definition of risk groups): | Unclear | Unclear | Yes | Unclear | Yes |
| Describe if and how the model was validated, either internally (eg, bootstrapping, cross-validation, split random sample) or externally (eg, temporal validation, geographic validation, different setting, different type of participants): | Unclear | Unclear | Yes | Unclear | Yes |
| Describe the performance measures of the model, e.g. (re)calibration, discrimination, (re)classification, net profit and if adjusted for optimism: | Unclear | Unclear | Yes | Unclear | Yes |
| Describe the participants who were excluded from the analysis: | No | No | Yes | No | Yes |
| Describe missing data on predictors and outcomes, as well as methods used for missing data: | No | No | No | No | No |
| 4.1 Was there a reasonable number of participants with the result? | Yes | Yes | Yes | Yes | Yes |
| 4.2 Were continuous and categorical predictors handled correctly? | Yes | Yes | Yes | Yes | Yes |
| 4.3 Were all enrolled participants included in the analysis? | Yes | Yes | Yes | Yes | Yes |
| 4.4 Were participants with missing data treated appropriately? | Yes | Yes | No | Yes | No |
| 4.5 Was selection of predictors based on univariate analysis avoided? | Yes | Yes | Yes | Yes | Yes |
| 4.6 Were the complexities of the data (eg censoring, competing risks, sampling of controls) adequately accounted for? | Yes | Yes | Yes | Yes | Yes |
| 4.7 Were relevant model performance measures adequately evaluated? | Yes | Yes | Yes | Yes | Yes |
| 4.8 Was model overfitting and optimism factored into model performance? | Yes | Yes | Yes | Yes | Yes |
| 4.9 Do the predictors and their assigned weights in the final model correspond to the results of the multivariate analysis? | No | No | Yes | No | Yes |
| **Risk of bias introduced by the analysis (low/high/Unclear)** | High | High | Low | High | Low |
| **Step 4: General evaluation** | This study does not have many details required to define the model | This study does not have many details required to define the model | - | This study does not have many details required to define the model | - |
| **Overall assessment of risk of bias** | High | High | Low | High | High |
| **General judgment of applicability** | High | High | Low | High | Low |
|  |  |  |  |  |  |
|  |  |  |  |  |  |
| **Domain 1** | **Gandjour (2022) (2)** | **Ganesan et al. (2021)** | **García et al. (2022)** | **Gavish et al. (2022) (1)** | **Gavish et al. (2022) (2)** |
| **A. Risk of bias** | **20/62022** | **21-06-22** | **21-06-22** | **22-06-22** | **22-06-22** |
| 1.1 Were appropriate data sources used, e.g. data from cohort studies, RCTs or nested case-controls? | Yes | Unclear | Yes | Yes | Yes |
| 1.2 Were all inclusions and exclusions of participants appropriate? | Yes | No | Yes | Yes | Yes |
| Risk of bias introduced by participant selection (low/high/unclear) | Low | High | Low | Low | Low |
| **B. Applicability** |  |  |  |  |  |
| Concern that included participants and setting do not match review question (low/high/unclear) | Low | Low | Low | Low | Low |
| **Domain 2** |  |  |  |  |  |
| **A. Risk of bias - predictors** |  |  |  |  |  |
| 2.1 Were predictors defined and evaluated in a similar way for all participants? | Yes | Yes | Yes | Yes | Yes |
| 2.2 Were predictor evaluations performed without knowing the outcome data? | Yes | Yes | Yes | Yes | Yes |
| 2.3 Are all the predictors available at the time the model is intended to be used? | Yes | Yes | Yes | Yes | Yes |
| Risk of bias introduced by the predictors or their assessment (low/high/unclear) | Low | Low | Low | Low | Low |
| **B. Applicability** |  |  |  |  |  |
| Concern that the definition, evaluation, or timing of predictors in the model do not match the review question (low/high/unclear) | Low | Low | Low | Low | Low |
| **Domain 3** |  |  |  |  |  |
| **A. Risk of bias - outcome** |  |  |  |  |  |
| Was the result properly determined? | Yes | Yes | Yes | Yes | Yes |
| Was a standard or prespecified outcome definition used? | Yes | Yes | Yes | Yes | Yes |
| Were predictors excluded from the outcome definition? | Yes | Yes | Yes | Yes | Yes |
| Was the outcome similarly defined and determined for all participants? | Yes | Yes | Yes | Yes | Yes |
| Was the outcome determined without knowing the predictor information? | Yes | Yes | Yes | Yes | Yes |
| Was the time interval between the evaluation of the predictor and the determination of the result adequate? | Yes | Yes | Yes | Yes | Yes |
| Risk of bias introduced by the result or its determination (low/high/unclear) | Low | Low | Low | Low | Low |
| **B. Applicability** |  |  |  |  |  |
| If a composite outcome was used, please describe the relative frequency/distribution of each contributing outcome: | Yes | Yes | Yes | Yes | Yes |
| Concern that the outcome, its definition, timing, or determination does not match the review question (low/high/unclear) | Low | Low | Low | Low | Low |
| **Domain 4** |  |  |  |  |  |
| **A. Risk of bias** |  |  |  |  |  |
| Describe the number of participants, number of candidate predictors, outcome events, and events per candidate predictor: | Yes | Yes | Yes | Yes | Yes |
| Describe how the model was developed (for example, with respect to modeling technique (eg, survival or logistic modeling), selection of predictors, and definition of risk groups): | Yes | Yes | Yes | Yes | Yes |
| Describe if and how the model was validated, either internally (eg, bootstrapping, cross-validation, split random sample) or externally (eg, temporal validation, geographic validation, different setting, different type of participants): | Yes | Yes | Yes | Yes | Yes |
| Describe the performance measures of the model, e.g. (re)calibration, discrimination, (re)classification, net profit and if adjusted for optimism: | Yes | Yes | Yes | Yes | Yes |
| Describe the participants who were excluded from the analysis: | Yes | Yes | Yes | Yes | Yes |
| Describe missing data on predictors and outcomes, as well as methods used for missing data: | No | No | No | No | No |
| 4.1 Was there a reasonable number of participants with the result? | Yes | Yes | Yes | Yes | Yes |
| 4.2 Were continuous and categorical predictors handled correctly? | Yes | Yes | Yes | Yes | Yes |
| 4.3 Were all enrolled participants included in the analysis? | Yes | Yes | Yes | Yes | Yes |
| 4.4 Were participants with missing data treated appropriately? | No | No | No | No | No |
| 4.5 Was selection of predictors based on univariate analysis avoided? | Yes | Yes | Yes | Yes | Yes |
| 4.6 Were the complexities of the data (eg censoring, competing risks, sampling of controls) adequately accounted for? | Yes | Yes | Yes | Yes | Yes |
| 4.7 Were relevant model performance measures adequately evaluated? | Yes | Yes | Yes | Yes | Yes |
| 4.8 Was model overfitting and optimism factored into model performance? | Yes | Yes | Yes | Yes | Yes |
| 4.9 Do the predictors and their assigned weights in the final model correspond to the results of the multivariate analysis? | Yes | Yes | Yes | Yes | Yes |
| **Risk of bias introduced by the analysis (low/high/Unclear)** | Low | Low | Low | Low | Low |
| **Step 4: General evaluation** | **-** | This study does not have many details required to define the model | **-** | **-** | **-** |
| **Overall assessment of risk of bias** | Low | High | Low | Low | Low |
| **General judgment of applicability** | Low | Low | Low | Low | Low |
|  |  |  |  |  |  |
|  |  |  |  |  |  |
| **Domain 1** | **Genari et al. (2022)** | **Ghafari et al. (2022)** | **Ghosh et al. (2020)** | **Giacopelli (2020)** | **Di Giamberardino et al. (2021)** |
| **A. Risk of bias** | **25-06-22** | **25-06-22** | **25-06-22** | **25-06-22** | **25-06-22** |
| 1.1 Were appropriate data sources used, e.g. data from cohort studies, RCTs or nested case-controls? | Yes | Unclear | Yes | Unclear | Unclear |
| 1.2 Were all inclusions and exclusions of participants appropriate? | Yes | No | Yes | Unclear | Unclear |
| Risk of bias introduced by participant selection (low/high/unclear) | Low | High | Low | High | High |
| **B. Applicability** |  |  |  |  |  |
| Concern that included participants and setting do not match review question (low/high/unclear) | Low | Low | Low | Low | Low |
| **Domain 2** |  |  |  |  |  |
| **A. Risk of bias - predictors** |  |  |  |  |  |
| 2.1 Were predictors defined and evaluated in a similar way for all participants? | Yes | Yes | Yes | No | Yes |
| 2.2 Were predictor evaluations performed without knowing the outcome data? | Yes | Yes | Yes | Unclear | Yes |
| 2.3 Are all the predictors available at the time the model is intended to be used? | Yes | Yes | Yes | Unclear | Yes |
| Risk of bias introduced by the predictors or their assessment (low/high/unclear) | Low | Low | Low | High | Low |
| **B. Applicability** |  |  |  |  |  |
| Concern that the definition, evaluation, or timing of predictors in the model do not match the review question (low/high/unclear) | Low | Low | Low | Unclear | Low |
| **Domain 3** |  |  |  |  |  |
| **A. Risk of bias - outcome** |  |  |  |  |  |
| Was the result properly determined? | Yes | Yes | Yes | Yes | Yes |
| Was a standard or prespecified outcome definition used? | Yes | Yes | Yes | No | Yes |
| Were predictors excluded from the outcome definition? | Yes | Yes | Yes | Unclear | Yes |
| Was the outcome similarly defined and determined for all participants? | Yes | Yes | Yes | Unclear | Yes |
| Was the outcome determined without knowing the predictor information? | Yes | Yes | Yes | Unclear | Yes |
| Was the time interval between the evaluation of the predictor and the determination of the result adequate? | Yes | Yes | Yes | Yes | Yes |
| Risk of bias introduced by the result or its determination (low/high/unclear) | Low | Low | Low | High | Low |
| **B. Applicability** |  |  |  |  |  |
| If a composite outcome was used, please describe the relative frequency/distribution of each contributing outcome: | Yes | Yes | Yes | Unclear | Yes |
| Concern that the outcome, its definition, timing, or determination does not match the review question (low/high/unclear) | Low | Low | Low | Unclear | Low |
| **Domain 4** |  |  |  |  |  |
| **A. Risk of bias** |  |  |  |  |  |
| Describe the number of participants, number of candidate predictors, outcome events, and events per candidate predictor: | Yes | Yes | Yes | No | Yes |
| Describe how the model was developed (for example, with respect to modeling technique (eg, survival or logistic modeling), selection of predictors, and definition of risk groups): | Yes | Yes | Yes | Unclear | Yes |
| Describe if and how the model was validated, either internally (eg, bootstrapping, cross-validation, split random sample) or externally (eg, temporal validation, geographic validation, different setting, different type of participants): | Yes | Yes | Yes | Unclear | Yes |
| Describe the performance measures of the model, e.g. (re)calibration, discrimination, (re)classification, net profit and if adjusted for optimism: | Yes | Yes | Yes | Unclear | Yes |
| Describe the participants who were excluded from the analysis: | Yes | Yes | Yes | No | Yes |
| Describe missing data on predictors and outcomes, as well as methods used for missing data: | No | No | No | No | No |
| 4.1 Was there a reasonable number of participants with the result? | Yes | Yes | Yes | Yes | Yes |
| 4.2 Were continuous and categorical predictors handled correctly? | Yes | Yes | Yes | Yes | Yes |
| 4.3 Were all enrolled participants included in the analysis? | Yes | Yes | Yes | Yes | Yes |
| 4.4 Were participants with missing data treated appropriately? | No | No | No | Yes | No |
| 4.5 Was selection of predictors based on univariate analysis avoided? | Yes | Yes | Yes | Yes | Yes |
| 4.6 Were the complexities of the data (eg censoring, competing risks, sampling of controls) adequately accounted for? | Yes | Yes | Yes | Yes | Yes |
| 4.7 Were relevant model performance measures adequately evaluated? | Yes | Yes | Yes | Yes | Yes |
| 4.8 Was model overfitting and optimism factored into model performance? | Yes | Yes | Yes | Yes | Yes |
| 4.9 Do the predictors and their assigned weights in the final model correspond to the results of the multivariate analysis? | Yes | Yes | Yes | No | Yes |
| **Risk of bias introduced by the analysis (low/high/Unclear)** | Low | Low | Low | High | Low |
| **Step 4: General evaluation** | **-** | This study does not have many details required to define the model | - | This study does not have many details required to define the model | There is no clear information on the origin of the data |
| **Overall assessment of risk of bias** | Low | High | Low | High | High |
| **General judgment of applicability** | Low | Low | Low | High | Low |
|  |  |  |  |  |  |
|  |  |  |  |  |  |
| **Domain 1** | **Giardina et al. (2022)** | **Glover et al. (2020)** | **Glover et al. (2021)** | **Goldstein et al. (2021)** |  |
| **A. Risk of bias** | **25-06-22** | **25-06-22** | **25-06-22** | **25-06-22** |  |
| 1.1 Were appropriate data sources used, e.g. data from cohort studies, RCTs or nested case-controls? | Yes | Yes | Yes | Yes |  |
| 1.2 Were all inclusions and exclusions of participants appropriate? | Yes | Yes | Yes | Unclear |  |
| Risk of bias introduced by participant selection (low/high/unclear) | Low | Low | Low | Low |  |
| **B. Applicability** |  |  |  |  |  |
| Concern that included participants and setting do not match review question (low/high/unclear) | Low | Low | Low | Low |  |
| **Domain 2** |  |  |  |  |  |
| **A. Risk of bias - predictors** |  |  |  |  |  |
| 2.1 Were predictors defined and evaluated in a similar way for all participants? | Yes | Yes | Yes | Unclear |  |
| 2.2 Were predictor evaluations performed without knowing the outcome data? | Yes | Yes | Yes | Unclear |  |
| 2.3 Are all the predictors available at the time the model is intended to be used? | Yes | Yes | Yes | Unclear |  |
| Risk of bias introduced by the predictors or their assessment (low/high/unclear) | Low | Low | Low | High |  |
| **B. Applicability** |  |  |  |  |  |
| Concern that the definition, evaluation, or timing of predictors in the model do not match the review question (low/high/unclear) | Low | Low | Low | Low |  |
| **Domain 3** |  |  |  |  |  |
| **A. Risk of bias - outcome** |  |  |  |  |  |
| Was the result properly determined? | Yes | Yes | Yes | Yes |  |
| Was a standard or prespecified outcome definition used? | Yes | Yes | Yes | Yes |  |
| Were predictors excluded from the outcome definition? | Yes | Yes | Yes | Yes |  |
| Was the outcome similarly defined and determined for all participants? | Yes | Yes | Yes | Yes |  |
| Was the outcome determined without knowing the predictor information? | Yes | Yes | Yes | Yes |  |
| Was the time interval between the evaluation of the predictor and the determination of the result adequate? | Yes | Yes | Yes | Yes |  |
| Risk of bias introduced by the result or its determination (low/high/unclear) | Low | Low | Low | Low |  |
| **B. Applicability** |  |  |  |  |  |
| If a composite outcome was used, please describe the relative frequency/distribution of each contributing outcome: | Yes | Yes | Yes | Yes |  |
| Concern that the outcome, its definition, timing, or determination does not match the review question (low/high/unclear) | Low | Low | Low | Yes |  |
| **Domain 4** |  |  |  |  |  |
| **A. Risk of bias** |  |  |  |  |  |
| Describe the number of participants, number of candidate predictors, outcome events, and events per candidate predictor: | Yes | Yes | Yes | Yes |  |
| Describe how the model was developed (for example, with respect to modeling technique (eg, survival or logistic modeling), selection of predictors, and definition of risk groups): | Yes | Yes | Yes | Yes |  |
| Describe if and how the model was validated, either internally (eg, bootstrapping, cross-validation, split random sample) or externally (eg, temporal validation, geographic validation, different setting, different type of participants): | Yes | Yes | Yes | Yes |  |
| Describe the performance measures of the model, e.g. (re)calibration, discrimination, (re)classification, net profit and if adjusted for optimism: | Yes | Yes | Yes | Yes |  |
| Describe the participants who were excluded from the analysis: | Yes | Yes | Yes | Yes |  |
| Describe missing data on predictors and outcomes, as well as methods used for missing data: | Yes | Yes | Yes | No |  |
| 4.1 Was there a reasonable number of participants with the result? | Yes | Yes | Yes | Yes |  |
| 4.2 Were continuous and categorical predictors handled correctly? | Yes | Yes | Yes | Yes |  |
| 4.3 Were all enrolled participants included in the analysis? | Yes | Yes | Yes | Yes |  |
| 4.4 Were participants with missing data treated appropriately? | Yes | Yes | Yes | No |  |
| 4.5 Was selection of predictors based on univariate analysis avoided? | Yes | Yes | Yes | Yes |  |
| 4.6 Were the complexities of the data (eg censoring, competing risks, sampling of controls) adequately accounted for? | Yes | Yes | Yes | Yes |  |
| 4.7 Were relevant model performance measures adequately evaluated? | Yes | Yes | Yes | Yes |  |
| 4.8 Was model overfitting and optimism factored into model performance? | Yes | Yes | Yes | Yes |  |
| 4.9 Do the predictors and their assigned weights in the final model correspond to the results of the multivariate analysis? | Yes | Yes | Yes | Yes |  |
| **Risk of bias introduced by the analysis (low/high/Unclear)** | Low | Low | Low | Low |  |
| **Step 4: General evaluation** | **-** | **-** | **-** | This article does not clearly define how the data was evaluated |  |
| **Overall assessment of risk of bias** | Low | Low | Low | Low |  |
| **General judgment of applicability** | Low | Low | Low | High |  |
|  |  |  |  |  |  |
|  |  |  |  |  |  |
| **Domain 1** | **Gomes et al. (2022)** | **González-Parra et al. (2021)** | **Martínez-Rodríguez et al. (2021)** | **Gonzalez-Parra et al. (2022)** | **Good et al. (2020)** |
| **A. Risk of bias** | **25-06-22** | **25-06-22** | **25-06-22** | **25-06-22** | **25-06-22** |
| 1.1 Were appropriate data sources used, e.g. data from cohort studies, RCTs or nested case-controls? | Yes | Unclear | Unclear | Unclear | Unclear |
| 1.2 Were all inclusions and exclusions of participants appropriate? | Yes | Unclear | Unclear | Unclear | No |
| Risk of bias introduced by participant selection (low/high/unclear) | Low | High | High | High | High |
| **B. Applicability** |  |  |  |  |  |
| Concern that included participants and setting do not match review question (low/high/unclear) | Low | Low | Low | Low | Low |
| **Domain 2** |  |  |  |  |  |
| **A. Risk of bias - predictors** |  |  |  |  |  |
| 2.1 Were predictors defined and evaluated in a similar way for all participants? | Yes | Yes | Yes | Yes | No |
| 2.2 Were predictor evaluations performed without knowing the outcome data? | Yes | Yes | Yes | Yes | Unclear |
| 2.3 Are all the predictors available at the time the model is intended to be used? | Yes | Yes | Yes | Yes | Unclear |
| Risk of bias introduced by the predictors or their assessment (low/high/unclear) | Low | Low | Low | Low | High |
| **B. Applicability** |  |  |  |  |  |
| Concern that the definition, evaluation, or timing of predictors in the model do not match the review question (low/high/unclear) | Low | Low | Low | Low | Unclear |
| **Domain 3** |  |  |  |  |  |
| **A. Risk of bias - outcome** |  |  |  |  |  |
| Was the result properly determined? | Yes | Yes | Yes | Yes | Yes |
| Was a standard or prespecified outcome definition used? | Yes | Yes | Yes | Yes | No |
| Were predictors excluded from the outcome definition? | Yes | Yes | Yes | Yes | Unclear |
| Was the outcome similarly defined and determined for all participants? | Yes | Yes | Yes | Yes | Unclear |
| Was the outcome determined without knowing the predictor information? | Yes | Yes | Yes | Yes | Unclear |
| Was the time interval between the evaluation of the predictor and the determination of the result adequate? | Yes | Yes | Yes | Yes | Yes |
| Risk of bias introduced by the result or its determination (low/high/unclear) | Low | Low | Low | Low | High |
| **B. Applicability** |  |  |  |  |  |
| If a composite outcome was used, please describe the relative frequency/distribution of each contributing outcome: | Yes | Yes | Yes | Yes | Unclear |
| Concern that the outcome, its definition, timing, or determination does not match the review question (low/high/unclear) | Low | Low | Low | Low | Unclear |
| **Domain 4** |  |  |  |  |  |
| **A. Risk of bias** |  |  |  |  |  |
| Describe the number of participants, number of candidate predictors, outcome events, and events per candidate predictor: | Yes | Yes | Yes | Yes | No |
| Describe how the model was developed (for example, with respect to modeling technique (eg, survival or logistic modeling), selection of predictors, and definition of risk groups): | Yes | Yes | Yes | Yes | Unclear |
| Describe if and how the model was validated, either internally (eg, bootstrapping, cross-validation, split random sample) or externally (eg, temporal validation, geographic validation, different setting, different type of participants): | Yes | Yes | Yes | Yes | Unclear |
| Describe the performance measures of the model, e.g. (re)calibration, discrimination, (re)classification, net profit and if adjusted for optimism: | Yes | Yes | Yes | Yes | Unclear |
| Describe the participants who were excluded from the analysis: | Yes | Yes | Yes | Yes | No |
| Describe missing data on predictors and outcomes, as well as methods used for missing data: | Yes | No | No | No | No |
| 4.1 Was there a reasonable number of participants with the result? | Yes | Yes | Yes | Yes | Yes |
| 4.2 Were continuous and categorical predictors handled correctly? | Yes | Yes | Yes | Yes | Yes |
| 4.3 Were all enrolled participants included in the analysis? | Yes | Yes | Yes | Yes | Yes |
| 4.4 Were participants with missing data treated appropriately? | Yes | No | No | No | Yes |
| 4.5 Was selection of predictors based on univariate analysis avoided? | Yes | Yes | Yes | Yes | Yes |
| 4.6 Were the complexities of the data (eg censoring, competing risks, sampling of controls) adequately accounted for? | Yes | Yes | Yes | Yes | Yes |
| 4.7 Were relevant model performance measures adequately evaluated? | Yes | Yes | Yes | Yes | Yes |
| 4.8 Was model overfitting and optimism factored into model performance? | Yes | Yes | Yes | Yes | Yes |
| 4.9 Do the predictors and their assigned weights in the final model correspond to the results of the multivariate analysis? | Yes | Yes | Yes | Yes | No |
| **Risk of bias introduced by the analysis (low/high/Unclear)** | Low | Low | Low | Low | High |
| **Step 4: General evaluation** | **-** | There is no clear information on the origin of the data | There is no clear information on the origin of the data | There is no clear information on the origin of the data | This study does not have many details required to define the model |
| **Overall assessment of risk of bias** | Low | High | High | High | High |
| **General judgment of applicability** | Low | Low | Low | Low | High |
|  |  |  |  |  |  |
|  |  |  |  |  |  |
| **Domain 1** | **Gozzi et al. (2021)** | **Gozzi et al. (2022)** | **Guerstein et al. (2020)** | **Gumel et al. (2021)** |  |
| **A. Risk of bias** | **25-06-22** | **25-06-22** | **25-06-22** | **25-06-22** |  |
| 1.1 Were appropriate data sources used, e.g. data from cohort studies, RCTs or nested case-controls? | Yes | Yes | Unclear | Yes |  |
| 1.2 Were all inclusions and exclusions of participants appropriate? | Yes | Yes | Unclear | Yes |  |
| Risk of bias introduced by participant selection (low/high/unclear) | Low | Low | High | Low |  |
| **B. Applicability** |  |  |  |  |  |
| Concern that included participants and setting do not match review question (low/high/unclear) | Low | Low | Low | Low |  |
| **Domain 2** |  |  |  |  |  |
| **A. Risk of bias - predictors** |  |  |  |  |  |
| 2.1 Were predictors defined and evaluated in a similar way for all participants? | Yes | Yes | Yes | Yes |  |
| 2.2 Were predictor evaluations performed without knowing the outcome data? | Yes | Yes | Yes | Yes |  |
| 2.3 Are all the predictors available at the time the model is intended to be used? | Yes | Yes | Yes | Yes |  |
| Risk of bias introduced by the predictors or their assessment (low/high/unclear) | Low | Low | Low | Low |  |
| **B. Applicability** |  |  |  |  |  |
| Concern that the definition, evaluation, or timing of predictors in the model do not match the review question (low/high/unclear) | Low | Low | Low | Low |  |
| **Domain 3** |  |  |  |  |  |
| **A. Risk of bias - outcome** |  |  |  |  |  |
| Was the result properly determined? | Yes | Yes | Yes | Yes |  |
| Was a standard or prespecified outcome definition used? | Yes | Yes | Yes | Yes |  |
| Were predictors excluded from the outcome definition? | Yes | Yes | Yes | Yes |  |
| Was the outcome similarly defined and determined for all participants? | Yes | Yes | Yes | Yes |  |
| Was the outcome determined without knowing the predictor information? | Yes | Yes | Yes | Yes |  |
| Was the time interval between the evaluation of the predictor and the determination of the result adequate? | Yes | Yes | Yes | Yes |  |
| Risk of bias introduced by the result or its determination (low/high/unclear) | Low | Low | Low | Low |  |
| **B. Applicability** |  |  |  |  |  |
| If a composite outcome was used, please describe the relative frequency/distribution of each contributing outcome: | Yes | Yes | Yes | Yes |  |
| Concern that the outcome, its definition, timing, or determination does not match the review question (low/high/unclear) | Low | Low | Low | Low |  |
| **Domain 4** |  |  |  |  |  |
| **A. Risk of bias** |  |  |  |  |  |
| Describe the number of participants, number of candidate predictors, outcome events, and events per candidate predictor: | Yes | Yes | Yes | Yes |  |
| Describe how the model was developed (for example, with respect to modeling technique (eg, survival or logistic modeling), selection of predictors, and definition of risk groups): | Yes | Yes | Yes | Yes |  |
| Describe if and how the model was validated, either internally (eg, bootstrapping, cross-validation, split random sample) or externally (eg, temporal validation, geographic validation, different setting, different type of participants): | Yes | Yes | Yes | Yes |  |
| Describe the performance measures of the model, e.g. (re)calibration, discrimination, (re)classification, net profit and if adjusted for optimism: | Yes | Yes | Yes | Yes |  |
| Describe the participants who were excluded from the analysis: | Yes | Yes | Yes | Yes |  |
| Describe missing data on predictors and outcomes, as well as methods used for missing data: | Yes | Yes | No | Yes |  |
| 4.1 Was there a reasonable number of participants with the result? | Yes | Yes | Yes | Yes |  |
| 4.2 Were continuous and categorical predictors handled correctly? | Yes | Yes | Yes | Yes |  |
| 4.3 Were all enrolled participants included in the analysis? | Yes | Yes | Yes | Yes |  |
| 4.4 Were participants with missing data treated appropriately? | Yes | Yes | No | Yes |  |
| 4.5 Was selection of predictors based on univariate analysis avoided? | Yes | Yes | Yes | Yes |  |
| 4.6 Were the complexities of the data (eg censoring, competing risks, sampling of controls) adequately accounted for? | Yes | Yes | Yes | Yes |  |
| 4.7 Were relevant model performance measures adequately evaluated? | Yes | Yes | Yes | Yes |  |
| 4.8 Was model overfitting and optimism factored into model performance? | Yes | Yes | Yes | Yes |  |
| 4.9 Do the predictors and their assigned weights in the final model correspond to the results of the multivariate analysis? | Yes | Yes | Yes | Yes |  |
| **Risk of bias introduced by the analysis (low/high/Unclear)** | Low | Low | Low | Low |  |
| **Step 4: General evaluation** | **-** | **-** | There is no clear information on the origin of the data | **-** |  |
| **Overall assessment of risk of bias** | Low | Low | High | Low |  |
| **General judgment of applicability** | Low | Low | Low | Low |  |
|  |  |  |  |  |  |
|  |  |  |  |  |  |
| **Domain 1** | **Gutiérrez-Jara et al. (2022)** | **Guzmán-Merino et al. (2021)** | **Hagens et al. (2021)** | **Hammouni et al. (2021)** | **Han et al. (2021)** |
| **A. Risk of bias** | **25-06-22** | **25-06-22** | **25-06-22** | **25-06-22** | **25-06-22** |
| 1.1 Were appropriate data sources used, e.g. data from cohort studies, RCTs or nested case-controls? | Unclear | Yes | Yes | Yes | Yes |
| 1.2 Were all inclusions and exclusions of participants appropriate? | Unclear | Yes | Yes | Yes | Yes |
| Risk of bias introduced by participant selection (low/high/unclear) | High | Low | Low | Low | Low |
| **B. Applicability** |  |  |  |  |  |
| Concern that included participants and setting do not match review question (low/high/unclear) | Low | Low | Low | Low | Low |
| **Domain 2** |  |  |  |  |  |
| **A. Risk of bias - predictors** |  |  |  |  |  |
| 2.1 Were predictors defined and evaluated in a similar way for all participants? | Yes | Yes | Yes | Yes | Yes |
| 2.2 Were predictor evaluations performed without knowing the outcome data? | Yes | Yes | Yes | Yes | Yes |
| 2.3 Are all the predictors available at the time the model is intended to be used? | Yes | Yes | Yes | Yes | Yes |
| Risk of bias introduced by the predictors or their assessment (low/high/unclear) | Low | Low | Low | Low | Low |
| **B. Applicability** |  |  |  |  |  |
| Concern that the definition, evaluation, or timing of predictors in the model do not match the review question (low/high/unclear) | Low | Low | Low | Low | Low |
| **Domain 3** |  |  |  |  |  |
| **A. Risk of bias - outcome** |  |  |  |  |  |
| Was the result properly determined? | Yes | Yes | Yes | Yes | Yes |
| Was a standard or prespecified outcome definition used? | Yes | Yes | Yes | Yes | Yes |
| Were predictors excluded from the outcome definition? | Yes | Yes | Yes | Yes | Yes |
| Was the outcome similarly defined and determined for all participants? | Yes | Yes | Yes | Yes | Yes |
| Was the outcome determined without knowing the predictor information? | Yes | Yes | Yes | Yes | Yes |
| Was the time interval between the evaluation of the predictor and the determination of the result adequate? | Yes | Yes | Yes | Yes | Yes |
| Risk of bias introduced by the result or its determination (low/high/unclear) | Low | Low | Low | Low | Low |
| **B. Applicability** |  |  |  |  |  |
| If a composite outcome was used, please describe the relative frequency/distribution of each contributing outcome: | Yes | Yes | Yes | Yes | Yes |
| Concern that the outcome, its definition, timing, or determination does not match the review question (low/high/unclear) | Low | Low | Low | Low | Low |
| **Domain 4** |  |  |  |  |  |
| **A. Risk of bias** |  |  |  |  |  |
| Describe the number of participants, number of candidate predictors, outcome events, and events per candidate predictor: | Yes | Yes | Yes | Yes | Yes |
| Describe how the model was developed (for example, with respect to modeling technique (eg, survival or logistic modeling), selection of predictors, and definition of risk groups): | Yes | Yes | Yes | Yes | Yes |
| Describe if and how the model was validated, either internally (eg, bootstrapping, cross-validation, split random sample) or externally (eg, temporal validation, geographic validation, different setting, different type of participants): | Yes | Yes | Yes | Yes | Yes |
| Describe the performance measures of the model, e.g. (re)calibration, discrimination, (re)classification, net profit and if adjusted for optimism: | Yes | Yes | Yes | Yes | Yes |
| Describe the participants who were excluded from the analysis: | Yes | Yes | Yes | Yes | Yes |
| Describe missing data on predictors and outcomes, as well as methods used for missing data: | Yes | Yes | Yes | Yes | Yes |
| 4.1 Was there a reasonable number of participants with the result? | Yes | Yes | Yes | Yes | Yes |
| 4.2 Were continuous and categorical predictors handled correctly? | Yes | Yes | Yes | Yes | Yes |
| 4.3 Were all enrolled participants included in the analysis? | Yes | Yes | Yes | Yes | Yes |
| 4.4 Were participants with missing data treated appropriately? | Yes | Yes | Yes | Yes | Yes |
| 4.5 Was selection of predictors based on univariate analysis avoided? | Yes | Yes | Yes | Yes | Yes |
| 4.6 Were the complexities of the data (eg censoring, competing risks, sampling of controls) adequately accounted for? | Yes | Yes | Yes | Yes | Yes |
| 4.7 Were relevant model performance measures adequately evaluated? | Yes | Yes | Yes | Yes | Yes |
| 4.8 Was model overfitting and optimism factored into model performance? | Yes | Yes | Yes | Yes | Yes |
| 4.9 Do the predictors and their assigned weights in the final model correspond to the results of the multivariate analysis? | Yes | Yes | Yes | Yes | Yes |
| **Risk of bias introduced by the analysis (low/high/Unclear)** | Low | Low | Low | Low | Low |
| **Step 4: General evaluation** | There is not enough information related to the chosen population, so there is a risk of introducing bias in the selection | - | - | **-** | **-** |
| **Overall assessment of risk of bias** | High | Low | Low | Low | Low |
| **General judgment of applicability** | Low | Low | Low | Low | Low |
|  |  |  |  |  |  |
|  |  |  |  |  |  |
| **Domain 1** | **Hanly et al. (2022)** | **Hartnertt et al. (2021)** | **Hawkes et al. (2022)** | **Hinch et al. (2021)** | **Hjorleifsson et al. (2022)** |
| **A. Risk of bias** | **25-06-22** | **25-06-22** | **25-06-22** | **25-06-22** | **25-06-22** |
| 1.1 Were appropriate data sources used, e.g. data from cohort studies, RCTs or nested case-controls? | Yes | Unclear | Yes | Yes | Unclear |
| 1.2 Were all inclusions and exclusions of participants appropriate? | Yes | No | Yes | Yes | No |
| Risk of bias introduced by participant selection (low/high/unclear) | Low | High | Low | Low | High |
| **B. Applicability** |  |  |  |  |  |
| Concern that included participants and setting do not match review question (low/high/unclear) | Low | Low | Low | Low | Low |
| **Domain 2** |  |  |  |  |  |
| **A. Risk of bias - predictors** |  |  |  |  |  |
| 2.1 Were predictors defined and evaluated in a similar way for all participants? | Yes | No | Yes | Yes | No |
| 2.2 Were predictor evaluations performed without knowing the outcome data? | Yes | Unclear | Yes | Yes | Unclear |
| 2.3 Are all the predictors available at the time the model is intended to be used? | Yes | Unclear | Yes | Yes | Unclear |
| Risk of bias introduced by the predictors or their assessment (low/high/unclear) | Low | High | Low | Low | High |
| **B. Applicability** |  |  |  |  |  |
| Concern that the definition, evaluation, or timing of predictors in the model do not match the review question (low/high/unclear) | Low | Unclear | Low | Low | Unclear |
| **Domain 3** |  |  |  |  |  |
| **A. Risk of bias - outcome** |  |  |  |  |  |
| Was the result properly determined? | Yes | Yes | Yes | Yes | Yes |
| Was a standard or prespecified outcome definition used? | Yes | No | Yes | Yes | No |
| Were predictors excluded from the outcome definition? | Yes | Unclear | Yes | Yes | Unclear |
| Was the outcome similarly defined and determined for all participants? | Yes | Unclear | Yes | Yes | Unclear |
| Was the outcome determined without knowing the predictor information? | Yes | Unclear | Yes | Yes | Unclear |
| Was the time interval between the evaluation of the predictor and the determination of the result adequate? | Yes | Yes | Yes | Yes | Yes |
| Risk of bias introduced by the result or its determination (low/high/unclear) | Low | High | Low | Low | High |
| **B. Applicability** |  |  |  |  |  |
| If a composite outcome was used, please describe the relative frequency/distribution of each contributing outcome: | Yes | Unclear | Yes | Yes | Unclear |
| Concern that the outcome, its definition, timing, or determination does not match the review question (low/high/unclear) | Low | Unclear | Low | Low | Unclear |
| **Domain 4** |  |  |  |  |  |
| **A. Risk of bias** |  |  |  |  |  |
| Describe the number of participants, number of candidate predictors, outcome events, and events per candidate predictor: | Yes | No | Yes | Yes | No |
| Describe how the model was developed (for example, with respect to modeling technique (eg, survival or logistic modeling), selection of predictors, and definition of risk groups): | Yes | Unclear | Yes | Yes | Unclear |
| Describe if and how the model was validated, either internally (eg, bootstrapping, cross-validation, split random sample) or externally (eg, temporal validation, geographic validation, different setting, different type of participants): | Yes | Unclear | Yes | Yes | Unclear |
| Describe the performance measures of the model, e.g. (re)calibration, discrimination, (re)classification, net profit and if adjusted for optimism: | Yes | Unclear | Yes | Yes | Unclear |
| Describe the participants who were excluded from the analysis: | Yes | No | Yes | Yes | No |
| Describe missing data on predictors and outcomes, as well as methods used for missing data: | Yes | No | Yes | Yes | No |
| 4.1 Was there a reasonable number of participants with the result? | Yes | Yes | Yes | Yes | Yes |
| 4.2 Were continuous and categorical predictors handled correctly? | Yes | Yes | Yes | Yes | Yes |
| 4.3 Were all enrolled participants included in the analysis? | Yes | Yes | Yes | Yes | Yes |
| 4.4 Were participants with missing data treated appropriately? | Yes | Yes | Yes | Yes | Yes |
| 4.5 Was selection of predictors based on univariate analysis avoided? | Yes | Yes | Yes | Yes | Yes |
| 4.6 Were the complexities of the data (eg censoring, competing risks, sampling of controls) adequately accounted for? | Yes | Yes | Yes | Yes | Yes |
| 4.7 Were relevant model performance measures adequately evaluated? | Yes | Yes | Yes | Yes | Yes |
| 4.8 Was model overfitting and optimism factored into model performance? | Yes | Yes | Yes | Yes | Yes |
| 4.9 Do the predictors and their assigned weights in the final model correspond to the results of the multivariate analysis? | Yes | No | Yes | Yes | No |
| **Risk of bias introduced by the analysis (low/high/Unclear)** | Low | High | Low | Low | High |
| **Step 4: General evaluation** | **-** | This study does not have many details required to define the model | - | - | This study does not have many details required to define the model |
| **Overall assessment of risk of bias** | Low | High | Low | Low | High |
| **General judgment of applicability** | Low | High | Low | Low | High |
|  |  |  |  |  |  |
|  |  |  |  |  |  |
| **Domain 1** | **Hladish et al. (2022)** | **Hoertel et al. (2021)** | **Hogan et al. (2021)** | **Holmdahl et al. (2021)** |  |
| **A. Risk of bias** | **26-06-22** | **26-06-22** | **26-06-22** | **26-06-22** |  |
| 1.1 Were appropriate data sources used, e.g. data from cohort studies, RCTs or nested case-controls? | Unclear | Unclear | Yes | Yes |  |
| 1.2 Were all inclusions and exclusions of participants appropriate? | No | No | Yes | Yes |  |
| Risk of bias introduced by participant selection (low/high/unclear) | High | High | Low | Low |  |
| **B. Applicability** |  |  |  |  |  |
| Concern that included participants and setting do not match review question (low/high/unclear) | Low | Low | Low | Low |  |
| **Domain 2** |  |  |  |  |  |
| **A. Risk of bias - predictors** |  |  |  |  |  |
| 2.1 Were predictors defined and evaluated in a similar way for all participants? | No | Yes | Yes | Yes |  |
| 2.2 Were predictor evaluations performed without knowing the outcome data? | Unclear | Yes | Yes | Yes |  |
| 2.3 Are all the predictors available at the time the model is intended to be used? | Unclear | Yes | Yes | Yes |  |
| Risk of bias introduced by the predictors or their assessment (low/high/unclear) | High | Low | Low | Low |  |
| **B. Applicability** |  |  |  |  |  |
| Concern that the definition, evaluation, or timing of predictors in the model do not match the review question (low/high/unclear) | Unclear | Low | Low | Low |  |
| **Domain 3** |  |  |  |  |  |
| **A. Risk of bias - outcome** |  |  |  |  |  |
| Was the result properly determined? | Yes | Yes | Yes | Yes |  |
| Was a standard or prespecified outcome definition used? | No | Yes | Yes | Yes |  |
| Were predictors excluded from the outcome definition? | Unclear | Yes | Yes | Yes |  |
| Was the outcome similarly defined and determined for all participants? | Unclear | Yes | Yes | Yes |  |
| Was the outcome determined without knowing the predictor information? | Unclear | Yes | Yes | Yes |  |
| Was the time interval between the evaluation of the predictor and the determination of the result adequate? | Yes | Yes | Yes | Yes |  |
| Risk of bias introduced by the result or its determination (low/high/unclear) | High | Low | Low | Low |  |
| **B. Applicability** |  |  |  |  |  |
| If a composite outcome was used, please describe the relative frequency/distribution of each contributing outcome: | Unclear | Yes | Yes | Yes |  |
| Concern that the outcome, its definition, timing, or determination does not match the review question (low/high/unclear) | Unclear | Low | Low | Low |  |
| **Domain 4** |  |  |  |  |  |
| **A. Risk of bias** |  |  |  |  |  |
| Describe the number of participants, number of candidate predictors, outcome events, and events per candidate predictor: | No | Yes | Yes | Yes |  |
| Describe how the model was developed (for example, with respect to modeling technique (eg, survival or logistic modeling), selection of predictors, and definition of risk groups): | Unclear | Yes | Yes | Yes |  |
| Describe if and how the model was validated, either internally (eg, bootstrapping, cross-validation, split random sample) or externally (eg, temporal validation, geographic validation, different setting, different type of participants): | Unclear | Yes | Yes | Yes |  |
| Describe the performance measures of the model, e.g. (re)calibration, discrimination, (re)classification, net profit and if adjusted for optimism: | Unclear | Yes | Yes | Yes |  |
| Describe the participants who were excluded from the analysis: | No | Yes | Yes | Yes |  |
| Describe missing data on predictors and outcomes, as well as methods used for missing data: | No | Yes | Yes | Yes |  |
| 4.1 Was there a reasonable number of participants with the result? | Yes | Yes | Yes | Yes |  |
| 4.2 Were continuous and categorical predictors handled correctly? | Yes | Yes | Yes | Yes |  |
| 4.3 Were all enrolled participants included in the analysis? | Yes | Yes | Yes | Yes |  |
| 4.4 Were participants with missing data treated appropriately? | Yes | Yes | Yes | Yes |  |
| 4.5 Was selection of predictors based on univariate analysis avoided? | Yes | Yes | Yes | Yes |  |
| 4.6 Were the complexities of the data (eg censoring, competing risks, sampling of controls) adequately accounted for? | Yes | Yes | Yes | Yes |  |
| 4.7 Were relevant model performance measures adequately evaluated? | Yes | Yes | Yes | Yes |  |
| 4.8 Was model overfitting and optimism factored into model performance? | Yes | Yes | Yes | Yes |  |
| 4.9 Do the predictors and their assigned weights in the final model correspond to the results of the multivariate analysis? | No | Yes | Yes | Yes |  |
| **Risk of bias introduced by the analysis (low/high/Unclear)** | High | Low | Low | Low |  |
| **Step 4: General evaluation** | This study does not have many details required to define the model | This study does not have details of the origin of the population, it only mentions that it is based in France in the general population of the country | **-** | **-** |  |
| **Overall assessment of risk of bias** | High | High | Low | Low |  |
| **General judgment of applicability** | High | Low | Low | Low |  |
|  |  |  |  |  |  |
|  |  |  |  |  |  |
| **Domain 1** | **Huang et al. (2021)** | **Hupert et al. (2021)** | **Iboi et al. (2020)** | **Jablonska et al. (2021)** | **Jayasundara et al. (2021)** |
| **A. Risk of bias** | **26-06-22** | **26-06-22** | **01-07-22** | **01-07-22** | **01-07-22** |
| 1.1 Were appropriate data sources used, e.g. data from cohort studies, RCTs or nested case-controls? | Yes | Yes | Unclear | Yes | Yes |
| 1.2 Were all inclusions and exclusions of participants appropriate? | Yes | Unclear | No | Unclear | Unclear |
| Risk of bias introduced by participant selection (low/high/unclear) | Low | Low | High | Low | Low |
| **B. Applicability** |  |  |  |  |  |
| Concern that included participants and setting do not match review question (low/high/unclear) | Low | Low | Low | Low | Low |
| **Domain 2** |  |  |  |  |  |
| **A. Risk of bias - predictors** |  |  |  |  |  |
| 2.1 Were predictors defined and evaluated in a similar way for all participants? | Yes | Yes | Yes | Yes | Yes |
| 2.2 Were predictor evaluations performed without knowing the outcome data? | Yes | Yes | Yes | Yes | Yes |
| 2.3 Are all the predictors available at the time the model is intended to be used? | Yes | Yes | Yes | Yes | Yes |
| Risk of bias introduced by the predictors or their assessment (low/high/unclear) | Low | Low | Low | Low | Low |
| **B. Applicability** |  |  |  |  |  |
| Concern that the definition, evaluation, or timing of predictors in the model do not match the review question (low/high/unclear) | Low | Low | Low | Low | Low |
| **Domain 3** |  |  |  |  |  |
| **A. Risk of bias - outcome** |  |  |  |  |  |
| Was the result properly determined? | Yes | Yes | Yes | Yes | Yes |
| Was a standard or prespecified outcome definition used? | Yes | Yes | Yes | Yes | Yes |
| Were predictors excluded from the outcome definition? | Yes | Yes | Yes | Yes | Yes |
| Was the outcome similarly defined and determined for all participants? | Yes | Yes | Yes | Yes | Yes |
| Was the outcome determined without knowing the predictor information? | Yes | Yes | Yes | Yes | Yes |
| Was the time interval between the evaluation of the predictor and the determination of the result adequate? | Yes | Yes | Yes | Yes | Yes |
| Risk of bias introduced by the result or its determination (low/high/unclear) | Low | Low | Low | Low | Low |
| **B. Applicability** |  |  |  |  |  |
| If a composite outcome was used, please describe the relative frequency/distribution of each contributing outcome: | Yes | Yes | Yes | Yes | Yes |
| Concern that the outcome, its definition, timing, or determination does not match the review question (low/high/unclear) | Low | Low | Low | Low | Low |
| **Domain 4** |  |  |  |  |  |
| **A. Risk of bias** |  |  |  |  |  |
| Describe the number of participants, number of candidate predictors, outcome events, and events per candidate predictor: | Yes | Yes | Yes | Yes | Yes |
| Describe how the model was developed (for example, with respect to modeling technique (eg, survival or logistic modeling), selection of predictors, and definition of risk groups): | Yes | Yes | Yes | Yes | Yes |
| Describe if and how the model was validated, either internally (eg, bootstrapping, cross-validation, split random sample) or externally (eg, temporal validation, geographic validation, different setting, different type of participants): | Yes | Yes | Yes | Yes | Yes |
| Describe the performance measures of the model, e.g. (re)calibration, discrimination, (re)classification, net profit and if adjusted for optimism: | Yes | Yes | Yes | Yes | Yes |
| Describe the participants who were excluded from the analysis: | Yes | Yes | Yes | Yes | Yes |
| Describe missing data on predictors and outcomes, as well as methods used for missing data: | Yes | Yes | Yes | Yes | Yes |
| 4.1 Was there a reasonable number of participants with the result? | Yes | Yes | Yes | Yes | Yes |
| 4.2 Were continuous and categorical predictors handled correctly? | Yes | Yes | Yes | Yes | Yes |
| 4.3 Were all enrolled participants included in the analysis? | Yes | Yes | Yes | Yes | Yes |
| 4.4 Were participants with missing data treated appropriately? | Yes | No | No | No | No |
| 4.5 Was selection of predictors based on univariate analysis avoided? | Yes | Yes | Yes | Yes | Yes |
| 4.6 Were the complexities of the data (eg censoring, competing risks, sampling of controls) adequately accounted for? | Yes | Yes | Yes | Yes | Yes |
| 4.7 Were relevant model performance measures adequately evaluated? | Yes | Yes | Yes | Yes | Yes |
| 4.8 Was model overfitting and optimism factored into model performance? | Yes | Yes | Yes | Yes | Yes |
| 4.9 Do the predictors and their assigned weights in the final model correspond to the results of the multivariate analysis? | Yes | Yes | Yes | Yes | Yes |
| **Risk of bias introduced by the analysis (low/high/Unclear)** | Low | Low | Low | Low | Low |
| **Step 4: General evaluation** | **-** | **-** | There are not many details of the population | **-** | **-** |
| **Overall assessment of risk of bias** | Low | Low | High | Low | Low |
| **General judgment of applicability** | Low | Low | Low | Low | Low |
|  |  |  |  |  |  |
|  |  |  |  |  |  |
| **Domain 1** | **Jentsch et al. (2021)** | **Jiménez-Rodríguez et al. (2021)** | **Kahn et al. (2022)** | **Karabay et al. (2021)** |  |
| **A. Risk of bias** | **01-07-22** | **01-07-22** | **01-07-22** | **01-07-22** |  |
| 1.1 Were appropriate data sources used, e.g. data from cohort studies, RCTs or nested case-controls? | Yes | Yes | Yes | Yes |  |
| 1.2 Were all inclusions and exclusions of participants appropriate? | Unclear | Unclear | Unclear | Unclear |  |
| Risk of bias introduced by participant selection (low/high/unclear) | Low | Low | Low | Low |  |
| **B. Applicability** |  |  |  |  |  |
| Concern that included participants and setting do not match review question (low/high/unclear) | Low | Low | Low | Low |  |
| **Domain 2** |  |  |  |  |  |
| **A. Risk of bias - predictors** |  |  |  |  |  |
| 2.1 Were predictors defined and evaluated in a similar way for all participants? | Yes | Yes | Yes | Yes |  |
| 2.2 Were predictor evaluations performed without knowing the outcome data? | Yes | Yes | Yes | Yes |  |
| 2.3 Are all the predictors available at the time the model is intended to be used? | Yes | Yes | Yes | Yes |  |
| Risk of bias introduced by the predictors or their assessment (low/high/unclear) | Low | Low | Low | Low |  |
| **B. Applicability** |  |  |  |  |  |
| Concern that the definition, evaluation, or timing of predictors in the model do not match the review question (low/high/unclear) | Low | Low | Low | Low |  |
| **Domain 3** |  |  |  |  |  |
| **A. Risk of bias - outcome** |  |  |  |  |  |
| Was the result properly determined? | Yes | Yes | Yes | Yes |  |
| Was a standard or prespecified outcome definition used? | Yes | Yes | Yes | Yes |  |
| Were predictors excluded from the outcome definition? | Yes | Yes | Yes | Yes |  |
| Was the outcome similarly defined and determined for all participants? | Yes | Yes | Yes | Yes |  |
| Was the outcome determined without knowing the predictor information? | Yes | Yes | Yes | Yes |  |
| Was the time interval between the evaluation of the predictor and the determination of the result adequate? | Yes | Yes | Yes | Yes |  |
| Risk of bias introduced by the result or its determination (low/high/unclear) | Low | Low | Low | Low |  |
| **B. Applicability** |  |  |  |  |  |
| If a composite outcome was used, please describe the relative frequency/distribution of each contributing outcome: | Yes | Yes | Yes | Yes |  |
| Concern that the outcome, its definition, timing, or determination does not match the review question (low/high/unclear) | Low | Low | Low | Low |  |
| **Domain 4** |  |  |  |  |  |
| **A. Risk of bias** |  |  |  |  |  |
| Describe the number of participants, number of candidate predictors, outcome events, and events per candidate predictor: | Yes | Yes | Yes | Yes |  |
| Describe how the model was developed (for example, with respect to modeling technique (eg, survival or logistic modeling), selection of predictors, and definition of risk groups): | Yes | Yes | Yes | Yes |  |
| Describe if and how the model was validated, either internally (eg, bootstrapping, cross-validation, split random sample) or externally (eg, temporal validation, geographic validation, different setting, different type of participants): | Yes | Yes | Yes | Yes |  |
| Describe the performance measures of the model, e.g. (re)calibration, discrimination, (re)classification, net profit and if adjusted for optimism: | Yes | Yes | Yes | Yes |  |
| Describe the participants who were excluded from the analysis: | Yes | Yes | Yes | Yes |  |
| Describe missing data on predictors and outcomes, as well as methods used for missing data: | Yes | Yes | Yes | Yes |  |
| 4.1 Was there a reasonable number of participants with the result? | Yes | Yes | Yes | Yes |  |
| 4.2 Were continuous and categorical predictors handled correctly? | Yes | Yes | Yes | Yes |  |
| 4.3 Were all enrolled participants included in the analysis? | Yes | Yes | Yes | Yes |  |
| 4.4 Were participants with missing data treated appropriately? | No | No | No | No |  |
| 4.5 Was selection of predictors based on univariate analysis avoided? | Yes | Yes | Yes | Yes |  |
| 4.6 Were the complexities of the data (eg censoring, competing risks, sampling of controls) adequately accounted for? | Yes | Yes | Yes | Yes |  |
| 4.7 Were relevant model performance measures adequately evaluated? | Yes | Yes | Yes | Yes |  |
| 4.8 Was model overfitting and optimism factored into model performance? | Yes | Yes | Yes | Yes |  |
| 4.9 Do the predictors and their assigned weights in the final model correspond to the results of the multivariate analysis? | Yes | Yes | Yes | Yes |  |
| **Risk of bias introduced by the analysis (low/high/Unclear)** | Low | Low | Low | Low |  |
| **Step 4: General evaluation** | **-** | **-** | **-** | **-** |  |
| **Overall assessment of risk of bias** | Low | Low | Low | Low |  |
| **General judgment of applicability** | Low | Low | Low | Low |  |
|  |  |  |  |  |  |
|  |  |  |  |  |  |
| **Domain 1** | **Karabay et al. (2021)** | **Kassa et al. (2021)** | **Keeling M et al. (2021)** | **Keeling M et al. (2022)** | **Kemp et al. (2021)** |
| **A. Risk of bias** | **01-07-22** | **01-07-22** | **01-07-22** | **01-07-22** | **02-07-22** |
| 1.1 Were appropriate data sources used, e.g. data from cohort studies, RCTs or nested case-controls? | Yes | Yes | Yes | Yes | Yes |
| 1.2 Were all inclusions and exclusions of participants appropriate? | Unclear | Unclear | Unclear | Unclear | Unclear |
| Risk of bias introduced by participant selection (low/high/unclear) | Low | Low | Low | Low | Low |
| **B. Applicability** |  |  |  |  |  |
| Concern that included participants and setting do not match review question (low/high/unclear) | Low | Low | Low | Low | Low |
| **Domain 2** |  |  |  |  |  |
| **A. Risk of bias - predictors** |  |  |  |  |  |
| 2.1 Were predictors defined and evaluated in a similar way for all participants? | Yes | Yes | Yes | Yes | Yes |
| 2.2 Were predictor evaluations performed without knowing the outcome data? | Yes | Yes | Yes | Yes | Yes |
| 2.3 Are all the predictors available at the time the model is intended to be used? | Yes | Yes | Yes | Yes | Yes |
| Risk of bias introduced by the predictors or their assessment (low/high/unclear) | Low | Low | Low | Low | Low |
| **B. Applicability** |  |  |  |  |  |
| Concern that the definition, evaluation, or timing of predictors in the model do not match the review question (low/high/unclear) | Low | Low | Low | Low | Low |
| **Domain 3** |  |  |  |  |  |
| **A. Risk of bias - outcome** |  |  |  |  |  |
| Was the result properly determined? | Yes | Yes | Yes | Yes | Yes |
| Was a standard or prespecified outcome definition used? | Yes | Yes | Yes | Yes | Yes |
| Were predictors excluded from the outcome definition? | Yes | Yes | Yes | Yes | Yes |
| Was the outcome similarly defined and determined for all participants? | Yes | Yes | Yes | Yes | Yes |
| Was the outcome determined without knowing the predictor information? | Yes | Yes | Yes | Yes | Yes |
| Was the time interval between the evaluation of the predictor and the determination of the result adequate? | Yes | Yes | Yes | Yes | Yes |
| Risk of bias introduced by the result or its determination (low/high/unclear) | Low | Low | Low | Low | Low |
| **B. Applicability** |  |  |  |  |  |
| If a composite outcome was used, please describe the relative frequency/distribution of each contributing outcome: | Yes | Yes | Yes | Yes | Yes |
| Concern that the outcome, its definition, timing, or determination does not match the review question (low/high/unclear) | Low | Low | Low | Low | Low |
| **Domain 4** |  |  |  |  |  |
| **A. Risk of bias** |  |  |  |  |  |
| Describe the number of participants, number of candidate predictors, outcome events, and events per candidate predictor: | Yes | Yes | Yes | Yes | Yes |
| Describe how the model was developed (for example, with respect to modeling technique (eg, survival or logistic modeling), selection of predictors, and definition of risk groups): | Yes | Yes | Yes | Yes | Yes |
| Describe if and how the model was validated, either internally (eg, bootstrapping, cross-validation, split random sample) or externally (eg, temporal validation, geographic validation, different setting, different type of participants): | Yes | Yes | Yes | Yes | Yes |
| Describe the performance measures of the model, e.g. (re)calibration, discrimination, (re)classification, net profit and if adjusted for optimism: | Yes | Yes | Yes | Yes | Yes |
| Describe the participants who were excluded from the analysis: | Yes | Yes | Yes | Yes | Yes |
| Describe missing data on predictors and outcomes, as well as methods used for missing data: | Yes | Yes | Yes | Yes | Yes |
| 4.1 Was there a reasonable number of participants with the result? | Yes | Yes | Yes | Yes | Yes |
| 4.2 Were continuous and categorical predictors handled correctly? | Yes | Yes | Yes | Yes | Yes |
| 4.3 Were all enrolled participants included in the analysis? | Yes | Yes | Yes | Yes | Yes |
| 4.4 Were participants with missing data treated appropriately? | No | No | No | No | No |
| 4.5 Was selection of predictors based on univariate analysis avoided? | Yes | Yes | Yes | Yes | Yes |
| 4.6 Were the complexities of the data (eg censoring, competing risks, sampling of controls) adequately accounted for? | Yes | Yes | Yes | Yes | Yes |
| 4.7 Were relevant model performance measures adequately evaluated? | Yes | Yes | Yes | Yes | Yes |
| 4.8 Was model overfitting and optimism factored into model performance? | Yes | Yes | Yes | Yes | Yes |
| 4.9 Do the predictors and their assigned weights in the final model correspond to the results of the multivariate analysis? | Yes | Yes | Yes | Yes | Yes |
| **Risk of bias introduced by the analysis (low/high/Unclear)** | Low | Low | Low | Low | Low |
| **Step 4: General evaluation** | **-** | **-** | **-** | **-** | **-** |
| **Overall assessment of risk of bias** | Low | Low | Low | Low | Low |
| **General judgment of applicability** | Low | Low | Low | Low | Low |
|  |  |  |  |  |  |
|  |  |  |  |  |  |
| **Domain 1** | **Kerr et al. (2021)** | **Khan et al. (2021)** | **Kim et al. (2021)** | **Kirwin et al. (2021)** | **Kitano et al. (2021)** |
| **A. Risk of bias** | **02-07-22** | **02-07-22** | **02-07-22** | **02-07-22** | **02-07-22** |
| 1.1 Were appropriate data sources used, e.g. data from cohort studies, RCTs or nested case-controls? | Yes | Yes | Yes | Yes | Yes |
| 1.2 Were all inclusions and exclusions of participants appropriate? | Yes | Yes | Yes | Yes | Yes |
| Risk of bias introduced by participant selection (low/high/unclear) | Low | Low | Low | Low | Low |
| **B. Applicability** |  |  |  |  |  |
| Concern that included participants and setting do not match review question (low/high/unclear) | Low | Low | Low | Low | Low |
| **Domain 2** |  |  |  |  |  |
| **A. Risk of bias - predictors** |  |  |  |  |  |
| 2.1 Were predictors defined and evaluated in a similar way for all participants? | Yes | Yes | Yes | Yes | Yes |
| 2.2 Were predictor evaluations performed without knowing the outcome data? | Yes | Yes | Yes | Yes | Yes |
| 2.3 Are all the predictors available at the time the model is intended to be used? | Yes | Yes | Yes | Yes | Yes |
| Risk of bias introduced by the predictors or their assessment (low/high/unclear) | Low | Low | Low | Low | Low |
| **B. Applicability** |  |  |  |  |  |
| Concern that the definition, evaluation, or timing of predictors in the model do not match the review question (low/high/unclear) | Low | Low | Low | Low | Low |
| **Domain 3** |  |  |  |  |  |
| **A. Risk of bias - outcome** |  |  |  |  |  |
| Was the result properly determined? | Yes | Yes | Yes | Yes | Yes |
| Was a standard or prespecified outcome definition used? | Yes | Yes | Yes | Yes | Yes |
| Were predictors excluded from the outcome definition? | Yes | Yes | Yes | Yes | Yes |
| Was the outcome similarly defined and determined for all participants? | Yes | Yes | Yes | Yes | Yes |
| Was the outcome determined without knowing the predictor information? | Yes | Yes | Yes | Yes | Yes |
| Was the time interval between the evaluation of the predictor and the determination of the result adequate? | Yes | Yes | Yes | Yes | Yes |
| Risk of bias introduced by the result or its determination (low/high/unclear) | Low | Low | Low | Low | Low |
| **B. Applicability** |  |  |  |  |  |
| If a composite outcome was used, please describe the relative frequency/distribution of each contributing outcome: | Yes | Yes | Yes | Yes | Yes |
| Concern that the outcome, its definition, timing, or determination does not match the review question (low/high/unclear) | Low | Low | Low | Low | Low |
| **Domain 4** |  |  |  |  |  |
| **A. Risk of bias** |  |  |  |  |  |
| Describe the number of participants, number of candidate predictors, outcome events, and events per candidate predictor: | Yes | Yes | Yes | Yes | Yes |
| Describe how the model was developed (for example, with respect to modeling technique (eg, survival or logistic modeling), selection of predictors, and definition of risk groups): | Yes | Yes | Yes | Yes | Yes |
| Describe if and how the model was validated, either internally (eg, bootstrapping, cross-validation, split random sample) or externally (eg, temporal validation, geographic validation, different setting, different type of participants): | Yes | Yes | Yes | Yes | Yes |
| Describe the performance measures of the model, e.g. (re)calibration, discrimination, (re)classification, net profit and if adjusted for optimism: | Yes | Yes | Yes | Yes | Yes |
| Describe the participants who were excluded from the analysis: | Yes | Yes | Yes | Yes | Yes |
| Describe missing data on predictors and outcomes, as well as methods used for missing data: | Yes | Yes | Yes | Yes | Yes |
| 4.1 Was there a reasonable number of participants with the result? | Yes | Yes | Yes | Yes | Yes |
| 4.2 Were continuous and categorical predictors handled correctly? | Yes | Yes | Yes | Yes | Yes |
| 4.3 Were all enrolled participants included in the analysis? | Yes | Yes | Yes | Yes | Yes |
| 4.4 Were participants with missing data treated appropriately? | No | No | No | No | No |
| 4.5 Was selection of predictors based on univariate analysis avoided? | Yes | Yes | Yes | Yes | Yes |
| 4.6 Were the complexities of the data (eg censoring, competing risks, sampling of controls) adequately accounted for? | Yes | Yes | Yes | Yes | Yes |
| 4.7 Were relevant model performance measures adequately evaluated? | Yes | Yes | Yes | Yes | Yes |
| 4.8 Was model overfitting and optimism factored into model performance? | Yes | Yes | Yes | Yes | Yes |
| 4.9 Do the predictors and their assigned weights in the final model correspond to the results of the multivariate analysis? | Yes | Yes | Yes | Yes | Yes |
| **Risk of bias introduced by the analysis (low/high/Unclear)** | Low | Low | Low | Low | Low |
| **Step 4: General evaluation** |  |  |  |  |  |
| **Overall assessment of risk of bias** | Low | Low | Low | Low | Low |
| **General judgment of applicability** | Low | Low | Low | Low | Low |
|  |  |  |  |  |  |
|  |  |  |  |  |  |
| **Domain 1** | **Ko et al. (2021)** | **Ko et al. (2022)** | **Kou et al. (2021)** | **Kraay et al. (2021)** | **Lasser et al. (2022)** |
| **A. Risk of bias** | **02-07-22** | **02-07-22** | **02-07-22** | **02-07-22** | **02-07-22** |
| 1.1 Were appropriate data sources used, e.g. data from cohort studies, RCTs or nested case-controls? | Yes | Unclear | Yes | Unclear | Yes |
| 1.2 Were all inclusions and exclusions of participants appropriate? | Yes | Unclear | Unclear | Unclear | Yes |
| Risk of bias introduced by participant selection (low/high/unclear) | Low | High | Low | High | Low |
| **B. Applicability** |  |  |  |  |  |
| Concern that included participants and setting do not match review question (low/high/unclear) | Low | Low | Low | Low | Low |
| **Domain 2** |  |  |  |  |  |
| **A. Risk of bias - predictors** |  |  |  |  |  |
| 2.1 Were predictors defined and evaluated in a similar way for all participants? | Yes | Yes | Yes | Yes | Yes |
| 2.2 Were predictor evaluations performed without knowing the outcome data? | Yes | Yes | Yes | Yes | Yes |
| 2.3 Are all the predictors available at the time the model is intended to be used? | Yes | Yes | Yes | Yes | Yes |
| Risk of bias introduced by the predictors or their assessment (low/high/unclear) | Low | Low | Low | Low | Low |
| **B. Applicability** |  |  |  |  |  |
| Concern that the definition, evaluation, or timing of predictors in the model do not match the review question (low/high/unclear) | Low | Low | Low | Low | Low |
| **Domain 3** |  |  |  |  |  |
| **A. Risk of bias - outcome** |  |  |  |  |  |
| Was the result properly determined? | Yes | Yes | Yes | Yes | Yes |
| Was a standard or prespecified outcome definition used? | Yes | Yes | Yes | Yes | Yes |
| Were predictors excluded from the outcome definition? | Yes | Yes | Yes | Yes | Yes |
| Was the outcome similarly defined and determined for all participants? | Yes | Yes | Yes | Yes | Yes |
| Was the outcome determined without knowing the predictor information? | Yes | Yes | Yes | Yes | Yes |
| Was the time interval between the evaluation of the predictor and the determination of the result adequate? | Yes | Yes | Yes | Yes | Yes |
| Risk of bias introduced by the result or its determination (low/high/unclear) | Low | Low | Low | Low | Low |
| **B. Applicability** |  |  |  |  |  |
| If a composite outcome was used, please describe the relative frequency/distribution of each contributing outcome: | Yes | Yes | Yes | Yes | NA |
| Concern that the outcome, its definition, timing, or determination does not match the review question (low/high/unclear) | Low | Low | Low | Low | Low |
| **Domain 4** |  |  |  |  |  |
| **A. Risk of bias** |  |  |  |  |  |
| Describe the number of participants, number of candidate predictors, outcome events, and events per candidate predictor: | Yes | Yes | Yes | Yes | Yes |
| Describe how the model was developed (for example, with respect to modeling technique (eg, survival or logistic modeling), selection of predictors, and definition of risk groups): | Yes | Yes | Yes | Yes | Yes |
| Describe if and how the model was validated, either internally (eg, bootstrapping, cross-validation, split random sample) or externally (eg, temporal validation, geographic validation, different setting, different type of participants): | Yes | Yes | Yes | Yes | Yes |
| Describe the performance measures of the model, e.g. (re)calibration, discrimination, (re)classification, net profit and if adjusted for optimism: | Yes | Yes | Yes | Yes | Yes |
| Describe the participants who were excluded from the analysis: | Yes | Yes | Yes | Yes | Yes |
| Describe missing data on predictors and outcomes, as well as methods used for missing data: | Yes | Yes | Yes | Yes | No |
| 4.1 Was there a reasonable number of participants with the result? | Yes | Yes | Yes | Yes | Yes |
| 4.2 Were continuous and categorical predictors handled correctly? | Yes | Yes | Yes | Yes | Yes |
| 4.3 Were all enrolled participants included in the analysis? | Yes | Yes | Yes | Yes | Yes |
| 4.4 Were participants with missing data treated appropriately? | No | No | No | No | Yes |
| 4.5 Was selection of predictors based on univariate analysis avoided? | Yes | Yes | Yes | Yes | Yes |
| 4.6 Were the complexities of the data (eg censoring, competing risks, sampling of controls) adequately accounted for? | Yes | Yes | Yes | Yes | Yes |
| 4.7 Were relevant model performance measures adequately evaluated? | Yes | Yes | Yes | Yes | Yes |
| 4.8 Was model overfitting and optimism factored into model performance? | Yes | Yes | Yes | Yes | Yes |
| 4.9 Do the predictors and their assigned weights in the final model correspond to the results of the multivariate analysis? | Yes | Yes | Yes | Yes | Yes |
| **Risk of bias introduced by the analysis (low/high/Unclear)** | Low | Low | Low | Low | Low |
| **Step 4: General evaluation** | **-** | There are no methodological data, although the selection criteria are not explicitly defined | - | There are no methodological data, although the selection criteria are not explicitly defined | **-** |
| **Overall assessment of risk of bias** | Low | High | Low | High | Low |
| **General judgment of applicability** | Low | Low | Low | Low | Low |
|  |  |  |  |  |  |
|  |  |  |  |  |  |
| **Domain 1** | **Lau et al. (2021)** | **Layton et al. (2022)** | **Lee et al. (2021)** | **Lemaitre et al. (2021)** | **Leung et al. (2021)** |
| **A. Risk of bias** | **02-07-22** | **02-07-22** | **02-07-22** | **02-07-22** | **02-07-22** |
| 1.1 Were appropriate data sources used, e.g. data from cohort studies, RCTs or nested case-controls? | Yes | Unclear | Yes | Yes | Unclear |
| 1.2 Were all inclusions and exclusions of participants appropriate? | Unclear | Unclear | Unclear | Unclear | Unclear |
| Risk of bias introduced by participant selection (low/high/unclear) | Low | High | Low | Low | High |
| **B. Applicability** |  |  |  |  |  |
| Concern that included participants and setting do not match review question (low/high/unclear) | Low | Low | Low | Low | Low |
| **Domain 2** |  |  |  |  |  |
| **A. Risk of bias - predictors** |  |  |  |  |  |
| 2.1 Were predictors defined and evaluated in a similar way for all participants? | Yes | Yes | Yes | Yes | Yes |
| 2.2 Were predictor evaluations performed without knowing the outcome data? | Yes | Yes | Yes | Yes | Yes |
| 2.3 Are all the predictors available at the time the model is intended to be used? | Yes | Yes | Yes | Yes | Yes |
| Risk of bias introduced by the predictors or their assessment (low/high/unclear) | Low | Low | Low | Low | Low |
| **B. Applicability** |  |  |  |  |  |
| Concern that the definition, evaluation, or timing of predictors in the model do not match the review question (low/high/unclear) | Low | Low | Low | Low | Low |
| **Domain 3** |  |  |  |  |  |
| **A. Risk of bias - outcome** |  |  |  |  |  |
| Was the result properly determined? | Yes | Yes | Yes | Yes | Yes |
| Was a standard or prespecified outcome definition used? | Yes | Yes | Yes | Yes | Yes |
| Were predictors excluded from the outcome definition? | Yes | Yes | Yes | Yes | Yes |
| Was the outcome similarly defined and determined for all participants? | Yes | Yes | Yes | Yes | Yes |
| Was the outcome determined without knowing the predictor information? | Yes | Yes | Yes | Yes | Yes |
| Was the time interval between the evaluation of the predictor and the determination of the result adequate? | Yes | Yes | Yes | Yes | Yes |
| Risk of bias introduced by the result or its determination (low/high/unclear) | Low | Low | Low | Low | Low |
| **B. Applicability** |  |  |  |  |  |
| If a composite outcome was used, please describe the relative frequency/distribution of each contributing outcome: | Yes | Yes | Yes | Yes | Yes |
| Concern that the outcome, its definition, timing, or determination does not match the review question (low/high/unclear) | Low | Low | Low | Low | Low |
| **Domain 4** |  |  |  |  |  |
| **A. Risk of bias** |  |  |  |  |  |
| Describe the number of participants, number of candidate predictors, outcome events, and events per candidate predictor: | Yes | Yes | Yes | Yes | Yes |
| Describe how the model was developed (for example, with respect to modeling technique (eg, survival or logistic modeling), selection of predictors, and definition of risk groups): | Yes | Yes | Yes | Yes | Yes |
| Describe if and how the model was validated, either internally (eg, bootstrapping, cross-validation, split random sample) or externally (eg, temporal validation, geographic validation, different setting, different type of participants): | Yes | Yes | Yes | Yes | Yes |
| Describe the performance measures of the model, e.g. (re)calibration, discrimination, (re)classification, net profit and if adjusted for optimism: | Yes | Yes | Yes | Yes | Yes |
| Describe the participants who were excluded from the analysis: | Yes | Yes | Yes | Yes | Yes |
| Describe missing data on predictors and outcomes, as well as methods used for missing data: | Yes | Yes | Yes | Yes | Yes |
| 4.1 Was there a reasonable number of participants with the result? | Yes | Yes | Yes | Yes | Yes |
| 4.2 Were continuous and categorical predictors handled correctly? | Yes | Yes | Yes | Yes | Yes |
| 4.3 Were all enrolled participants included in the analysis? | Yes | Yes | Yes | Yes | Yes |
| 4.4 Were participants with missing data treated appropriately? | No | No | No | No | No |
| 4.5 Was selection of predictors based on univariate analysis avoided? | Yes | Yes | Yes | Yes | Yes |
| 4.6 Were the complexities of the data (eg censoring, competing risks, sampling of controls) adequately accounted for? | Yes | Yes | Yes | Yes | Yes |
| 4.7 Were relevant model performance measures adequately evaluated? | Yes | Yes | Yes | Yes | Yes |
| 4.8 Was model overfitting and optimism factored into model performance? | Yes | Yes | Yes | Yes | Yes |
| 4.9 Do the predictors and their assigned weights in the final model correspond to the results of the multivariate analysis? | Yes | Yes | Yes | Yes | Yes |
| **Risk of bias introduced by the analysis (low/high/Unclear)** | Low | Low | Low | Low | Low |
| **Step 4: General evaluation** | **-** | There are no methodological data, although the selection criteria are not explicitly defined | - | - | There are no methodological data, although the selection criteria are not explicitly defined |
| **Overall assessment of risk of bias** | Low | High | Low | Low | High |
| **General judgment of applicability** | Low | Low | Low | Low | Low |
|  |  |  |  |  |  |
|  |  |  |  |  |  |
| **Domain 1** | **Li et al. (2021) (1)** | **Li et al. (2021) (2)** | **Li et al. (2021) (3)** | **Li et al. (2021) (4)** | **Li et al. (2021) (5)** |
| **A. Risk of bias** | **02-07-22** | **02-07-22** | **02-07-22** | **02-07-22** | **02-07-22** |
| 1.1 Were appropriate data sources used, e.g. data from cohort studies, RCTs or nested case-controls? | Unclear | Unclear | Yes | Yes | Yes |
| 1.2 Were all inclusions and exclusions of participants appropriate? | Unclear | Unclear | Unclear | Unclear | Unclear |
| Risk of bias introduced by participant selection (low/high/unclear) | High | High | Low | Low | Low |
| **B. Applicability** |  |  |  |  |  |
| Concern that included participants and setting do not match review question (low/high/unclear) | Low | Low | Low | Low | Low |
| **Domain 2** |  |  |  |  |  |
| **A. Risk of bias - predictors** |  |  |  |  |  |
| 2.1 Were predictors defined and evaluated in a similar way for all participants? | Yes | Yes | Yes | Yes | Yes |
| 2.2 Were predictor evaluations performed without knowing the outcome data? | Yes | Yes | Yes | Yes | Yes |
| 2.3 Are all the predictors available at the time the model is intended to be used? | Yes | Yes | Yes | Yes | Yes |
| Risk of bias introduced by the predictors or their assessment (low/high/unclear) | Low | Low | Low | Low | Low |
| **B. Applicability** |  |  |  |  |  |
| Concern that the definition, evaluation, or timing of predictors in the model do not match the review question (low/high/unclear) | Low | Low | Low | Low | Low |
| **Domain 3** |  |  |  |  |  |
| **A. Risk of bias - outcome** |  |  |  |  |  |
| Was the result properly determined? | Yes | Yes | Yes | Yes | Yes |
| Was a standard or prespecified outcome definition used? | Yes | Yes | Yes | Yes | Yes |
| Were predictors excluded from the outcome definition? | Yes | Yes | Yes | Yes | Yes |
| Was the outcome similarly defined and determined for all participants? | Yes | Yes | Yes | Yes | Yes |
| Was the outcome determined without knowing the predictor information? | Yes | Yes | Yes | Yes | Yes |
| Was the time interval between the evaluation of the predictor and the determination of the result adequate? | Yes | Yes | Yes | Yes | Yes |
| Risk of bias introduced by the result or its determination (low/high/unclear) | Low | Low | Low | Low | Low |
| **B. Applicability** |  |  |  |  |  |
| If a composite outcome was used, please describe the relative frequency/distribution of each contributing outcome: | Yes | Yes | Yes | Yes | Yes |
| Concern that the outcome, its definition, timing, or determination does not match the review question (low/high/unclear) | Low | Low | Low | Low | Low |
| **Domain 4** |  |  |  |  |  |
| **A. Risk of bias** |  |  |  |  |  |
| Describe the number of participants, number of candidate predictors, outcome events, and events per candidate predictor: | Yes | Yes | Yes | Yes | Yes |
| Describe how the model was developed (for example, with respect to modeling technique (eg, survival or logistic modeling), selection of predictors, and definition of risk groups): | Yes | Yes | Yes | Yes | Yes |
| Describe if and how the model was validated, either internally (eg, bootstrapping, cross-validation, split random sample) or externally (eg, temporal validation, geographic validation, different setting, different type of participants): | Yes | Yes | Yes | Yes | Yes |
| Describe the performance measures of the model, e.g. (re)calibration, discrimination, (re)classification, net profit and if adjusted for optimism: | Yes | Yes | Yes | Yes | Yes |
| Describe the participants who were excluded from the analysis: | Yes | Yes | Yes | Yes | Yes |
| Describe missing data on predictors and outcomes, as well as methods used for missing data: | Yes | Yes | Yes | Yes | Yes |
| 4.1 Was there a reasonable number of participants with the result? | Yes | Yes | Yes | Yes | Yes |
| 4.2 Were continuous and categorical predictors handled correctly? | Yes | Yes | Yes | Yes | Yes |
| 4.3 Were all enrolled participants included in the analysis? | Yes | Yes | Yes | Yes | Yes |
| 4.4 Were participants with missing data treated appropriately? | No | No | No | No | No |
| 4.5 Was selection of predictors based on univariate analysis avoided? | Yes | Yes | Yes | Yes | Yes |
| 4.6 Were the complexities of the data (eg censoring, competing risks, sampling of controls) adequately accounted for? | Yes | Yes | Yes | Yes | Yes |
| 4.7 Were relevant model performance measures adequately evaluated? | Yes | Yes | Yes | Yes | Yes |
| 4.8 Was model overfitting and optimism factored into model performance? | Yes | Yes | Yes | Yes | Yes |
| 4.9 Do the predictors and their assigned weights in the final model correspond to the results of the multivariate analysis? | Yes | Yes | Yes | Yes | Yes |
| **Risk of bias introduced by the analysis (low/high/Unclear)** | Low | Low | Low | Low | Low |
| **Step 4: General evaluation** | There is not enough information related to the chosen population, so there is a risk of introducing bias in the selection | There are no methodological data, although the selection criteria are not explicitly defined | **-** | **-** | **-** |
| **Overall assessment of risk of bias** | High | High | Low | Low | Low |
| **General judgment of applicability** | Low | Low | Low | Low | Low |
|  |  |  |  |  |  |
|  |  |  |  |  |  |
| **Domain 1** | **Li et al. (2021) (6)** | **Li et al. (2022) (7)** | **Li et al. (2021) (8)** | **Lin et al. (2022) (1)** | **Lin et al. (2022) (2)** |
| **A. Risk of bias** | **02-07-22** | **02-07-22** | **02-07-22** | **08-07-22** | **08-07-22** |
| 1.1 Were appropriate data sources used, e.g. data from cohort studies, RCTs or nested case-controls? | Yes | Yes | Yes | Yes | Yes |
| 1.2 Were all inclusions and exclusions of participants appropriate? | Unclear | Unclear | Unclear | Unclear | Unclear |
| Risk of bias introduced by participant selection (low/high/unclear) | Low | Low | Low | Low | Low |
| **B. Applicability** |  |  |  |  |  |
| Concern that included participants and setting do not match review question (low/high/unclear) | Low | Low | Low | Low | Low |
| **Domain 2** |  |  |  |  |  |
| **A. Risk of bias - predictors** |  |  |  |  |  |
| 2.1 Were predictors defined and evaluated in a similar way for all participants? | Yes | Yes | Yes | Yes | Yes |
| 2.2 Were predictor evaluations performed without knowing the outcome data? | Yes | Yes | Yes | Yes | Yes |
| 2.3 Are all the predictors available at the time the model is intended to be used? | Yes | Yes | Yes | Yes | Yes |
| Risk of bias introduced by the predictors or their assessment (low/high/unclear) | Low | Low | Low | Low | Low |
| **B. Applicability** |  |  |  |  |  |
| Concern that the definition, evaluation, or timing of predictors in the model do not match the review question (low/high/unclear) | Low | Low | Low | Low | Low |
| **Domain 3** |  |  |  |  |  |
| **A. Risk of bias - outcome** |  |  |  |  |  |
| Was the result properly determined? | Yes | Yes | Yes | Yes | Yes |
| Was a standard or prespecified outcome definition used? | Yes | Yes | Yes | Yes | Yes |
| Were predictors excluded from the outcome definition? | Yes | Yes | Yes | Yes | Yes |
| Was the outcome similarly defined and determined for all participants? | Yes | Yes | Yes | Yes | Yes |
| Was the outcome determined without knowing the predictor information? | Yes | Yes | Yes | Yes | Yes |
| Was the time interval between the evaluation of the predictor and the determination of the result adequate? | Yes | Yes | Yes | Yes | Yes |
| Risk of bias introduced by the result or its determination (low/high/unclear) | Low | Low | Low | Low | Low |
| **B. Applicability** |  |  |  |  |  |
| If a composite outcome was used, please describe the relative frequency/distribution of each contributing outcome: | Yes | Yes | Yes | Yes | Yes |
| Concern that the outcome, its definition, timing, or determination does not match the review question (low/high/unclear) | Low | Low | Low | Low | Low |
| **Domain 4** |  |  |  |  |  |
| **A. Risk of bias** |  |  |  |  |  |
| Describe the number of participants, number of candidate predictors, outcome events, and events per candidate predictor: | Yes | Yes | Yes | Yes | Yes |
| Describe how the model was developed (for example, with respect to modeling technique (eg, survival or logistic modeling), selection of predictors, and definition of risk groups): | Yes | Yes | Yes | Yes | Yes |
| Describe if and how the model was validated, either internally (eg, bootstrapping, cross-validation, split random sample) or externally (eg, temporal validation, geographic validation, different setting, different type of participants): | Yes | Yes | Yes | Yes | Yes |
| Describe the performance measures of the model, e.g. (re)calibration, discrimination, (re)classification, net profit and if adjusted for optimism: | Yes | Yes | Yes | Yes | Yes |
| Describe the participants who were excluded from the analysis: | Yes | Yes | Yes | Yes | Yes |
| Describe missing data on predictors and outcomes, as well as methods used for missing data: | Yes | Yes | Yes | Yes | Yes |
| 4.1 Was there a reasonable number of participants with the result? | Yes | Yes | Yes | Yes | Yes |
| 4.2 Were continuous and categorical predictors handled correctly? | Yes | Yes | Yes | Yes | Yes |
| 4.3 Were all enrolled participants included in the analysis? | Yes | Yes | Yes | Yes | Yes |
| 4.4 Were participants with missing data treated appropriately? | No | No | No | No | No |
| 4.5 Was selection of predictors based on univariate analysis avoided? | Yes | Yes | Yes | Yes | Yes |
| 4.6 Were the complexities of the data (eg censoring, competing risks, sampling of controls) adequately accounted for? | Yes | Yes | Yes | Yes | Yes |
| 4.7 Were relevant model performance measures adequately evaluated? | Yes | Yes | Yes | Yes | Yes |
| 4.8 Was model overfitting and optimism factored into model performance? | Yes | Yes | Yes | Yes | Yes |
| 4.9 Do the predictors and their assigned weights in the final model correspond to the results of the multivariate analysis? | Yes | Yes | Yes | Yes | Yes |
| **Risk of bias introduced by the analysis (low/high/Unclear)** | Low | Low | Low | Low | Low |
| **Step 4: General evaluation** |  |  |  |  |  |
| **Overall assessment of risk of bias** | Low | Low | Low | Low | Low |
| **General judgment of applicability** | Low | Low | Low | Low | Low |
|  |  |  |  |  |  |
|  |  |  |  |  |  |
| **Domain 1** | **Liu et al. (2022) (2)** | **Liu et al. (2022) (3)** | **Liu et al. (2022) (4)** | **Luo et al. (2021)** |  |
| **A. Risk of bias** | **08-07-22** | **08-07-22** | **08-07-22** | **08-07-22** |  |
| 1.1 Were appropriate data sources used, e.g. data from cohort studies, RCTs or nested case-controls? | Yes | Yes | Yes | Unclear |  |
| 1.2 Were all inclusions and exclusions of participants appropriate? | Unclear | Unclear | Unclear | No |  |
| Risk of bias introduced by participant selection (low/high/unclear) | Low | Low | Low | High |  |
| **B. Applicability** |  |  |  |  |  |
| Concern that included participants and setting do not match review question (low/high/unclear) | Low | Low | Low | Low |  |
| **Domain 2** |  |  |  |  |  |
| **A. Risk of bias - predictors** |  |  |  |  |  |
| 2.1 Were predictors defined and evaluated in a similar way for all participants? | Yes | Yes | Yes | No |  |
| 2.2 Were predictor evaluations performed without knowing the outcome data? | Yes | Yes | Yes | Unclear |  |
| 2.3 Are all the predictors available at the time the model is intended to be used? | Yes | Yes | Yes | Unclear |  |
| Risk of bias introduced by the predictors or their assessment (low/high/unclear) | Low | Low | Low | High |  |
| **B. Applicability** |  |  |  |  |  |
| Concern that the definition, evaluation, or timing of predictors in the model do not match the review question (low/high/unclear) | Low | Low | Low | Unclear |  |
| **Domain 3** |  |  |  |  |  |
| **A. Risk of bias - outcome** |  |  |  |  |  |
| Was the result properly determined? | Yes | Yes | Yes | Yes |  |
| Was a standard or prespecified outcome definition used? | Yes | Yes | Yes | No |  |
| Were predictors excluded from the outcome definition? | Yes | Yes | Yes | Unclear |  |
| Was the outcome similarly defined and determined for all participants? | Yes | Yes | Yes | Unclear |  |
| Was the outcome determined without knowing the predictor information? | Yes | Yes | Yes | Unclear |  |
| Was the time interval between the evaluation of the predictor and the determination of the result adequate? | Yes | Yes | Yes | Yes |  |
| Risk of bias introduced by the result or its determination (low/high/unclear) | Low | Low | Low | High |  |
| **B. Applicability** |  |  |  |  |  |
| If a composite outcome was used, please describe the relative frequency/distribution of each contributing outcome: | Yes | Yes | Yes | Unclear |  |
| Concern that the outcome, its definition, timing, or determination does not match the review question (low/high/unclear) | Low | Low | Low | Unclear |  |
| **Domain 4** |  |  |  |  |  |
| **A. Risk of bias** |  |  |  |  |  |
| Describe the number of participants, number of candidate predictors, outcome events, and events per candidate predictor: | Yes | Yes | Yes | No |  |
| Describe how the model was developed (for example, with respect to modeling technique (eg, survival or logistic modeling), selection of predictors, and definition of risk groups): | Yes | Yes | Yes | Unclear |  |
| Describe if and how the model was validated, either internally (eg, bootstrapping, cross-validation, split random sample) or externally (eg, temporal validation, geographic validation, different setting, different type of participants): | Yes | Yes | Yes | Unclear |  |
| Describe the performance measures of the model, e.g. (re)calibration, discrimination, (re)classification, net profit and if adjusted for optimism: | Yes | Yes | Yes | Unclear |  |
| Describe the participants who were excluded from the analysis: | Yes | Yes | Yes | No |  |
| Describe missing data on predictors and outcomes, as well as methods used for missing data: | Yes | Yes | Yes | No |  |
| 4.1 Was there a reasonable number of participants with the result? | Yes | Yes | Yes | Yes |  |
| 4.2 Were continuous and categorical predictors handled correctly? | Yes | Yes | Yes | Yes |  |
| 4.3 Were all enrolled participants included in the analysis? | Yes | Yes | Yes | Yes |  |
| 4.4 Were participants with missing data treated appropriately? | No | No | No | No |  |
| 4.5 Was selection of predictors based on univariate analysis avoided? | Yes | Yes | Yes | Yes |  |
| 4.6 Were the complexities of the data (eg censoring, competing risks, sampling of controls) adequately accounted for? | Yes | Yes | Yes | Yes |  |
| 4.7 Were relevant model performance measures adequately evaluated? | Yes | Yes | Yes | Yes |  |
| 4.8 Was model overfitting and optimism factored into model performance? | Yes | Yes | Yes | Yes |  |
| 4.9 Do the predictors and their assigned weights in the final model correspond to the results of the multivariate analysis? | Yes | Yes | Yes | No |  |
| **Risk of bias introduced by the analysis (low/high/Unclear)** | Low | Low | Low | High |  |
| **Step 4: General evaluation** | **-** | **-** | - | This study does not have many details required to define the model |  |
| **Overall assessment of risk of bias** | Low | Low | Low | High |  |
| **General judgment of applicability** | Low | Low | Low | High |  |
|  |  |  |  |  |  |
|  |  |  |  |  |  |
| **Domain 1** | **Machado et al. (2022)** | **Maier et al. (2021)** | **Mairanowski et al. (2021) (1)** | **Mairanowski et al. (2021) (2)** | **Majumder et al. (2022)** |
| **A. Risk of bias** | **08-07-22** | **08-07-22** | **08-07-22** | **08-07-22** | **08-07-22** |
| 1.1 Were appropriate data sources used, e.g. data from cohort studies, RCTs or nested case-controls? | Yes | Yes | Unclear | Unclear | Unclear |
| 1.2 Were all inclusions and exclusions of participants appropriate? | Unclear | Unclear | No | No | No |
| Risk of bias introduced by participant selection (low/high/unclear) | Low | Low | High | High | High |
| **B. Applicability** |  |  |  |  |  |
| Concern that included participants and setting do not match review question (low/high/unclear) | Low | Low | Low | Low | Low |
| **Domain 2** |  |  |  |  |  |
| **A. Risk of bias - predictors** |  |  |  |  |  |
| 2.1 Were predictors defined and evaluated in a similar way for all participants? | Yes | Yes | No | No | No |
| 2.2 Were predictor evaluations performed without knowing the outcome data? | Yes | Yes | Unclear | Unclear | Unclear |
| 2.3 Are all the predictors available at the time the model is intended to be used? | Yes | Yes | Unclear | Unclear | Unclear |
| Risk of bias introduced by the predictors or their assessment (low/high/unclear) | Low | Low | High | High | High |
| **B. Applicability** |  |  |  |  |  |
| Concern that the definition, evaluation, or timing of predictors in the model do not match the review question (low/high/unclear) | Low | Low | Unclear | Unclear | Unclear |
| **Domain 3** |  |  |  |  |  |
| **A. Risk of bias - outcome** |  |  |  |  |  |
| Was the result properly determined? | Yes | Yes | Yes | Yes | Yes |
| Was a standard or prespecified outcome definition used? | Yes | Yes | No | No | No |
| Were predictors excluded from the outcome definition? | Yes | Yes | Unclear | Unclear | Unclear |
| Was the outcome similarly defined and determined for all participants? | Yes | Yes | Unclear | Unclear | Unclear |
| Was the outcome determined without knowing the predictor information? | Yes | Yes | Unclear | Unclear | Unclear |
| Was the time interval between the evaluation of the predictor and the determination of the result adequate? | Yes | Yes | Yes | Yes | Yes |
| Risk of bias introduced by the result or its determination (low/high/unclear) | Low | Low | High | High | High |
| **B. Applicability** |  |  |  |  |  |
| If a composite outcome was used, please describe the relative frequency/distribution of each contributing outcome: | Yes | Yes | Unclear | Unclear | Unclear |
| Concern that the outcome, its definition, timing, or determination does not match the review question (low/high/unclear) | Low | Low | Unclear | Unclear | Unclear |
| **Domain 4** |  |  |  |  |  |
| **A. Risk of bias** |  |  |  |  |  |
| Describe the number of participants, number of candidate predictors, outcome events, and events per candidate predictor: | Yes | Yes | No | No | No |
| Describe how the model was developed (for example, with respect to modeling technique (eg, survival or logistic modeling), selection of predictors, and definition of risk groups): | Yes | Yes | Unclear | Unclear | Unclear |
| Describe if and how the model was validated, either internally (eg, bootstrapping, cross-validation, split random sample) or externally (eg, temporal validation, geographic validation, different setting, different type of participants): | Yes | Yes | Unclear | Unclear | Unclear |
| Describe the performance measures of the model, e.g. (re)calibration, discrimination, (re)classification, net profit and if adjusted for optimism: | Yes | Yes | Unclear | Unclear | Unclear |
| Describe the participants who were excluded from the analysis: | Yes | Yes | No | No | No |
| Describe missing data on predictors and outcomes, as well as methods used for missing data: | Yes | Yes | No | No | No |
| 4.1 Was there a reasonable number of participants with the result? | Yes | Yes | Yes | Yes | Yes |
| 4.2 Were continuous and categorical predictors handled correctly? | Yes | Yes | Yes | Yes | Yes |
| 4.3 Were all enrolled participants included in the analysis? | Yes | Yes | Yes | Yes | Yes |
| 4.4 Were participants with missing data treated appropriately? | No | No | Yes | Yes | Yes |
| 4.5 Was selection of predictors based on univariate analysis avoided? | Yes | Yes | Yes | Yes | Yes |
| 4.6 Were the complexities of the data (eg censoring, competing risks, sampling of controls) adequately accounted for? | Yes | Yes | Yes | Yes | Yes |
| 4.7 Were relevant model performance measures adequately evaluated? | Yes | Yes | Yes | Yes | Yes |
| 4.8 Was model overfitting and optimism factored into model performance? | Yes | Yes | Yes | Yes | Yes |
| 4.9 Do the predictors and their assigned weights in the final model correspond to the results of the multivariate analysis? | Yes | Yes | No | No | No |
| **Risk of bias introduced by the analysis (low/high/Unclear)** | Low | Low | High | High | High |
| **Step 4: General evaluation** | **-** | - | This study does not have many details required to define the model | This study does not have many details required to define the model | This study does not have many details required to define the model |
| **Overall assessment of risk of bias** | Low | Low | High | High | High |
| **General judgment of applicability** | Low | Low | High | High | High |
|  |  |  |  |  |  |
|  |  |  |  |  |  |
| **Domain 1** | **Makhoul et al. (2020)** | **Makhoul et al. (2021)** | **Mallela et al. (2022)** | **Mandal et al. (2021) (1)** | **Mandal et al. (2021) (2)** |
| **A. Risk of bias** | **08-07-22** | **08-07-22** | **08-07-22** | **08-07-22** | **08-07-22** |
| 1.1 Were appropriate data sources used, e.g. data from cohort studies, RCTs or nested case-controls? | Yes | Yes | Yes | Yes | Yes |
| 1.2 Were all inclusions and exclusions of participants appropriate? | Unclear | Unclear | Unclear | Unclear | Unclear |
| Risk of bias introduced by participant selection (low/high/unclear) | Low | Low | Low | Low | Low |
| **B. Applicability** |  |  |  |  |  |
| Concern that included participants and setting do not match review question (low/high/unclear) | Low | Low | Low | Low | Low |
| **Domain 2** |  |  |  |  |  |
| **A. Risk of bias - predictors** |  |  |  |  |  |
| 2.1 Were predictors defined and evaluated in a similar way for all participants? | Yes | Yes | Yes | Yes | Yes |
| 2.2 Were predictor evaluations performed without knowing the outcome data? | Yes | Yes | Yes | Yes | Yes |
| 2.3 Are all the predictors available at the time the model is intended to be used? | Yes | Yes | Yes | Yes | Yes |
| Risk of bias introduced by the predictors or their assessment (low/high/unclear) | Low | Low | Low | Low | Low |
| **B. Applicability** |  |  |  |  |  |
| Concern that the definition, evaluation, or timing of predictors in the model do not match the review question (low/high/unclear) | Low | Low | Low | Low | Low |
| **Domain 3** |  |  |  |  |  |
| **A. Risk of bias - outcome** |  |  |  |  |  |
| Was the result properly determined? | Yes | Yes | Yes | Yes | Yes |
| Was a standard or prespecified outcome definition used? | Yes | Yes | Yes | Yes | Yes |
| Were predictors excluded from the outcome definition? | Yes | Yes | Yes | Yes | Yes |
| Was the outcome similarly defined and determined for all participants? | Yes | Yes | Yes | Yes | Yes |
| Was the outcome determined without knowing the predictor information? | Yes | Yes | Yes | Yes | Yes |
| Was the time interval between the evaluation of the predictor and the determination of the result adequate? | Yes | Yes | Yes | Yes | Yes |
| Risk of bias introduced by the result or its determination (low/high/unclear) | Low | Low | Low | Low | Low |
| **B. Applicability** |  |  |  |  |  |
| If a composite outcome was used, please describe the relative frequency/distribution of each contributing outcome: | Yes | Yes | Yes | Yes | Yes |
| Concern that the outcome, its definition, timing, or determination does not match the review question (low/high/unclear) | Low | Low | Low | Low | Low |
| **Domain 4** |  |  |  |  |  |
| **A. Risk of bias** |  |  |  |  |  |
| Describe the number of participants, number of candidate predictors, outcome events, and events per candidate predictor: | Yes | Yes | Yes | Yes | Yes |
| Describe how the model was developed (for example, with respect to modeling technique (eg, survival or logistic modeling), selection of predictors, and definition of risk groups): | Yes | Yes | Yes | Yes | Yes |
| Describe if and how the model was validated, either internally (eg, bootstrapping, cross-validation, split random sample) or externally (eg, temporal validation, geographic validation, different setting, different type of participants): | Yes | Yes | Yes | Yes | Yes |
| Describe the performance measures of the model, e.g. (re)calibration, discrimination, (re)classification, net profit and if adjusted for optimism: | Yes | Yes | Yes | Yes | Yes |
| Describe the participants who were excluded from the analysis: | Yes | Yes | Yes | Yes | Yes |
| Describe missing data on predictors and outcomes, as well as methods used for missing data: | Yes | Yes | Yes | Yes | Yes |
| 4.1 Was there a reasonable number of participants with the result? | Yes | Yes | Yes | Yes | Yes |
| 4.2 Were continuous and categorical predictors handled correctly? | Yes | Yes | Yes | Yes | Yes |
| 4.3 Were all enrolled participants included in the analysis? | Yes | Yes | Yes | Yes | Yes |
| 4.4 Were participants with missing data treated appropriately? | No | No | No | No | No |
| 4.5 Was selection of predictors based on univariate analysis avoided? | Yes | Yes | Yes | Yes | Yes |
| 4.6 Were the complexities of the data (eg censoring, competing risks, sampling of controls) adequately accounted for? | Yes | Yes | Yes | Yes | Yes |
| 4.7 Were relevant model performance measures adequately evaluated? | Yes | Yes | Yes | Yes | Yes |
| 4.8 Was model overfitting and optimism factored into model performance? | Yes | Yes | Yes | Yes | Yes |
| 4.9 Do the predictors and their assigned weights in the final model correspond to the results of the multivariate analysis? | Yes | Yes | Yes | Yes | Yes |
| **Risk of bias introduced by the analysis (low/high/Unclear)** | Low | Low | Low | Low | Low |
| **Step 4: General evaluation** | **-** | **-** | **-** | **-** | **-** |
| **Overall assessment of risk of bias** | Low | Low | Low | Low | Low |
| **General judgment of applicability** | Low | Low | Low | Low | Low |
|  |  |  |  |  |  |
|  |  |  |  |  |  |
| **Domain 1** | **Marziano et al. (2021)** | **Massonnaud et al. (2021)** | **Mathiot et al. (2021)** | **Matrajt et al. (2020)** | **Matrajt et al. (2021)** |
| **A. Risk of bias** | **08-07-22** | **08-07-22** | **08-07-22** | **08-07-22** | **08-07-22** |
| 1.1 Were appropriate data sources used, e.g. data from cohort studies, RCTs or nested case-controls? | Unclear | Unclear | Yes | Yes | Yes |
| 1.2 Were all inclusions and exclusions of participants appropriate? | No | No | Unclear | Unclear | Unclear |
| Risk of bias introduced by participant selection (low/high/unclear) | High | High | Low | Low | Low |
| **B. Applicability** |  |  |  |  |  |
| Concern that included participants and setting do not match review question (low/high/unclear) | Low | Low | Low | Low | Low |
| **Domain 2** |  |  |  |  |  |
| **A. Risk of bias - predictors** |  |  |  |  |  |
| 2.1 Were predictors defined and evaluated in a similar way for all participants? | No | Yes | Yes | Yes | Yes |
| 2.2 Were predictor evaluations performed without knowing the outcome data? | Unclear | Yes | Yes | Yes | Yes |
| 2.3 Are all the predictors available at the time the model is intended to be used? | Unclear | Yes | Yes | Yes | Yes |
| Risk of bias introduced by the predictors or their assessment (low/high/unclear) | High | Low | Low | Low | Low |
| **B. Applicability** |  |  |  |  |  |
| Concern that the definition, evaluation, or timing of predictors in the model do not match the review question (low/high/unclear) | Unclear | Low | Low | Low | Low |
| **Domain 3** |  |  |  |  |  |
| **A. Risk of bias - outcome** |  |  |  |  |  |
| Was the result properly determined? | Yes | Yes | Yes | Yes | Yes |
| Was a standard or prespecified outcome definition used? | No | Yes | Yes | Yes | Yes |
| Were predictors excluded from the outcome definition? | Unclear | Yes | Yes | Yes | Yes |
| Was the outcome similarly defined and determined for all participants? | Unclear | Yes | Yes | Yes | Yes |
| Was the outcome determined without knowing the predictor information? | Unclear | Yes | Yes | Yes | Yes |
| Was the time interval between the evaluation of the predictor and the determination of the result adequate? | Yes | Yes | Yes | Yes | Yes |
| Risk of bias introduced by the result or its determination (low/high/unclear) | High | Low | Low | Low | Low |
| **B. Applicability** |  |  |  |  |  |
| If a composite outcome was used, please describe the relative frequency/distribution of each contributing outcome: | Unclear | Yes | Yes | Yes | Yes |
| Concern that the outcome, its definition, timing, or determination does not match the review question (low/high/unclear) | Unclear | Low | Low | Low | Low |
| **Domain 4** |  |  |  |  |  |
| **A. Risk of bias** |  |  |  |  |  |
| Describe the number of participants, number of candidate predictors, outcome events, and events per candidate predictor: | No | Yes | Yes | Yes | Yes |
| Describe how the model was developed (for example, with respect to modeling technique (eg, survival or logistic modeling), selection of predictors, and definition of risk groups): | Unclear | Yes | Yes | Yes | Yes |
| Describe if and how the model was validated, either internally (eg, bootstrapping, cross-validation, split random sample) or externally (eg, temporal validation, geographic validation, different setting, different type of participants): | Unclear | Yes | Yes | Yes | Yes |
| Describe the performance measures of the model, e.g. (re)calibration, discrimination, (re)classification, net profit and if adjusted for optimism: | Unclear | Yes | Yes | Yes | Yes |
| Describe the participants who were excluded from the analysis: | No | Yes | Yes | Yes | Yes |
| Describe missing data on predictors and outcomes, as well as methods used for missing data: | No | Yes | Yes | Yes | Yes |
| 4.1 Was there a reasonable number of participants with the result? | Yes | Yes | Yes | Yes | Yes |
| 4.2 Were continuous and categorical predictors handled correctly? | Yes | Yes | Yes | Yes | Yes |
| 4.3 Were all enrolled participants included in the analysis? | Yes | Yes | Yes | Yes | Yes |
| 4.4 Were participants with missing data treated appropriately? | Yes | No | No | No | No |
| 4.5 Was selection of predictors based on univariate analysis avoided? | Yes | Yes | Yes | Yes | Yes |
| 4.6 Were the complexities of the data (eg censoring, competing risks, sampling of controls) adequately accounted for? | Yes | Yes | Yes | Yes | Yes |
| 4.7 Were relevant model performance measures adequately evaluated? | Yes | Yes | Yes | Yes | Yes |
| 4.8 Was model overfitting and optimism factored into model performance? | Yes | Yes | Yes | Yes | Yes |
| 4.9 Do the predictors and their assigned weights in the final model correspond to the results of the multivariate analysis? | No | Yes | Yes | Yes | Yes |
| **Risk of bias introduced by the analysis (low/high/Unclear)** | High | Low | Low | Low | Low |
| **Step 4: General evaluation** | This study does not have many details required to define the model | **-** | **-** | **-** | **-** |
| **Overall assessment of risk of bias** | High | High | Low | Low | Low |
| **General judgment of applicability** | High | Low | Low | Low | Low |
|  |  |  |  |  |  |
|  |  |  |  |  |  |
| **Domain 1** | **Matrajt et al. (2022)** | **McBryde et al. (2021)** | **Milne et al. (2022)** | **Min et al. (2021)** | **Miura et al. (2021)** |
| **A. Risk of bias** | **08-07-22** | **08-07-22** | **08-07-22** | **08-07-22** | **08-07-22** |
| 1.1 Were appropriate data sources used, e.g. data from cohort studies, RCTs or nested case-controls? | Yes | Yes | Yes | Yes | Yes |
| 1.2 Were all inclusions and exclusions of participants appropriate? | Unclear | Unclear | Unclear | Unclear | Unclear |
| Risk of bias introduced by participant selection (low/high/unclear) | Low | Low | Low | Low | Low |
| **B. Applicability** |  |  |  |  |  |
| Concern that included participants and setting do not match review question (low/high/unclear) | Low | Low | Low | Low | Low |
| **Domain 2** |  |  |  |  |  |
| **A. Risk of bias - predictors** |  |  |  |  |  |
| 2.1 Were predictors defined and evaluated in a similar way for all participants? | Yes | Yes | Yes | Yes | Yes |
| 2.2 Were predictor evaluations performed without knowing the outcome data? | Yes | Yes | Yes | Yes | Yes |
| 2.3 Are all the predictors available at the time the model is intended to be used? | Yes | Yes | Yes | Yes | Yes |
| Risk of bias introduced by the predictors or their assessment (low/high/unclear) | Low | Low | Low | Low | Low |
| **B. Applicability** |  |  |  |  |  |
| Concern that the definition, evaluation, or timing of predictors in the model do not match the review question (low/high/unclear) | Low | Low | Low | Low | Low |
| **Domain 3** |  |  |  |  |  |
| **A. Risk of bias - outcome** |  |  |  |  |  |
| Was the result properly determined? | Yes | Yes | Yes | Yes | Yes |
| Was a standard or prespecified outcome definition used? | Yes | Yes | Yes | Yes | Yes |
| Were predictors excluded from the outcome definition? | Yes | Yes | Yes | Yes | Yes |
| Was the outcome similarly defined and determined for all participants? | Yes | Yes | Yes | Yes | Yes |
| Was the outcome determined without knowing the predictor information? | Yes | Yes | Yes | Yes | Yes |
| Was the time interval between the evaluation of the predictor and the determination of the result adequate? | Yes | Yes | Yes | Yes | Yes |
| Risk of bias introduced by the result or its determination (low/high/unclear) | Low | Low | Low | Low | Low |
| **B. Applicability** |  |  |  |  |  |
| If a composite outcome was used, please describe the relative frequency/distribution of each contributing outcome: | Yes | Yes | Yes | Yes | Yes |
| Concern that the outcome, its definition, timing, or determination does not match the review question (low/high/unclear) | Low | Low | Low | Low | Low |
| **Domain 4** |  |  |  |  |  |
| **A. Risk of bias** |  |  |  |  |  |
| Describe the number of participants, number of candidate predictors, outcome events, and events per candidate predictor: | Yes | Yes | Yes | Yes | Yes |
| Describe how the model was developed (for example, with respect to modeling technique (eg, survival or logistic modeling), selection of predictors, and definition of risk groups): | Yes | Yes | Yes | Yes | Yes |
| Describe if and how the model was validated, either internally (eg, bootstrapping, cross-validation, split random sample) or externally (eg, temporal validation, geographic validation, different setting, different type of participants): | Yes | Yes | Yes | Yes | Yes |
| Describe the performance measures of the model, e.g. (re)calibration, discrimination, (re)classification, net profit and if adjusted for optimism: | Yes | Yes | Yes | Yes | Yes |
| Describe the participants who were excluded from the analysis: | Yes | Yes | Yes | Yes | Yes |
| Describe missing data on predictors and outcomes, as well as methods used for missing data: | Yes | Yes | Yes | Yes | Yes |
| 4.1 Was there a reasonable number of participants with the result? | Yes | Yes | Yes | Yes | Yes |
| 4.2 Were continuous and categorical predictors handled correctly? | Yes | Yes | Yes | Yes | Yes |
| 4.3 Were all enrolled participants included in the analysis? | Yes | Yes | Yes | Yes | Yes |
| 4.4 Were participants with missing data treated appropriately? | No | No | No | No | No |
| 4.5 Was selection of predictors based on univariate analysis avoided? | Yes | Yes | Yes | Yes | Yes |
| 4.6 Were the complexities of the data (eg censoring, competing risks, sampling of controls) adequately accounted for? | Yes | Yes | Yes | Yes | Yes |
| 4.7 Were relevant model performance measures adequately evaluated? | Yes | Yes | Yes | Yes | Yes |
| 4.8 Was model overfitting and optimism factored into model performance? | Yes | Yes | Yes | Yes | Yes |
| 4.9 Do the predictors and their assigned weights in the final model correspond to the results of the multivariate analysis? | Yes | Yes | Yes | Yes | Yes |
| **Risk of bias introduced by the analysis (low/high/Unclear)** | Low | Low | Low | Low | Low |
| **Step 4: General evaluation** | **-** | **-** | **-** | **-** | **-** |
| **Overall assessment of risk of bias** | Low | Low | Low | Low | Low |
| **General judgment of applicability** | Low | Low | Low | Low | Low |
|  |  |  |  |  |  |
|  |  |  |  |  |  |
| **Domain 1** | **Moghadas et al. (2021) (1)** | **Moghadas et al. (2021) (2)** | **Moghadas et al. (2021) (3)** | **Moldokmatova et al. (2021)** | **Moore et al. (2021)** |
| **A. Risk of bias** | **08-07-22** | **08-07-22** | **08-07-22** | **08-07-22** | **09-07-22** |
| 1.1 Were appropriate data sources used, e.g. data from cohort studies, RCTs or nested case-controls? | Yes | Yes | Yes | Yes | Yes |
| 1.2 Were all inclusions and exclusions of participants appropriate? | Yes | Unclear | Unclear | Unclear | Unclear |
| Risk of bias introduced by participant selection (low/high/unclear) | Low | Low | Low | Low | Low |
| **B. Applicability** |  |  |  |  |  |
| Concern that included participants and setting do not match review question (low/high/unclear) | Low | Low | Low | Low | Low |
| **Domain 2** |  |  |  |  |  |
| **A. Risk of bias - predictors** |  |  |  |  |  |
| 2.1 Were predictors defined and evaluated in a similar way for all participants? | Yes | Yes | Yes | Yes | Yes |
| 2.2 Were predictor evaluations performed without knowing the outcome data? | Yes | Yes | Yes | Yes | Yes |
| 2.3 Are all the predictors available at the time the model is intended to be used? | Yes | Yes | Yes | Yes | Yes |
| Risk of bias introduced by the predictors or their assessment (low/high/unclear) | Low | Low | Low | Low | Low |
| **B. Applicability** |  |  |  |  |  |
| Concern that the definition, evaluation, or timing of predictors in the model do not match the review question (low/high/unclear) | Low | Low | Low | Low | Low |
| **Domain 3** |  |  |  |  |  |
| **A. Risk of bias - outcome** |  |  |  |  |  |
| Was the result properly determined? | Yes | Yes | Yes | Yes | Yes |
| Was a standard or prespecified outcome definition used? | Yes | Yes | Yes | Yes | Yes |
| Were predictors excluded from the outcome definition? | Yes | Yes | Yes | Yes | Yes |
| Was the outcome similarly defined and determined for all participants? | Yes | Yes | Yes | Yes | Yes |
| Was the outcome determined without knowing the predictor information? | Yes | Yes | Yes | Yes | Yes |
| Was the time interval between the evaluation of the predictor and the determination of the result adequate? | Yes | Yes | Yes | Yes | Yes |
| Risk of bias introduced by the result or its determination (low/high/unclear) | Low | Low | Low | Low | Low |
| **B. Applicability** |  |  |  |  |  |
| If a composite outcome was used, please describe the relative frequency/distribution of each contributing outcome: | Yes | Yes | Yes | Yes | Yes |
| Concern that the outcome, its definition, timing, or determination does not match the review question (low/high/unclear) | Low | Low | Low | Low | Low |
| **Domain 4** |  |  |  |  |  |
| **A. Risk of bias** |  |  |  |  |  |
| Describe the number of participants, number of candidate predictors, outcome events, and events per candidate predictor: | Yes | Yes | Yes | Yes | Yes |
| Describe how the model was developed (for example, with respect to modeling technique (eg, survival or logistic modeling), selection of predictors, and definition of risk groups): | Yes | Yes | Yes | Yes | Yes |
| Describe if and how the model was validated, either internally (eg, bootstrapping, cross-validation, split random sample) or externally (eg, temporal validation, geographic validation, different setting, different type of participants): | Yes | Yes | Yes | Yes | Yes |
| Describe the performance measures of the model, e.g. (re)calibration, discrimination, (re)classification, net profit and if adjusted for optimism: | Yes | Yes | Yes | Yes | Yes |
| Describe the participants who were excluded from the analysis: | Yes | Yes | Yes | Yes | Yes |
| Describe missing data on predictors and outcomes, as well as methods used for missing data: | Yes | Yes | Yes | Yes | Yes |
| 4.1 Was there a reasonable number of participants with the result? | Yes | Yes | Yes | Yes | Yes |
| 4.2 Were continuous and categorical predictors handled correctly? | Yes | Yes | Yes | Yes | Yes |
| 4.3 Were all enrolled participants included in the analysis? | Yes | Yes | Yes | Yes | Yes |
| 4.4 Were participants with missing data treated appropriately? | No | No | No | No | No |
| 4.5 Was selection of predictors based on univariate analysis avoided? | Yes | Yes | Yes | Yes | Yes |
| 4.6 Were the complexities of the data (eg censoring, competing risks, sampling of controls) adequately accounted for? | Yes | Yes | Yes | Yes | Yes |
| 4.7 Were relevant model performance measures adequately evaluated? | Yes | Yes | Yes | Yes | Yes |
| 4.8 Was model overfitting and optimism factored into model performance? | Yes | Yes | Yes | Yes | Yes |
| 4.9 Do the predictors and their assigned weights in the final model correspond to the results of the multivariate analysis? | Yes | Yes | Yes | Yes | Yes |
| **Risk of bias introduced by the analysis (low/high/Unclear)** | Low | Low | Low | Low | Low |
| **Step 4: General evaluation** | **-** | **-** | **-** | **-** | **-** |
| **Overall assessment of risk of bias** | Low | Low | Low | Low | Low |
| **General judgment of applicability** | Low | Low | Low | Low | Low |
|  |  |  |  |  |  |
|  |  |  |  |  |  |
| **Domain 1** | **Moore et al. (2021)** | **Morales-Zamora et al. (2022)** | **Mukandavire et al. (2020)** | **Mumtaz et al. (2021)** | **Musa et al. (2020)** |
| **A. Risk of bias** | **09-07-22** | **09-07-22** | **09-07-22** | **09-07-22** | **09-07-22** |
| 1.1 Were appropriate data sources used, e.g. data from cohort studies, RCTs or nested case-controls? | Yes | Yes | Yes | Unclear | Yes |
| 1.2 Were all inclusions and exclusions of participants appropriate? | Unclear | Yes | Yes | Unclear | Yes |
| Risk of bias introduced by participant selection (low/high/unclear) | Low | Low | Low | Unclear | Low |
| **B. Applicability** |  |  |  |  |  |
| Concern that included participants and setting do not match review question (low/high/unclear) | Low | Low | Low | Low | Low |
| **Domain 2** |  |  |  |  |  |
| **A. Risk of bias - predictors** |  |  |  |  |  |
| 2.1 Were predictors defined and evaluated in a similar way for all participants? | Yes | Yes | Yes | Yes | Yes |
| 2.2 Were predictor evaluations performed without knowing the outcome data? | Yes | Yes | Yes | Yes | Yes |
| 2.3 Are all the predictors available at the time the model is intended to be used? | Yes | Yes | Yes | Yes | Yes |
| Risk of bias introduced by the predictors or their assessment (low/high/unclear) | Low | Low | Low | Low | Low |
| **B. Applicability** |  |  |  |  |  |
| Concern that the definition, evaluation, or timing of predictors in the model do not match the review question (low/high/unclear) | Low | Low | Low | Low | Low |
| **Domain 3** |  |  |  |  |  |
| **A. Risk of bias - outcome** |  |  |  |  |  |
| Was the result properly determined? | Yes | Yes | Yes | Yes | Yes |
| Was a standard or prespecified outcome definition used? | Yes | Yes | Unclear | Unclear | Yes |
| Were predictors excluded from the outcome definition? | Yes | Yes | Yes | Yes | Yes |
| Was the outcome similarly defined and determined for all participants? | Yes | Yes | Yes | Yes | Yes |
| Was the outcome determined without knowing the predictor information? | Yes | Yes | Yes | Yes | Yes |
| Was the time interval between the evaluation of the predictor and the determination of the result adequate? | Yes | Yes | Yes | Yes | Yes |
| Risk of bias introduced by the result or its determination (low/high/unclear) | Low | Low | Low | Low | Low |
| **B. Applicability** |  |  |  |  |  |
| If a composite outcome was used, please describe the relative frequency/distribution of each contributing outcome: | Yes | NA | NA | NA | NA |
| Concern that the outcome, its definition, timing, or determination does not match the review question (low/high/unclear) | Low | Low | Low | Low | Low |
| **Domain 4** |  |  |  |  |  |
| **A. Risk of bias** |  |  |  |  |  |
| Describe the number of participants, number of candidate predictors, outcome events, and events per candidate predictor: | Yes | Yes | Yes | Yes | Yes |
| Describe how the model was developed (for example, with respect to modeling technique (eg, survival or logistic modeling), selection of predictors, and definition of risk groups): | Yes | Yes | Yes | Yes | Yes |
| Describe if and how the model was validated, either internally (eg, bootstrapping, cross-validation, split random sample) or externally (eg, temporal validation, geographic validation, different setting, different type of participants): | Yes | Yes | Yes | Yes | Yes |
| Describe the performance measures of the model, e.g. (re)calibration, discrimination, (re)classification, net profit and if adjusted for optimism: | Yes | Yes | Yes | Yes | Yes |
| Describe the participants who were excluded from the analysis: | Yes | Yes | Yes | Yes | Yes |
| Describe missing data on predictors and outcomes, as well as methods used for missing data: | Yes | Yes | Yes | Yes | Yes |
| 4.1 Was there a reasonable number of participants with the result? | Yes | Yes | Yes | Yes | Yes |
| 4.2 Were continuous and categorical predictors handled correctly? | Yes | Yes | Yes | Yes | Yes |
| 4.3 Were all enrolled participants included in the analysis? | Yes | Yes | Yes | Yes | Yes |
| 4.4 Were participants with missing data treated appropriately? | No | No | No | No | No |
| 4.5 Was selection of predictors based on univariate analysis avoided? | Yes | Yes | Yes | Yes | Yes |
| 4.6 Were the complexities of the data (eg censoring, competing risks, sampling of controls) adequately accounted for? | Yes | Yes | Yes | Yes | Yes |
| 4.7 Were relevant model performance measures adequately evaluated? | Yes | Yes | Yes | Yes | Yes |
| 4.8 Was model overfitting and optimism factored into model performance? | Yes | Yes | Yes | Yes | Yes |
| 4.9 Do the predictors and their assigned weights in the final model correspond to the results of the multivariate analysis? | Yes | Yes | Yes | Yes | Yes |
| **Risk of bias introduced by the analysis (low/high/Unclear)** | Low | Low | Low | Low | Low |
| **Step 4: General evaluation** | **-** | **-** | **-** | No specific definitions of population inclusion are found | **-** |
| **Overall assessment of risk of bias** | Low | Low | Low | Unclear | Low |
| **General judgment of applicability** | Low | Low | Low | Low | Low |
|  |  |  |  |  |  |
|  |  |  |  |  |  |
| **Domain 1** | **Rabiu et al. (2021)** | **Nakhaeizadeh et al. (2022)** | **Nam et al. (2021)** | **Nichita et al. (2021)** | **Nixon et al. (2021)** |
| **A. Risk of bias** | **09-07-22** | **09-07-22** | **09-07-22** | **09-07-22** | **09-07-22** |
| 1.1 Were appropriate data sources used, e.g. data from cohort studies, RCTs or nested case-controls? | Yes | Unclear | Yes | Yes | Yes |
| 1.2 Were all inclusions and exclusions of participants appropriate? | Yes | Unclear | Yes | Yes | Yes |
| Risk of bias introduced by participant selection (low/high/unclear) | Low | Unclear | Low | Low | Low |
| **B. Applicability** |  |  |  |  |  |
| Concern that included participants and setting do not match review question (low/high/unclear) | Low | Low | Low | Low | Low |
| **Domain 2** |  |  |  |  |  |
| **A. Risk of bias - predictors** |  |  |  |  |  |
| 2.1 Were predictors defined and evaluated in a similar way for all participants? | Yes | Yes | Yes | Yes | Yes |
| 2.2 Were predictor evaluations performed without knowing the outcome data? | Yes | Yes | Yes | Yes | Yes |
| 2.3 Are all the predictors available at the time the model is intended to be used? | Yes | Yes | Yes | Yes | Yes |
| Risk of bias introduced by the predictors or their assessment (low/high/unclear) | Low | Low | Low | Low | Low |
| **B. Applicability** |  |  |  |  |  |
| Concern that the definition, evaluation, or timing of predictors in the model do not match the review question (low/high/unclear) | Low | Low | Low | Low | Low |
| **Domain 3** |  |  |  |  |  |
| **A. Risk of bias - outcome** |  |  |  |  |  |
| Was the result properly determined? | Yes | Yes | Yes | Yes | Yes |
| Was a standard or prespecified outcome definition used? | Yes | Yes | Yes | Yes | Yes |
| Were predictors excluded from the outcome definition? | Yes | Yes | Yes | Yes | Yes |
| Was the outcome similarly defined and determined for all participants? | Yes | Yes | Yes | Yes | Yes |
| Was the outcome determined without knowing the predictor information? | Yes | Yes | Yes | Yes | Yes |
| Was the time interval between the evaluation of the predictor and the determination of the result adequate? | Yes | Yes | Yes | Yes | Yes |
| Risk of bias introduced by the result or its determination (low/high/unclear) | Low | Low | Low | Low | Low |
| **B. Applicability** |  |  |  |  |  |
| If a composite outcome was used, please describe the relative frequency/distribution of each contributing outcome: | NA | NA | NA | NA | NA |
| Concern that the outcome, its definition, timing, or determination does not match the review question (low/high/unclear) | Low | Low | Low | Low | Low |
| **Domain 4** |  |  |  |  |  |
| **A. Risk of bias** |  |  |  |  |  |
| Describe the number of participants, number of candidate predictors, outcome events, and events per candidate predictor: | Yes | Yes | Yes | Yes | Yes |
| Describe how the model was developed (for example, with respect to modeling technique (eg, survival or logistic modeling), selection of predictors, and definition of risk groups): | Yes | Yes | Yes | Yes | Yes |
| Describe if and how the model was validated, either internally (eg, bootstrapping, cross-validation, split random sample) or externally (eg, temporal validation, geographic validation, different setting, different type of participants): | Yes | Yes | Yes | Yes | Yes |
| Describe the performance measures of the model, e.g. (re)calibration, discrimination, (re)classification, net profit and if adjusted for optimism: | Yes | Yes | Yes | Yes | Yes |
| Describe the participants who were excluded from the analysis: | Yes | Yes | Yes | Yes | Yes |
| Describe missing data on predictors and outcomes, as well as methods used for missing data: | Yes | Yes | Yes | Yes | Yes |
| 4.1 Was there a reasonable number of participants with the result? | Yes | Yes | Yes | Yes | Yes |
| 4.2 Were continuous and categorical predictors handled correctly? | Yes | Yes | Yes | Yes | Yes |
| 4.3 Were all enrolled participants included in the analysis? | Yes | Yes | Yes | Yes | Yes |
| 4.4 Were participants with missing data treated appropriately? | No | No | No | No | No |
| 4.5 Was selection of predictors based on univariate analysis avoided? | Yes | Yes | Yes | Yes | Yes |
| 4.6 Were the complexities of the data (eg censoring, competing risks, sampling of controls) adequately accounted for? | Yes | Yes | Yes | Yes | Yes |
| 4.7 Were relevant model performance measures adequately evaluated? | Yes | Yes | Yes | Yes | Yes |
| 4.8 Was model overfitting and optimism factored into model performance? | Yes | Yes | Yes | Yes | Yes |
| 4.9 Do the predictors and their assigned weights in the final model correspond to the results of the multivariate analysis? | Yes | Yes | Yes | Yes | Yes |
| **Risk of bias introduced by the analysis (low/high/Unclear)** | Low | Low | Low | Low | Low |
| **Step 4: General evaluation** | **-** | No specific definitions of population inclusion are found | **-** | **-** | **-** |
| **Overall assessment of risk of bias** | Low | Unclear | Low | Low | Low |
| **General judgment of applicability** | Low | Low | Low | Low | Low |
|  |  |  |  |  |  |
|  |  |  |  |  |  |
| **Domain 1** | **Nuraini et al. (2021)** | **Oloniiju et al. (2022)** | **Omae et al. (2022) (1)** | **Omae et al. (2022)** | **Omar et al. (2021)** |
| **A. Risk of bias** | **09-07-22** | **09-07-22** | **09-07-22** | **09-07-22** | **09-07-22** |
| 1.1 Were appropriate data sources used, e.g. data from cohort studies, RCTs or nested case-controls? | Yes | Yes | Yes | Yes | Yes |
| 1.2 Were all inclusions and exclusions of participants appropriate? | Yes | Yes | Yes | Yes | Unclear |
| Risk of bias introduced by participant selection (low/high/unclear) | Low | Low | Low | Low | Low |
| **B. Applicability** |  |  |  |  |  |
| Concern that included participants and setting do not match review question (low/high/unclear) | Low | Low | Low | Low | Low |
| **Domain 2** |  |  |  |  |  |
| **A. Risk of bias - predictors** |  |  |  |  |  |
| 2.1 Were predictors defined and evaluated in a similar way for all participants? | Yes | Yes | Yes | Yes | Yes |
| 2.2 Were predictor evaluations performed without knowing the outcome data? | Yes | Yes | Yes | Yes | Yes |
| 2.3 Are all the predictors available at the time the model is intended to be used? | Yes | Yes | Yes | Yes | Yes |
| Risk of bias introduced by the predictors or their assessment (low/high/unclear) | Low | Low | Low | Low | Low |
| **B. Applicability** |  |  |  |  |  |
| Concern that the definition, evaluation, or timing of predictors in the model do not match the review question (low/high/unclear) | Low | Low | Low | Low | Low |
| **Domain 3** |  |  |  |  |  |
| **A. Risk of bias - outcome** |  |  |  |  |  |
| Was the result properly determined? | Yes | Yes | Yes | Yes | Yes |
| Was a standard or prespecified outcome definition used? | Yes | Yes | Yes | Yes | Yes |
| Were predictors excluded from the outcome definition? | Yes | Yes | Yes | Yes | Yes |
| Was the outcome similarly defined and determined for all participants? | Yes | Yes | Yes | Yes | Yes |
| Was the outcome determined without knowing the predictor information? | Yes | Yes | Yes | Yes | Yes |
| Was the time interval between the evaluation of the predictor and the determination of the result adequate? | Yes | Yes | Yes | Yes | Yes |
| Risk of bias introduced by the result or its determination (low/high/unclear) | Low | Low | Low | Low | Low |
| **B. Applicability** |  |  |  |  |  |
| If a composite outcome was used, please describe the relative frequency/distribution of each contributing outcome: | NA | NA | NA | NA | NA |
| Concern that the outcome, its definition, timing, or determination does not match the review question (low/high/unclear) | Low | Low | Low | Low | Low |
| **Domain 4** |  |  |  |  |  |
| **A. Risk of bias** |  |  |  |  |  |
| Describe the number of participants, number of candidate predictors, outcome events, and events per candidate predictor: | Yes | Yes | Yes | Yes | Yes |
| Describe how the model was developed (for example, with respect to modeling technique (eg, survival or logistic modeling), selection of predictors, and definition of risk groups): | Yes | Yes | Yes | Yes | Yes |
| Describe if and how the model was validated, either internally (eg, bootstrapping, cross-validation, split random sample) or externally (eg, temporal validation, geographic validation, different setting, different type of participants): | Yes | Yes | Yes | Yes | Yes |
| Describe the performance measures of the model, e.g. (re)calibration, discrimination, (re)classification, net profit and if adjusted for optimism: | Yes | Yes | Yes | Yes | Yes |
| Describe the participants who were excluded from the analysis: | Yes | Yes | Yes | Yes | Yes |
| Describe missing data on predictors and outcomes, as well as methods used for missing data: | Yes | Yes | Yes | Yes | Yes |
| 4.1 Was there a reasonable number of participants with the result? | Yes | Yes | Yes | Yes | Yes |
| 4.2 Were continuous and categorical predictors handled correctly? | Yes | Yes | Yes | Yes | Yes |
| 4.3 Were all enrolled participants included in the analysis? | Yes | Yes | Yes | Yes | Yes |
| 4.4 Were participants with missing data treated appropriately? | No | No | No | No | No |
| 4.5 Was selection of predictors based on univariate analysis avoided? | Yes | Yes | Yes | Yes | Yes |
| 4.6 Were the complexities of the data (eg censoring, competing risks, sampling of controls) adequately accounted for? | Yes | Yes | Yes | Yes | Yes |
| 4.7 Were relevant model performance measures adequately evaluated? | Yes | Yes | Yes | Yes | Yes |
| 4.8 Was model overfitting and optimism factored into model performance? | Yes | Yes | Yes | Yes | Yes |
| 4.9 Do the predictors and their assigned weights in the final model correspond to the results of the multivariate analysis? | Yes | Yes | Yes | Yes | Yes |
| **Risk of bias introduced by the analysis (low/high/Unclear)** | Low | Low | Low | Low | Low |
| **Step 4: General evaluation** |  |  |  |  |  |
| **Overall assessment of risk of bias** | Low | Low | Low | Low | Low |
| **General judgment of applicability** | Low | Low | Low | Low | Low |
|  |  |  |  |  |  |
|  |  |  |  |  |  |
| **Domain 1** | **Omar et al. (2022)** | **Padula et al. (2021)** | **Pae (2021)** | **Pageaud et al. (2021)** | **Paltiel et al. (2021)** |
| **A. Risk of bias** | **09-07-22** | **09-07-22** | **09-07-22** | **09-07-22** | **09-07-22** |
| 1.1 Were appropriate data sources used, e.g. data from cohort studies, RCTs or nested case-controls? | Yes | Yes | Unclear | Yes | Yes |
| 1.2 Were all inclusions and exclusions of participants appropriate? | Unclear | Unclear | Unclear | Unclear | Unclear |
| Risk of bias introduced by participant selection (low/high/unclear) | Low | Low | Unclear | Low | Low |
| **B. Applicability** |  |  |  |  |  |
| Concern that included participants and setting do not match review question (low/high/unclear) | Low | Low | Low | Low | Low |
| **Domain 2** |  |  |  |  |  |
| **A. Risk of bias - predictors** |  |  |  |  |  |
| 2.1 Were predictors defined and evaluated in a similar way for all participants? | Yes | Yes | Yes | Yes | Yes |
| 2.2 Were predictor evaluations performed without knowing the outcome data? | Yes | Yes | Yes | Yes | Yes |
| 2.3 Are all the predictors available at the time the model is intended to be used? | Yes | Yes | Yes | Yes | Yes |
| Risk of bias introduced by the predictors or their assessment (low/high/unclear) | Low | Low | Low | Low | Low |
| **B. Applicability** |  |  |  |  |  |
| Concern that the definition, evaluation, or timing of predictors in the model do not match the review question (low/high/unclear) | Low | Low | Low | Low | Low |
| **Domain 3** |  |  |  |  |  |
| **A. Risk of bias - outcome** |  |  |  |  |  |
| Was the result properly determined? | Yes | Yes | Yes | Yes | Yes |
| Was a standard or prespecified outcome definition used? | Yes | Yes | Yes | Yes | Yes |
| Were predictors excluded from the outcome definition? | Yes | Yes | Yes | Yes | Yes |
| Was the outcome similarly defined and determined for all participants? | Yes | Yes | Yes | Yes | Yes |
| Was the outcome determined without knowing the predictor information? | Yes | Yes | Yes | Yes | Yes |
| Was the time interval between the evaluation of the predictor and the determination of the result adequate? | Yes | Yes | Yes | Yes | Yes |
| Risk of bias introduced by the result or its determination (low/high/unclear) | Low | Low | Low | Low | Low |
| **B. Applicability** |  |  |  |  |  |
| If a composite outcome was used, please describe the relative frequency/distribution of each contributing outcome: | NA | NA | NA | Yes | Yes |
| Concern that the outcome, its definition, timing, or determination does not match the review question (low/high/unclear) | Low | Low | Low | Low | Low |
| **Domain 4** |  |  |  |  |  |
| **A. Risk of bias** |  |  |  |  |  |
| Describe the number of participants, number of candidate predictors, outcome events, and events per candidate predictor: | Yes | Yes | Yes | Yes | Yes |
| Describe how the model was developed (for example, with respect to modeling technique (eg, survival or logistic modeling), selection of predictors, and definition of risk groups): | Yes | Yes | Yes | Yes | Yes |
| Describe if and how the model was validated, either internally (eg, bootstrapping, cross-validation, split random sample) or externally (eg, temporal validation, geographic validation, different setting, different type of participants): | Yes | Yes | Yes | Yes | Yes |
| Describe the performance measures of the model, e.g. (re)calibration, discrimination, (re)classification, net profit and if adjusted for optimism: | Yes | Yes | Yes | Yes | Yes |
| Describe the participants who were excluded from the analysis: | Yes | Yes | Yes | Yes | Yes |
| Describe missing data on predictors and outcomes, as well as methods used for missing data: | Yes | Yes | Yes | Yes | Yes |
| 4.1 Was there a reasonable number of participants with the result? | Yes | Yes | Yes | Yes | Yes |
| 4.2 Were continuous and categorical predictors handled correctly? | Yes | Yes | Yes | Yes | Yes |
| 4.3 Were all enrolled participants included in the analysis? | Yes | Yes | Yes | Yes | Yes |
| 4.4 Were participants with missing data treated appropriately? | No | No | No | No | No |
| 4.5 Was selection of predictors based on univariate analysis avoided? | Yes | Yes | Yes | Yes | Yes |
| 4.6 Were the complexities of the data (eg censoring, competing risks, sampling of controls) adequately accounted for? | Yes | Yes | Yes | Yes | Yes |
| 4.7 Were relevant model performance measures adequately evaluated? | Yes | Yes | Yes | Yes | Yes |
| 4.8 Was model overfitting and optimism factored into model performance? | Yes | Yes | Yes | Yes | Yes |
| 4.9 Do the predictors and their assigned weights in the final model correspond to the results of the multivariate analysis? | Yes | Yes | Yes | Yes | Yes |
| **Risk of bias introduced by the analysis (low/high/Unclear)** | Low | Low | Low | Low | Low |
| **Step 4: General evaluation** | **-** | **-** | There are no specific data on the selection criteria of the population included, however, the origin of the database is mentioned | **-** | **-** |
| **Overall assessment of risk of bias** | Low | Low | Unclear | Low | Low |
| **General judgment of applicability** | Low | Low | Low | Low | Low |
|  |  |  |  |  |  |
|  |  |  |  |  |  |
| **Domain 1** | **Pan et al. (2022)** | **Parino et al. (2021)** | **Parolini et al. (2022)** | **Patel et al. (2021)** | **Pearson et al. (2021)** |
| **A. Risk of bias** | **09-07-22** | **09-07-22** | **09-07-22** | **09-07-22** | **16-07-22** |
| 1.1 Were appropriate data sources used, e.g. data from cohort studies, RCTs or nested case-controls? | Yes | Unclear | Unclear | Unclear | Unclear |
| 1.2 Were all inclusions and exclusions of participants appropriate? | Unclear | Unclear | No | No | No |
| Risk of bias introduced by participant selection (low/high/unclear) | Low | Unclear | High | High | High |
| **B. Applicability** |  |  |  |  |  |
| Concern that included participants and setting do not match review question (low/high/unclear) | Low | Low | Low | Low | Low |
| **Domain 2** |  |  |  |  |  |
| **A. Risk of bias - predictors** |  |  |  |  |  |
| 2.1 Were predictors defined and evaluated in a similar way for all participants? | Yes | Yes | Yes | Yes | No |
| 2.2 Were predictor evaluations performed without knowing the outcome data? | Yes | Yes | Yes | Yes | Unclear |
| 2.3 Are all the predictors available at the time the model is intended to be used? | Yes | Yes | Yes | Yes | Unclear |
| Risk of bias introduced by the predictors or their assessment (low/high/unclear) | Low | Low | Low | Low | High |
| **B. Applicability** |  |  |  |  |  |
| Concern that the definition, evaluation, or timing of predictors in the model do not match the review question (low/high/unclear) | Low | Low | Low | Low | Unclear |
| **Domain 3** |  |  |  |  |  |
| **A. Risk of bias - outcome** |  |  |  |  |  |
| Was the result properly determined? | Yes | Yes | Yes | Yes | Yes |
| Was a standard or prespecified outcome definition used? | Yes | Unclear | Yes | Yes | No |
| Were predictors excluded from the outcome definition? | Yes | Yes | Yes | Yes | Unclear |
| Was the outcome similarly defined and determined for all participants? | Yes | Yes | Yes | Yes | Unclear |
| Was the outcome determined without knowing the predictor information? | Yes | Yes | Yes | Yes | Unclear |
| Was the time interval between the evaluation of the predictor and the determination of the result adequate? | Yes | Yes | Yes | Yes | Yes |
| Risk of bias introduced by the result or its determination (low/high/unclear) | Low | Low | Low | Low | High |
| **B. Applicability** |  |  |  |  |  |
| If a composite outcome was used, please describe the relative frequency/distribution of each contributing outcome: | Yes | NA | Yes | Yes | Unclear |
| Concern that the outcome, its definition, timing, or determination does not match the review question (low/high/unclear) | Low | Low | Low | Low | Unclear |
| **Domain 4** |  |  |  |  |  |
| **A. Risk of bias** |  |  |  |  |  |
| Describe the number of participants, number of candidate predictors, outcome events, and events per candidate predictor: | Yes | Yes | Yes | Yes | No |
| Describe how the model was developed (for example, with respect to modeling technique (eg, survival or logistic modeling), selection of predictors, and definition of risk groups): | Yes | Yes | Yes | Yes | Unclear |
| Describe if and how the model was validated, either internally (eg, bootstrapping, cross-validation, split random sample) or externally (eg, temporal validation, geographic validation, different setting, different type of participants): | Yes | Yes | Yes | Yes | Unclear |
| Describe the performance measures of the model, e.g. (re)calibration, discrimination, (re)classification, net profit and if adjusted for optimism: | Yes | Yes | Yes | Yes | Unclear |
| Describe the participants who were excluded from the analysis: | Yes | Yes | Yes | Yes | No |
| Describe missing data on predictors and outcomes, as well as methods used for missing data: | Yes | Yes | Yes | Yes | No |
| 4.1 Was there a reasonable number of participants with the result? | Yes | Yes | Yes | Yes | Yes |
| 4.2 Were continuous and categorical predictors handled correctly? | Yes | Yes | Yes | Yes | Yes |
| 4.3 Were all enrolled participants included in the analysis? | Yes | Yes | Yes | Yes | Yes |
| 4.4 Were participants with missing data treated appropriately? | No | No | No | No | Yes |
| 4.5 Was selection of predictors based on univariate analysis avoided? | Yes | Yes | Yes | Yes | Yes |
| 4.6 Were the complexities of the data (eg censoring, competing risks, sampling of controls) adequately accounted for? | Yes | Yes | Yes | Yes | Yes |
| 4.7 Were relevant model performance measures adequately evaluated? | Yes | Yes | Yes | Yes | Yes |
| 4.8 Was model overfitting and optimism factored into model performance? | Yes | Yes | Yes | Yes | Yes |
| 4.9 Do the predictors and their assigned weights in the final model correspond to the results of the multivariate analysis? | Yes | Yes | Yes | Yes | No |
| **Risk of bias introduced by the analysis (low/high/Unclear)** | Low | Low | Low | Low | High |
| **Step 4: General evaluation** | **-** | No specific definitions of population inclusion are found | The details of the subjects included are not clear. Although they mention that a cohort is established | The details of the subjects included are not clear. Although they mention that a cohort is established | This study does not have many details required to define the model |
| **Overall assessment of risk of bias** | Low | Unclear | High | High | High |
| **General judgment of applicability** | Low | Low | Low | Low | High |
|  |  |  |  |  |  |
|  |  |  |  |  |  |
| **Domain 1** | **Pérez et al. (2021)** | **Rachaniotis et al. (2021)** | **Rahmandan et al. (2021)** | **Raina et al. (2021)** | **Rajakaruna et al. (2022)** |
| **A. Risk of bias** | **16-07-22** | **16-07-22** | **16-07-22** | **16-07-22** | **16-07-22** |
| 1.1 Were appropriate data sources used, e.g. data from cohort studies, RCTs or nested case-controls? | Yes | Yes | Yes | Yes | Yes |
| 1.2 Were all inclusions and exclusions of participants appropriate? | Unclear | Unclear | Unclear | Unclear | Yes |
| Risk of bias introduced by participant selection (low/high/unclear) | Low | Low | Low | Low | Low |
| **B. Applicability** |  |  |  |  |  |
| Concern that included participants and setting do not match review question (low/high/unclear) | Low | Low | Low | Low | Low |
| **Domain 2** |  |  |  |  |  |
| **A. Risk of bias - predictors** |  |  |  |  |  |
| 2.1 Were predictors defined and evaluated in a similar way for all participants? | Yes | Yes | Yes | Yes | Yes |
| 2.2 Were predictor evaluations performed without knowing the outcome data? | Yes | Yes | Yes | Yes | Yes |
| 2.3 Are all the predictors available at the time the model is intended to be used? | Yes | Yes | Yes | Yes | Yes |
| Risk of bias introduced by the predictors or their assessment (low/high/unclear) | Low | Low | Low | Low | Low |
| **B. Applicability** |  |  |  |  |  |
| Concern that the definition, evaluation, or timing of predictors in the model do not match the review question (low/high/unclear) | Low | Low | Low | Low | Low |
| **Domain 3** |  |  |  |  |  |
| **A. Risk of bias - outcome** |  |  |  |  |  |
| Was the result properly determined? | Yes | Yes | Yes | Yes | Yes |
| Was a standard or prespecified outcome definition used? | Yes | Yes | Yes | Yes | Yes |
| Were predictors excluded from the outcome definition? | Yes | Yes | Yes | Yes | Yes |
| Was the outcome similarly defined and determined for all participants? | Yes | Yes | Yes | Yes | Yes |
| Was the outcome determined without knowing the predictor information? | Yes | Yes | Yes | Yes | Yes |
| Was the time interval between the evaluation of the predictor and the determination of the result adequate? | Yes | Yes | Yes | Yes | Yes |
| Risk of bias introduced by the result or its determination (low/high/unclear) | Low | Low | Low | Low | Low |
| **B. Applicability** |  |  |  |  |  |
| If a composite outcome was used, please describe the relative frequency/distribution of each contributing outcome: | Yes | Yes | Yes | Yes | Yes |
| Concern that the outcome, its definition, timing, or determination does not match the review question (low/high/unclear) | Low | Low | Low | Low | Low |
| **Domain 4** |  |  |  |  |  |
| **A. Risk of bias** |  |  |  |  |  |
| Describe the number of participants, number of candidate predictors, outcome events, and events per candidate predictor: | Yes | Yes | Yes | Yes | Yes |
| Describe how the model was developed (for example, with respect to modeling technique (eg, survival or logistic modeling), selection of predictors, and definition of risk groups): | Yes | Yes | Yes | Yes | Yes |
| Describe if and how the model was validated, either internally (eg, bootstrapping, cross-validation, split random sample) or externally (eg, temporal validation, geographic validation, different setting, different type of participants): | Yes | Yes | Yes | Yes | Yes |
| Describe the performance measures of the model, e.g. (re)calibration, discrimination, (re)classification, net profit and if adjusted for optimism: | Yes | Yes | Yes | Yes | Yes |
| Describe the participants who were excluded from the analysis: | Yes | Yes | Yes | Yes | Yes |
| Describe missing data on predictors and outcomes, as well as methods used for missing data: | Yes | Yes | Yes | Yes | Yes |
| 4.1 Was there a reasonable number of participants with the result? | Yes | Yes | Yes | Yes | Yes |
| 4.2 Were continuous and categorical predictors handled correctly? | Yes | Yes | Yes | Yes | Yes |
| 4.3 Were all enrolled participants included in the analysis? | Yes | Yes | Yes | Yes | Yes |
| 4.4 Were participants with missing data treated appropriately? | No | No | No | No | No |
| 4.5 Was selection of predictors based on univariate analysis avoided? | Yes | Yes | Yes | Yes | Yes |
| 4.6 Were the complexities of the data (eg censoring, competing risks, sampling of controls) adequately accounted for? | Yes | Yes | Yes | Yes | Yes |
| 4.7 Were relevant model performance measures adequately evaluated? | Yes | Yes | Yes | Yes | Yes |
| 4.8 Was model overfitting and optimism factored into model performance? | Yes | Yes | Yes | Yes | Yes |
| 4.9 Do the predictors and their assigned weights in the final model correspond to the results of the multivariate analysis? | Yes | Yes | Yes | Yes | Yes |
| **Risk of bias introduced by the analysis (low/high/Unclear)** | Low | Low | Low | Low | Low |
| **Step 4: General evaluation** | **-** | **-** | **-** | **-** | **-** |
| **Overall assessment of risk of bias** | Low | Low | Low | Low | Low |
| **General judgment of applicability** | Low | Low | Low | Low | Low |
|  |  |  |  |  |  |
|  |  |  |  |  |  |
| **Domain 1** | **Rajapaksha et al. (2021)** | **Rajput et al. (2021)** | **Rana et al. (2022)** | **Rao et al. (2021)** | **Reddy et al. (2021)** |
| **A. Risk of bias** | **16-07-22** | **16-07-22** | **16-07-22** | **16-07-22** | **16-07-22** |
| 1.1 Were appropriate data sources used, e.g. data from cohort studies, RCTs or nested case-controls? | Yes | Yes | Yes | Yes | Yes |
| 1.2 Were all inclusions and exclusions of participants appropriate? | Unclear | Unclear | Unclear | Unclear | Unclear |
| Risk of bias introduced by participant selection (low/high/unclear) | Low | Low | Low | Low | Low |
| **B. Applicability** |  |  |  |  |  |
| Concern that included participants and setting do not match review question (low/high/unclear) | Low | Low | Low | Low | Low |
| **Domain 2** |  |  |  |  |  |
| **A. Risk of bias - predictors** |  |  |  |  |  |
| 2.1 Were predictors defined and evaluated in a similar way for all participants? | Yes | Yes | Yes | Yes | Yes |
| 2.2 Were predictor evaluations performed without knowing the outcome data? | Yes | Yes | Yes | Yes | Yes |
| 2.3 Are all the predictors available at the time the model is intended to be used? | Yes | Yes | Yes | Yes | Yes |
| Risk of bias introduced by the predictors or their assessment (low/high/unclear) | Low | Low | Low | Low | Low |
| **B. Applicability** |  |  |  |  |  |
| Concern that the definition, evaluation, or timing of predictors in the model do not match the review question (low/high/unclear) | Low | Low | Low | Low | Low |
| **Domain 3** |  |  |  |  |  |
| **A. Risk of bias - outcome** |  |  |  |  |  |
| Was the result properly determined? | Yes | Yes | Yes | Yes | Yes |
| Was a standard or prespecified outcome definition used? | Yes | Yes | Yes | Yes | Yes |
| Were predictors excluded from the outcome definition? | Yes | Yes | Yes | Yes | Yes |
| Was the outcome similarly defined and determined for all participants? | Yes | Yes | Yes | Yes | Yes |
| Was the outcome determined without knowing the predictor information? | Yes | Yes | Yes | Yes | Yes |
| Was the time interval between the evaluation of the predictor and the determination of the result adequate? | Yes | Yes | Yes | Yes | Yes |
| Risk of bias introduced by the result or its determination (low/high/unclear) | Low | Low | Low | Low | Low |
| **B. Applicability** |  |  |  |  |  |
| If a composite outcome was used, please describe the relative frequency/distribution of each contributing outcome: | Yes | Yes | Yes | Yes | Yes |
| Concern that the outcome, its definition, timing, or determination does not match the review question (low/high/unclear) | Low | Low | Low | Low | Low |
| **Domain 4** |  |  |  |  |  |
| **A. Risk of bias** |  |  |  |  |  |
| Describe the number of participants, number of candidate predictors, outcome events, and events per candidate predictor: | Yes | Yes | Yes | Yes | Yes |
| Describe how the model was developed (for example, with respect to modeling technique (eg, survival or logistic modeling), selection of predictors, and definition of risk groups): | Yes | Yes | Yes | Yes | Yes |
| Describe if and how the model was validated, either internally (eg, bootstrapping, cross-validation, split random sample) or externally (eg, temporal validation, geographic validation, different setting, different type of participants): | Yes | Yes | Yes | Yes | Yes |
| Describe the performance measures of the model, e.g. (re)calibration, discrimination, (re)classification, net profit and if adjusted for optimism: | Yes | Yes | Yes | Yes | Yes |
| Describe the participants who were excluded from the analysis: | Yes | Yes | Yes | Yes | Yes |
| Describe missing data on predictors and outcomes, as well as methods used for missing data: | Yes | Yes | Yes | Yes | Yes |
| 4.1 Was there a reasonable number of participants with the result? | Yes | Yes | Yes | Yes | Yes |
| 4.2 Were continuous and categorical predictors handled correctly? | Yes | Yes | Yes | Yes | Yes |
| 4.3 Were all enrolled participants included in the analysis? | Yes | Yes | Yes | Yes | Yes |
| 4.4 Were participants with missing data treated appropriately? | No | No | No | No | No |
| 4.5 Was selection of predictors based on univariate analysis avoided? | Yes | Yes | Yes | Yes | Yes |
| 4.6 Were the complexities of the data (eg censoring, competing risks, sampling of controls) adequately accounted for? | Yes | Yes | Yes | Yes | Yes |
| 4.7 Were relevant model performance measures adequately evaluated? | Yes | Yes | Yes | Yes | Yes |
| 4.8 Was model overfitting and optimism factored into model performance? | Yes | Yes | Yes | Yes | Yes |
| 4.9 Do the predictors and their assigned weights in the final model correspond to the results of the multivariate analysis? | Yes | Yes | Yes | Yes | Yes |
| **Risk of bias introduced by the analysis (low/high/Unclear)** | Low | Low | Low | Low | Low |
| **Step 4: General evaluation** | **-** | **-** | **-** | **-** | **-** |
| **Overall assessment of risk of bias** | Low | Low | Low | Low | Low |
| **General judgment of applicability** | Low | Low | Low | Low | Low |
|  |  |  |  |  |  |
|  |  |  |  |  |  |
| **Domain 1** | **Reyné et al. (2022)** | **Robles-Fontán et al. (2020)** | **Rocha et al. (2021)** | **Rodríguez et al. (2021)** | **Romero-Brufau et al. (2021)** |
| **A. Risk of bias** | **16-07-22** | **16-07-22** | **16-07-22** | **16-07-22** | **16-07-22** |
| 1.1 Were appropriate data sources used, e.g. data from cohort studies, RCTs or nested case-controls? | Yes | Yes | Yes | Yes | Unclear |
| 1.2 Were all inclusions and exclusions of participants appropriate? | Unclear | Unclear | Unclear | Yes | Yes |
| Risk of bias introduced by participant selection (low/high/unclear) | Low | Low | Low | Low | Unclear |
| **B. Applicability** |  |  |  |  |  |
| Concern that included participants and setting do not match review question (low/high/unclear) | Low | Low | Low | Low | Low |
| **Domain 2** |  |  |  |  |  |
| **A. Risk of bias - predictors** |  |  |  |  |  |
| 2.1 Were predictors defined and evaluated in a similar way for all participants? | Yes | Yes | Yes | Yes | Yes |
| 2.2 Were predictor evaluations performed without knowing the outcome data? | Yes | Yes | Yes | Yes | Yes |
| 2.3 Are all the predictors available at the time the model is intended to be used? | Yes | Yes | Yes | Yes | Yes |
| Risk of bias introduced by the predictors or their assessment (low/high/unclear) | Low | Low | Low | Low | Low |
| **B. Applicability** |  |  |  |  |  |
| Concern that the definition, evaluation, or timing of predictors in the model do not match the review question (low/high/unclear) | Low | Low | Low | Low | Low |
| **Domain 3** |  |  |  |  |  |
| **A. Risk of bias - outcome** |  |  |  |  |  |
| Was the result properly determined? | Yes | Yes | Yes | Yes | Yes |
| Was a standard or prespecified outcome definition used? | Yes | Yes | Yes | Yes | Yes |
| Were predictors excluded from the outcome definition? | Yes | Yes | Yes | Yes | Yes |
| Was the outcome similarly defined and determined for all participants? | Yes | Yes | Yes | Yes | Yes |
| Was the outcome determined without knowing the predictor information? | Yes | Yes | Yes | Yes | Yes |
| Was the time interval between the evaluation of the predictor and the determination of the result adequate? | Yes | Yes | Yes | Yes | Yes |
| Risk of bias introduced by the result or its determination (low/high/unclear) | Low | Low | Low | Low | Low |
| **B. Applicability** |  |  |  |  |  |
| If a composite outcome was used, please describe the relative frequency/distribution of each contributing outcome: | Yes | Yes | Yes | Yes | Yes |
| Concern that the outcome, its definition, timing, or determination does not match the review question (low/high/unclear) | Low | Low | Low | Low | Low |
| **Domain 4** |  |  |  |  |  |
| **A. Risk of bias** |  |  |  |  |  |
| Describe the number of participants, number of candidate predictors, outcome events, and events per candidate predictor: | Yes | Yes | Yes | Yes | Yes |
| Describe how the model was developed (for example, with respect to modeling technique (eg, survival or logistic modeling), selection of predictors, and definition of risk groups): | Yes | Yes | Yes | Yes | Yes |
| Describe if and how the model was validated, either internally (eg, bootstrapping, cross-validation, split random sample) or externally (eg, temporal validation, geographic validation, different setting, different type of participants): | Yes | Yes | Yes | Yes | Yes |
| Describe the performance measures of the model, e.g. (re)calibration, discrimination, (re)classification, net profit and if adjusted for optimism: | Yes | Yes | Yes | Yes | Yes |
| Describe the participants who were excluded from the analysis: | Yes | Yes | Yes | Yes | Yes |
| Describe missing data on predictors and outcomes, as well as methods used for missing data: | Yes | Yes | Yes | Yes | Yes |
| 4.1 Was there a reasonable number of participants with the result? | Yes | Yes | Yes | Yes | Yes |
| 4.2 Were continuous and categorical predictors handled correctly? | Yes | Yes | Yes | Yes | Yes |
| 4.3 Were all enrolled participants included in the analysis? | Yes | Yes | Yes | Yes | Yes |
| 4.4 Were participants with missing data treated appropriately? | No | No | NA | NA | NA |
| 4.5 Was selection of predictors based on univariate analysis avoided? | Yes | Yes | Yes | Yes | Yes |
| 4.6 Were the complexities of the data (eg censoring, competing risks, sampling of controls) adequately accounted for? | Yes | Yes | Yes | Yes | Yes |
| 4.7 Were relevant model performance measures adequately evaluated? | Yes | Yes | Yes | Yes | Yes |
| 4.8 Was model overfitting and optimism factored into model performance? | Yes | Yes | Yes | Yes | Yes |
| 4.9 Do the predictors and their assigned weights in the final model correspond to the results of the multivariate analysis? | Yes | Yes | Yes | Yes | Yes |
| **Risk of bias introduced by the analysis (low/high/Unclear)** | Low | Low | Low | Low | Low |
| **Step 4: General evaluation** | **-** | **-** | **-** | **-** | The parameters for the population are taken into account, but the source of this was not reliably defined since they only mention that the results of a clinical trial were taken into account |
| **Overall assessment of risk of bias** | Low | Low | Low | Low | High |
| **General judgment of applicability** | Low | Low | Low | Low | Low |
|  |  |  |  |  |  |
|  |  |  |  |  |  |
| **Domain 1** | **Roy et al. (2021) (1)** | **Roy et al. (2021)** | **Sadarangani et al. (2021)** | **Sah et al. (2021)** |  |
| **A. Risk of bias** | **16-07-22** | **16-07-22** | **16-07-22** | **16-07-22** |  |
| 1.1 Were appropriate data sources used, e.g. data from cohort studies, RCTs or nested case-controls? | Unclear | Unclear | Unclear | Unclear |  |
| 1.2 Were all inclusions and exclusions of participants appropriate? | Yes | No | No | Unclear |  |
| Risk of bias introduced by participant selection (low/high/unclear) | Unclear | High | High | Low |  |
| **B. Applicability** |  |  |  |  |  |
| Concern that included participants and setting do not match review question (low/high/unclear) | Low | Unclear | Unclear | Low |  |
| **Domain 2** |  |  |  |  |  |
| **A. Risk of bias - predictors** |  |  |  |  |  |
| 2.1 Were predictors defined and evaluated in a similar way for all participants? | Yes | Unclear | Unclear | Yes |  |
| 2.2 Were predictor evaluations performed without knowing the outcome data? | Yes | Unclear | Unclear | Yes |  |
| 2.3 Are all the predictors available at the time the model is intended to be used? | Yes | Yes | Yes | Yes |  |
| Risk of bias introduced by the predictors or their assessment (low/high/unclear) | Low | High | High | Low |  |
| **B. Applicability** |  |  |  |  |  |
| Concern that the definition, evaluation, or timing of predictors in the model do not match the review question (low/high/unclear) | Low | Unclear | Unclear | Low |  |
| **Domain 3** |  |  |  |  |  |
| **A. Risk of bias - outcome** |  |  |  |  |  |
| Was the result properly determined? | Yes | Yes | Yes | Yes |  |
| Was a standard or prespecified outcome definition used? | Yes | Unclear | Unclear | Yes |  |
| Were predictors excluded from the outcome definition? | Yes | Yes | Yes | Yes |  |
| Was the outcome similarly defined and determined for all participants? | Yes | Yes | Yes | Yes |  |
| Was the outcome determined without knowing the predictor information? | Yes | Yes | Yes | Yes |  |
| Was the time interval between the evaluation of the predictor and the determination of the result adequate? | Yes | Yes | Yes | Yes |  |
| Risk of bias introduced by the result or its determination (low/high/unclear) | Low | High | High | Low |  |
| **B. Applicability** |  |  |  |  |  |
| If a composite outcome was used, please describe the relative frequency/distribution of each contributing outcome: | Yes | Yes | Yes | Yes |  |
| Concern that the outcome, its definition, timing, or determination does not match the review question (low/high/unclear) | Low | Low | Low | Low |  |
| **Domain 4** |  |  |  |  |  |
| **A. Risk of bias** |  |  |  |  |  |
| Describe the number of participants, number of candidate predictors, outcome events, and events per candidate predictor: | Yes | Unclear | Unclear | Yes |  |
| Describe how the model was developed (for example, with respect to modeling technique (eg, survival or logistic modeling), selection of predictors, and definition of risk groups): | Yes | Yes | Yes | Yes |  |
| Describe if and how the model was validated, either internally (eg, bootstrapping, cross-validation, split random sample) or externally (eg, temporal validation, geographic validation, different setting, different type of participants): | Yes | Unclear | Unclear | Yes |  |
| Describe the performance measures of the model, e.g. (re)calibration, discrimination, (re)classification, net profit and if adjusted for optimism: | Yes | Unclear | Unclear | Yes |  |
| Describe the participants who were excluded from the analysis: | Yes | NA | NA | Yes |  |
| Describe missing data on predictors and outcomes, as well as methods used for missing data: | Yes | Unclear | Unclear | Yes |  |
| 4.1 Was there a reasonable number of participants with the result? | Yes | Unclear | Unclear | Yes |  |
| 4.2 Were continuous and categorical predictors handled correctly? | Yes | Unclear | Unclear | Yes |  |
| 4.3 Were all enrolled participants included in the analysis? | Yes | Unclear | Unclear | Yes |  |
| 4.4 Were participants with missing data treated appropriately? | NA | Unclear | Unclear | NA |  |
| 4.5 Was selection of predictors based on univariate analysis avoided? | Yes | Unclear | Unclear | Yes |  |
| 4.6 Were the complexities of the data (eg censoring, competing risks, sampling of controls) adequately accounted for? | Yes | Unclear | Unclear | Yes |  |
| 4.7 Were relevant model performance measures adequately evaluated? | Yes | Yes | Yes | Yes |  |
| 4.8 Was model overfitting and optimism factored into model performance? | Yes | Yes | Yes | Yes |  |
| 4.9 Do the predictors and their assigned weights in the final model correspond to the results of the multivariate analysis? | Yes | Unclear | Unclear | Yes |  |
| **Risk of bias introduced by the analysis (low/high/Unclear)** | Low | High | High | Low |  |
| **Step 4: General evaluation** | The parameters for the population are taken into account, but the source of the population was not defined | There are no methods described | There is no information regarding the population used, nor are the methodological details expanded | **-** |  |
| **Overall assessment of risk of bias** | High | High | High | Low |  |
| **General judgment of applicability** | Low | High | High | Low |  |
|  |  |  |  |  |  |
|  |  |  |  |  |  |
| **Domain 1** | **Saldaña et al. (2022)** | **Sandmann et al. (2021)** | **Sanz-Leon et al. (2022)** | **Savinkina et al. (2022)** | **Scarabaggio et al. (2021)** |
| **A. Risk of bias** | **16-07-22** | **16-07-22** | **16-07-22** | **16-07-22** | **16-07-22** |
| 1.1 Were appropriate data sources used, e.g. data from cohort studies, RCTs or nested case-controls? | Yes | Yes | Yes | Yes | Yes |
| 1.2 Were all inclusions and exclusions of participants appropriate? | Unclear | Unclear | Unclear | Unclear | Unclear |
| Risk of bias introduced by participant selection (low/high/unclear) | Low | Low | Low | Low | Low |
| **B. Applicability** |  |  |  |  |  |
| Concern that included participants and setting do not match review question (low/high/unclear) | Low | Low | Low | Low | Low |
| **Domain 2** |  |  |  |  |  |
| **A. Risk of bias - predictors** |  |  |  |  |  |
| 2.1 Were predictors defined and evaluated in a similar way for all participants? | Yes | Yes | Yes | Yes | Yes |
| 2.2 Were predictor evaluations performed without knowing the outcome data? | Yes | Yes | Yes | Yes | Yes |
| 2.3 Are all the predictors available at the time the model is intended to be used? | Yes | Yes | Yes | Yes | Yes |
| Risk of bias introduced by the predictors or their assessment (low/high/unclear) | Low | Low | Low | Low | Low |
| **B. Applicability** |  |  |  |  |  |
| Concern that the definition, evaluation, or timing of predictors in the model do not match the review question (low/high/unclear) | Low | Low | Low | Low | Low |
| **Domain 3** |  |  |  |  |  |
| **A. Risk of bias - outcome** |  |  |  |  |  |
| Was the result properly determined? | Yes | Yes | Yes | Yes | Yes |
| Was a standard or prespecified outcome definition used? | Yes | Yes | Yes | Yes | Yes |
| Were predictors excluded from the outcome definition? | Yes | Yes | Yes | Yes | Yes |
| Was the outcome similarly defined and determined for all participants? | Yes | Yes | Yes | Yes | Yes |
| Was the outcome determined without knowing the predictor information? | Yes | Yes | Yes | Yes | Yes |
| Was the time interval between the evaluation of the predictor and the determination of the result adequate? | Yes | Yes | Yes | Yes | Yes |
| Risk of bias introduced by the result or its determination (low/high/unclear) | Low | Low | Low | Low | Low |
| **B. Applicability** |  |  |  |  |  |
| If a composite outcome was used, please describe the relative frequency/distribution of each contributing outcome: | Yes | Yes | Yes | Yes | Yes |
| Concern that the outcome, its definition, timing, or determination does not match the review question (low/high/unclear) | Low | Low | Low | Low | Low |
| **Domain 4** |  |  |  |  |  |
| **A. Risk of bias** |  |  |  |  |  |
| Describe the number of participants, number of candidate predictors, outcome events, and events per candidate predictor: | Yes | Yes | Yes | Yes | Yes |
| Describe how the model was developed (for example, with respect to modeling technique (eg, survival or logistic modeling), selection of predictors, and definition of risk groups): | Yes | Yes | Yes | Yes | Yes |
| Describe if and how the model was validated, either internally (eg, bootstrapping, cross-validation, split random sample) or externally (eg, temporal validation, geographic validation, different setting, different type of participants): | Yes | Yes | Yes | Yes | Yes |
| Describe the performance measures of the model, e.g. (re)calibration, discrimination, (re)classification, net profit and if adjusted for optimism: | Yes | Yes | Yes | Yes | Yes |
| Describe the participants who were excluded from the analysis: | Yes | Yes | Yes | Yes | Yes |
| Describe missing data on predictors and outcomes, as well as methods used for missing data: | Yes | Yes | Yes | Yes | Yes |
| 4.1 Was there a reasonable number of participants with the result? | Yes | Yes | Yes | Yes | Yes |
| 4.2 Were continuous and categorical predictors handled correctly? | Yes | Yes | Yes | Yes | Yes |
| 4.3 Were all enrolled participants included in the analysis? | Yes | Yes | Yes | Yes | Yes |
| 4.4 Were participants with missing data treated appropriately? | NA | NA | NA | NA | NA |
| 4.5 Was selection of predictors based on univariate analysis avoided? | Yes | Yes | Yes | Yes | Yes |
| 4.6 Were the complexities of the data (eg censoring, competing risks, sampling of controls) adequately accounted for? | Yes | Yes | Yes | Yes | Yes |
| 4.7 Were relevant model performance measures adequately evaluated? | Yes | Yes | Yes | Yes | Yes |
| 4.8 Was model overfitting and optimism factored into model performance? | Yes | Yes | Yes | Yes | Yes |
| 4.9 Do the predictors and their assigned weights in the final model correspond to the results of the multivariate analysis? | Yes | Yes | Yes | Yes | Yes |
| **Risk of bias introduced by the analysis (low/high/Unclear)** | Low | Low | Low | Low | Low |
| **Step 4: General evaluation** | **-** | **-** | **-** | **-** | **-** |
| **Overall assessment of risk of bias** | Low | Low | Low | Low | Low |
| **General judgment of applicability** | Low | Low | Low | Low | Low |
|  |  |  |  |  |  |
|  |  |  |  |  |  |
| **Domain 1** | **Schneider et al. (2022)** | **Schulenburg et al. (2022)** | **Shadi et al. (2022)** | **Shen et al. (2021)** | **Shim (2021) (1)** |
| **A. Risk of bias** | **16-07-22** | **16-07-22** | **16-07-22** | **16-07-22** | **16-07-22** |
| 1.1 Were appropriate data sources used, e.g. data from cohort studies, RCTs or nested case-controls? | Unclear | Unclear | Unclear | Yes | Yes |
| 1.2 Were all inclusions and exclusions of participants appropriate? | Unclear | No | No | Yes | Yes |
| Risk of bias introduced by participant selection (low/high/unclear) | Unclear | High | High | Low | Low |
| **B. Applicability** |  |  |  |  |  |
| Concern that included participants and setting do not match review question (low/high/unclear) | Unclear | Low | Low | Low | Low |
| **Domain 2** |  |  |  |  |  |
| **A. Risk of bias - predictors** |  |  |  |  |  |
| 2.1 Were predictors defined and evaluated in a similar way for all participants? | Yes | Yes | Yes | Yes | Yes |
| 2.2 Were predictor evaluations performed without knowing the outcome data? | Yes | Yes | Yes | Yes | Yes |
| 2.3 Are all the predictors available at the time the model is intended to be used? | Yes | Yes | Yes | Yes | Yes |
| Risk of bias introduced by the predictors or their assessment (low/high/unclear) | Low | Low | Low | Low | Low |
| **B. Applicability** |  |  |  |  |  |
| Concern that the definition, evaluation, or timing of predictors in the model do not match the review question (low/high/unclear) | Low | Low | Low | Low | Low |
| **Domain 3** |  |  |  |  |  |
| **A. Risk of bias - outcome** |  |  |  |  |  |
| Was the result properly determined? | Yes | Yes | Yes | Yes | Yes |
| Was a standard or prespecified outcome definition used? | Yes | Yes | Yes | Yes | Yes |
| Were predictors excluded from the outcome definition? | Yes | Yes | Yes | Yes | Yes |
| Was the outcome similarly defined and determined for all participants? | Yes | Yes | Yes | Yes | Yes |
| Was the outcome determined without knowing the predictor information? | Yes | Yes | Yes | Yes | Yes |
| Was the time interval between the evaluation of the predictor and the determination of the result adequate? | Yes | Yes | Yes | Yes | Yes |
| Risk of bias introduced by the result or its determination (low/high/unclear) | Low | Low | Low | Low | Low |
| **B. Applicability** |  |  |  |  |  |
| If a composite outcome was used, please describe the relative frequency/distribution of each contributing outcome: | Yes | Yes | Yes | Yes | Yes |
| Concern that the outcome, its definition, timing, or determination does not match the review question (low/high/unclear) | Low | Low | Low | Low | Low |
| **Domain 4** |  |  |  |  |  |
| **A. Risk of bias** |  |  |  |  |  |
| Describe the number of participants, number of candidate predictors, outcome events, and events per candidate predictor: | Yes | Yes | Yes | Yes | Yes |
| Describe how the model was developed (for example, with respect to modeling technique (eg, survival or logistic modeling), selection of predictors, and definition of risk groups): | Yes | Yes | Yes | Yes | Yes |
| Describe if and how the model was validated, either internally (eg, bootstrapping, cross-validation, split random sample) or externally (eg, temporal validation, geographic validation, different setting, different type of participants): | Yes | Yes | Yes | Yes | Yes |
| Describe the performance measures of the model, e.g. (re)calibration, discrimination, (re)classification, net profit and if adjusted for optimism: | Yes | Yes | Yes | Yes | Yes |
| Describe the participants who were excluded from the analysis: | Yes | Yes | Yes | Yes | Yes |
| Describe missing data on predictors and outcomes, as well as methods used for missing data: | Yes | Yes | Yes | Yes | Yes |
| 4.1 Was there a reasonable number of participants with the result? | Yes | Yes | Yes | Yes | Yes |
| 4.2 Were continuous and categorical predictors handled correctly? | Yes | Yes | Yes | Yes | Yes |
| 4.3 Were all enrolled participants included in the analysis? | Yes | Yes | Yes | Yes | Yes |
| 4.4 Were participants with missing data treated appropriately? | NA | NA | NA | NA | NA |
| 4.5 Was selection of predictors based on univariate analysis avoided? | Yes | Yes | Yes | Yes | Yes |
| 4.6 Were the complexities of the data (eg censoring, competing risks, sampling of controls) adequately accounted for? | Yes | Yes | Yes | Yes | Yes |
| 4.7 Were relevant model performance measures adequately evaluated? | Yes | Yes | Yes | Yes | Yes |
| 4.8 Was model overfitting and optimism factored into model performance? | Yes | Yes | Yes | Yes | Yes |
| 4.9 Do the predictors and their assigned weights in the final model correspond to the results of the multivariate analysis? | Yes | Yes | Yes | Yes | Yes |
| **Risk of bias introduced by the analysis (low/high/Unclear)** | Low | Low | Low | Low | Low |
| **Step 4: General evaluation** | There is not enough information related to the chosen population, so there is a risk of introducing bias in the selection | The details of the included population are not clear | The details of the included population are not clear | **-** | **-** |
| **Overall assessment of risk of bias** | High | High | High | Low | Low |
| **General judgment of applicability** | Low | Low | Low | Low | Low |
|  |  |  |  |  |  |
|  |  |  |  |  |  |
| **Domain 1** | **Shim (2021) (2)** | **Silva et al. (2021)** | **Castro e Silva et al. (2022)** | **Sivadas et al. (2021)** | **Somekh et al. (2022)** |
| **A. Risk of bias** | **16-07-22** | **16-07-22** | **17-07-22** | **17-07-22** | **17-07-22** |
| 1.1 Were appropriate data sources used, e.g. data from cohort studies, RCTs or nested case-controls? | Yes | Unclear | Unclear | Unclear | Yes |
| 1.2 Were all inclusions and exclusions of participants appropriate? | Unclear | No | No | No | Unclear |
| Risk of bias introduced by participant selection (low/high/unclear) | Low | High | High | High | Low |
| **B. Applicability** |  |  |  |  |  |
| Concern that included participants and setting do not match review question (low/high/unclear) | Low | Low | Low | Unclear | Low |
| **Domain 2** |  |  |  |  |  |
| **A. Risk of bias - predictors** |  |  |  |  |  |
| 2.1 Were predictors defined and evaluated in a similar way for all participants? | Yes | Yes | Yes | Unclear | Yes |
| 2.2 Were predictor evaluations performed without knowing the outcome data? | Yes | Yes | Yes | Unclear | Yes |
| 2.3 Are all the predictors available at the time the model is intended to be used? | Yes | Yes | Yes | Yes | Yes |
| Risk of bias introduced by the predictors or their assessment (low/high/unclear) | Low | Low | Low | High | Low |
| **B. Applicability** |  |  |  |  |  |
| Concern that the definition, evaluation, or timing of predictors in the model do not match the review question (low/high/unclear) | Low | Low | Low | Unclear | Low |
| **Domain 3** |  |  |  |  |  |
| **A. Risk of bias - outcome** |  |  |  |  |  |
| Was the result properly determined? | Yes | Yes | Yes | Yes | Yes |
| Was a standard or prespecified outcome definition used? | Yes | Yes | Yes | Unclear | Yes |
| Were predictors excluded from the outcome definition? | Yes | Yes | Yes | Yes | Yes |
| Was the outcome similarly defined and determined for all participants? | Yes | Yes | Yes | Yes | Yes |
| Was the outcome determined without knowing the predictor information? | Yes | Yes | Yes | Yes | Yes |
| Was the time interval between the evaluation of the predictor and the determination of the result adequate? | Yes | Yes | Yes | Yes | Yes |
| Risk of bias introduced by the result or its determination (low/high/unclear) | Low | Low | Low | High | Low |
| **B. Applicability** |  |  |  |  |  |
| If a composite outcome was used, please describe the relative frequency/distribution of each contributing outcome: | Yes | Yes | Yes | Yes | Yes |
| Concern that the outcome, its definition, timing, or determination does not match the review question (low/high/unclear) | Low | Low | Low | Low | Low |
| **Domain 4** |  |  |  |  |  |
| **A. Risk of bias** |  |  |  |  |  |
| Describe the number of participants, number of candidate predictors, outcome events, and events per candidate predictor: | Yes | Yes | Yes | Unclear | Yes |
| Describe how the model was developed (for example, with respect to modeling technique (eg, survival or logistic modeling), selection of predictors, and definition of risk groups): | Yes | Yes | Yes | Yes | Yes |
| Describe if and how the model was validated, either internally (eg, bootstrapping, cross-validation, split random sample) or externally (eg, temporal validation, geographic validation, different setting, different type of participants): | Yes | Yes | Yes | Unclear | Yes |
| Describe the performance measures of the model, e.g. (re)calibration, discrimination, (re)classification, net profit and if adjusted for optimism: | Yes | Yes | Yes | Unclear | Yes |
| Describe the participants who were excluded from the analysis: | Yes | Yes | Yes | NA | Yes |
| Describe missing data on predictors and outcomes, as well as methods used for missing data: | Yes | Yes | Yes | Unclear | Yes |
| 4.1 Was there a reasonable number of participants with the result? | Yes | Yes | Yes | Unclear | Yes |
| 4.2 Were continuous and categorical predictors handled correctly? | Yes | Yes | Yes | Unclear | Yes |
| 4.3 Were all enrolled participants included in the analysis? | Yes | Yes | Yes | Unclear | Yes |
| 4.4 Were participants with missing data treated appropriately? | NA | NA | NA | Unclear | NA |
| 4.5 Was selection of predictors based on univariate analysis avoided? | Yes | Yes | Yes | Unclear | Yes |
| 4.6 Were the complexities of the data (eg censoring, competing risks, sampling of controls) adequately accounted for? | Yes | Yes | Yes | Unclear | Yes |
| 4.7 Were relevant model performance measures adequately evaluated? | Yes | Yes | Yes | Yes | Yes |
| 4.8 Was model overfitting and optimism factored into model performance? | Yes | Yes | Yes | Yes | Yes |
| 4.9 Do the predictors and their assigned weights in the final model correspond to the results of the multivariate analysis? | Yes | Yes | Yes | Unclear | Yes |
| **Risk of bias introduced by the analysis (low/high/Unclear)** | Low | Low | Low | High | Low |
| **Step 4: General evaluation** | **-** | The details of the included population are not clear | The details of the included population are not clear | There is no information regarding the population used, nor are the methodological details expanded | **-** |
| **Overall assessment of risk of bias** | Low | High | High | High | Low |
| **General judgment of applicability** | Low | Low | Low | High | Low |
|  |  |  |  |  |  |
|  |  |  |  |  |  |
| **Domain 1** | **Sonabend et al. (2021)** | **Song et al. (2021) (1)** | **Song et al. (2021) (2)** | **Stanojevic et al. (2021)** | **Stevenson et al. (2021)** |
| **A. Risk of bias** | **17-07-22** | **17-07-22** | **17-07-22** | **17-07-22** | **17-07-22** |
| 1.1 Were appropriate data sources used, e.g. data from cohort studies, RCTs or nested case-controls? | Yes | Unclear | Yes | Unclear | Yes |
| 1.2 Were all inclusions and exclusions of participants appropriate? | Unclear | No | Unclear | Unclear | Unclear |
| Risk of bias introduced by participant selection (low/high/unclear) | Low | High | Low | Unclear | Low |
| **B. Applicability** |  |  |  |  |  |
| Concern that included participants and setting do not match review question (low/high/unclear) | Low | Low | Low | Low | Low |
| **Domain 2** |  |  |  |  |  |
| **A. Risk of bias - predictors** |  |  |  |  |  |
| 2.1 Were predictors defined and evaluated in a similar way for all participants? | Yes | Yes | Yes | Yes | Yes |
| 2.2 Were predictor evaluations performed without knowing the outcome data? | Yes | Yes | Yes | Yes | Yes |
| 2.3 Are all the predictors available at the time the model is intended to be used? | Yes | Yes | Yes | Yes | Yes |
| Risk of bias introduced by the predictors or their assessment (low/high/unclear) | Low | Low | Low | Low | Low |
| **B. Applicability** |  |  |  |  |  |
| Concern that the definition, evaluation, or timing of predictors in the model do not match the review question (low/high/unclear) | Low | Low | Low | Low | Low |
| **Domain 3** |  |  |  |  |  |
| **A. Risk of bias - outcome** |  |  |  |  |  |
| Was the result properly determined? | Yes | Yes | Yes | Yes | Yes |
| Was a standard or prespecified outcome definition used? | Yes | Yes | Yes | Yes | Yes |
| Were predictors excluded from the outcome definition? | Yes | Yes | Yes | Yes | Yes |
| Was the outcome similarly defined and determined for all participants? | Yes | Yes | Yes | Yes | Yes |
| Was the outcome determined without knowing the predictor information? | Yes | Yes | Yes | Yes | Yes |
| Was the time interval between the evaluation of the predictor and the determination of the result adequate? | Yes | Yes | Yes | Yes | Yes |
| Risk of bias introduced by the result or its determination (low/high/unclear) | Low | Low | Low | Low | Low |
| **B. Applicability** |  |  |  |  |  |
| If a composite outcome was used, please describe the relative frequency/distribution of each contributing outcome: | Yes | Yes | Yes | Yes | Yes |
| Concern that the outcome, its definition, timing, or determination does not match the review question (low/high/unclear) | Low | Low | Low | Low | Low |
| **Domain 4** |  |  |  |  |  |
| **A. Risk of bias** |  |  |  |  |  |
| Describe the number of participants, number of candidate predictors, outcome events, and events per candidate predictor: | Yes | Yes | Yes | Yes | Yes |
| Describe how the model was developed (for example, with respect to modeling technique (eg, survival or logistic modeling), selection of predictors, and definition of risk groups): | Yes | Yes | Yes | Yes | Yes |
| Describe if and how the model was validated, either internally (eg, bootstrapping, cross-validation, split random sample) or externally (eg, temporal validation, geographic validation, different setting, different type of participants): | Yes | Yes | Yes | Yes | Yes |
| Describe the performance measures of the model, e.g. (re)calibration, discrimination, (re)classification, net profit and if adjusted for optimism: | Yes | Yes | Yes | Yes | Yes |
| Describe the participants who were excluded from the analysis: | Yes | Yes | Yes | Yes | Yes |
| Describe missing data on predictors and outcomes, as well as methods used for missing data: | Yes | Yes | Yes | Yes | Yes |
| 4.1 Was there a reasonable number of participants with the result? | Yes | Yes | Yes | Yes | Yes |
| 4.2 Were continuous and categorical predictors handled correctly? | Yes | Yes | Yes | Yes | Yes |
| 4.3 Were all enrolled participants included in the analysis? | Yes | Yes | Yes | Yes | Yes |
| 4.4 Were participants with missing data treated appropriately? | NA | NA | NA | NA | NA |
| 4.5 Was selection of predictors based on univariate analysis avoided? | Yes | Yes | Yes | Yes | Yes |
| 4.6 Were the complexities of the data (eg censoring, competing risks, sampling of controls) adequately accounted for? | Yes | Yes | Yes | Yes | Yes |
| 4.7 Were relevant model performance measures adequately evaluated? | Yes | Yes | Yes | Yes | Yes |
| 4.8 Was model overfitting and optimism factored into model performance? | Yes | Yes | Yes | Yes | Yes |
| 4.9 Do the predictors and their assigned weights in the final model correspond to the results of the multivariate analysis? | Yes | Yes | Yes | Yes | Yes |
| **Risk of bias introduced by the analysis (low/high/Unclear)** | Low | Low | Low | Low | Low |
| **Step 4: General evaluation** | **-** | The details of the included population are not clear | - | There is not enough information related to the population | **-** |
| **Overall assessment of risk of bias** | Low | High | Low | Unclear | Low |
| **General judgment of applicability** | Low | Low | Low | Low | Low |
|  |  |  |  |  |  |
|  |  |  |  |  |  |
| **Domain 1** | **Steyn et al. (2022)** | **Stollenwerk et al. (2021)** | **Storlie et al. (2021)** | **Sulis et al. (2021)** | **Sun et al. (2021)** |
| **A. Risk of bias** | **17-07-22** | **17-07-22** | **17-07-22** | **17-07-22** | **17-07-22** |
| 1.1 Were appropriate data sources used, e.g. data from cohort studies, RCTs or nested case-controls? | Unclear | Yes | Unclear | Unclear | Yes |
| 1.2 Were all inclusions and exclusions of participants appropriate? | Unclear | Unclear | No | No | Unclear |
| Risk of bias introduced by participant selection (low/high/unclear) | Unclear | Low | High | High | Low |
| **B. Applicability** |  |  |  |  |  |
| Concern that included participants and setting do not match review question (low/high/unclear) | Low | Low | Unclear | Unclear | Low |
| **Domain 2** |  |  |  |  |  |
| **A. Risk of bias - predictors** |  |  |  |  |  |
| 2.1 Were predictors defined and evaluated in a similar way for all participants? | Yes | Yes | Unclear | Unclear | Yes |
| 2.2 Were predictor evaluations performed without knowing the outcome data? | Yes | Yes | Unclear | Unclear | Yes |
| 2.3 Are all the predictors available at the time the model is intended to be used? | Yes | Yes | Yes | Yes | Yes |
| Risk of bias introduced by the predictors or their assessment (low/high/unclear) | Low | Low | High | High | Low |
| **B. Applicability** |  |  |  |  |  |
| Concern that the definition, evaluation, or timing of predictors in the model do not match the review question (low/high/unclear) | Low | Low | Unclear | Unclear | Low |
| **Domain 3** |  |  |  |  |  |
| **A. Risk of bias - outcome** |  |  |  |  |  |
| Was the result properly determined? | Yes | Yes | Yes | Yes | Yes |
| Was a standard or prespecified outcome definition used? | Yes | Yes | Unclear | Unclear | Yes |
| Were predictors excluded from the outcome definition? | Yes | Yes | Yes | Yes | Yes |
| Was the outcome similarly defined and determined for all participants? | Yes | Yes | Yes | Yes | Yes |
| Was the outcome determined without knowing the predictor information? | Yes | Yes | Yes | Yes | Yes |
| Was the time interval between the evaluation of the predictor and the determination of the result adequate? | Yes | Yes | Yes | Yes | Yes |
| Risk of bias introduced by the result or its determination (low/high/unclear) | Low | Low | High | High | Low |
| **B. Applicability** |  |  |  |  |  |
| If a composite outcome was used, please describe the relative frequency/distribution of each contributing outcome: | Yes | Yes | Yes | Yes | Yes |
| Concern that the outcome, its definition, timing, or determination does not match the review question (low/high/unclear) | Low | Low | Low | Low | Low |
| **Domain 4** |  |  |  |  |  |
| **A. Risk of bias** |  |  |  |  |  |
| Describe the number of participants, number of candidate predictors, outcome events, and events per candidate predictor: | Yes | Yes | Unclear | Unclear | Yes |
| Describe how the model was developed (for example, with respect to modeling technique (eg, survival or logistic modeling), selection of predictors, and definition of risk groups): | Yes | Yes | Yes | Yes | Yes |
| Describe if and how the model was validated, either internally (eg, bootstrapping, cross-validation, split random sample) or externally (eg, temporal validation, geographic validation, different setting, different type of participants): | Yes | Yes | Unclear | Unclear | Yes |
| Describe the performance measures of the model, e.g. (re)calibration, discrimination, (re)classification, net profit and if adjusted for optimism: | Yes | Yes | Unclear | Unclear | Yes |
| Describe the participants who were excluded from the analysis: | Yes | Yes | NA | NA | Yes |
| Describe missing data on predictors and outcomes, as well as methods used for missing data: | Yes | Yes | Unclear | Unclear | Yes |
| 4.1 Was there a reasonable number of participants with the result? | Yes | Yes | Unclear | Unclear | Yes |
| 4.2 Were continuous and categorical predictors handled correctly? | Yes | Yes | Unclear | Unclear | Yes |
| 4.3 Were all enrolled participants included in the analysis? | Yes | Yes | Unclear | Unclear | Yes |
| 4.4 Were participants with missing data treated appropriately? | NA | NA | Unclear | Unclear | NA |
| 4.5 Was selection of predictors based on univariate analysis avoided? | Yes | Yes | Unclear | Unclear | Yes |
| 4.6 Were the complexities of the data (eg censoring, competing risks, sampling of controls) adequately accounted for? | Yes | Yes | Unclear | Unclear | Yes |
| 4.7 Were relevant model performance measures adequately evaluated? | Yes | Yes | Yes | Yes | Yes |
| 4.8 Was model overfitting and optimism factored into model performance? | Yes | Yes | Yes | Yes | Yes |
| 4.9 Do the predictors and their assigned weights in the final model correspond to the results of the multivariate analysis? | Yes | Yes | Unclear | Unclear | Yes |
| **Risk of bias introduced by the analysis (low/high/Unclear)** | Low | Low | High | High | Low |
| **Step 4: General evaluation** | There is not enough information related to the chosen population, so there is a risk of introducing bias in the selection | - | There is no information regarding the population used, nor are the methodological details expanded | There is no information regarding the population used, nor are the methodological details expanded | **-** |
| **Overall assessment of risk of bias** | Unclear | Low | High | High | Low |
| **General judgment of applicability** | Low | Low | High | High | Low |
|  |  |  |  |  |  |
|  |  |  |  |  |  |
| **Domain 1** | **Sunohara et al. (2021)** | **Suphanchaimat et al. (2021) (1)** | **Suphanchaimat et al. (2021) (2)** | **Suphanchaimat et al. (2022)** | **Swan et al. (2020)** |
| **A. Risk of bias** | **17-07-22** | **17-07-22** | **18-07-22** | **18-07-22** | **18-07-22** |
| 1.1 Were appropriate data sources used, e.g. data from cohort studies, RCTs or nested case-controls? | Unclear | Unclear | Yes | Yes | Yes |
| 1.2 Were all inclusions and exclusions of participants appropriate? | No | No | Unclear | Unclear | Unclear |
| Risk of bias introduced by participant selection (low/high/unclear) | High | High | Low | Low | Low |
| **B. Applicability** |  |  |  |  |  |
| Concern that included participants and setting do not match review question (low/high/unclear) | Low | Unclear | Low | Low | Low |
| **Domain 2** |  |  |  |  |  |
| **A. Risk of bias - predictors** |  |  |  |  |  |
| 2.1 Were predictors defined and evaluated in a similar way for all participants? | Yes | Unclear | Yes | Yes | Yes |
| 2.2 Were predictor evaluations performed without knowing the outcome data? | Yes | Unclear | Yes | Yes | Yes |
| 2.3 Are all the predictors available at the time the model is intended to be used? | Yes | Yes | Yes | Yes | Yes |
| Risk of bias introduced by the predictors or their assessment (low/high/unclear) | Low | High | Low | Low | Low |
| **B. Applicability** |  |  |  |  |  |
| Concern that the definition, evaluation, or timing of predictors in the model do not match the review question (low/high/unclear) | Low | Unclear | Low | Low | Low |
| **Domain 3** |  |  |  |  |  |
| **A. Risk of bias - outcome** |  |  |  |  |  |
| Was the result properly determined? | Yes | Yes | Yes | Yes | Yes |
| Was a standard or prespecified outcome definition used? | Yes | Unclear | Yes | Yes | Yes |
| Were predictors excluded from the outcome definition? | Yes | Yes | Yes | Yes | Yes |
| Was the outcome similarly defined and determined for all participants? | Yes | Yes | Yes | Yes | Yes |
| Was the outcome determined without knowing the predictor information? | Yes | Yes | Yes | Yes | Yes |
| Was the time interval between the evaluation of the predictor and the determination of the result adequate? | Yes | Yes | Yes | Yes | Yes |
| Risk of bias introduced by the result or its determination (low/high/unclear) | Low | High | Low | Low | Low |
| **B. Applicability** |  |  |  |  |  |
| If a composite outcome was used, please describe the relative frequency/distribution of each contributing outcome: | Yes | Yes | Yes | Yes | Yes |
| Concern that the outcome, its definition, timing, or determination does not match the review question (low/high/unclear) | Low | Low | Low | Low | Low |
| **Domain 4** |  |  |  |  |  |
| **A. Risk of bias** |  |  |  |  |  |
| Describe the number of participants, number of candidate predictors, outcome events, and events per candidate predictor: | Yes | Unclear | Yes | Yes | Yes |
| Describe how the model was developed (for example, with respect to modeling technique (eg, survival or logistic modeling), selection of predictors, and definition of risk groups): | Yes | Yes | Yes | Yes | Yes |
| Describe if and how the model was validated, either internally (eg, bootstrapping, cross-validation, split random sample) or externally (eg, temporal validation, geographic validation, different setting, different type of participants): | Yes | Unclear | Yes | Yes | Yes |
| Describe the performance measures of the model, e.g. (re)calibration, discrimination, (re)classification, net profit and if adjusted for optimism: | Yes | Unclear | Yes | Yes | Yes |
| Describe the participants who were excluded from the analysis: | Yes | NA | Yes | Yes | Yes |
| Describe missing data on predictors and outcomes, as well as methods used for missing data: | Yes | Unclear | Yes | Yes | Yes |
| 4.1 Was there a reasonable number of participants with the result? | Yes | Unclear | Yes | Yes | Yes |
| 4.2 Were continuous and categorical predictors handled correctly? | Yes | Unclear | Yes | Yes | Yes |
| 4.3 Were all enrolled participants included in the analysis? | Yes | Unclear | Yes | Yes | Yes |
| 4.4 Were participants with missing data treated appropriately? | NA | Unclear | NA | NA | NA |
| 4.5 Was selection of predictors based on univariate analysis avoided? | Yes | Unclear | Yes | Yes | Yes |
| 4.6 Were the complexities of the data (eg censoring, competing risks, sampling of controls) adequately accounted for? | Yes | Unclear | Yes | Yes | Yes |
| 4.7 Were relevant model performance measures adequately evaluated? | Yes | Yes | Yes | Yes | Yes |
| 4.8 Was model overfitting and optimism factored into model performance? | Yes | Yes | Yes | Yes | Yes |
| 4.9 Do the predictors and their assigned weights in the final model correspond to the results of the multivariate analysis? | Yes | Unclear | Yes | Yes | Yes |
| **Risk of bias introduced by the analysis (low/high/Unclear)** | Low | High | Low | Low | Low |
| **Step 4: General evaluation** | There is not enough information related to the chosen population, so there is a risk of introducing bias in the selection | No details of the population are found and there is an absence of the methodological data used | **-** | **-** | **-** |
| **Overall assessment of risk of bias** | High | High | Low | Low | Low |
| **General judgment of applicability** | Low | High | Low | Low | Low |
|  |  |  |  |  |  |
|  |  |  |  |  |  |
| **Domain 1** | **Swan et al. (2021) (1)** | **Swan et al. (2021) (2)** | **Tan et al. (2022)** | **Tang et al. (2021) (1)** | **Tang et al. (2021) (2)** |
| **A. Risk of bias** | **18-07-22** | **18-07-22** | **18-07-22** | **18-07-22** | **18-07-22** |
| 1.1 Were appropriate data sources used, e.g. data from cohort studies, RCTs or nested case-controls? | Unclear | Unclear | Yes | Yes | Yes |
| 1.2 Were all inclusions and exclusions of participants appropriate? | No | No | Unclear | Unclear | Unclear |
| Risk of bias introduced by participant selection (low/high/unclear) | High | High | Low | Low | Low |
| **B. Applicability** |  |  |  |  |  |
| Concern that included participants and setting do not match review question (low/high/unclear) | Low | Low | Low | Low | Low |
| **Domain 2** |  |  |  |  |  |
| **A. Risk of bias - predictors** |  |  |  |  |  |
| 2.1 Were predictors defined and evaluated in a similar way for all participants? | Yes | Yes | Yes | Yes | Yes |
| 2.2 Were predictor evaluations performed without knowing the outcome data? | Yes | Yes | Yes | Yes | Yes |
| 2.3 Are all the predictors available at the time the model is intended to be used? | Yes | Yes | Yes | Yes | Yes |
| Risk of bias introduced by the predictors or their assessment (low/high/unclear) | Low | Low | Low | Low | Low |
| **B. Applicability** |  |  |  |  |  |
| Concern that the definition, evaluation, or timing of predictors in the model do not match the review question (low/high/unclear) | Low | Low | Low | Low | Low |
| **Domain 3** |  |  |  |  |  |
| **A. Risk of bias - outcome** |  |  |  |  |  |
| Was the result properly determined? | Yes | Yes | Yes | Yes | Yes |
| Was a standard or prespecified outcome definition used? | Yes | Yes | Yes | Unclear | Yes |
| Were predictors excluded from the outcome definition? | Yes | Yes | Yes | Yes | Yes |
| Was the outcome similarly defined and determined for all participants? | Yes | Yes | Yes | Yes | Yes |
| Was the outcome determined without knowing the predictor information? | Yes | Yes | Yes | Yes | Yes |
| Was the time interval between the evaluation of the predictor and the determination of the result adequate? | Yes | Yes | Yes | Yes | Yes |
| Risk of bias introduced by the result or its determination (low/high/unclear) | Low | Low | Low | High | Low |
| **B. Applicability** |  |  |  |  |  |
| If a composite outcome was used, please describe the relative frequency/distribution of each contributing outcome: | Yes | Yes | Yes | Yes | Yes |
| Concern that the outcome, its definition, timing, or determination does not match the review question (low/high/unclear) | Low | Low | Low | Low | Low |
| **Domain 4** |  |  |  |  |  |
| **A. Risk of bias** |  |  |  |  |  |
| Describe the number of participants, number of candidate predictors, outcome events, and events per candidate predictor: | Yes | Yes | Yes | Unclear | Yes |
| Describe how the model was developed (for example, with respect to modeling technique (eg, survival or logistic modeling), selection of predictors, and definition of risk groups): | Yes | Yes | Yes | Yes | Yes |
| Describe if and how the model was validated, either internally (eg, bootstrapping, cross-validation, split random sample) or externally (eg, temporal validation, geographic validation, different setting, different type of participants): | Yes | Yes | Yes | Unclear | Yes |
| Describe the performance measures of the model, e.g. (re)calibration, discrimination, (re)classification, net profit and if adjusted for optimism: | Yes | Yes | Yes | Unclear | Yes |
| Describe the participants who were excluded from the analysis: | Yes | Yes | Yes | NA | Yes |
| Describe missing data on predictors and outcomes, as well as methods used for missing data: | Yes | Yes | Yes | Unclear | Yes |
| 4.1 Was there a reasonable number of participants with the result? | Yes | Yes | Yes | Unclear | Yes |
| 4.2 Were continuous and categorical predictors handled correctly? | Yes | Yes | Yes | Unclear | Yes |
| 4.3 Were all enrolled participants included in the analysis? | Yes | Yes | Yes | Unclear | Yes |
| 4.4 Were participants with missing data treated appropriately? | NA | NA | NA | Unclear | NA |
| 4.5 Was selection of predictors based on univariate analysis avoided? | Yes | Yes | Yes | Unclear | Yes |
| 4.6 Were the complexities of the data (eg censoring, competing risks, sampling of controls) adequately accounted for? | Yes | Yes | Yes | Unclear | Yes |
| 4.7 Were relevant model performance measures adequately evaluated? | Yes | Yes | Yes | Yes | Yes |
| 4.8 Was model overfitting and optimism factored into model performance? | Yes | Yes | Yes | Yes | Yes |
| 4.9 Do the predictors and their assigned weights in the final model correspond to the results of the multivariate analysis? | Yes | Yes | Yes | Unclear | Yes |
| **Risk of bias introduced by the analysis (low/high/Unclear)** | Low | Low | Low | High | Low |
| **Step 4: General evaluation** | There is not enough information related to the chosen population, so there is a risk of introducing bias in the selection | There is not enough information related to the population | - | There is no information regarding the population used, nor are the methodological details expanded | - |
| **Overall assessment of risk of bias** | High | High | Low | Low | Low |
| **General judgment of applicability** | Low | Low | Low | High | Low |
|  |  |  |  |  |  |
|  |  |  |  |  |  |
| **Domain 1** | **Tang et al. (2021) (3)** | **Tatapudi et al. (2021)** | **Teslya et al. (2021)** | **Tetteh et al. (2021)** |  |
| **A. Risk of bias** | **06-08-22** | **06-08-22** | **06-08-22** | **06-08-22** |  |
| 1.1 Were appropriate data sources used, e.g. data from cohort studies, RCTs or nested case-controls? | Unclear | Yes | Yes | Yes |  |
| 1.2 Were all inclusions and exclusions of participants appropriate? | No | Unclear | Unclear | Unclear |  |
| Risk of bias introduced by participant selection (low/high/unclear) | High | Low | Low | Low |  |
| **B. Applicability** |  |  |  |  |  |
| Concern that included participants and setting do not match review question (low/high/unclear) | Low | Low | Low | Low |  |
| **Domain 2** |  |  |  |  |  |
| **A. Risk of bias - predictors** |  |  |  |  |  |
| 2.1 Were predictors defined and evaluated in a similar way for all participants? | Yes | Yes | Yes | Yes |  |
| 2.2 Were predictor evaluations performed without knowing the outcome data? | Yes | Yes | Yes | Yes |  |
| 2.3 Are all the predictors available at the time the model is intended to be used? | Yes | Yes | Yes | Yes |  |
| Risk of bias introduced by the predictors or their assessment (low/high/unclear) | Low | Low | Low | Low |  |
| **B. Applicability** |  |  |  |  |  |
| Concern that the definition, evaluation, or timing of predictors in the model do not match the review question (low/high/unclear) | Low | Low | Low | Low |  |
| **Domain 3** |  |  |  |  |  |
| **A. Risk of bias - outcome** |  |  |  |  |  |
| Was the result properly determined? | Yes | Yes | Yes | Yes |  |
| Was a standard or prespecified outcome definition used? | Yes | Yes | Yes | Yes |  |
| Were predictors excluded from the outcome definition? | Yes | Yes | Yes | Yes |  |
| Was the outcome similarly defined and determined for all participants? | Yes | Yes | Yes | Yes |  |
| Was the outcome determined without knowing the predictor information? | Yes | Yes | Yes | Yes |  |
| Was the time interval between the evaluation of the predictor and the determination of the result adequate? | Yes | Yes | Yes | Yes |  |
| Risk of bias introduced by the result or its determination (low/high/unclear) | Low | Low | Low | Low |  |
| **B. Applicability** |  |  |  |  |  |
| If a composite outcome was used, please describe the relative frequency/distribution of each contributing outcome: | Yes | Yes | Yes | Yes |  |
| Concern that the outcome, its definition, timing, or determination does not match the review question (low/high/unclear) | Low | Low | Low | Low |  |
| **Domain 4** |  |  |  |  |  |
| **A. Risk of bias** |  |  |  |  |  |
| Describe the number of participants, number of candidate predictors, outcome events, and events per candidate predictor: | Yes | Yes | Yes | Yes |  |
| Describe how the model was developed (for example, with respect to modeling technique (eg, survival or logistic modeling), selection of predictors, and definition of risk groups): | Yes | Yes | Yes | Yes |  |
| Describe if and how the model was validated, either internally (eg, bootstrapping, cross-validation, split random sample) or externally (eg, temporal validation, geographic validation, different setting, different type of participants): | Yes | Yes | Yes | Yes |  |
| Describe the performance measures of the model, e.g. (re)calibration, discrimination, (re)classification, net profit and if adjusted for optimism: | Yes | Yes | Yes | Yes |  |
| Describe the participants who were excluded from the analysis: | Yes | Yes | Yes | Yes |  |
| Describe missing data on predictors and outcomes, as well as methods used for missing data: | Yes | Yes | Yes | Yes |  |
| 4.1 Was there a reasonable number of participants with the result? | Yes | Yes | Yes | Yes |  |
| 4.2 Were continuous and categorical predictors handled correctly? | Yes | Yes | Yes | Yes |  |
| 4.3 Were all enrolled participants included in the analysis? | Yes | Yes | Yes | Yes |  |
| 4.4 Were participants with missing data treated appropriately? | NA | NA | NA | NA |  |
| 4.5 Was selection of predictors based on univariate analysis avoided? | Yes | Yes | Yes | Yes |  |
| 4.6 Were the complexities of the data (eg censoring, competing risks, sampling of controls) adequately accounted for? | Yes | Yes | Yes | Yes |  |
| 4.7 Were relevant model performance measures adequately evaluated? | Yes | Yes | Yes | Yes |  |
| 4.8 Was model overfitting and optimism factored into model performance? | Yes | Yes | Yes | Yes |  |
| 4.9 Do the predictors and their assigned weights in the final model correspond to the results of the multivariate analysis? | Yes | Yes | Yes | Yes |  |
| **Risk of bias introduced by the analysis (low/high/Unclear)** | Low | Low | Low | Low |  |
| **Step 4: General evaluation** | There is not enough information related to the chosen population, so there is a risk of introducing bias in the selection | **-** | **-** | **-** |  |
| **Overall assessment of risk of bias** | High | Low | Low | Low |  |
| **General judgment of applicability** | Low | Low | Low | Low |  |
|  |  |  |  |  |  |
|  |  |  |  |  |  |
| **Domain 1** | **Thompson et al. (2021)** | **Tonkens et al. (2021)** | **Topîrceanu (2021)** | **Torku et al. (2021)** | **Tran et al. (2021)** |
| **A. Risk of bias** | **06-08-22** | **06-08-22** | **06-08-22** | **06-08-22** | **06-08-22** |
| 1.1 Were appropriate data sources used, e.g. data from cohort studies, RCTs or nested case-controls? | Yes | Unclear | Unclear | Yes | Yes |
| 1.2 Were all inclusions and exclusions of participants appropriate? | Yes | No | No | Yes | Yes |
| Risk of bias introduced by participant selection (low/high/unclear) | Low | High | High | Low | Low |
| **B. Applicability** |  |  |  |  |  |
| Concern that included participants and setting do not match review question (low/high/unclear) | Low | Low | Low | Low | Low |
| **Domain 2** |  |  |  |  |  |
| **A. Risk of bias - predictors** |  |  |  |  |  |
| 2.1 Were predictors defined and evaluated in a similar way for all participants? | Yes | Yes | Yes | Yes | Yes |
| 2.2 Were predictor evaluations performed without knowing the outcome data? | Yes | Yes | Yes | Yes | Yes |
| 2.3 Are all the predictors available at the time the model is intended to be used? | Yes | Yes | Yes | Yes | Yes |
| Risk of bias introduced by the predictors or their assessment (low/high/unclear) | Low | Low | Low | Low | Low |
| **B. Applicability** |  |  |  |  |  |
| Concern that the definition, evaluation, or timing of predictors in the model do not match the review question (low/high/unclear) | Low | Low | Low | Low | Low |
| **Domain 3** |  |  |  |  |  |
| **A. Risk of bias - outcome** |  |  |  |  |  |
| Was the result properly determined? | Yes | Yes | Yes | Yes | Yes |
| Was a standard or prespecified outcome definition used? | Yes | Yes | Yes | Yes | Yes |
| Were predictors excluded from the outcome definition? | Yes | Yes | Yes | Yes | Yes |
| Was the outcome similarly defined and determined for all participants? | Yes | Yes | Yes | Yes | Yes |
| Was the outcome determined without knowing the predictor information? | Yes | Yes | Yes | Yes | Yes |
| Was the time interval between the evaluation of the predictor and the determination of the result adequate? | Yes | Yes | Yes | Yes | Yes |
| Risk of bias introduced by the result or its determination (low/high/unclear) | Low | Low | Low | Low | Low |
| **B. Applicability** |  |  |  |  |  |
| If a composite outcome was used, please describe the relative frequency/distribution of each contributing outcome: | Yes | Yes | Yes | Yes | Yes |
| Concern that the outcome, its definition, timing, or determination does not match the review question (low/high/unclear) | Low | Low | Low | Low | Low |
| **Domain 4** |  |  |  |  |  |
| **A. Risk of bias** |  |  |  |  |  |
| Describe the number of participants, number of candidate predictors, outcome events, and events per candidate predictor: | Yes | Yes | Yes | Yes | Yes |
| Describe how the model was developed (for example, with respect to modeling technique (eg, survival or logistic modeling), selection of predictors, and definition of risk groups): | Yes | Yes | Yes | Yes | Yes |
| Describe if and how the model was validated, either internally (eg, bootstrapping, cross-validation, split random sample) or externally (eg, temporal validation, geographic validation, different setting, different type of participants): | Yes | Yes | Yes | Yes | Yes |
| Describe the performance measures of the model, e.g. (re)calibration, discrimination, (re)classification, net profit and if adjusted for optimism: | Yes | Yes | Yes | Yes | Yes |
| Describe the participants who were excluded from the analysis: | Yes | Yes | Yes | Yes | Yes |
| Describe missing data on predictors and outcomes, as well as methods used for missing data: | Yes | Yes | Yes | Yes | Yes |
| 4.1 Was there a reasonable number of participants with the result? | Yes | Yes | Yes | Yes | Yes |
| 4.2 Were continuous and categorical predictors handled correctly? | Yes | Yes | Yes | Yes | Yes |
| 4.3 Were all enrolled participants included in the analysis? | Yes | Yes | Yes | Yes | Yes |
| 4.4 Were participants with missing data treated appropriately? | NA | NA | NA | NA | NA |
| 4.5 Was selection of predictors based on univariate analysis avoided? | Yes | Yes | Yes | Yes | Yes |
| 4.6 Were the complexities of the data (eg censoring, competing risks, sampling of controls) adequately accounted for? | Yes | Yes | Yes | Yes | Yes |
| 4.7 Were relevant model performance measures adequately evaluated? | Yes | Yes | Yes | Yes | Yes |
| 4.8 Was model overfitting and optimism factored into model performance? | Yes | Yes | Yes | Yes | Yes |
| 4.9 Do the predictors and their assigned weights in the final model correspond to the results of the multivariate analysis? | Yes | Yes | Yes | Yes | Yes |
| **Risk of bias introduced by the analysis (low/high/Unclear)** | Low | Low | Low | Low | Low |
| **Step 4: General evaluation** | **-** | There is not enough information related to the population | There is not enough information related to the population | **-** | **-** |
| **Overall assessment of risk of bias** | Low | High | High | Low | Low |
| **General judgment of applicability** | Low | Low | Low | Low | Low |
|  |  |  |  |  |  |
|  |  |  |  |  |  |
| **Domain 1** | **Truszkowska et al. (2021)** | **Truszkowska et al. (2022)** | **Usherwood et al. (2021)** | **Utamura et al. (2021)** | **Van Egeren et al. (2021)** |
| **A. Risk of bias** | **06-08-22** | **08-08-22** | **08-08-22** | **08-08-22** | **08-08-22** |
| 1.1 Were appropriate data sources used, e.g. data from cohort studies, RCTs or nested case-controls? | Yes | Yes | Unclear | Yes | Unclear |
| 1.2 Were all inclusions and exclusions of participants appropriate? | Yes | Yes | No | Yes | No |
| Risk of bias introduced by participant selection (low/high/unclear) | Low | Low | High | Low | High |
| **B. Applicability** |  |  |  |  |  |
| Concern that included participants and setting do not match review question (low/high/unclear) | Low | Low | Low | Low | Low |
| **Domain 2** |  |  |  |  |  |
| **A. Risk of bias - predictors** |  |  |  |  |  |
| 2.1 Were predictors defined and evaluated in a similar way for all participants? | Yes | Yes | Yes | Yes | Yes |
| 2.2 Were predictor evaluations performed without knowing the outcome data? | Yes | Yes | Yes | Yes | Yes |
| 2.3 Are all the predictors available at the time the model is intended to be used? | Yes | Yes | Yes | Yes | Yes |
| Risk of bias introduced by the predictors or their assessment (low/high/unclear) | Low | Low | Low | Low | Low |
| **B. Applicability** |  |  |  |  |  |
| Concern that the definition, evaluation, or timing of predictors in the model do not match the review question (low/high/unclear) | Low | Low | Low | Low | Low |
| **Domain 3** |  |  |  |  |  |
| **A. Risk of bias - outcome** |  |  |  |  |  |
| Was the result properly determined? | Yes | Yes | Yes | Yes | Yes |
| Was a standard or prespecified outcome definition used? | Yes | Yes | Yes | Yes | Yes |
| Were predictors excluded from the outcome definition? | Yes | Yes | Yes | Yes | Yes |
| Was the outcome similarly defined and determined for all participants? | Yes | Yes | Yes | Yes | Yes |
| Was the outcome determined without knowing the predictor information? | Yes | Yes | Yes | Yes | Yes |
| Was the time interval between the evaluation of the predictor and the determination of the result adequate? | Yes | Yes | Yes | Yes | Yes |
| Risk of bias introduced by the result or its determination (low/high/unclear) | Low | Low | Low | Low | Low |
| **B. Applicability** |  |  |  |  |  |
| If a composite outcome was used, please describe the relative frequency/distribution of each contributing outcome: | Yes | Yes | Yes | Yes | Yes |
| Concern that the outcome, its definition, timing, or determination does not match the review question (low/high/unclear) | Low | Low | Low | Low | Low |
| **Domain 4** |  |  |  |  |  |
| **A. Risk of bias** |  |  |  |  |  |
| Describe the number of participants, number of candidate predictors, outcome events, and events per candidate predictor: | Yes | Yes | Yes | Yes | Yes |
| Describe how the model was developed (for example, with respect to modeling technique (eg, survival or logistic modeling), selection of predictors, and definition of risk groups): | Yes | Yes | Yes | Yes | Yes |
| Describe if and how the model was validated, either internally (eg, bootstrapping, cross-validation, split random sample) or externally (eg, temporal validation, geographic validation, different setting, different type of participants): | Yes | Yes | Yes | Yes | Yes |
| Describe the performance measures of the model, e.g. (re)calibration, discrimination, (re)classification, net profit and if adjusted for optimism: | Yes | Yes | Yes | Yes | Yes |
| Describe the participants who were excluded from the analysis: | Yes | Yes | Yes | Yes | Yes |
| Describe missing data on predictors and outcomes, as well as methods used for missing data: | Yes | Yes | Yes | Yes | Yes |
| 4.1 Was there a reasonable number of participants with the result? | Yes | Yes | Yes | Yes | Yes |
| 4.2 Were continuous and categorical predictors handled correctly? | Yes | Yes | Yes | Yes | Yes |
| 4.3 Were all enrolled participants included in the analysis? | Yes | Yes | Yes | Yes | Yes |
| 4.4 Were participants with missing data treated appropriately? | NA | NA | NA | NA | NA |
| 4.5 Was selection of predictors based on univariate analysis avoided? | Yes | Yes | Yes | Yes | Yes |
| 4.6 Were the complexities of the data (eg censoring, competing risks, sampling of controls) adequately accounted for? | Yes | Yes | Yes | Yes | Yes |
| 4.7 Were relevant model performance measures adequately evaluated? | Yes | Yes | Yes | Yes | Yes |
| 4.8 Was model overfitting and optimism factored into model performance? | Yes | Yes | Yes | Yes | Yes |
| 4.9 Do the predictors and their assigned weights in the final model correspond to the results of the multivariate analysis? | Yes | Yes | Yes | Yes | Yes |
| **Risk of bias introduced by the analysis (low/high/Unclear)** | Low | Low | Low | Low | Low |
| **Step 4: General evaluation** | **-** | **-** | There is not enough information related to the population | - | There is not enough information related to the population |
| **Overall assessment of risk of bias** | Low | Low | High | Low | High |
| **General judgment of applicability** | Low | Low | Low | Low | Low |
|  |  |  |  |  |  |
|  |  |  |  |  |  |
| **Domain 1** | **Van Gordon et al. (2021)** | **Van Heusden et al. (2021)** | **Gómezet al. (2022)** | **Viana et al. (2021)** | **Vignals et al. (2021)** |
| **A. Risk of bias** | **08-08-22** | **08-08-22** | **08-08-22** | **08-08-22** | **08-08-22** |
| 1.1 Were appropriate data sources used, e.g. data from cohort studies, RCTs or nested case-controls? | Yes | Yes | Yes | Yes | Yes |
| 1.2 Were all inclusions and exclusions of participants appropriate? | Yes | Yes | Yes | Yes | Yes |
| Risk of bias introduced by participant selection (low/high/unclear) | Low | Low | Low | Low | Low |
| **B. Applicability** |  |  |  |  |  |
| Concern that included participants and setting do not match review question (low/high/unclear) | Low | Low | Low | Low | Low |
| **Domain 2** |  |  |  |  |  |
| **A. Risk of bias - predictors** |  |  |  |  |  |
| 2.1 Were predictors defined and evaluated in a similar way for all participants? | Yes | Yes | Yes | Yes | Yes |
| 2.2 Were predictor evaluations performed without knowing the outcome data? | Yes | Yes | Yes | Yes | Yes |
| 2.3 Are all the predictors available at the time the model is intended to be used? | Yes | Yes | Yes | Yes | Yes |
| Risk of bias introduced by the predictors or their assessment (low/high/unclear) | Low | Low | Low | Low | Low |
| **B. Applicability** |  |  |  |  |  |
| Concern that the definition, evaluation, or timing of predictors in the model do not match the review question (low/high/unclear) | Low | Low | Low | Low | Low |
| **Domain 3** |  |  |  |  |  |
| **A. Risk of bias - outcome** |  |  |  |  |  |
| Was the result properly determined? | Yes | Yes | Yes | Yes | Yes |
| Was a standard or prespecified outcome definition used? | Yes | Yes | Yes | Yes | Yes |
| Were predictors excluded from the outcome definition? | Yes | Yes | Yes | Yes | Yes |
| Was the outcome similarly defined and determined for all participants? | Yes | Yes | Yes | Yes | Yes |
| Was the outcome determined without knowing the predictor information? | Yes | Yes | Yes | Yes | Yes |
| Was the time interval between the evaluation of the predictor and the determination of the result adequate? | Yes | Yes | Yes | Yes | Yes |
| Risk of bias introduced by the result or its determination (low/high/unclear) | Low | Low | Low | Low | Low |
| **B. Applicability** |  |  |  |  |  |
| If a composite outcome was used, please describe the relative frequency/distribution of each contributing outcome: | Yes | Yes | Yes | Yes | Yes |
| Concern that the outcome, its definition, timing, or determination does not match the review question (low/high/unclear) | Low | Low | Low | Low | Low |
| **Domain 4** |  |  |  |  |  |
| **A. Risk of bias** |  |  |  |  |  |
| Describe the number of participants, number of candidate predictors, outcome events, and events per candidate predictor: | Yes | Yes | Yes | Yes | Yes |
| Describe how the model was developed (for example, with respect to modeling technique (eg, survival or logistic modeling), selection of predictors, and definition of risk groups): | Yes | Yes | Yes | Yes | Yes |
| Describe if and how the model was validated, either internally (eg, bootstrapping, cross-validation, split random sample) or externally (eg, temporal validation, geographic validation, different setting, different type of participants): | Yes | Yes | Yes | Yes | Yes |
| Describe the performance measures of the model, e.g. (re)calibration, discrimination, (re)classification, net profit and if adjusted for optimism: | Yes | Yes | Yes | Yes | Yes |
| Describe the participants who were excluded from the analysis: | Yes | Yes | Yes | Yes | Yes |
| Describe missing data on predictors and outcomes, as well as methods used for missing data: | Yes | Yes | Yes | Yes | Yes |
| 4.1 Was there a reasonable number of participants with the result? | Yes | Yes | Yes | Yes | Yes |
| 4.2 Were continuous and categorical predictors handled correctly? | Yes | Yes | Yes | Yes | Yes |
| 4.3 Were all enrolled participants included in the analysis? | Yes | Yes | Yes | Yes | Yes |
| 4.4 Were participants with missing data treated appropriately? | NA | NA | NA | NA | NA |
| 4.5 Was selection of predictors based on univariate analysis avoided? | Yes | Yes | Yes | Yes | Yes |
| 4.6 Were the complexities of the data (eg censoring, competing risks, sampling of controls) adequately accounted for? | Yes | Yes | Yes | Yes | Yes |
| 4.7 Were relevant model performance measures adequately evaluated? | Yes | Yes | Yes | Yes | Yes |
| 4.8 Was model overfitting and optimism factored into model performance? | Yes | Yes | Yes | Yes | Yes |
| 4.9 Do the predictors and their assigned weights in the final model correspond to the results of the multivariate analysis? | Yes | Yes | Yes | Yes | Yes |
| **Risk of bias introduced by the analysis (low/high/Unclear)** | Low | Low | Low | Low | Low |
| **Step 4: General evaluation** | **-** | **-** | **-** | **-** | **-** |
| **Overall assessment of risk of bias** | Low | Low | Low | Low | Low |
| **General judgment of applicability** | Low | Low | Low | Low | Low |
|  |  |  |  |  |  |
|  |  |  |  |  |  |
| **Domain 1** | **Vilches et al. (2021) (1)** | **Vilches et al. (2021) (2)** | **Vilches et al. (2021) (3)** | **Vilches et al. (2022) (4)** | **De Visscher et al. (2021)** |
| **A. Risk of bias** | **08-08-22** | **08-08-22** | **08-08-22** | **08-08-22** | **08-08-22** |
| 1.1 Were appropriate data sources used, e.g. data from cohort studies, RCTs or nested case-controls? | Yes | Unclear | Unclear | Yes | Yes |
| 1.2 Were all inclusions and exclusions of participants appropriate? | Yes | No | No | Yes | Yes |
| Risk of bias introduced by participant selection (low/high/unclear) | Low | High | High | Low | Low |
| **B. Applicability** |  |  |  |  |  |
| Concern that included participants and setting do not match review question (low/high/unclear) | Low | Low | Low | Low | Low |
| **Domain 2** |  |  |  |  |  |
| **A. Risk of bias - predictors** |  |  |  |  |  |
| 2.1 Were predictors defined and evaluated in a similar way for all participants? | Yes | Yes | Yes | Yes | Yes |
| 2.2 Were predictor evaluations performed without knowing the outcome data? | Yes | Yes | Yes | Yes | Yes |
| 2.3 Are all the predictors available at the time the model is intended to be used? | Yes | Yes | Yes | Yes | Yes |
| Risk of bias introduced by the predictors or their assessment (low/high/unclear) | Low | Low | Low | Low | Low |
| **B. Applicability** |  |  |  |  |  |
| Concern that the definition, evaluation, or timing of predictors in the model do not match the review question (low/high/unclear) | Low | Low | Low | Low | Low |
| **Domain 3** |  |  |  |  |  |
| **A. Risk of bias - outcome** |  |  |  |  |  |
| Was the result properly determined? | Yes | Yes | Yes | Yes | Yes |
| Was a standard or prespecified outcome definition used? | Unclear | Yes | Yes | Unclear | Yes |
| Were predictors excluded from the outcome definition? | Yes | Yes | Yes | Yes | Yes |
| Was the outcome similarly defined and determined for all participants? | Yes | Yes | Yes | Yes | Yes |
| Was the outcome determined without knowing the predictor information? | Yes | Yes | Yes | Yes | Yes |
| Was the time interval between the evaluation of the predictor and the determination of the result adequate? | Yes | Yes | Yes | Yes | Yes |
| Risk of bias introduced by the result or its determination (low/high/unclear) | High | Low | Low | High | Low |
| **B. Applicability** |  |  |  |  |  |
| If a composite outcome was used, please describe the relative frequency/distribution of each contributing outcome: | Yes | Yes | Yes | Yes | Yes |
| Concern that the outcome, its definition, timing, or determination does not match the review question (low/high/unclear) | Low | Low | Low | Low | Low |
| **Domain 4** |  |  |  |  |  |
| **A. Risk of bias** |  |  |  |  |  |
| Describe the number of participants, number of candidate predictors, outcome events, and events per candidate predictor: | Unclear | Yes | Yes | Unclear | Yes |
| Describe how the model was developed (for example, with respect to modeling technique (eg, survival or logistic modeling), selection of predictors, and definition of risk groups): | Yes | Yes | Yes | Yes | Yes |
| Describe if and how the model was validated, either internally (eg, bootstrapping, cross-validation, split random sample) or externally (eg, temporal validation, geographic validation, different setting, different type of participants): | Unclear | Yes | Yes | Unclear | Yes |
| Describe the performance measures of the model, e.g. (re)calibration, discrimination, (re)classification, net profit and if adjusted for optimism: | Unclear | Yes | Yes | Unclear | Yes |
| Describe the participants who were excluded from the analysis: | NA | Yes | Yes | NA | Yes |
| Describe missing data on predictors and outcomes, as well as methods used for missing data: | Unclear | Yes | Yes | Unclear | Yes |
| 4.1 Was there a reasonable number of participants with the result? | Unclear | Yes | Yes | Unclear | Yes |
| 4.2 Were continuous and categorical predictors handled correctly? | Unclear | Yes | Yes | Unclear | Yes |
| 4.3 Were all enrolled participants included in the analysis? | Unclear | Yes | Yes | Unclear | Yes |
| 4.4 Were participants with missing data treated appropriately? | Unclear | NA | NA | Unclear | NA |
| 4.5 Was selection of predictors based on univariate analysis avoided? | Unclear | Yes | Yes | Unclear | Yes |
| 4.6 Were the complexities of the data (eg censoring, competing risks, sampling of controls) adequately accounted for? | Unclear | Yes | Yes | Unclear | Yes |
| 4.7 Were relevant model performance measures adequately evaluated? | Yes | Yes | Yes | Yes | Yes |
| 4.8 Was model overfitting and optimism factored into model performance? | Yes | Yes | Yes | Yes | Yes |
| 4.9 Do the predictors and their assigned weights in the final model correspond to the results of the multivariate analysis? | Unclear | Yes | Yes | Unclear | Yes |
| **Risk of bias introduced by the analysis (low/high/Unclear)** | High | Low | Low | High | Low |
| **Step 4: General evaluation** | There is not enough information related to the chosen population, so there is a risk of introducing bias in the selection | - | - | There is no information regarding the population used, nor are the methodological details expanded | **-** |
| **Overall assessment of risk of bias** | Low | High | High | Low | Low |
| **General judgment of applicability** | High | Low | Low | High | Low |
|  |  |  |  |  |  |
|  |  |  |  |  |  |
| **Domain 1** | **Volodymyrovych et al. (2021)** | **Walker et al. (2022)** | **Wang et al. (2021) (1)** | **Wang et al. (2021) (2)** | **Wang et al. (2022)** |
| **A. Risk of bias** | **08-08-22** | **08-08-22** | **08-08-22** | **09-08-22** | **09-08-22** |
| 1.1 Were appropriate data sources used, e.g. data from cohort studies, RCTs or nested case-controls? | Yes | Yes | Yes | Yes | Yes |
| 1.2 Were all inclusions and exclusions of participants appropriate? | Yes | Yes | Yes | Yes | Yes |
| Risk of bias introduced by participant selection (low/high/unclear) | Low | Low | Low | Low | Low |
| **B. Applicability** |  |  |  |  |  |
| Concern that included participants and setting do not match review question (low/high/unclear) | Low | Low | Low | Low | Low |
| **Domain 2** |  |  |  |  |  |
| **A. Risk of bias - predictors** |  |  |  |  |  |
| 2.1 Were predictors defined and evaluated in a similar way for all participants? | Yes | Yes | Yes | Yes | Yes |
| 2.2 Were predictor evaluations performed without knowing the outcome data? | Yes | Yes | Yes | Yes | Yes |
| 2.3 Are all the predictors available at the time the model is intended to be used? | Yes | Yes | Yes | Yes | Yes |
| Risk of bias introduced by the predictors or their assessment (low/high/unclear) | Low | Low | Low | Low | Low |
| **B. Applicability** |  |  |  |  |  |
| Concern that the definition, evaluation, or timing of predictors in the model do not match the review question (low/high/unclear) | Low | Low | Low | Low | Low |
| **Domain 3** |  |  |  |  |  |
| **A. Risk of bias - outcome** |  |  |  |  |  |
| Was the result properly determined? | Yes | Yes | Yes | Yes | Yes |
| Was a standard or prespecified outcome definition used? | Yes | Yes | Unclear | Unclear | Yes |
| Were predictors excluded from the outcome definition? | Yes | Yes | Yes | Yes | Yes |
| Was the outcome similarly defined and determined for all participants? | Yes | Yes | Yes | Yes | Yes |
| Was the outcome determined without knowing the predictor information? | Yes | Yes | Yes | Yes | Yes |
| Was the time interval between the evaluation of the predictor and the determination of the result adequate? | Yes | Yes | Yes | Yes | Yes |
| Risk of bias introduced by the result or its determination (low/high/unclear) | Low | Low | High | High | Low |
| **B. Applicability** |  |  |  |  |  |
| If a composite outcome was used, please describe the relative frequency/distribution of each contributing outcome: | Yes | Yes | Yes | Yes | Yes |
| Concern that the outcome, its definition, timing, or determination does not match the review question (low/high/unclear) | Low | Low | Low | Low | Low |
| **Domain 4** |  |  |  |  |  |
| **A. Risk of bias** |  |  |  |  |  |
| Describe the number of participants, number of candidate predictors, outcome events, and events per candidate predictor: | Yes | Yes | Unclear | Unclear | Yes |
| Describe how the model was developed (for example, with respect to modeling technique (eg, survival or logistic modeling), selection of predictors, and definition of risk groups): | Yes | Yes | Yes | Yes | Yes |
| Describe if and how the model was validated, either internally (eg, bootstrapping, cross-validation, split random sample) or externally (eg, temporal validation, geographic validation, different setting, different type of participants): | Yes | Yes | Unclear | Unclear | Yes |
| Describe the performance measures of the model, e.g. (re)calibration, discrimination, (re)classification, net profit and if adjusted for optimism: | Yes | Yes | Unclear | Unclear | Yes |
| Describe the participants who were excluded from the analysis: | Yes | Yes | NA | NA | Yes |
| Describe missing data on predictors and outcomes, as well as methods used for missing data: | Yes | Yes | Unclear | Unclear | Yes |
| 4.1 Was there a reasonable number of participants with the result? | Yes | Yes | Unclear | Unclear | Yes |
| 4.2 Were continuous and categorical predictors handled correctly? | Yes | Yes | Unclear | Unclear | Yes |
| 4.3 Were all enrolled participants included in the analysis? | Yes | Yes | Unclear | Unclear | Yes |
| 4.4 Were participants with missing data treated appropriately? | NA | NA | Unclear | Unclear | NA |
| 4.5 Was selection of predictors based on univariate analysis avoided? | Yes | Yes | Unclear | Unclear | Yes |
| 4.6 Were the complexities of the data (eg censoring, competing risks, sampling of controls) adequately accounted for? | Yes | Yes | Unclear | Unclear | Yes |
| 4.7 Were relevant model performance measures adequately evaluated? | Yes | Yes | Yes | Yes | Yes |
| 4.8 Was model overfitting and optimism factored into model performance? | Yes | Yes | Yes | Yes | Yes |
| 4.9 Do the predictors and their assigned weights in the final model correspond to the results of the multivariate analysis? | Yes | Yes | Unclear | Unclear | Yes |
| **Risk of bias introduced by the analysis (low/high/Unclear)** | Low | Low | High | High | Low |
| **Step 4: General evaluation** | **-** | **-** | There is no information regarding the population used, the information related to modeling is not clear | There is no information regarding the population used, the information related to modeling is not clear | **-** |
| **Overall assessment of risk of bias** | Low | Low | Low | Low | Low |
| **General judgment of applicability** | Low | Low | High | High | Low |
|  |  |  |  |  |  |
|  |  |  |  |  |  |
| **Domain 1** | **Webb (2021)** | **Więcek et al. (2021)** | **Więcek et al. (2022)** | **Wieland et al. (2021)** | **Conn et al. (2021)** |
| **A. Risk of bias** | **09-08-22** | **09-08-22** | **09-08-22** | **09-08-22** | **09-08-22** |
| 1.1 Were appropriate data sources used, e.g. data from cohort studies, RCTs or nested case-controls? | Yes | Yes | Yes | Yes | Yes |
| 1.2 Were all inclusions and exclusions of participants appropriate? | Yes | Yes | Yes | Yes | Yes |
| Risk of bias introduced by participant selection (low/high/unclear) | Low | Low | Low | Low | Low |
| **B. Applicability** |  |  |  |  |  |
| Concern that included participants and setting do not match review question (low/high/unclear) | Low | Low | Low | Low | Low |
| **Domain 2** |  |  |  |  |  |
| **A. Risk of bias - predictors** |  |  |  |  |  |
| 2.1 Were predictors defined and evaluated in a similar way for all participants? | Yes | Yes | Yes | Yes | Yes |
| 2.2 Were predictor evaluations performed without knowing the outcome data? | Yes | Yes | Yes | Yes | Yes |
| 2.3 Are all the predictors available at the time the model is intended to be used? | Yes | Yes | Yes | Yes | Yes |
| Risk of bias introduced by the predictors or their assessment (low/high/unclear) | Low | Low | Low | Low | Low |
| **B. Applicability** |  |  |  |  |  |
| Concern that the definition, evaluation, or timing of predictors in the model do not match the review question (low/high/unclear) | Low | Low | Low | Low | Low |
| **Domain 3** |  |  |  |  |  |
| **A. Risk of bias - outcome** |  |  |  |  |  |
| Was the result properly determined? | Yes | Yes | Yes | Yes | Yes |
| Was a standard or prespecified outcome definition used? | Yes | Yes | Yes | Unclear | Yes |
| Were predictors excluded from the outcome definition? | Yes | Yes | Yes | Yes | Yes |
| Was the outcome similarly defined and determined for all participants? | Yes | Yes | Yes | Yes | Yes |
| Was the outcome determined without knowing the predictor information? | Yes | Yes | Yes | Yes | Yes |
| Was the time interval between the evaluation of the predictor and the determination of the result adequate? | Yes | Yes | Yes | Yes | Yes |
| Risk of bias introduced by the result or its determination (low/high/unclear) | Low | Low | Low | High | Low |
| **B. Applicability** |  |  |  |  |  |
| If a composite outcome was used, please describe the relative frequency/distribution of each contributing outcome: | Yes | Yes | Yes | Yes | Yes |
| Concern that the outcome, its definition, timing, or determination does not match the review question (low/high/unclear) | Low | Low | Low | Low | Low |
| **Domain 4** |  |  |  |  |  |
| **A. Risk of bias** |  |  |  |  |  |
| Describe the number of participants, number of candidate predictors, outcome events, and events per candidate predictor: | Yes | Yes | Yes | Unclear | Yes |
| Describe how the model was developed (for example, with respect to modeling technique (eg, survival or logistic modeling), selection of predictors, and definition of risk groups): | Yes | Yes | Yes | Yes | Yes |
| Describe if and how the model was validated, either internally (eg, bootstrapping, cross-validation, split random sample) or externally (eg, temporal validation, geographic validation, different setting, different type of participants): | Yes | Yes | Yes | Unclear | Yes |
| Describe the performance measures of the model, e.g. (re)calibration, discrimination, (re)classification, net profit and if adjusted for optimism: | Yes | Yes | Yes | Unclear | Yes |
| Describe the participants who were excluded from the analysis: | Yes | Yes | Yes | NA | Yes |
| Describe missing data on predictors and outcomes, as well as methods used for missing data: | Yes | Yes | Yes | Unclear | Yes |
| 4.1 Was there a reasonable number of participants with the result? | Yes | Yes | Yes | Unclear | Yes |
| 4.2 Were continuous and categorical predictors handled correctly? | Yes | Yes | Yes | Unclear | Yes |
| 4.3 Were all enrolled participants included in the analysis? | Yes | Yes | Yes | Unclear | Yes |
| 4.4 Were participants with missing data treated appropriately? | NA | NA | NA | Unclear | NA |
| 4.5 Was selection of predictors based on univariate analysis avoided? | Yes | Yes | Yes | Unclear | Yes |
| 4.6 Were the complexities of the data (eg censoring, competing risks, sampling of controls) adequately accounted for? | Yes | Yes | Yes | Unclear | Yes |
| 4.7 Were relevant model performance measures adequately evaluated? | Yes | Yes | Yes | Yes | Yes |
| 4.8 Was model overfitting and optimism factored into model performance? | Yes | Yes | Yes | Yes | Yes |
| 4.9 Do the predictors and their assigned weights in the final model correspond to the results of the multivariate analysis? | Yes | Yes | Yes | Unclear | Yes |
| **Risk of bias introduced by the analysis (low/high/Unclear)** | Low | Low | Low | High | Low |
| **Step 4: General evaluation** | **-** | **-** | - | There is no information regarding the population used, the information related to modeling is not clear | - |
| **Overall assessment of risk of bias** | Low | Low | Low | Low | Low |
| **General judgment of applicability** | Low | Low | Low | High | Low |
|  |  |  |  |  |  |
|  |  |  |  |  |  |
| **Domain 1** | **Wirtz (2021)** | **Wong et al. (2021)** | **Xavier et al. (2022)** | **Xiong et al. (2022)** |  |
| **A. Risk of bias** | **09-08-22** | **09-08-22** | **09-08-22** | **09-08-22** |  |
| 1.1 Were appropriate data sources used, e.g. data from cohort studies, RCTs or nested case-controls? | Yes | Unclear | Yes | Yes |  |
| 1.2 Were all inclusions and exclusions of participants appropriate? | Yes | Unclear | Yes | Yes |  |
| Risk of bias introduced by participant selection (low/high/unclear) | Low | Unclear | Low | Low |  |
| **B. Applicability** |  |  |  |  |  |
| Concern that included participants and setting do not match review question (low/high/unclear) | Low | Unclear | Low | Low |  |
| **Domain 2** |  |  |  |  |  |
| **A. Risk of bias - predictors** |  |  |  |  |  |
| 2.1 Were predictors defined and evaluated in a similar way for all participants? | Yes | Yes | Yes | Yes |  |
| 2.2 Were predictor evaluations performed without knowing the outcome data? | Yes | Yes | Yes | Yes |  |
| 2.3 Are all the predictors available at the time the model is intended to be used? | Yes | Yes | Yes | Yes |  |
| Risk of bias introduced by the predictors or their assessment (low/high/unclear) | Low | Low | Low | Low |  |
| **B. Applicability** |  |  |  |  |  |
| Concern that the definition, evaluation, or timing of predictors in the model do not match the review question (low/high/unclear) | Low | Low | Low | Low |  |
| **Domain 3** |  |  |  |  |  |
| **A. Risk of bias - outcome** |  |  |  |  |  |
| Was the result properly determined? | Yes | Yes | Yes | Yes |  |
| Was a standard or prespecified outcome definition used? | Yes | Unclear | Yes | Yes |  |
| Were predictors excluded from the outcome definition? | Yes | Yes | Yes | Yes |  |
| Was the outcome similarly defined and determined for all participants? | Yes | Yes | Yes | Yes |  |
| Was the outcome determined without knowing the predictor information? | Yes | Yes | Yes | Yes |  |
| Was the time interval between the evaluation of the predictor and the determination of the result adequate? | Yes | Yes | Yes | Yes |  |
| Risk of bias introduced by the result or its determination (low/high/unclear) | Low | High | Low | Low |  |
| **B. Applicability** |  |  |  |  |  |
| If a composite outcome was used, please describe the relative frequency/distribution of each contributing outcome: | Yes | Yes | Yes | Yes |  |
| Concern that the outcome, its definition, timing, or determination does not match the review question (low/high/unclear) | Low | Low | Low | Low |  |
| **Domain 4** |  |  |  |  |  |
| **A. Risk of bias** |  |  |  |  |  |
| Describe the number of participants, number of candidate predictors, outcome events, and events per candidate predictor: | Yes | Yes | Yes | Yes |  |
| Describe how the model was developed (for example, with respect to modeling technique (eg, survival or logistic modeling), selection of predictors, and definition of risk groups): | Yes | Yes | Yes | Yes |  |
| Describe if and how the model was validated, either internally (eg, bootstrapping, cross-validation, split random sample) or externally (eg, temporal validation, geographic validation, different setting, different type of participants): | Yes | Yes | Yes | Yes |  |
| Describe the performance measures of the model, e.g. (re)calibration, discrimination, (re)classification, net profit and if adjusted for optimism: | Yes | Yes | Yes | Yes |  |
| Describe the participants who were excluded from the analysis: | Yes | Yes | Yes | Yes |  |
| Describe missing data on predictors and outcomes, as well as methods used for missing data: | Yes | Yes | Yes | Yes |  |
| 4.1 Was there a reasonable number of participants with the result? | Yes | Yes | Yes | Yes |  |
| 4.2 Were continuous and categorical predictors handled correctly? | Yes | Yes | Yes | Yes |  |
| 4.3 Were all enrolled participants included in the analysis? | Yes | Yes | Yes | Yes |  |
| 4.4 Were participants with missing data treated appropriately? | NA | NA | NA | NA |  |
| 4.5 Was selection of predictors based on univariate analysis avoided? | Yes | Yes | Yes | Yes |  |
| 4.6 Were the complexities of the data (eg censoring, competing risks, sampling of controls) adequately accounted for? | Yes | Yes | Yes | Yes |  |
| 4.7 Were relevant model performance measures adequately evaluated? | Yes | Yes | Yes | Yes |  |
| 4.8 Was model overfitting and optimism factored into model performance? | Yes | Yes | Yes | Yes |  |
| 4.9 Do the predictors and their assigned weights in the final model correspond to the results of the multivariate analysis? | Yes | Yes | Yes | Yes |  |
| **Risk of bias introduced by the analysis (low/high/Unclear)** | Low | Low | Low | Low |  |
| **Step 4: General evaluation** | - | There is no explicit information on the origin of the data. There are also methodological gaps | - | **-** |  |
| **Overall assessment of risk of bias** | Low | Unclear | Low | Low |  |
| **General judgment of applicability** | Low | High | Low | Low |  |
|  |  |  |  |  |  |
|  |  |  |  |  |  |
| **Domain 1** | **Yang et al. (2021) (1)** | **Yang et al. (2021) (2)** | **Yang et al. (2021) (3)** | **Yang et al. (2021) (4)** | **Yang et al. (2022) (1)** |
| **A. Risk of bias** | **09-08-22** | **09-08-22** | **09-08-22** | **09-08-22** | **09-08-22** |
| 1.1 Were appropriate data sources used, e.g. data from cohort studies, RCTs or nested case-controls? | Yes | Yes | Yes | Unclear | Yes |
| 1.2 Were all inclusions and exclusions of participants appropriate? | Yes | Yes | Yes | Unclear | Yes |
| Risk of bias introduced by participant selection (low/high/unclear) | Low | Low | Low | Unclear | Low |
| **B. Applicability** |  |  |  |  |  |
| Concern that included participants and setting do not match review question (low/high/unclear) | Low | Low | Low | Unclear | Low |
| **Domain 2** |  |  |  |  |  |
| **A. Risk of bias - predictors** |  |  |  |  |  |
| 2.1 Were predictors defined and evaluated in a similar way for all participants? | Yes | Yes | Yes | Yes | Yes |
| 2.2 Were predictor evaluations performed without knowing the outcome data? | Yes | Yes | Yes | Yes | Yes |
| 2.3 Are all the predictors available at the time the model is intended to be used? | Yes | Yes | Yes | Yes | Yes |
| Risk of bias introduced by the predictors or their assessment (low/high/unclear) | Low | Low | Low | Low | Low |
| **B. Applicability** |  |  |  |  |  |
| Concern that the definition, evaluation, or timing of predictors in the model do not match the review question (low/high/unclear) | Low | Low | Low | Low | Low |
| **Domain 3** |  |  |  |  |  |
| **A. Risk of bias - outcome** |  |  |  |  |  |
| Was the result properly determined? | Yes | Yes | Yes | Yes | Yes |
| Was a standard or prespecified outcome definition used? | Yes | Yes | Yes | Unclear | Yes |
| Were predictors excluded from the outcome definition? | Yes | Yes | Yes | Yes | Yes |
| Was the outcome similarly defined and determined for all participants? | Yes | Yes | Yes | Yes | Yes |
| Was the outcome determined without knowing the predictor information? | Yes | Yes | Yes | Yes | Yes |
| Was the time interval between the evaluation of the predictor and the determination of the result adequate? | Yes | Yes | Yes | Yes | Yes |
| Risk of bias introduced by the result or its determination (low/high/unclear) | Low | Low | Low | High | Low |
| **B. Applicability** |  |  |  |  |  |
| If a composite outcome was used, please describe the relative frequency/distribution of each contributing outcome: | Yes | Yes | Yes | Yes | Yes |
| Concern that the outcome, its definition, timing, or determination does not match the review question (low/high/unclear) | Low | Low | Low | Low | Low |
| **Domain 4** |  |  |  |  |  |
| **A. Risk of bias** |  |  |  |  |  |
| Describe the number of participants, number of candidate predictors, outcome events, and events per candidate predictor: | Yes | Yes | Yes | Yes | Yes |
| Describe how the model was developed (for example, with respect to modeling technique (eg, survival or logistic modeling), selection of predictors, and definition of risk groups): | Yes | Yes | Yes | Yes | Yes |
| Describe if and how the model was validated, either internally (eg, bootstrapping, cross-validation, split random sample) or externally (eg, temporal validation, geographic validation, different setting, different type of participants): | Yes | Yes | Yes | Yes | Yes |
| Describe the performance measures of the model, e.g. (re)calibration, discrimination, (re)classification, net profit and if adjusted for optimism: | Yes | Yes | Yes | Yes | Yes |
| Describe the participants who were excluded from the analysis: | Yes | Yes | Yes | Yes | Yes |
| Describe missing data on predictors and outcomes, as well as methods used for missing data: | Yes | Yes | Yes | Yes | Yes |
| 4.1 Was there a reasonable number of participants with the result? | Yes | Yes | Yes | Yes | Yes |
| 4.2 Were continuous and categorical predictors handled correctly? | Yes | Yes | Yes | Yes | Yes |
| 4.3 Were all enrolled participants included in the analysis? | Yes | Yes | Yes | Yes | Yes |
| 4.4 Were participants with missing data treated appropriately? | NA | NA | NA | NA | NA |
| 4.5 Was selection of predictors based on univariate analysis avoided? | Yes | Yes | Yes | Yes | Yes |
| 4.6 Were the complexities of the data (eg censoring, competing risks, sampling of controls) adequately accounted for? | Yes | Yes | Yes | Yes | Yes |
| 4.7 Were relevant model performance measures adequately evaluated? | Yes | Yes | Yes | Yes | Yes |
| 4.8 Was model overfitting and optimism factored into model performance? | Yes | Yes | Yes | Yes | Yes |
| 4.9 Do the predictors and their assigned weights in the final model correspond to the results of the multivariate analysis? | Yes | Yes | Yes | Yes | Yes |
| **Risk of bias introduced by the analysis (low/high/Unclear)** | Low | Low | Low | Low | Low |
| **Step 4: General evaluation** | **-** | **-** | - | There is no explicit information on the origin of the data. There are also methodological gaps | - |
| **Overall assessment of risk of bias** | Low | Low | Low | Unclear | Low |
| **General judgment of applicability** | Low | Low | Low | High | Low |
|  |  |  |  |  |  |
|  |  |  |  |  |  |
| **Domain 1** | **Yang et al. (2022) (2)** | **Yang et al. (2021) (5)** | **Young et al. (2021)** | **Yu et al. (2021)** | **Yuan et al. (2022)** |
| **A. Risk of bias** | **09-08-22** | **09-08-22** | **09-08-22** | **10-08-22** | **10-08-22** |
| 1.1 Were appropriate data sources used, e.g. data from cohort studies, RCTs or nested case-controls? | Yes | Yes | Unclear | Yes | Yes |
| 1.2 Were all inclusions and exclusions of participants appropriate? | Yes | Yes | No | Yes | Yes |
| Risk of bias introduced by participant selection (low/high/unclear) | Low | Low | High | Low | Low |
| **B. Applicability** |  |  |  |  |  |
| Concern that included participants and setting do not match review question (low/high/unclear) | Low | Low | Low | Low | Low |
| **Domain 2** |  |  |  |  |  |
| **A. Risk of bias - predictors** |  |  |  |  |  |
| 2.1 Were predictors defined and evaluated in a similar way for all participants? | Yes | Yes | Yes | Yes | Yes |
| 2.2 Were predictor evaluations performed without knowing the outcome data? | Yes | Yes | Yes | Yes | Yes |
| 2.3 Are all the predictors available at the time the model is intended to be used? | Yes | Yes | Yes | Yes | Yes |
| Risk of bias introduced by the predictors or their assessment (low/high/unclear) | Low | Low | Low | Low | Low |
| **B. Applicability** |  |  |  |  |  |
| Concern that the definition, evaluation, or timing of predictors in the model do not match the review question (low/high/unclear) | Low | Low | Low | Low | Low |
| **Domain 3** |  |  |  |  |  |
| **A. Risk of bias - outcome** |  |  |  |  |  |
| Was the result properly determined? | Yes | Yes | Yes | Yes | Yes |
| Was a standard or prespecified outcome definition used? | Yes | Yes | Yes | Yes | Yes |
| Were predictors excluded from the outcome definition? | Yes | Yes | Yes | Yes | Yes |
| Was the outcome similarly defined and determined for all participants? | Yes | Yes | Yes | Yes | Yes |
| Was the outcome determined without knowing the predictor information? | Yes | Yes | Yes | Yes | Yes |
| Was the time interval between the evaluation of the predictor and the determination of the result adequate? | Yes | Yes | Yes | Yes | Yes |
| Risk of bias introduced by the result or its determination (low/high/unclear) | Low | Low | Low | Low | Low |
| **B. Applicability** |  |  |  |  |  |
| If a composite outcome was used, please describe the relative frequency/distribution of each contributing outcome: | Yes | Yes | Yes | Yes | Yes |
| Concern that the outcome, its definition, timing, or determination does not match the review question (low/high/unclear) | Low | Low | Low | Low | Low |
| **Domain 4** |  |  |  |  |  |
| **A. Risk of bias** |  |  |  |  |  |
| Describe the number of participants, number of candidate predictors, outcome events, and events per candidate predictor: | Yes | Yes | Yes | Yes | Yes |
| Describe how the model was developed (for example, with respect to modeling technique (eg, survival or logistic modeling), selection of predictors, and definition of risk groups): | Yes | Yes | Yes | Yes | Yes |
| Describe if and how the model was validated, either internally (eg, bootstrapping, cross-validation, split random sample) or externally (eg, temporal validation, geographic validation, different setting, different type of participants): | Yes | Yes | Yes | Yes | Yes |
| Describe the performance measures of the model, e.g. (re)calibration, discrimination, (re)classification, net profit and if adjusted for optimism: | Yes | Yes | Yes | Yes | Yes |
| Describe the participants who were excluded from the analysis: | Yes | Yes | Yes | Yes | Yes |
| Describe missing data on predictors and outcomes, as well as methods used for missing data: | Yes | Yes | Yes | Yes | Yes |
| 4.1 Was there a reasonable number of participants with the result? | Yes | Yes | Yes | Yes | Yes |
| 4.2 Were continuous and categorical predictors handled correctly? | Yes | Yes | Yes | Yes | Yes |
| 4.3 Were all enrolled participants included in the analysis? | Yes | Yes | Yes | Yes | Yes |
| 4.4 Were participants with missing data treated appropriately? | NA | NA | NA | NA | NA |
| 4.5 Was selection of predictors based on univariate analysis avoided? | Yes | Yes | Yes | Yes | Yes |
| 4.6 Were the complexities of the data (eg censoring, competing risks, sampling of controls) adequately accounted for? | Yes | Yes | Yes | Yes | Yes |
| 4.7 Were relevant model performance measures adequately evaluated? | Yes | Yes | Yes | Yes | Yes |
| 4.8 Was model overfitting and optimism factored into model performance? | Yes | Yes | Yes | Yes | Yes |
| 4.9 Do the predictors and their assigned weights in the final model correspond to the results of the multivariate analysis? | Yes | Yes | Yes | Yes | Yes |
| **Risk of bias introduced by the analysis (low/high/Unclear)** | Low | Low | Low | Low | Low |
| **Step 4: General evaluation** | **-** | - | There is no information related to the origin of the data | - | **-** |
| **Overall assessment of risk of bias** | Low | Low | High | Low | Low |
| **General judgment of applicability** | Low | Low | Low | Low | Low |
|  |  |  |  |  |  |
|  |  |  |  |  |  |
| **Domain 1** | **Zachreson et al. (2022)** | **Zhang et al. (2021) (1)** | **Zhang et al. (2021) (2)** | **Zhang et al. (2022)** | **Zhao et al. (2021) (1)** |
| **A. Risk of bias** | **10-08-22** | **10-08-22** | **10-08-22** | **10-08-22** | **10-08-22** |
| 1.1 Were appropriate data sources used, e.g. data from cohort studies, RCTs or nested case-controls? | Unclear | Yes | Unclear | Yes | Unclear |
| 1.2 Were all inclusions and exclusions of participants appropriate? | Unclear | Yes | Unclear | Yes | Unclear |
| Risk of bias introduced by participant selection (low/high/unclear) | Unclear | Low | Unclear | Low | Unclear |
| **B. Applicability** |  |  |  |  |  |
| Concern that included participants and setting do not match review question (low/high/unclear) | Low | Low | Low | Low | Low |
| **Domain 2** |  |  |  |  |  |
| **A. Risk of bias - predictors** |  |  |  |  |  |
| 2.1 Were predictors defined and evaluated in a similar way for all participants? | Yes | Yes | Yes | Yes | Yes |
| 2.2 Were predictor evaluations performed without knowing the outcome data? | Yes | Yes | Yes | Yes | Yes |
| 2.3 Are all the predictors available at the time the model is intended to be used? | Yes | Yes | Yes | Yes | Yes |
| Risk of bias introduced by the predictors or their assessment (low/high/unclear) | Low | Low | Low | Low | Low |
| **B. Applicability** |  |  |  |  |  |
| Concern that the definition, evaluation, or timing of predictors in the model do not match the review question (low/high/unclear) | Low | Low | Low | Low | Low |
| **Domain 3** |  |  |  |  |  |
| **A. Risk of bias - outcome** |  |  |  |  |  |
| Was the result properly determined? | Yes | Yes | Yes | Yes | Yes |
| Was a standard or prespecified outcome definition used? | Yes | Yes | Yes | Yes | Yes |
| Were predictors excluded from the outcome definition? | Yes | Yes | Yes | Yes | Yes |
| Was the outcome similarly defined and determined for all participants? | Yes | Yes | Yes | Yes | Yes |
| Was the outcome determined without knowing the predictor information? | Yes | Yes | Yes | Yes | Yes |
| Was the time interval between the evaluation of the predictor and the determination of the result adequate? | Yes | Yes | Yes | Yes | Yes |
| Risk of bias introduced by the result or its determination (low/high/unclear) | Low | Low | Low | Low | Low |
| **B. Applicability** |  |  |  |  |  |
| If a composite outcome was used, please describe the relative frequency/distribution of each contributing outcome: | Yes | Yes | Yes | Yes | Yes |
| Concern that the outcome, its definition, timing, or determination does not match the review question (low/high/unclear) | Low | Low | Low | Low | Low |
| **Domain 4** |  |  |  |  |  |
| **A. Risk of bias** |  |  |  |  |  |
| Describe the number of participants, number of candidate predictors, outcome events, and events per candidate predictor: | Yes | Yes | Yes | Yes | Yes |
| Describe how the model was developed (for example, with respect to modeling technique (eg, survival or logistic modeling), selection of predictors, and definition of risk groups): | Yes | Yes | Yes | Yes | Yes |
| Describe if and how the model was validated, either internally (eg, bootstrapping, cross-validation, split random sample) or externally (eg, temporal validation, geographic validation, different setting, different type of participants): | Yes | Yes | Yes | Yes | Yes |
| Describe the performance measures of the model, e.g. (re)calibration, discrimination, (re)classification, net profit and if adjusted for optimism: | Yes | Yes | Yes | Yes | Yes |
| Describe the participants who were excluded from the analysis: | Yes | Yes | Yes | Yes | Yes |
| Describe missing data on predictors and outcomes, as well as methods used for missing data: | Yes | Yes | Yes | Yes | Yes |
| 4.1 Was there a reasonable number of participants with the result? | Yes | Yes | Yes | Yes | Yes |
| 4.2 Were continuous and categorical predictors handled correctly? | Yes | Yes | Yes | Yes | Yes |
| 4.3 Were all enrolled participants included in the analysis? | Yes | Yes | Yes | Yes | Yes |
| 4.4 Were participants with missing data treated appropriately? | NA | NA | NA | NA | NA |
| 4.5 Was selection of predictors based on univariate analysis avoided? | Yes | Yes | Yes | Yes | Yes |
| 4.6 Were the complexities of the data (eg censoring, competing risks, sampling of controls) adequately accounted for? | Yes | Yes | Yes | Yes | Yes |
| 4.7 Were relevant model performance measures adequately evaluated? | Yes | Yes | Yes | Yes | Yes |
| 4.8 Was model overfitting and optimism factored into model performance? | Yes | Yes | Yes | Yes | Yes |
| 4.9 Do the predictors and their assigned weights in the final model correspond to the results of the multivariate analysis? | Yes | Yes | Yes | Yes | Yes |
| **Risk of bias introduced by the analysis (low/high/Unclear)** | Low | Low | Low | Low | Low |
| **Step 4: General evaluation** | There is not enough information related to the chosen population, so there is a risk of introducing bias in the selection | - | There is no clear information on the population | - | There is no clear information on the population |
| **Overall assessment of risk of bias** | Unclear | Low | Unclear | Low | Unclear |
| **General judgment of applicability** | Low | Low | Low | Low | Low |
|  |  |  |  |  |  |
|  |  |  |  |  |  |
| **Domain 1** | **Zhao et al. (2021) (2)** | **Zhao et al. (2021) (3)** | **Zhao et al. (2021) (4)** | **Zhao et al. (2021) (5)** | **Zhou et al. (2021)** |
| **A. Risk of bias** | **10-08-22** | **10-08-22** | **10-08-22** | **10-08-22** | **10-08-22** |
| 1.1 Were appropriate data sources used, e.g. data from cohort studies, RCTs or nested case-controls? | Yes | Yes | Yes | Yes | Yes |
| 1.2 Were all inclusions and exclusions of participants appropriate? | Yes | Yes | Yes | Yes | Yes |
| Risk of bias introduced by participant selection (low/high/unclear) | Low | Low | Low | Low | Low |
| **B. Applicability** |  |  |  |  |  |
| Concern that included participants and setting do not match review question (low/high/unclear) | Low | Low | Low | Low | Low |
| **Domain 2** |  |  |  |  |  |
| **A. Risk of bias - predictors** |  |  |  |  |  |
| 2.1 Were predictors defined and evaluated in a similar way for all participants? | Yes | Yes | Yes | Yes | Yes |
| 2.2 Were predictor evaluations performed without knowing the outcome data? | Yes | Yes | Yes | Yes | Yes |
| 2.3 Are all the predictors available at the time the model is intended to be used? | Yes | Yes | Yes | Yes | Yes |
| Risk of bias introduced by the predictors or their assessment (low/high/unclear) | Low | Low | Low | Low | Low |
| **B. Applicability** |  |  |  |  |  |
| Concern that the definition, evaluation, or timing of predictors in the model do not match the review question (low/high/unclear) | Low | Low | Low | Low | Low |
| **Domain 3** |  |  |  |  |  |
| **A. Risk of bias - outcome** |  |  |  |  |  |
| Was the result properly determined? | Yes | Yes | Yes | Yes | Yes |
| Was a standard or prespecified outcome definition used? | Yes | Yes | Yes | Yes | Yes |
| Were predictors excluded from the outcome definition? | Yes | Yes | Yes | Yes | Yes |
| Was the outcome similarly defined and determined for all participants? | Yes | Yes | Yes | Yes | Yes |
| Was the outcome determined without knowing the predictor information? | Yes | Yes | Yes | Yes | Yes |
| Was the time interval between the evaluation of the predictor and the determination of the result adequate? | Yes | Yes | Yes | Yes | Yes |
| Risk of bias introduced by the result or its determination (low/high/unclear) | Low | Low | Low | Low | Low |
| **B. Applicability** |  |  |  |  |  |
| If a composite outcome was used, please describe the relative frequency/distribution of each contributing outcome: | Yes | Yes | Yes | Yes | Yes |
| Concern that the outcome, its definition, timing, or determination does not match the review question (low/high/unclear) | Low | Low | Low | Low | Low |
| **Domain 4** |  |  |  |  |  |
| **A. Risk of bias** |  |  |  |  |  |
| Describe the number of participants, number of candidate predictors, outcome events, and events per candidate predictor: | Yes | Yes | Yes | Yes | Yes |
| Describe how the model was developed (for example, with respect to modeling technique (eg, survival or logistic modeling), selection of predictors, and definition of risk groups): | Yes | Yes | Yes | Yes | Yes |
| Describe if and how the model was validated, either internally (eg, bootstrapping, cross-validation, split random sample) or externally (eg, temporal validation, geographic validation, different setting, different type of participants): | Yes | Yes | Yes | Yes | Yes |
| Describe the performance measures of the model, e.g. (re)calibration, discrimination, (re)classification, net profit and if adjusted for optimism: | Yes | Yes | Yes | Yes | Yes |
| Describe the participants who were excluded from the analysis: | Yes | Yes | Yes | Yes | Yes |
| Describe missing data on predictors and outcomes, as well as methods used for missing data: | Yes | Yes | Yes | Yes | Yes |
| 4.1 Was there a reasonable number of participants with the result? | Yes | Yes | Yes | Yes | Yes |
| 4.2 Were continuous and categorical predictors handled correctly? | Yes | Yes | Yes | Yes | Yes |
| 4.3 Were all enrolled participants included in the analysis? | Yes | Yes | Yes | Yes | Yes |
| 4.4 Were participants with missing data treated appropriately? | NA | NA | NA | NA | NA |
| 4.5 Was selection of predictors based on univariate analysis avoided? | Yes | Yes | Yes | Yes | Yes |
| 4.6 Were the complexities of the data (eg censoring, competing risks, sampling of controls) adequately accounted for? | Yes | Yes | Yes | Yes | Yes |
| 4.7 Were relevant model performance measures adequately evaluated? | Yes | Yes | Yes | Yes | Yes |
| 4.8 Was model overfitting and optimism factored into model performance? | Yes | Yes | Yes | Yes | Yes |
| 4.9 Do the predictors and their assigned weights in the final model correspond to the results of the multivariate analysis? | Yes | Yes | Yes | Yes | Yes |
| **Risk of bias introduced by the analysis (low/high/Unclear)** | Low | Low | Low | Low | Low |
| **Step 4: General evaluation** | **-** | **-** | **-** | **-** | **-** |
| **Overall assessment of risk of bias** | Low | Low | Low | Low | Low |
| **General judgment of applicability** | Low | Low | Low | Low | Low |
|  |  |  |  |  |  |
|  |  |  |  |  |  |
| **Domain 1** | **Suan Zhu et al. (2022)** | **Zia et al. (2021)** | **Zou et al. (2021)** | **Zou et al. (2022)** |  |
| **A. Risk of bias** | **10-08-22** | **10-08-22** | **10-08-22** | **10-08-22** |  |
| 1.1 Were appropriate data sources used, e.g. data from cohort studies, RCTs or nested case-controls? | Yes | Unclear | Unclear | Yes |  |
| 1.2 Were all inclusions and exclusions of participants appropriate? | Yes | Unclear | Unclear | Yes |  |
| Risk of bias introduced by participant selection (low/high/unclear) | Low | Unclear | Unclear | Low |  |
| **B. Applicability** |  |  |  |  |  |
| Concern that included participants and setting do not match review question (low/high/unclear) | Low | Low | Low | Low |  |
| **Domain 2** |  |  |  |  |  |
| **A. Risk of bias - predictors** |  |  |  |  |  |
| 2.1 Were predictors defined and evaluated in a similar way for all participants? | Yes | Yes | Yes | Yes |  |
| 2.2 Were predictor evaluations performed without knowing the outcome data? | Yes | Yes | Yes | Yes |  |
| 2.3 Are all the predictors available at the time the model is intended to be used? | Yes | Yes | Yes | Yes |  |
| Risk of bias introduced by the predictors or their assessment (low/high/unclear) | Low | Low | Low | Low |  |
| **B. Applicability** |  |  |  |  |  |
| Concern that the definition, evaluation, or timing of predictors in the model do not match the review question (low/high/unclear) | Low | Low | Low | Low |  |
| **Domain 3** |  |  |  |  |  |
| **A. Risk of bias - outcome** |  |  |  |  |  |
| Was the result properly determined? | Yes | Yes | Yes | Yes |  |
| Was a standard or prespecified outcome definition used? | Yes | Yes | Yes | Yes |  |
| Were predictors excluded from the outcome definition? | Yes | Yes | Yes | Yes |  |
| Was the outcome similarly defined and determined for all participants? | Yes | Yes | Yes | Yes |  |
| Was the outcome determined without knowing the predictor information? | Yes | Yes | Yes | Yes |  |
| Was the time interval between the evaluation of the predictor and the determination of the result adequate? | Yes | Yes | Yes | Yes |  |
| Risk of bias introduced by the result or its determination (low/high/unclear) | Low | Low | Low | Low |  |
| **B. Applicability** |  |  |  |  |  |
| If a composite outcome was used, please describe the relative frequency/distribution of each contributing outcome: | Yes | Yes | Yes | Yes |  |
| Concern that the outcome, its definition, timing, or determination does not match the review question (low/high/unclear) | Low | Low | Low | Low |  |
| **Domain 4** |  |  |  |  |  |
| **A. Risk of bias** |  |  |  |  |  |
| Describe the number of participants, number of candidate predictors, outcome events, and events per candidate predictor: | Yes | Yes | Yes | Yes |  |
| Describe how the model was developed (for example, with respect to modeling technique (eg, survival or logistic modeling), selection of predictors, and definition of risk groups): | Yes | Yes | Yes | Yes |  |
| Describe if and how the model was validated, either internally (eg, bootstrapping, cross-validation, split random sample) or externally (eg, temporal validation, geographic validation, different setting, different type of participants): | Yes | Yes | Yes | Yes |  |
| Describe the performance measures of the model, e.g. (re)calibration, discrimination, (re)classification, net profit and if adjusted for optimism: | Yes | Yes | Yes | Yes |  |
| Describe the participants who were excluded from the analysis: | Yes | Yes | Yes | Yes |  |
| Describe missing data on predictors and outcomes, as well as methods used for missing data: | Yes | Yes | Yes | Yes |  |
| 4.1 Was there a reasonable number of participants with the result? | Yes | Yes | Yes | Yes |  |
| 4.2 Were continuous and categorical predictors handled correctly? | Yes | Yes | Yes | Yes |  |
| 4.3 Were all enrolled participants included in the analysis? | Yes | Yes | Yes | Yes |  |
| 4.4 Were participants with missing data treated appropriately? | NA | NA | NA | NA |  |
| 4.5 Was selection of predictors based on univariate analysis avoided? | Yes | Yes | Yes | Yes |  |
| 4.6 Were the complexities of the data (eg censoring, competing risks, sampling of controls) adequately accounted for? | Yes | Yes | Yes | Yes |  |
| 4.7 Were relevant model performance measures adequately evaluated? | Yes | Yes | Yes | Yes |  |
| 4.8 Was model overfitting and optimism factored into model performance? | Yes | Yes | Yes | Yes |  |
| 4.9 Do the predictors and their assigned weights in the final model correspond to the results of the multivariate analysis? | Yes | Yes | Yes | Yes |  |
| **Risk of bias introduced by the analysis (low/high/Unclear)** | Low | Low | Low | Low |  |
| **Step 4: General evaluation** | **-** | There is no clear information on the population | There is no clear information on the population | **-** |  |
| **Overall assessment of risk of bias** | Low | Unclear | Unclear | Low |  |
| **General judgment of applicability** | Low | Low | Low | Low |  |
